# Haemoglobin thresholds to define anaemia from age 6 months to 65 years: estimates from international data sources

**DOI:** 10.1016/S2352-3026(24)00030-9

**Published:** 2024-02-29

**Authors:** Sabine Braat, Katherine L Fielding, Jiru Han, Victoria E Jackson, Sophie Zaloumis, Jessica Xu Hui Xu, Gemma Moir-Meyer, Sophia M Blaauwendraad, Vincent W V Jaddoe, Romy Gaillard, Patricia C Parkin, Cornelia M Borkhoff, Charles D G Keown-Stoneman, Catherine S Birken, Jonathon L Maguire, Genes & Health Research Team, Melanie Bahlo, Eliza M Davidson, Sant-Rayn Pasricha

**Affiliations:** a Population Health and Immunity Division, The Walter and Eliza Hall Institute of Medical Research, Melbourne, VIC, Australia; b Methods and Implementation Support for Clinical and Health research Hub, Faculty of Medicine, Dentistry and Health Sciences, The University of Melbourne, Melbourne, VIC, Australia; c Medical Biology, Faculty of Medicine, Dentistry and Health Sciences, The University of Melbourne, Melbourne, VIC, Australia; d Clinical Haematology, The Austin Hospital, Heidelberg, VIC, Australia; e Generation R Study Group, and Department of Pediatrics, Erasmus University Medical Center, Rotterdam, Netherlands; f Division of Pediatric Medicine and the Pediatric Outcomes Research Team, The Hospital for Sick Children, Toronto, ON, Canada; g Dalla Lana School of Public Health, University of Toronto, Toronto, ON, Canada; h Unity Health Toronto, Toronto, ON, Canada; i Wolfson Institute of Population Health and Blizard Institute, Barts and the London School of Medicine and Dentistry, Queen Mary University of London, London, UK; j Diagnostic Haematology, The Royal Melbourne Hospital, Parkville, VIC, Australia; k Clinical Haematology, Peter MacCallum Cancer Centre and The Royal Melbourne Hospital, Parkville, VIC, Australia

## Abstract

**Background:**

Detection of anaemia is crucial for clinical medicine and public health. Current WHO anaemia definitions are based on statistical thresholds (fifth centiles) set more than 50 years ago. We sought to establish evidence for the statistical haemoglobin thresholds for anaemia that can be applied globally and inform WHO and clinical guidelines.

**Methods:**

In this analysis we identified international data sources from populations in the USA, England, Australia, China, the Netherlands, Canada, Ecuador, and Bangladesh with sufficient clinical and laboratory information collected between 1998 and 2020 to obtain a healthy reference sample. Individuals with clinical or biochemical evidence of a condition that could reduce haemoglobin concentrations were excluded. We estimated haemoglobin thresholds (ie, 5th centiles) for children aged 6–23 months, 24–59 months, 5–11 years, and 12–17 years, and adults aged 18–65 years (including during pregnancy) for individual datasets and pooled across data sources. We also collated findings from three large-scale genetic studies to summarise genetic variants affecting haemoglobin concentrations in different ancestral populations.

**Findings:**

We identified eight data sources comprising 18 individual datasets that were eligible for inclusion in the analysis. In pooled analyses, the haemoglobin fifth centile was 104·4 g/L (90% CI 103·5–105·3) in 924 children aged 6–23 months, 110·2 g/L (109·5–110·9) in 1874 children aged 24–59 months, and 114·4 g/L (113·6–115·2) in 1839 children aged 5–11 years. Values diverged by sex in adolescents and adults. In pooled analyses, the fifth centile was 122·2 g/L (90% CI 121·3–123·1) in 1741 female adolescents aged 12–17 years and 128·2 g/L (126·4–130·0) in 1103 male adolescents aged 12–17 years. In pooled analyses of adults aged 18–65 years, the fifth centile was 119·7 g/L (90% CI 119·1–120·3) in 3640 non-pregnant females and 134·9 g/L (134·2–135·6) in 2377 males. Fifth centiles in pregnancy were 110·3 g/L (90% CI 109·5–111·0) in the first trimester (n=772) and 105·9 g/L (104·0–107·7) in the second trimester (n=111), with insufficient data for analysis in the third trimester. There were insufficient data for adults older than 65 years. We did not identify ancestry-specific high prevalence of non-clinically relevant genetic variants that influence haemoglobin concentrations.

**Interpretation:**

Our results enable global harmonisation of clinical and public health haemoglobin thresholds for diagnosis of anaemia. Haemoglobin thresholds are similar between sexes until adolescence, after which males have higher thresholds than females. We did not find any evidence that thresholds should differ between people of differering ancestries.

**Funding:**

World Health Organization and the Bill & Melinda Gates Foundation.

## Introduction

Anaemia develops when the red cell mass is insufficient to meet physiological oxygen-carrying needs and is usually formally diagnosed when the haemoglobin concentration falls below a defined threshold for age and sex.^1^ Accurate case definition of anaemia is crucial for clinical diagnosis and treatment, and for understanding the magnitude and distribution of this condition as a public health problem. WHO's anaemia definitions aim to reflect the lower fifth centile of the haemoglobin distribution of a reference population of healthy individuals. WHO thresholds were initially proposed in 1958,^2^ then updated in 1968,^3^ and have remained essentially unchanged since.^4^ WHO thresholds were based on studies with restricted measurement of biomarkers of iron and other forms of haematinic deficiency and inflammation. There is limited consensus on anaemia definitions,^5^ leading to heterogeneous definitions between sources, expert groups, and public health bodies,^6^ translating into inconsistent clinical definitions.^7^

Establishing a valid diagnosis of anaemia is crucial for treating patients and detecting the range of diseases that could underlie this condition.^8^ Anaemia can cause clinical effects directly (eg, anaemia causes a substantial burden of disease); however, even in the absence of overt clinical effects, haemoglobin-based thresholds for anaemia are used as important triggers for investigation and optimisation of underlying contributors. Haemoglobin concentrations are reduced by acute, recurrent, chronic medical or surgical illness. Inflammation (eg, due to autoimmune disease, infection, cancer, heart failure, and even obesity) suppresses erythropoiesis due to functional iron deficiency and might also reduce red cell survival.^8^ Haemoglobin concentrations are reduced in renal impairment due to reduced erythropoietin production and functional iron deficiency. Many medications can reduce haemoglobin concentrations idiosyncratically or in a dose–dependent manner. Anaemia can persist weeks or months beyond an acute illness^9^, ^10^ and normalisation of acute inflammatory markers. Conversely, hypoxia (eg, smoking, respiratory or cardiac disease, obesity or sleep apnoea, or elevated altitude of residence)^11^ can increase haemoglobin concentrations. Diagnosis of anaemia lies at the centre of many clinical pathways, such as investigation of gastrointestinal bleeding,^12^ preoperative optimisation,^13^ screening for haemoglobinopathy,^14^ and eligibility for blood donation.^15^ Anaemia is an adverse prognostic factor for multiple conditions, including heart failure,^16^ major surgery,^17^ cancer,^18^ and HIV.^19^ Most patients admitted to hospital with critical illness are anaemic in hospital, and about half remain anaemic 6 months^20^ or even 12 months after hospital admission.^9^

**Research in context**

**Evidence before this study**

WHO's haemoglobin thresholds to define anaemia are statistical thresholds reflecting the fifth centile of haemoglobin distribution in apparently healthy populations, set more than 50 years ago and maintained since then. Before undertaking the current analysis, we first evaluated available documentation of the source of these recommendations: the thresholds were defined on the basis of five small studies, which were restricted in terms of geographical location, epidemiological design, sample size, and laboratory evaluation of iron stores and inflammation as well as other haematinic biomarkers, and transparency of the statistical analysis. These studies included minimal data from women and did not include data from children or infants. Next, we searched for studies that had sought to evaluate thresholds, using a 2019 WHO-commissioned review as an initial resource. This review summarised results from 60 studies conducted globally between 1975 and 2018, and noted markedly heterogenous results, with studies inconsistently excluding individuals with iron deficiency or inflammation, and a recommendation to conduct studies with more rigorous inclusion criteria. More recently, a post-hoc analysis of international datasets from population nutrition surveys through the BRINDA (Biomarkers Reflecting Inflammation and Nutritional Determinants of Anemia) Collaboration illustrated the constraints of the use of population haematology (collected with a mixture of capillary and venous sampling) and biochemical data with insufficient clinical information, showing an approximately 33 g/L variation in fifth centile of haemoglobin concentration across different countries (eg, finding a threshold for young children aged 6–59 months ranging from approximately 79 g/L in Pakistan and Cote D'Ivoire to approximately 113 g/L in the USA; and a threshold in non-pregnant women ranging from 88 g/L in India to 121 g/L in the USA). An analysis based on data from a national nutrition survey conducted in India also suggested thresholds lower than WHO recommendations in children and adolescents.

**Added value of this study**

In this analysis, we identified and used datasets that enabled exclusion of clinical conditions that could influence haemoglobin concentrations, along with biochemical measures of iron status, inflammation, and, where possible, renal and other organ function, to create reference populations of healthy individuals from which the fifth centile of the haemoglobin distribution could be estimated. Our data include children aged 6 months to 18 years, and adults aged up to 65 years. Our results suggest relatively consistent haemoglobin thresholds across diverse populations, including from south and east Asia, and reveal minimal secular variation within countries even as populations (and reasons for exclusion) changed over recent decades. We also summarised findings from three existing large-scale, multi-ancestry, genome-wide association studies to search for non-clinically relevant, high-frequency genetic variants that might influence haemoglobin concentrations in populations of different ancestries, and that could hence justify varied haemoglobin thresholds between different ancestries.

**Implications of all the available evidence**

Haemoglobin thresholds defining anaemia in representative healthy individuals vary across the lifecycle and differ by sex in people aged 12–65 years. Additionally, our analyses of genetic variants indicate that haemoglobin thresholds for anaemia should not be adjusted for individuals of different ancestry. Our analyses provide a single set of thresholds that can be applied globally in clinical practice and public health.

Valid definitions of anaemia are also crucial for guiding population interventions and tracking progress towards global targets. Based on current definitions, anaemia affects 40% of all preschool children and 30% of women, including 46% of pregnant women globally,^21^ and accounts for 52 million years lived with disability.^22^ Reducing anaemia prevalence in women by 50% is a WHO global nutrition target^23^ and a sub-indicator of the UN Sustainable Development Goals.^24^ Interventions that directly (eg, iron supplementation^25^ or fortification^26^) or indirectly (eg, malaria control) reduce anaemia are recommended for many low-income countries, with implementation decisions based on anaemia prevalence.^27^

Haemoglobin thresholds to define anaemia usually vary between men and women, although this distinction has been challenged.^28^, ^29^ Thresholds can also vary in children and during pregnancy. Haemoglobin concentrations can be lower in African^5^, ^30^ or Asian populations,^31^ but it is unclear whether this discrepancy reflects clinically significant genetic conditions affecting red cells, undefined clinical illnesses, or a different underlying baseline driven by non-clinical genetic variation.^30^

WHO has recognised the need for further evidence for haemoglobin thresholds to define anaemia, including the influence of genetic factors (both carrier states for rare genetic conditions such as thalassaemias and haemoglobinopathies, and the role of genetics in overall variation),^32^ in order to update global guidelines. Here, we present an analysis utilising multiple large-scale datasets in ethnically diverse populations that included sufficiently detailed clinical and laboratory parameters to enable post-hoc assembly of reference populations of healthy individuals without discernible risk factors for anaemia, in which the fifth centile of haemoglobin distribution can be estimated across the lifecycle. Additionally, we summarised ancestry-specific allele frequencies and effects on haemoglobin concentrations of genetic polymorphisms and structural variants identified through existing large-scale, multi-ancestry genome-wide association studies (GWAS). We aimed to provide a rationale for a set of thresholds that can be applied globally for clinical and population use.

## Methods

### Study design and data sources

To support clinical and public health guidelines we sought to establish thresholds for children, adolescents, adult men, and adult women including pregnant women (by trimester). We aimed to define the lower fifth centile of the distribution among healthy individuals (ie, a healthy reference population). Central to the design of the analysis was the need to establish a reference population from existing datasets (posteriori approach) through exclusion of individuals with evidence of conditions or circumstances that could influence haemoglobin concentration.^33^ We searched for datasets comprising appropriate clinical and laboratory information to enable post-hoc derivation of an apparently healthy reference sample; this approach included assessing national health surveys (using the search terms “country name” AND “national health database”) and prospective clinical cohorts, with a focus on countries where malaria was not endemic (ie, with low background inflammation) between May, 2021, and September, 2022 (further details are provided in the appendix, p 5).

Although exclusion criteria were standardised, the available data and their coding varied between studies (eg, in classification of ancestry, consumption of alcohol, terminology, or questions about history of illness). Studies were only selected if both clinical and laboratory data were available. The specific nature of clinical data varied between surveys, and within each survey all available clinical parameters relevant to anaemia were used. This approach resulted in heterogeneous exclusion criteria between surveys, but the optimal healthy population within each survey. Clinical criteria for exclusion included reporting of chronic systemic medical illness; any recent acute illness (2–4 weeks); recent hospital admission (up to 12 months); use of medications (excluding hormonal contraception where possible); current smoking or excess alcohol consumption; and obesity or low bodyweight (ascertained by BMI cutoffs). Where data were available, we excluded individuals living at elevations above 750 m. Missing clinical criteria were not applied as exclusions, except for pregnancy.

Laboratory criteria were required for all participants; those missing data for haemoglobin, ferritin, or C-reactive protein (CRP) were excluded from the analysis. Haemoglobin measurements were conducted on venous blood by use of an automated analyser or high-quality point-of-care device (table). Ferritin concentrations were applied as per WHO guidelines for the use of ferritin, defining iron deficiency as ferritin concentrations lower than 12 μg/L in children younger than 5 years, and ferritin concentrations lower than 15 μg/L in all other groups.^34^ CRP higher than 5 mg/mL was used to indicate inflammation; individuals with elevated ferritin (as per WHO guidelines) were also excluded since this could portend inflammation or liver disease.^35^ Where available, other markers of health were used on the basis of consensus reference ranges. Sensitivity analyses included raising the ferritin threshold to define iron deficiency or lowering the CRP threshold to define inflammation, or restricting mean cell volume (in children and adolescents only), or a combination of the above. Detailed inclusion and exclusion of individuals from each dataset to obtain the reference sample are shown in the appendix (pp 6–9).

**Table:** Summary of data sources and demographics

	**Design**	**Location**	**Study period**	Population (sample size*; sample size final reference population)	**Blood sample**	**Haemoglobin testing method**
Australian Health Survey (AHS)	National population health survey	Australia	2011–12	Adults aged 18–65 years (N=7262; N=1268)Adolescents aged 12–17 years (N=734; N=380)	Non-fasting venous	Sysmex XE-2100
Benefits and Risks of Iron interventions in Children (BRISC)	Randomised controlled trial	Bangladesh	2017–20	Children aged 11 months (N=1365†; N=255)	Non-fasting venous	Hemocue 301+
China Health and Nutrition Survey (CHNS)	National population health survey	China‡	2009	Adults aged 18–65 years (N=7172; N=796)Children and adolescents aged 5–17 years (N=814; N=475)	Non-fasting venous	Sysmex XE-2100
Encuesta Nacional de Salud y Nutrición (ENSANUT)	National population health survey	Ecuador‡	2011–13	Children aged 6–59 months (N=2047; N=275)	Non-fasting venous	Sysmex XE-2100
Generation R	Prospective cohort study	Netherlands	2002–06	Pregnant women aged 18–45 years (N=7487; N=883)	Non-fasting venous	Automated Laboratory Analyzer§
Health Survey for England (HSE)	National population health survey	England	1998, 2006, 2009	Adults aged 18–65 years (N=15 315; N=2290)	Non-fasting venous	Coulter STKS, Abbott CD 4000
National Health and Nutrition Examination Survey (NHANES)	National population health survey	USA	1999–2000, 2001–02, 2003–04, 2005–06, 2007–08, 2009–10, 2015–16, 2017–18	Adults aged 18–65 years (N=19 346; N=1663)Children and adolescents aged 6 months to 17 years (N=12 883; N=4524)	Non-fasting venous	Beckman-Coulter analysers
TARGet Kids!	Open longitudinal cohort study of healthy children	Canada	2010–19	Children aged 6 months to 11 years (N=4700¶; N=1572)	Non-fasting venous	Sysmex XN-9000

CRP=C-reactive protein. The Australian Health Survey consists of the National Health Survey (NHS) and National Nutrition and Physical Activity Survey (NNPAS). The BRISC trial is registered on the Australian New Zealand Clinical Trials Registry (ACTRN12617000660381). The Generation R study is registered with the Dutch Trial Register (NL6484). The TARGet Kids! study is registered with ClinicalTrials.gov (NCT01869530). All data sources included male and female participants (apart from Generation R, which only included pregnant women).

* Defined as those within the age range of the populations (eg, aged 18–65 years) and with non-missing haemoglobin, ferritin, and CRP concentrations except for Generation R defined as those with non-missing haemoglobin.

† Additionally restricted to those who received an iron intervention.

‡ Exclusions applied to those who resided above 750 m altitude (ENSANUT) or residents of province (Guizhou) due to altitude above 750 m (CHNS).

§ Specific analyser unavailable; automated laboratory haematology instrument.

¶ Number of unique participants.

We sought to ensure ethnic diversity but recognised that population field surveys in low-income settings can contain a high burden of unreported, undetected, or recently resolved inflammation even if acute biomarkers of inflammation had normalised, thus preventing exclusion of individuals with recent illness that might have lowered haemoglobin concentration unless detailed clinical data were included.^36^ As recommended, we recalculated estimated glomerular filtration rates with non-race-based equations.^37^

We sought to establish whether different ancestries have non-clinically relevant high-frequency genetic variations that influence haemoglobin concentration; the absence of such variants would enable a single set of thresholds. Thus, in a separate study to the determination of the fifth centile of the reference population described above, we used ancestry-specific databases to interrogate genome-wide associations, effect sizes, and minor allele frequencies of single-nucleotide polymorphisms (SNPs) and structural variants associated with haemoglobin concentration. Full details are available in the appendix (pp 22–23).

Each included study had received ethics approval and obtained informed consent from participants (appendix p 25); use of these datasets for these analyses was deemed by Walter and Eliza Hall Institute of Medical Research (WEHI) Governance, Risk and Compliance to meet the criteria for exemption from ethics review.

### Statistical analysis

Lower 2·5th and fifth centiles were estimated and two-sided confidence intervals (CIs) obtained with the methodology described below and detailed in the appendix (pp 16–22). The Clinical & Laboratory Standards Institute recommends using a 90% CI.^38^ We estimated discrete thresholds (based on conventionally applied age categories) for each data source and continuous thresholds (based on the participant's age [and sex if applicable] within age categories, except for pregnant women) across all data sources. For each threshold, the sample size was determined by the number of participants who fulfilled the criteria of an apparently healthy individual among those with a non-missing haemoglobin, ferritin, and CRP value. Outlying values were identified with Tukey's method,^39^ and the results presented here are those after excluding outliers from the reference sample.

For discrete thresholds, the assumption of normality was checked and the parametric theoretical centile of a Gaussian distribution, with corresponding 90% CI, was used to estimate the centile.^40^ For the national population health surveys, we aimed to account for the survey design by estimating survey-weighted centiles calculated with survey-weighted quantile regression. The implementation of survey-weighted quantile regression proceeded as described for continuous thresholds below, except the model only included a single intercept term. Standard errors for the intercept term were derived from 1000 Canty and Davidson's bootstrap replicates and used to calculate 90% CIs assuming a normal approximation.^41^, ^42^ As a sensitivity analysis, unweighted centile estimates were also calculated with the parametric theoretical centile described earlier. Discrete centile estimates were pooled across all data sources through a fixed-effects meta-analysis, with a random-effects meta-analysis as a sensitivity analysis, and presented in forest plots.

For continuous thresholds, we used the Hoq^43^ method to estimate age-specific (and by sex, if applicable) continuous haemoglobin thresholds within each age category by combining individual data of all relevant data sources. This approach involved identifying the best fitting multivariable fractional polynomial model for the mean values of haemoglobin by use of Royston's method^44^ followed by a likelihood ratio test for an interaction between sex and the fractional polynomial representation of age. An age-by-sex interaction term was included in the model if it was statistically significant at the nominal significance level (p<0·05). Quantile regression was then used; no survey weighting was applied to the national population health surveys. Age-dependent haemoglobin predictions were obtained from the unweighted quantile regression model and bootstrap percentile 90% CIs generated from 1000 bootstrap samples. Results were presented in plots to indicate how predicted percentiles change with age (and by sex, if applicable), superimposed onto a scatter plot of haemoglobin versus age within each age category.

Reference samples were derived with Stata (version 16.1), R (version 4.2.0, Generation R), or Stata (version 17.1) for datasets from Australia's National Health Survey (NHS) and Australia's National Nutrition and Physical Activity Survey (NNPAS). Haemoglobin thresholds were estimated with R (version 4.1.1 and version 4.2.0 [Generation R]), or Stata (version 17.1) for datasets from the NHS and NNPAS. Details of the packages used, including those for the genetic analysis, are provided in the appendix (pp 23–25).

### Role of the funding source

The funders of the study had no role in study design, data collection, data analysis, data interpretation, writing of the report, or the decision to submit the manuscript for publication.

## Results

**Figure 1:**
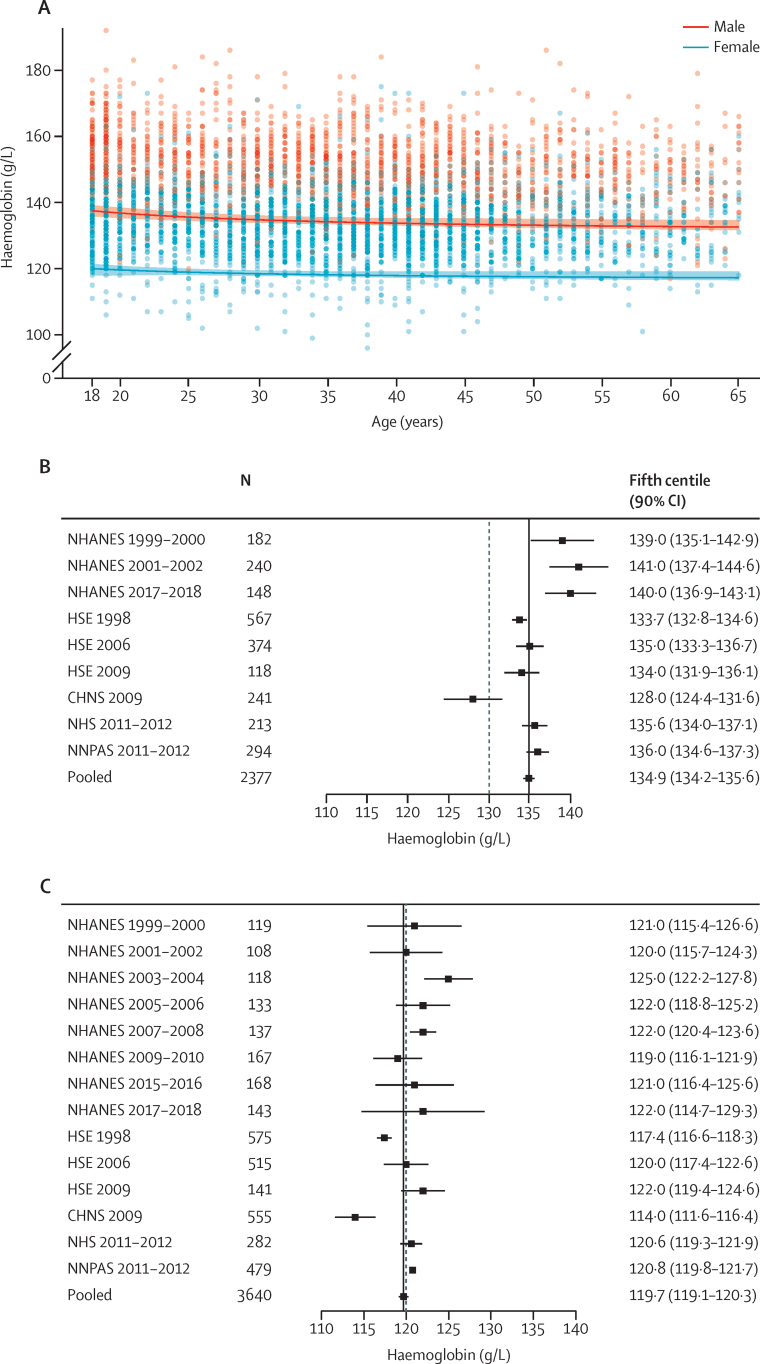
Derivation of haemoglobin thresholds in males and non-pregnant females aged 18–65 years (A) Continuous thresholds in adult males aged 18–65 years and non-pregnant females aged 18–65 years pooled across the following datasets: NHANES, HSE, and CHNS. The pooled continuous centiles and 90% CIs across data sources were estimated without accounting for the complex survey design and weighting. (B) Individual study and pooled (fixed effect, black line) estimates of the fifth centile of the haemoglobin distribution in the healthy reference population in male adults aged 18–65 years. (C) Individual study and pooled (fixed effect, black line) estimates of the fifth centile of the haemoglobin distribution in the healthy reference population in non-pregnant female adults aged 18–65 years. The dashed line in panels B and C represents the cutoff for anaemia according to the WHO guideline.^4^ CHNS=China Health and Nutrition Survey. HSE=Health Survey for England. NHANES=National Health and Nutrition Examination Survey (USA). NHS=National Health Survey (Australia). NNPAS=National Nutrition and Physical Activity Survey (Australia).

**Figure 2:**
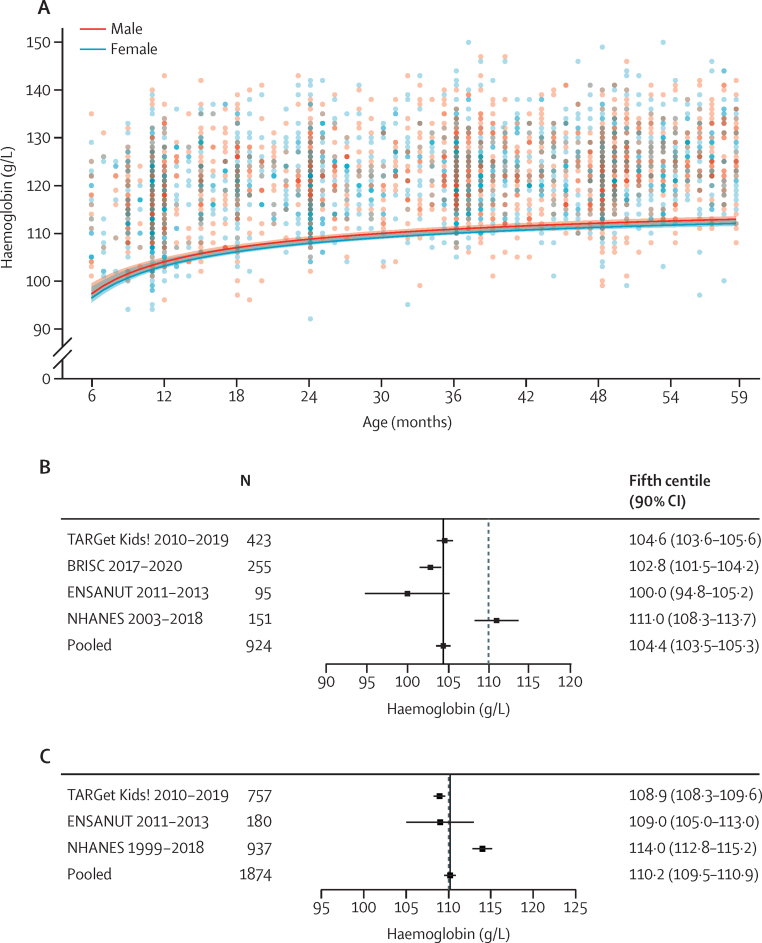
Derivation of haemoglobin thresholds in children aged 6–59 months (A) Continuous thresholds in male and female children aged 6–59 months pooled across the following datasets: TARGet Kids!, BRISC, ENSANUT, and NHANES. The pooled continuous centiles and 90% CIs across data sources were estimated without accounting for the complex survey design and weighting. (B) Individual study and pooled (fixed effect, black line) estimates of the fifth centile of the haemoglobin distribution in the healthy reference population in children aged 6–23 months. (C) Individual study and pooled (fixed effect, black line) estimates of the fifth centile of the haemoglobin distribution in the healthy reference population in children aged 24–59 months. The dashed line in panels B and C represents the cutoff for anaemia according to the WHO guideline.^4^ BRISC=Benefits and Risks of Iron interventions in Children (Bangladesh). ENSANUT=Encuesta Nacional de Salud y Nutrición (Ecuador). NHANES=National Health and Nutrition Examination Survey (USA). TARGet Kids!=Applied Research Group for Kids (Canada).

**Figure 3:**
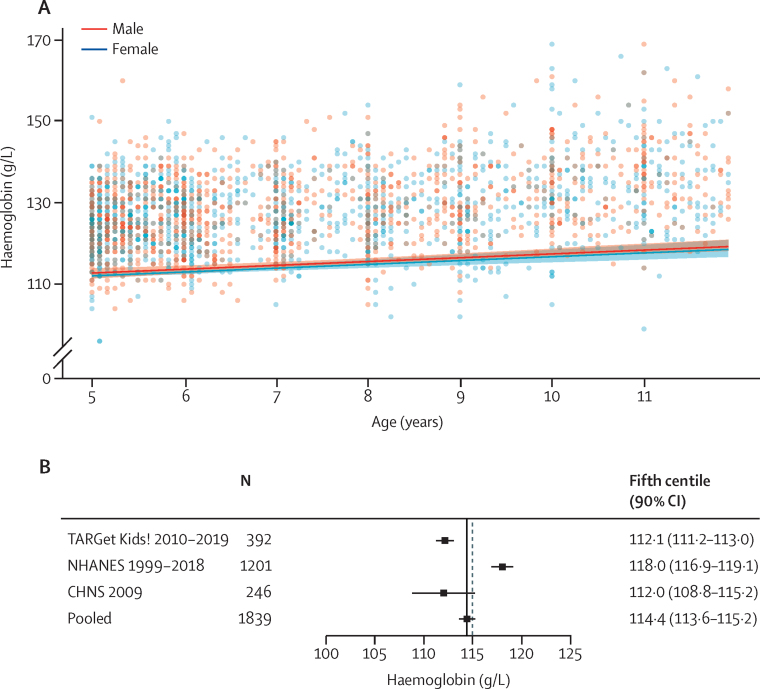
Derivation of haemoglobin thresholds in children aged 5–11 years (A) Continuous thresholds in male and female children aged 5–11 years pooled across the following datasets: TARGet Kids!, NHANES, and CHNS. The pooled continuous centiles and 90% CIs across data sources were estimated without accounting for the complex survey design and weighting. (B) Individual study and pooled (fixed effect, black line) estimates of the fifth centile of the haemoglobin distribution in the healthy reference population in children aged 5–11 years. The dashed line in panel B represents the cutoff of anaemia according to the WHO guideline.^4^ CHNS=China Health and Nutrition Survey. NHANES=National Health and Nutrition Examination Survey (USA). TARGet Kids!=Applied Research Group for Kids (Canada).

**Figure 4:**
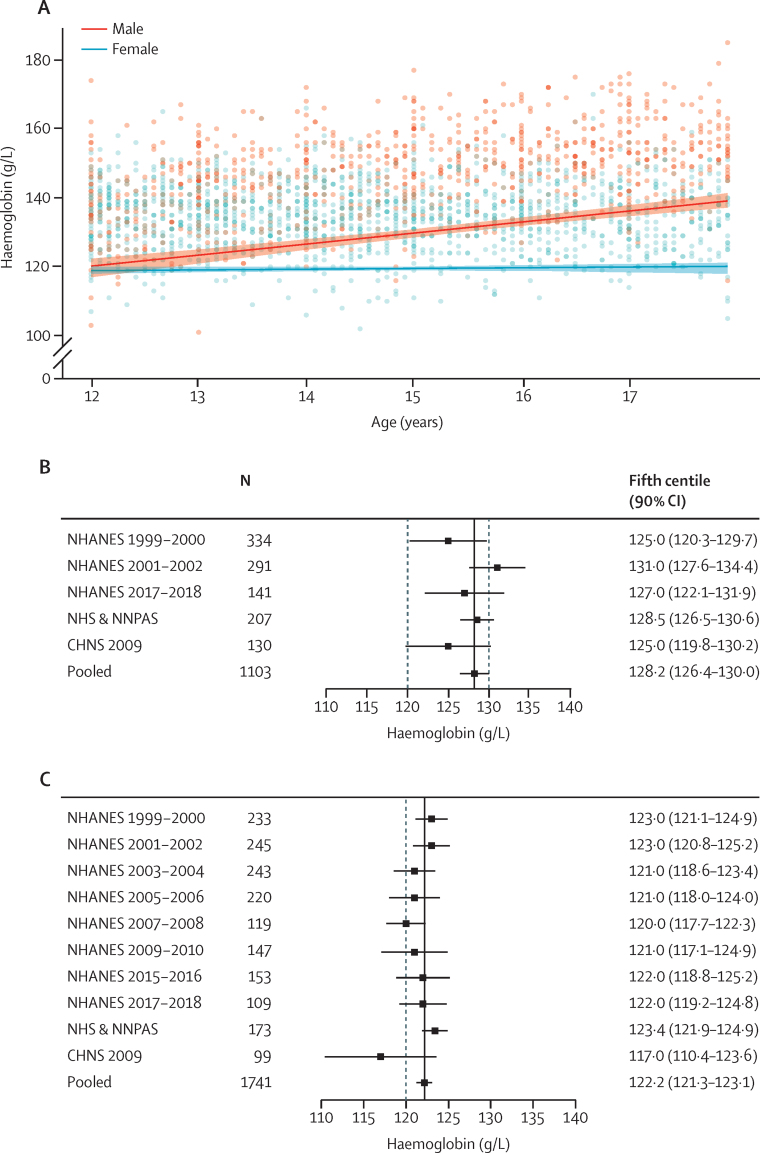
Derivation of haemoglobin thresholds in adolescents aged 12–17 years (A) Continuous thresholds in male and female adolescents aged 12–17 years pooled across the following datasets: NHANES and CHNS. The pooled continuous centiles and 90% CIs across data sources were estimated without accounting for the complex survey design and weighting. (B) Individual study and pooled (fixed effect, black line) estimates of the fifth centile of the haemoglobin distribution in the healthy reference population in male adolescents aged 12–17 years. (C) Individual study and pooled (fixed effect, black line) estimates of the fifth centile of the haemoglobin distribution in the healthy reference population in female adolescents aged 12–17 years. The dashed line in panels B and C represents the cutoff for anaemia according to the WHO guideline;^4^ there is a different cutoff for male adolescents aged 12–14 years (120 g/L) and those aged 15 years and older (130 g/L). CHNS=China Health and Nutrition Survey. NHANES=National Health and Nutrition Examination Survey (USA). NHS=National Health Survey (Australia). NNPAS=National Nutrition and Physical Activity Survey (Australia).

We identified eight data sources comprising 18 individual datasets that were eligible for inclusion in the analysis (appendix pp 10–16). Included studies are summarised in the table. Studies consisted of national health or nutrition surveys (Health Survey for England [HSE],^45^ National Health and Nutrition Examination Survey [NHANES]^46^ Australian Health Survey [AHS],^47^ China Health and Nutrition Survey [CHNS], and Encuesta Nacional de Salud y Nutrición [ENSANUT]^48^) observational studies (The Applied Research Group for Kids [TARGet Kids!],^49^ and Generation R^50^) and a clinical trial (Benefits and Risks of Iron interventions in Children [BRISC]).^51^ Information on sex or gender was self-reported, and data reported gender in NHANES, CHNS, ENSANUT, and Target Kids!, and sex in HSE, AHS, BRISC, and Generation R. All studies reported only male or female. In this report we refer to males and females using the term sex. We separately analysed thresholds for adult men aged 18–65 years, adult women aged 18–65 years, children aged 6–59 months (further sub-categorised as 6–23 months and 24–59 months), children aged 5–11 years, male and female adolescents aged 12–17 years, and pregnant women aged 18–45 years (by trimester). Insufficient data were available for children younger than 6 months, and our exclusion criteria approach precluded adequate populations of individuals aged 65 years or older from the analyses. Reference populations were derived from multi-ethnic populations in the USA (including individuals identifying as Black or Asian), England (including individuals identifying as Asian), Australia, Canada, and the Netherlands, and populations from China, Bangladesh, and Ecuador. For each survey, demographic characteristics (including age, self-reported ancestry, haemoglobin, iron status, and inflammation) for the overall and reference (healthy) sample are shown in the appendix (pp 26–76). Reasons for exclusion from the reference sample and exclusion details are provided in the appendix (pp 77–165). Among study participants with non-missing haemoglobin, ferritin, and CRP data, 16 867 (87·6%) of 19 249 adult men, 26 193 (87·8%) of 29 846 adult women, 3429 (78·8%) of 4353 children aged 6–23 months, 3995 (68·1%) of 5869 children aged 24–59 months, 2571 (58·3%) of 4413 children aged 5–11 years, 1517 (57·9%) of 2620 male children and adolescents aged 12–17 years, 3543 (67·0%) of 5288 female children and adolescents aged 12–17 years, 4279 (84·7%) of 5054 women in their first trimester of pregnancy, and 2083 (94·9%) of 2194 women in their second trimester of pregnancy were excluded from the healthy reference sample. Pooled analyses for the fifth centile for haemoglobin concentration in healthy individuals are presented in figures 1–4, while results for the 2·5th centile are available in the appendix (pp 166–68).

Continuous thresholds in males and females based on all available datasets are summarised in the appendix (p 187), indicating periods of change and divergence between sexes in thresholds across the life course from age 6 months to 65 years.

Figure 1 presents estimates of haemoglobin thresholds to define anaemia in adult men (n=2377) and women (n=3640). The data were derived from largely multi-ethnic populations (comprising individuals who self-identified as White, Black, and Asian) from the USA, England, Australia, and China (appendix pp 26–48). Reasons for exclusion of individuals from each dataset are summarised and detailed in the appendix (pp 77–120). Continuous analyses of the data indicate that in adults aged 18–65 years, the fifth centile haemoglobin concentration of the reference population is higher in males than in non-pregnant females (figure 1A; appendix p 187). Pooled analyses indicate that the fifth centile for haemoglobin concentrations in healthy adult men is 134·9 g/L (90% CI 134·2–135·6; figure 1B). In adult non-pregnant women, the fifth centile is 119·7 g/L (119·1–120·3; figure 1C). Sensitivity analyses were done where the ferritin threshold to define iron deficiency was raised from 15 μg/L to 30 μg/L, 45 μg/L, and 100 μg/L (appendix pp 169–70). Haemoglobin values in adult men are 135·7 g/L (90% CI 135·0–136·4) for the 30 μg/L ferritin threshold, 136·0 g/L (135·2–136·8) for the 45 μg/L ferritin threshold, and 137·9 g/L (136·9–138·8) for the 100 μg/L ferritin threshold; and in adult non-pregnant women the haemoglobin values are 121·1 g/L (120.5–121.8) for the 30 μg/L ferritin threshold, 122·2 g/L (121·4–123·0) for the 45 μg/L ferritin threshold, and 117·8 g/L (112·5–123·0) for the 100 μg/L ferritin threshold. In an additional analysis of adult non-pregnant women restricted to those studies that measured haemoglobin in both adult women and men, the fifth centile is 119·2 g/L (90% CI 118·6–119·8).

Figure 2 summarises estimates of haemoglobin thresholds in children aged 6–23 months (n=924) and those aged 24–59 months (n=1874). Data were derived from multi-ethnic populations in Canada,^49^ the USA, Ecuador,^48^ and Bangladesh (appendix pp 49–55). Reasons for exclusion are summarised in the appendix (pp 121–33). Continuous analysis indicates that thresholds are similar in males and females and hence sex-specific thresholds are not necessary in this age group (figure 2A; appendix p 187). Continuous analyses also indicate an increase in the threshold over the first 5 years of life but especially over the first 2 years (figure 2A; appendix p 187). Discrete thresholds were therefore ascertained in children aged 6–23 months and those aged 24–59 months. Pooled analysis indicates the fifth centile for haemoglobin concentrations in children aged 6–23 months is 104·4 g/L (90% CI 103·5–105·3; figure 2B), and in children aged 24–59 months it is 110·2 g/L (109·5–110·9; figure 2C). Sensitivity analyses were performed in which the ferritin threshold to define iron deficiency was raised, the CRP threshold to define inflammation was lowered from 5 mg/L to 1 mg/L, and in which only children with a mean cell volume higher than 73 fL (aged 6–23 months) or higher than 75 fL (aged 24–59 months) were included. Haemoglobin values for the increased ferritin thresholds were 104·3 g/L (90% CI 103·3–105·2) for a ferritin threshold of 15 μg/L, 103·8 g/L (102·5–105·1) for a ferritin threshold of 30 μg/L, and 103·2 g/L (101·1–105·4) for a ferritin threshold of 45 μg/L in children aged 6–23 months and 110·3 g/L (109·6–111·0) for a ferritin threshold of 15 μg/L, 110·3 g/L (109·2–111·4) for a ferritin threshold of 30 μg/L, and 109·1 g/L (106·8–111·4) for a ferritin threshold of 45 μg/L in children aged 24–59 months; the sample size of children with a ferritin threshold of 100 μg/L was too small. These sensitivity analyses indicate the fifth centile estimate is robust to these parameters, as well as to changes in the statistical methods (appendix pp 171–76).

Figure 3 summarises estimates of haemoglobin thresholds in children aged 5–11 years (n=1839). Data were derived from multi-ethnic populations across Canada, the USA, and China (appendix pp 56–58). Exclusions of individuals from each data set are summarised and detailed in the appendix (pp 134–42). Continuous analyses indicate that thresholds are similar in males and females and hence sex-specific thresholds are not necessary in this age group (figure 3A; appendix p 187). Pooled analyses indicated that the fifth centile for haemoglobin concentrations in this group is 114·4 g/L (90% CI 113·6–115·2; figure 3B). Sensitivity analyses indicated that these thresholds were robust to changes in assumptions and the statistical methods (appendix pp 177–79). Haemoglobin values for the changed thresholds for iron deficiency were 115·7 g/L (90% CI 114·7–116·6) for a ferritin threshold of 30 μg/L and 114·4 g/L (112·6–116·1) for a ferritin threshold of 45 μg/L; the sample size of children with a ferritin threshold of 100 μg/L was too small for an adjusted threshold.

Figure 4 summarises estimates of haemoglobin thresholds in adolescents aged 12–17 years (males: n=1103, females: n=1741). Data were derived from multi-ethnic populations across the USA, Australia, and China (appendix pp 59–73). Reasons for exclusion of individuals from each dataset are summarised and detailed in the appendix (pp 143–62). Continuous analyses show that thresholds diverge between males and females over this period (figure 4A; appendix p 187), indicating that sex-specific thresholds are necessary in this age group. Pooled analyses indicated that the fifth centile for haemoglobin concentrations in this age group is 128·2 g/L (90% CI 126·4–130·0; figure 4B) in males and 122·2 g/L (121·3–123·1; figure 4C) in females. Sensitivity analyses indicated that these thresholds were robust to changes in assumptions and the statistical methods (appendix pp 180–85). Haemoglobin values for the changed thresholds for iron deficiency were 130·0 g/L (90% CI 127·8–132·3) for a ferritin threshold of 30 μg/L and 131·0 g/L (128·3–133·7) for a ferritin threshold of 45 μg/L in males, and 124·1 g/L (122·8–125·5) for a ferritin threshold of 30 μg/L and 124·0 g/L (122·2–125·8) for a ferritin threshold of 45 μg/L in females; the sample size of children with a ferritin threshold of 100 μg/L was too small. In a post-hoc analysis of female adolescents aged 12–17 years restricted to those studies that measured haemoglobin in both males and females, the fifth centile is 122·9 g/L (90% CI 121·8–124·1).

Data from Generation R^50^ indicated that the fifth centile for haemoglobin thresholds in healthy pregnant women was 110·3 g/L (90% CI 109·5–111·0) in the first trimester (n=772), and 105·9 g/L (104·0–107·7) in the second trimester (n=111), with insufficient data for analysis in the third trimester. Participant characteristics are summarised in the appendix (pp 74–76). Exclusions from this dataset are shown in the appendix (pp 163–65). Thresholds were robust to changes in assumptions around the definition of iron deficiency (appendix p 186). Haemoglobin values for the increased thresholds for iron deficiency were 109·9 g/L (90% CI 109·0–110·7) for a ferritin threshold of 30 μg/L and 109·8 g/L (108·8–110·8) for a ferritin threshold of 45 μg/L in the first trimester.

We investigated whether common genetic variation could explain the differences in distribution of haemoglobin concentrations among different ancestry groups (appendix pp 188–94).^52^, ^53^, ^54^, ^55^, ^56^ We found no common SNPs (minor allele frequency [MAF] >0·05) showing large effects on haemoglobin, except for rs13331259 close to the *HBA1* gene and present at a MAF of 0·11 in the African population (appendix p 191). Similarly, a structural variant^54^ in this region (chr16:172001–177200_DEL, *HBA1, HBA2/2*) occurred more frequently in individuals of African ancestry (MAF 0·176, appendix p 192), suggesting there might be multiple haemoglobin-associated variants in this region that occur at higher frequencies in African populations, potentially due to positive selection.

We also evaluated rare variation across ancestries in five clinically relevant rare anaemia genes (*HBB, KLF1, PIEZO1, SLC4A1*, and *TMPRSS6*),^57^ and found predicted loss-of-function (pLoF) variants occurred at varying frequencies (appendix pp 193–94). *HBB* pLoF variants occurred more frequently in individuals of east Asian, south Asian, and Middle Eastern ancestries; however, cumulatively the frequency of pLoF variants is very low, less than 0·006. Rare pLoF variants in *TMPRSS6* have the highest prevalence in African and African American populations, with a cumulative pLoF variant frequency of around 0·03.

## Discussion

We estimated the fifth centile of haemoglobin concentration in derived reference populations of healthy children, adolescents, and adults, to estimate statistical thresholds to define anaemia across the lifecycle. Analyses of genetic associations with haemoglobin concentration across different ancestries show no evidence of the presence of any non-clinically significant high-frequency variants. Collectively, our analyses guide a single set of global definitions for anaemia.

Statistical definitions for anaemia differ between adult women and men, with the haemoglobin threshold in women robust to sensitivity analyses based on higher ferritin thresholds (eg, <45 μg/L^12^) to indicate iron deficiency. Thresholds between sexes diverge in adolescence, increasing in males. Testosterone concentrations are higher in men than in women, and appear to drive correspondingly higher haemoglobin concentrations.^58^ A direct effect of testosterone has been observed in previous studies: for example, androgen deprivation in men causes a fall in haemoglobin concentrations that recovers when inhibition is reversed.^59^ Despite the same pre-transfusion haemoglobin concentration, male patients with transfusion-dependent beta-thalassaemia have higher erythropoiesis (eg, soluble transferrin receptor and reticulocyte concentrations) than female patients.^60^ Thus, our data do not support proposals that statistical thresholds to define anaemia should be similar between adult men and women,^28^ although it is important to recognise that in some studies, similarly reduced haemoglobin concentrations in both sexes have been associated with adverse clinical outcomes.^29^ Future prospective studies with careful consideration of iron status (beyond ferritin), hormone concentrations, and menstrual blood loss are essential to understand the separation of haemoglobin thresholds by sex.

Our analyses were able to incorporate data from children across a broad age range. Our results highlight the increase in haemoglobin concentration and hence threshold for anaemia that occurs across childhood, and especially in early life. Thresholds based on arbitrary age bands are possibly crude and necessitate jarring changes in the definition of anaemia as children grow and develop. Our data suggest the existing single categorisation at age 6–59 months used by WHO is too broad and suggest at least dividing this population into two smaller subcategories (eg, age 6–23 months and 24–59 months). Definitions of anaemia in male and female children can be combined until adolescence. Recent analyses sought to evaluate the effects of reduced haemoglobin concentrations on pregnancy outcomes, and interestingly indicated an increased risk of preterm birth at haemoglobin thresholds below the statistical thresholds proposed here.^61^

Our analysis builds on previous studies using chiefly US-based data to estimate fifth centiles of haemoglobin in iron-replete, non-inflamed populations^5^ by using multinational datasets with the addition of clinical criteria for health, and covers a broad age range. Our findings were robust to variations in criteria for iron deficiency and inflammation, to statistical methods, and to secular health trends. Analyses of population surveys of biochemically iron-replete and non-inflamed individuals across several low-income and middle-income countries have reported substantial heterogeneity of the fifth centile of haemoglobin thresholds between countries in women and preschool children (ie, those aged <5 years).^36^, ^62^, ^63^ These heterogenous thresholds could relate to high burdens of undetected inflammatory disease in these studies. For example, asymptomatic sub-microscopic *Plasmodium* parasitaemia is highly prevalent in children in sub-Saharan Africa,^64^ while respiratory and diarrhoeal infections are common in infants in south Asia.^51^ The high burden of inflammation and unreported frequent clinical illnesses mean that in such settings, a prospectively recruited study with carefully curated inclusion criteria is needed to define a healthy haemoglobin threshold.

Using GWAS we found evidence for the heterogeneity of both common and rare genetic effects on haemoglobin across different ancestries, including two variants proximal to the alpha-thalassaemia genes (*HBA1/HBA2*), which show larger effect sizes than might be expected given their frequency within individuals of African ancestry. These variants are clinically important and must be detectable by a diagnosis of anaemia. We also examined rare variants in genes that are recognised to be clinically relevant causes of inherited anaemia and found these to vary in frequency across populations. Although there was some evidence there might be a higher burden of rare variants with large effects in some ancestral populations, clinically relevant rare variants cumulatively occur at low frequencies and are thus unlikely to influence the overall population-level haemoglobin distribution. The GWAS used in our analyses have high power and incorporate replication. Thus our results do not support the adoption of lower haemoglobin thresholds in populations or individuals of different ancestries, as per recent proposals.^65^ A further limitation of ancestry-specific thresholds is that clinical classification of individuals into ancestral groups is imperfect, as there is substantial genetic diversity within so-called super-populations, and such approaches also do not account for admixed individuals.

Our study has several limitations. First, we defined statistical thresholds on the basis of the fifth centile of the distribution of haemoglobin values in a healthy reference population, rather than functional thresholds that indicate the risk of symptoms or disease; future studies could endeavour to develop functional thresholds. Studies that measured other iron indices, such as transferrin saturation, reticulocyte haemoglobin, soluble transferrin receptor, and hepcidin in clinically healthy populations, might have provided a more holistic picture of iron status.^8^ Scarce data were available on heavy menstrual bleeding, although this common syndrome would be likely to influence haemoglobin through its effects on ferritin concentration, which we measured in every case. Vitamin B12 and folate measurements were not consistently measured in these datasets. Because we sought to use datasets in which clinical data were available and applied conservative exclusion criteria, this approach precluded the use of cross-sectional datasets from many low-income settings where clinical information was scarce and undetected subclinical or recurrent clinical illnesses causing inflammation could have suppressed haemoglobin concentrations, which could lead to under-estimation of thresholds. We were able to use datasets from North and South America, Europe, Australia, south and east Asia, but not sub-Saharan Africa. Most participants were excluded from the healthy reference population because of clinical or biological factors that could affect their haemoglobin concentrations. Importantly, the reference samples generally comprised multi-ethnic populations (including African ancestries). Likewise, as is a common constraint with genetic analyses, the largest datasets were representative of European ancestry populations, although we specifically included studies recruiting substantial numbers of individuals of south and east Asian and African ancestries. A further limitation of the genetic summaries we have used is that they do not capture the full extent of genetic variation, such as variants on non-autosomal chromosomes. Ideally, a prospective multicentre study could clinically exclude individuals at risk of reduced haemoglobin concentration to define the reference group.^66^, ^67^

Analysis of thresholds in children younger than 6 months, in pregnant women specifically during the third trimester, and among individuals older than 65 years was not possible due to the scarcity of datasets containing sufficient clinical data and simultaneous collection of haemoglobin and inflammatory and iron parameters. Furthermore, among older populations, datasets contained insufficient numbers of individuals meeting our stringent health criteria. Further work, potentially involving prospective studies with carefully defined clinical inclusion criteria, is needed to more precisely estimate thresholds in these important populations.

In conclusion, this study provides an evidence base to harmonise haemoglobin thresholds to define anaemia in children and adults worldwide and to inform global guidelines.

For more on **TARGet Kids!** see https://www.targetkids.ca/

For more on the **China Health and Nutrition Survey** see https://www.cpc.unc.edu/projects/china

## Data sharing

Study data used for this analysis are either publicly available or were accessed with agreement from primary data collection sources, and will not be available from the study authors.

## Conflicts of interest

SP reports a role as consultant for CSL Vifor and roles as head of the WHO Collaborating Centre for Anaemia Detection and Control, and as co-chair of the Guideline Development Group Meetings for WHO haemoglobin thresholds to define anaemia in clinical medicine and public health. All other authors declare no competing interests.
