# Supplementary material for: Haemoglobin thresholds to define anaemia from age 6 months to 65 years: estimates from international data sources
Source: Lancet Haematol. 2024 Feb 29;11(4):e253–64. doi: 10.1016/S2352-3026(24)00030-9 (PMC10983828; doi:10.1016/S2352-3026(24)00030-9)
Supplement: Supplementary appendix [file mmc1.pdf]

THE LANCET
Haematology

Supplementary appendix

This appendix formed part of the original submission and has been peer reviewed. We post it as supplied by the authors.

Supplement to: Braat S, Fielding KL, Han J, et al. Haemoglobin thresholds to define anaemia from age 6 months to 65 years: estimates from international data sources. *Lancet Haematol* 2024; published online Feb 29. https://doi.org/10.1016/S2352-3026(24)00030-9

Haemoglobin thresholds to define anaemia from age 6 months to 65 years: estimates from international data sources
APPENDIX

Table of Contents

1. Database Search Strategy 5

2. Methods 6

2.1 Approach to exclusion criteria to define healthy reference population 6

2.2 Demographics Criteria 6

2.3 Environmental and Nutritional Criteria 6

2.4 Recent Health Criteria 7

2.5 General Health Criteria 7

2.6 Laboratory Criteria 8

2.7 Pregnancy – Additional Criteria 9

2.8 Children – Additional Criteria 9

2.9 Summary of Data Used to Estimate Centiles of Haemoglobin Concentration 10

2.9.1 AHS 10
2.9.2 BRISC 11
2.9.3 CHNS 11
2.9.4 ENSANUT 12
2.9.5 Generation R 13
2.9.6 HSE 13
2.9.7 NHANES 15
2.9.8 TARGet Kids! 15

2.10 Statistical Analyses 16

2.10.1 General Principles 17
2.10.2 Sample Size 18
2.10.3 Survey Weighting and Design 18
2.10.4 Discrete Thresholds 19
2.10.5 Continuous Thresholds 21

2.11 Genetic Analyses 22

2.12 Software 23

2.13 Ethics Approvals 25

3. Demographics 26

3.1 Demographics Adults (18-65 years) males 26

Table 3.1.1 US NHANES 1999-2000: Participant characteristics (Males) (18-65 years). 26
Table 3.1.2 US NHANES 2001-2002: Participant characteristics (Males) (18-65 years). 27
Table 3.1.3 US NHANES 2017-2018: Participant characteristics (Males) (18-65 years). 28
Table 3.1.4 England HSE 1998: Participant characteristics (Males) (18-65 years). 29

Table 3.1.5 England HSE 2006: Participant characteristics (Males) (18-65 years).. 30
Table 3.1.6 England HSE 2009: Participant characteristics (Males) (18-65 years).. 31
Table 3.1.7 CHN CHNS 2009: Participant characteristics (Males) (18-65 years). 32
Table 3.1.8 AUS NHS 2011-2012: Participant characteristics (Males) (18-65 years). 33
Table 3.1.9 AUS NNPAS 2011-2012: Participant characteristics (Males) (18-65 years). 34

3.2 Demographics Adults (18-65 years) females..35
Table 3.2.1 US NHANES 1999-2000: Participant characteristics (Females) (18-65 years). 35
Table 3.2.2 US NHANES 2001-2002: Participant characteristics (Females) (18-65 years). 36
Table 3.2.3 US NHANES 2003-2004: Participant characteristics (Females) (18-65 years). 37
Table 3.2.4 US NHANES 2005-2006: Participant characteristics (Females) (18-65 years). 38
Table 3.2.5 US NHANES 2007-2008: Participant characteristics (Females) (18-65 years). 39
Table 3.2.6 US NHANES 2009-2010: Participant characteristics (Females) (18-65 years). 40
Table 3.2.7 US NHANES 2015-2016: Participant characteristics (Females) (18-65 years). 41
Table 3.2.8 US NHANES 2017-2018: Participant characteristics (Females) (18-65 years). 42
Table 3.2.9 England HSE 1998: Participant characteristics (Females) (18-65 years). 43
Table 3.2.10 England HSE 2006: Participant characteristics (Females) (18-65 years). 44
Table 3.2.11 England HSE 2009: Participant characteristics (Females) (18-65 years). 45
Table 3.2.12 CHN CHNS 2009: Participant characteristics (Females) (18-65 years). 46
Table 3.2.13 AUS NHS 2011-2012: Participant characteristics (Females) (18-65 years). 47
Table 3.2.14 AUS NNPAS 2011-2012: Participant characteristics (Females) (18-65 years). 48

3.3 Demographics Children (6-23 months)..49
Table 3.3.1 CAN TARGet Kids! 2010-2019: Participant characteristics (6-23 months). 49
Table 3.3.2 BAN BRISC 2017-2020: Participant characteristics (11 months). 50
Table 3.3.3 ECU ENSANUT 2011-2013: Participant characteristics (6-23 months). 51
Table 3.3.4 US NHANES 2003-2018: Participant characteristics (12-23 months). 52

3.4 Demographics Children (24-59 months)..53
Table 3.4.1 CAN TARGet Kids! 2010-2019: Participant characteristics (24-59 months). 53
Table 3.4.2 ECU ENSANUT 2011-2013: Participant characteristics (24-59 months). 54
Table 3.4.3 US NHANES 1999-2018: Participant characteristics (24-59 months). 55

3.5 Demographics Children (5-11 years)...56
Table 3.5.1 CAN TARGet Kids! 2010-2019: Participant characteristics (5-11 years). 56
Table 3.5.2 US NHANES 1999-2018: Participant characteristics (5-11 years). 57
Table 3.5.3 CHN CHNS 2009: Participant characteristics (5-11 years). 58

3.6 Demographics Adolescents (12-17 years) males 59
Table 3.6.1 US NHANES 1999-2000: Participant characteristics (Males) (12-17 years). 59
Table 3.6.2 US NHANES 2001-2002: Participant characteristics (Males) (12-17 years). 60
Table 3.6.3 US NHANES 2017-2018: Participant characteristics (Males) (12-17 years). 61
Table 3.6.4 AUS NHS and NNPAS 2011-2012: Participant characteristics (Males) (12-17 years). 62
Table 3.6.5 CHN CHNS 2009: Participant characteristics (Males) (12-17 years). 63

3.7 Demographics Adolescents (12-17 years) females..64
Table 3.7.1 US NHANES 1999-2000: Participant characteristics (Females) (12-17 years). 64
Table 3.7.2 US NHANES 2001-2002: Participant characteristics (Females) (12-17 years). 65
Table 3.7.3 US NHANES 2003-2004: Participant characteristics (Females) (12-17 years). 66
Table 3.7.4 US NHANES 2005-2006: Participant characteristics (Females) (12-17 years). 67
Table 3.7.5 US NHANES 2007-2008: Participant characteristics (Females) (12-17 years). 68
Table 3.7.5 US NHANES 2009-2010: Participant characteristics (Females) (12-17 years). 69
Table 3.7.6 US NHANES 2015-2016: Participant characteristics (Females) (12-17 years). 70
Table 3.7.7 US NHANES 2017-2018: Participant characteristics (Females) (12-17 years). 71
Table 3.7.8 AUS NHS and NNPAS 2011-2012: Participant characteristics (Females) (12-17 years). 72

Table 3.7.9 CHN CHNS 2009: Participant characteristics (Females) (12-17 years). 73

3.8 Demographics Pregnant women 74
Table 3.8.1 NL Generation R: Participant characteristics (Trimester 1) (18-45 years). 74
Table 3.8.2 NL Generation R: Participant characteristics (Trimester 2) (18-45 years). 75
Table 3.8.3 NL Generation R: Participant characteristics (Trimester 3) (18-45 years). 76

4. Detailed Individual Exclusions 77

4.1 Detailed Individual Exclusions Adults (18-65 years) 77
Figure 4.1.1 Summary of exclusions of special interest from overall sample in males (18-65 years). 77
Figure 4.1.2 Summary of exclusions of special interest from overall sample in non-pregnant females (18-65 years). 78
Table 4.1.1 US NHANES 1999-2000: Participant characteristics of violations (18-65 years). 79
Table 4.1.2 US NHANES 2001-2002: Participant characteristics of violations (18-65 years). 82
Table 4.1.3 US NHANES 2003-2004: Participant characteristics of violations (Females) (18-65 years). 85
Table 4.1.4 US NHANES 2005-2006: Participant characteristics of violations (Females) (18-65 years). 88
Table 4.1.5 US NHANES 2007-2008: Participant characteristics of violations (Females) (18-65 years). 91
Table 4.1.6 US NHANES 2009-2010: Participant characteristics of violations (Females) (18-65 years). 94
Table 4.1.7 US NHANES 2015-2016: Participant characteristics of violations (Females) (18-65 years). 97
Table 4.1.8 US NHANES 2017-2018: Participant characteristics of violations (18-65 years). 100
Table 4.1.9 England HSE 1998: Participant characteristics of violations (18-65 years). 103
Table 4.1.10 England HSE 2006: Participant characteristics of violations (18-65 years). 107
Table 4.1.11 England HSE 2009: Participant characteristics of violations (18-65 years). 111
Table 4.1.12 CHN CHNS 2009: Participant characteristics of violations (18-65 years). 114
Table 4.1.13 AUS NHS 2011-2012: Participant characteristics of violations (18-65 years). 118
Table 4.1.14 AUS NNPAS 2011-2012: Participant characteristics of violations (18-65 years). 120

4.2 Detailed Individual Exclusions Children (6-23 months) 121
Figure 4.2 Summary of exclusions of special interest from overall sample in children (6-23 months). 121
Table 4.2.1 CAN TARGet Kids: Participant characteristics of violations (6-23 months). 122
Table 4.2.2 BAN BRISC 2017-2020: Participant characteristics of violations (6-23 months). 125
Table 4.2.3 ECU ENSANUT 2011-2013: Participant characteristics of violations (6-23 months). 126
Table 4.2.4 US NHANES 2003-2018: Participant characteristics of violations (12-23 months). 127

4.3 Detailed Individual Exclusions Children (24-59 months) 128
Figure 4.3 Summary of exclusions of special interest from overall sample in children (24-59 months). 128
Table 4.3.1 CAN TARGet Kids: Participant characteristics of violations (24-59 months). 129
Table 4.3.2 ECU ENSANUT 2011-2013: Participant characteristics of violations (24-59 months). 132
Table 4.3.3 US NHANES 1999-2018: Participant characteristics of violations (24-59 months). 133

4.4 Detailed Individual Exclusions Children (5-11 years) 134
Figure 4.4 Summary of exclusions of special interest from overall sample in children (5-11 years). 134
Table 4.4.1 CAN TARGet Kids: Participant characteristics of violations (5-11 years). 135
Table 4.4.2 US NHANES 1999-2018: Participant characteristics of violations (5-11 years). 138
Table 4.4.3 CHN CHNS 2009: Participant characteristics of violations (5-11 years). 140

4.5 Detailed Individual Exclusions Adolescents (12-17 years) 143
Figure 4.5.1 Summary of exclusions of special interest from overall sample in males (12-17 years). 143
Figure 4.5.2 Summary of exclusions of special interest from overall sample in non-pregnant females (12-17 years). 144
Table 4.5.1 US NHANES 1999-2000: Participant characteristics of violations (12-17 years). 145
Table 4.5.2 US NHANES 2001-2002: Participant characteristics of violations (12-17 years). 147
Table 4.5.3 US NHANES 2003-2004: Participant characteristics of violations (Females) (12-17 years). 149
Table 4.5.4 US NHANES 2005-2006: Participant characteristics of violations (Females) (12-17 years). 151

Table 4.5.5 US NHANES 2007-2008: Participant characteristics of violations (Females) (12-17 years). 153
Table 4.5.6 US NHANES 2009-2010: Participant characteristics of violations (Females) (12-17 years). 154
Table 4.5.7 US NHANES 2015-2016: Participant characteristics of violations (Females) (12-17 years). 155
Table 4.5.8 US NHANES 2017-2018: Participant characteristics of violations (12-17 years). 156
Table 4.5.9 AUS NHS 2011-2012: Participant characteristics of violations (12-17 years)........................... 158
Table 4.5.10 AUS NNPAS 2011-2012: Participant characteristics of violations (12-17 years)..................... 159
Table 4.5.11 CHN CHNS 2009: Participant characteristics of violations (12-17 years)............................... 160

4.6 Detailed Individual Exclusions Pregnant women 163
Table 4.6.1 NL Generation R: Participant characteristics of violations (Trimester 1) (18-45 years). 163
Table 4.6.2 NL Generation R: Participant characteristics of violations (Trimester 2) (18-45 years). 164
Table 4.6.3 NL Generation R: Participant characteristics of violations (Trimester 3) (18-45 years). 165

5. Discrete Thresholds.. 166
Figure 5.1: Adults (18-65 years): 2·5th centile (90% CI). 166
Figure 5.2: Children (6-23 months, 24-59 months, 5-11 years): 2·5th centile (90% CI). 167
Figure 5.3: Adolescents (12-17 years): 2·5th centile (90% CI).. 168
Figure 5.4: Sensitivity Analyses Adults (Males) (18-65 years): 5th centile (90% CI). 169
Figure 5.5: Sensitivity Analyses Adults (Females) (18-65 years): 5th centile (90% CI)................................ 170
Figure 5.6: Sensitivity Analyses Children (6-23 months): 5th centile (90% CI) (Part 1).............................. 171
Figure 5.7: Sensitivity Analyses Children (6-23 months): 5th centile (90% CI) (Part 2).............................. 172
Figure 5.8: Sensitivity Analyses Children (6-23 months): 5th centile (90% CI) (Part 3).............................. 173
Figure 5.9: Sensitivity Analyses Children (24-59 months): 5th centile (90% CI) (Part 1)............................ 174
Figure 5.10: Sensitivity Analyses Children (24-59 months): 5th centile (90% CI) (Part 2).......................... 175
Figure 5.11: Sensitivity Analyses Children (24-59 months): 5th centile (90% CI) (Part 3).......................... 176
Figure 5.12: Sensitivity Analyses Children (5-11 years): 5th centile (90% CI) (Part 1). 177
Figure 5.13: Sensitivity Analyses Children (5-11 years): 5th centile (90% CI) (Part 2). 178
Figure 5.14: Sensitivity Analyses Children (5-11 years): 5th centile (90% CI) (Part 3). 179
Figure 5.15: Sensitivity Analyses Adolescents (Males) (12-17 years): 5th centile (90% CI) (Part 1). 180
Figure 5.16: Sensitivity Analyses Adolescents (Males) (12-17 years): 5th centile (90% CI) (Part 2). 181
Figure 5.17: Sensitivity Analyses Adolescents (Males) (12-17 years): 5th centile (90% CI) (Part 3). 182
Figure 5.18: Sensitivity Analyses Adolescents (Females) (12-17 years): 5th centile (90% CI) (Part 1)......... 183
Figure 5.19: Sensitivity Analyses Adolescents (Females) (12-17 years): 5th centile (90% CI) (Part 2)......... 184
Figure 5.20: Sensitivity Analyses Adolescents (Females) (12-17 years): 5th centile (90% CI) (Part 3)......... 185
Figure 5.21: Sensitivity Analyses Pregnant Women (18-45 years): 5th centile (90% CI). 186

6. Continuous Thresholds 187
Figure 6.1. Continuous 5th centile thresholds across the life course (6 months to 65 years). 187

7. Ancestry/Genetics.. 188

7.1 Ancestry-Specific Genetic Associations with Variation in Haemoglobin Concentration 188
Table 7.1: Genetic association results (summary statistics) of haemoglobin concentrations used................. 189
Figure 7.1: Multi-ancestry and ancestry-specific haemoglobin-associated genome-wide significant loci...... 190
Figure 7.2: Scatter plot of minor allele frequency (MAF) and standardized effect size for SNPs. 191
Figure 7.3. Scatter plot of minor allele frequency (MAF) and standardized effect size for SVs.................... 192
Figure 7.4: Scatter plot of frequency and effect size in the UKBB cohort.. 193
Figure 7.5: Allele frequency plot of predicted loss-of-function (pLOF) variants.. 194

7.2 Genes & Health Research Team 195

8. References 196

1. Database Search Strategy

Our database search strategy took place between May 2021 and September 2022. We focused our initial search on National health or nutrition databases from countries that are part of The Organization for Economic Co-operation and Development (OECD). Our search terms included the respective country name AND “national health database”. Databases were required to have venous haemoglobin, ferritin, and inflammation biomarker C-Reactive Protein (CRP), as well as clinical information to ensure that an apparently healthy reference sample could be selected. National Health Surveys of England (Health Survey for England [HSE]), United States of America (National Health and Nutrition Examination Survey [NHANES]), and Australia (Australian Health Survey [AHS]) met these criteria and were included in our study. A National Health Survey from New Zealand was investigated but excluded as it did not contain ferritin, while data from the German Health Interview and Examination Survey for Adults (DEGS) could not be included due to limitations in quality of the haemoglobin point-of-care measurement. Additionally, we were unable to make contact to obtain data from National Health Surveys in Portugal and were unable to access data from the Canadian Health Measures Survey, due to regulatory requirements for data access. We were unable to identify databases that met our requirements from National surveys in Austria, Chile, Costa Rica, the Czech Republic, Denmark, Estonia, Finland, France, Greece, Hungary, Iceland, Ireland, Israel, Italy, Japan, Latvia, Lithuania, Luxembourg, Mexico, Norway, Poland Slovak Republic, and Sweden.

We also searched for prospective cohort or clinical trial studies from countries with a low background inflammation prevalence, that measured venous haemoglobin, ferritin, and CRP, and collected detailed health outcomes which would enable identification of a healthy reference sample, particularly in children and in pregnancy. This identified studies from Canada (The Applied Research Group for Kids [TARGet Kids!]) and The Netherlands (Generation R), which were included in our analyses. We obtained data from an Australian cohort study (HAPPI Kids) but excluded it upon learning that either ferritin or haemoglobin were measured in individuals, but not both. We expanded our search to non-OECD countries and identified National Health Surveys from China (China Health and Nutrition Survey [CHNS]) and Ecuador (Encuesta Nacional de Salud y Nutrición [ENSANUT]), which met our criteria. Finally, we identified and included data from a clinical trial of iron supplemented infants in Bangladesh (Benefits and Risks of Iron interventions in Children [BRISC]) in our analyses.

2. Methods

2.1 Approach to exclusion criteria to define healthy reference population

For each data source, the healthy reference sample comprised individuals who were not excluded from the study sample based on pre-defined criteria for an apparently healthy individual, defined separately for children and adolescents (6 months to <18 years), adults aged 18-65 years (non-pregnant) and pregnant women aged 18-45 years.

We applied the criteria to population studies, some of which have been repeated over multiple waves, in order to define a post-hoc healthy reference population. Surveys were ineligible if basic health data along with laboratory measurements (haemoglobin, ferritin, CRP at a minimum) were not available. Surveys were conducted in different countries (e.g., England, Australia, United States [US], Ecuador, China), and in some cases, the same survey platform (e.g. NHANES) was applied over multiple years. Each survey platform, as well as different years of the same platform, collected different information on health outcomes and health status, and some had a specific emphasis (e.g., on "cardiovascular health"). If more detailed criteria were available within an individual survey, these were generally applied (especially regarding health and medication use) even when this caused inconsistencies in criteria between surveys.

The general principles underpinning these inclusion and exclusion criteria are outlined below:

2.2 Demographics Criteria

Where available and applicable, data was collected from males and females. Across all surveys, ages 6 months to 65 years were analyzed, however the age range in each survey varied, and in many cases varied between years within the same survey platform. A separate analysis of pregnant women was performed, and pregnant women were excluded from the non-pregnant analysis where applicable (i.e. females in the age-groups of 12-17 years and 18-65 years).

Race/ethnicity/ancestry information was collected for each survey, although inconsistent terminology and reported categories were reported between survey platforms. In some surveys (children and adult groups) participants were able to select more than one ancestry group – in these cases, participants were considered as belonging to both ancestry groups. This data was used to describe the demographic of the groups included, but not used as exclusion criteria. Data regarding other relevant household demographic information (including remoteness, household income/deciles, or poverty ratio) were also collected where available. None of these criteria were used as exclusion criteria for any of the analyses.

2.3 Environmental and Nutritional Criteria

The surveys selected for this analysis were performed in countries with low rates of endemic infection. We excluded individuals living above 750m where individual altitude of residence was provided (Ecuador) or in provinces which

were known to be elevated (China). Only one study (TARGet Kids!) evaluated exposure to lead (these children were excluded); no surveys asked about other toxins.

Body Mass Index (BMI) was used to exclude those who are underweight or at risk of malnutrition (BMI<18·5kg/m^2) and those categorized as obese (BMI>30kg/m^2).

Participants who identified themselves as a current smoker were excluded. This exclusion criteria applied to any method of smoking including cigarettes, pipes, and cigars (questioned specifically in most surveys).

Participants were excluded if they reported excessive alcohol use defined using US Centers for Disease Control and Prevention (CDC) recommendations. The US CDC guidelines were chosen to use across all surveys as they aligned with much of Europe and were the highest recommended limits in any survey country. The US CDC criteria were applied to all surveys as follows (noting that a standard alcoholic drink contains 14g alcohol as defined in the US):

- No more than 1 drink per day for women, or 2 drinks per day for men, on a regular basis AND
- No more than 4 drinks on any one day for women, and no more than 8 drinks on any one day for men

Questions on illicit drug use were not asked, or responses were unavailable, for most surveys.

2.4 Recent Health Criteria

All adult surveys comprised population community surveys. Childrens' surveys included children selected as healthy by their routine care provider.

Participants who reported any recent illness in the time period preceding the survey were excluded. All general surveys included questions regarding recent acute illness, although the preceding time period varied between surveys, with some queried illness within 2 weeks, and some within 1 month. Some surveys asked about 'any' illness, where others specified coughs, colds, pneumonia, gastrointestinal illness.

Any participant who had been admitted to hospital in the last 12 months, or those who reported traumatic injury, major bleeding, pregnancy or childbirth within the preceding 12 months, was excluded where this information was available. Where data was available, participants who had recently donated blood were also excluded.

2.5 General Health Criteria

All surveys included questions regarding participants medical history and comorbidities, and these questions were used to exclude participants with significant health conditions. Where possible, participants weren't excluded for resolved health conditions (e.g., historical thyroid disease) or minor issues not expected to contribute to anaemia (e.g. astigmatism). However, where specific details were not available, participants who identified themselves as having any health condition were excluded. Outside of questions about general health or "diseases of blood", no survey asked specifically about history of haemoglobinopathy.

In general, all participants requiring any regular prescription medication were excluded. In two surveys, it was possible to exclude all except people on hormonal contraception. Where possible, participants taking supplements or vitamins were included, apart from those specifically on supplementation of hematinic (iron, B12, folate).

In children, we excluded all reporting any use of medications (including over-the-counter medications).

2.6 Laboratory Criteria

For this analysis, surveys were only included where haemoglobin, ferritin, and CRP results were available. In some surveys, specific years targeted specific populations (children, women, pregnant or non-pregnant adults) for some testing. If an individual had missing data for any of these parameters, they were excluded from this analysis.

Ferritin limits were set according to the "WHO guidelines on use of ferritin concentrations to assess iron status in individuals and populations", (1) Table 1, for "apparently healthy individuals":

- Age less than 5 years old: ferritin <12ug/L defines iron deficiency
- Age greater than 5 years old, including pregnant women in first trimester: ferritin <15ug/L defines iron deficiency; and ferritin >150ug/L in females, or >200ug/L in males, defines risk of iron overload

A CRP cut off of <5mg/L was used to exclude those with current inflammation.

No criteria were applied to haemoglobin, or any other full blood examination parameters. Mean Corpuscular Volume (MCV) was collected where available, but was not available for most surveys, and no criteria were based on this measurement except for sensitivity analyses in children as described.

Where available, abnormal renal function was an exclusion criterion (eGFR<90ml/min/1·73m^2).

Estimated Glomerular Filtration Rate (eGFR) was used to determine if non-pregnant adult participants and pregnant women had kidney disease in the AHS, HSE (relevant data available in year 2009 only), NHANES and CHNS. An eGFR less than 90 ml/min/1.73 m^2 was considered an indicator of abnormal kidney function in adults and pregnant women.

The eGFR (ml/min/1.73 m^2) was calculated by Chronic Kidney Disease Epidemiology (CKD-EPI) (2) equation using serum creatinine (mg/dL), age (years), and sex (male/female) without race adjustment. (2, 3) This formula was used for adults and pregnant women, noting that in pregnancy there is no accepted eGFR calculation or range.

$$eGFR = 142 \times min\left(\frac{creatinine}{K}, 1\right)^{\alpha} \times max\left(\frac{creatinine}{K}, 1\right)^{-1.2} \times 0.9938^{Age} \times SexFactor$$

Where creatinine = serum creatinine (mg/dL), K=0·7 for females or 0·9 for males, α = -0·241 for females or -0·302 for males, min = minimum, max = maximum, age = age (years), and SexFactor = 1·012 for females and 1 for males. The formula was implemented, and calculations were validated by randomly checking eGFR derivations with an online calculator https://www.kidney.org/professionals/kdoqi/gfr_calculator (accessed March 2022) based on the CKD-EPI equation. For some NHANES survey years, serum creatinine as provided required a conversion in units

(from µmol/L to mg/dL by multiplying by 1/88·4) and/or calibration due to use of non-standardized laboratory methods before deriving eGFR.

For children, we did not implement an exclusion criterion for eGFR because the rates of undiagnosed chronic kidney disease in children with no other comorbidities are likely be very low, and creatinine was not consistently available across surveys.

Where available, abnormal liver function tests were also used to exclude participants.

2.7 Pregnancy – Additional Criteria

Only two surveys included adequate numbers of pregnant participants for analysis – NHANES, and Generation R. Data from pregnant participants were excluded based on all available general health parameters as above where available; with an additional set of criteria to exclude those with pregnancy related illness or complications.

In NHANES, where pregnant participants were a subset of the general survey, the same criteria for health were applied as for other adults. An altered BMI range was used, and all available questions regarding pregnancy status (month of pregnancy) and pregnancy complications (gestational diabetes, and hypertension during pregnancy) were used as exclusion criteria.

Generation R was a targeted study of pregnant women. Fewer questions regarding general health were available, with more data available about pregnancy associated health problems.

Where possible, only those with singleton pregnancies were included. Those with gestational diabetes, pregnancy induced hypertension, pre-eclampsia, or HELLP syndrome were excluded. Pregnancy related cardiomyopathy or other serious complications were excluded.

2.8 Children – Additional Criteria

In children, many of the same parameters were sought to identify the reference population as in adults. Additionally, in those under 2 years old, any child noted to have had birth complications was excluded (such as premature birth or low birth weight, NICU admission).

Any child with active health problems was excluded. This included children with potentially minor conditions (e.g., eczema or allergies), however sufficient information on severity was not generally available and thus a restrictive approach as adopted to ensure exclusion of any condition that may affect general health, nutrition, and growth, or that may cause inflammation.

2.9 Summary of Data Used to Estimate Centiles of Haemoglobin Concentration

2.9.1 AHS

The Australian Health Survey (AHS) was a large and comprehensive national health survey conducted in a sample of private dwellings in urban and rural areas in Australia. (4, 12) All people selected in the Australian Health Survey were selected either in the National Health Survey (NHS) (13) or National Nutrition and Physical Activity Survey (NNPAS). (14) The National Health Measures Survey, which included a blood sample for those aged 12 years and over, was collected in 10,192 persons who participated in either the NHS or NNPAS.

**Topic**	**National Health Survey**
Location	Australia
Study type	Cross-sectional
Primary purpose	To collect information about the health status of the population, including long-term health conditions experienced; health-related aspects of people's lifestyles; use of health services such as consultations with health practitioners and actions people have recently taken for their health; and demographic and socioeconomic characteristics. In addition, the national health measures survey component aimed at collecting information about chronic disease biomarkers, including tests for diabetes, cholesterol, triglycerides, kidney disease and liver function, nutrient biomarkers, including tests for iron, folate, iodine and vitamin D levels.
Date range	6 March 2011 – 17 March 2012, the national health measures survey component occurred between March 2011 and September 2012
Age range	The blood sample in the national health measures survey component was conducted in those 12 years of age and older
In/exclusion and/or Sampling strategy	Sampling strategy: Private households selected at random using a multistage area sample of private dwellings and surveyed over a 12-month period. Within each household, one adult and one child were randomly selected for inclusion and were interviewed by trained Australian Bureau of Statistics interviewers.<br><br>Exclusions: Very Remote areas of Australia and discrete Aboriginal and Torres Strait Islander communities; Non-private dwellings such as motels/hospitals; Certain diplomatic personnel of overseas governments; Persons whose usual place of residence was outside Australia; Members of non-Australian Defence forces (and their dependents) stationed in Australia; Visitors to private dwellings.
Sample size	20,426 persons from 15,565 dwellings, with a subset participating in the national health measures survey component
Sample size eligible for analysis*	18-65 years (men): 1,912<br>18-65 years (women): 2,480<br>12-17 years (male): 209<br>12-17 years (female): 243
Intervention	None

* Defined as having non-missing haemoglobin, ferritin, and C-reactive protein.

**Topic**	**National Nutrition and Physical Activity Survey**
Location	Australia
Study type	Cross-sectional
Primary purpose	To collect a range of information health related issues, including health status, risk factors, health service usage and medications, detailed physical activity information using self-reported and pedometer collection methods, along with detailed information on dietary intake and foods consumed. In addition, the national health measures survey component aimed at collecting information about chronic disease biomarkers, including tests for diabetes, cholesterol, triglycerides, kidney disease and liver function, nutrient biomarkers, including tests for iron, folate, iodine and vitamin D levels.
Date range	29 May 2011 – 9 June 2012, the national health measures survey component occurred between March 2011 and September 2012
Age range	The blood sample in the national health measures survey component was conducted in those 12 years of age and older.

Topic	National Nutrition and Physical Activity Survey
In/exclusion and/or Sampling strategy	Sampling strategy – Private households selected at random using a multistage area sample of private dwellings and surveyed over a 12-month period. Within each household, one adult and one child were randomly selected for inclusion and were interviewed by trained Australian Bureau of Statistics interviewers. The random sample selection code in the survey instrument was designed to give adults aged 65 years and over a double chance of being selected.<br><br>Exclusions: Very Remote areas of Australia and discrete Aboriginal and Torres Strait Islander communities; Non-private dwellings such as motels/hospitals; Certain diplomatic personnel of overseas governments; Persons whose usual place of residence was outside Australia; Members of non-Australian Defence forces (and their dependents) stationed in Australia; Visitors to private dwellings.
Sample size	12,153 persons from 9,519 dwellings, with a subset participating in the national health measures survey component
Sample size eligible for analysis*	18-65 years (men): 1,277<br>18-65 years (women): 1,593<br>12-17 years (male): 149<br>12-17 years (female): 133
Intervention	None

* Defined as having non-missing haemoglobin, ferritin, and C-reactive protein.

2.9.2 BRISC

The Benefits and risks of Iron interventions in children (BRISC) trial aimed to evaluate the impact of iron or MNPs supplementation on child cognitive function, behaviour and development in young children in Bangladesh. (5) This three-arm randomized controlled was conducted in rural Bangladesh, where 3300 8-month-old children were randomly assigned to receive either iron drops, home-fortification of food with MNPs, or placebo as part of a daily regimen for three months.

Topic	Benefits and risks of Iron interventions in children
Location	Bangladesh
Study type	Randomised controlled trial
Primary purpose	To determine the benefits and risks of iron interventions in 8-month-old children
Date range	6 July 2017 – 20 February 2019
Age range	11 months at end of 3-months intervention period
In/exclusion and/or Sampling strategy	Sampling strategy: House-to-house enumeration of the entire study area to identify potentially eligible children. Children 7.5 to 8.5 months of age were screened.<br><br>Exclusions: Children with marked anemia (a hemoglobin level of <8.0g per deciliter), current febrile illness, severe acute malnutrition, a known inherited red-cell disorder or previous transfusion, or known developmental delay were excluded. Furthermore, iron levels in drinking water were tested at screening, and children were excluded if the level exceeded 1 mg per liter.
Sample size	2,200 11-months old children who received iron intervention
Sample size eligible for analysis*	6-23 months:1,365
Intervention	Iron interventions (iron syrup and micronutrient sprinkles)

* Defined as having non-missing haemoglobin, ferritin, and C-reactive protein.

2.9.3 CHNS

The China Health and Nutrition Survey (CHNS) is a longitudinal, household-based survey covering nine provinces in China. (6) It examines the impact of national and local health, nutrition, and family planning policies on the well-being of the Chinese population. The study also assesses changes in community organizations, programs, and individual factors to understand the effects of societal transformation on health and nutrition. Out of all the years

during which the CHNS survey has been conducted since 1989, only the data collected in 2009 includes biomarker information. (15)

**Topic**	**China Health and Nutrition Survey**
Location	China
Study type	Observational longitudinal cohort study.
Primary purpose	To assess the impacts of health, nutrition, and family planning policies and programs enacted by both national and local governments. Additionally, the survey aims to explore the influence of the ongoing social and economic transformation within Chinese society on the health and nutritional well-being of its population.
Date range	2009
Age range	Individuals aged 7 or older
In/exclusion and/or Sampling strategy	Sampling strategy: A multistage, random cluster process was used to determine the surveyed sample within each province. Within the nine provinces, counties were categorized by income (low, middle, and high), and a weighted sampling scheme facilitated the random selection of four counties in each province. The selection process also involved choosing the provincial capital and, when possible, a lower-income city, with the exception that two provinces required the selection of other large cities instead of provincial capitals. Random selection was further applied to villages, townships within counties, and urban/suburban neighbourhoods within cities. The primary sampling units have now expanded to 360, encompassing 60 urban neighbourhoods, 60 suburban neighbourhoods, 30 towns, and 180 villages.
Sample size	12178 individuals participated in the 2009 survey, with 9244 providing a blood sample
Sample size eligible for analysis*	18-65 years (men): 3,352<br>18-65 years (women): 3,820<br>5-11 years: 404<br>12-17 years (male): 223<br>12-17 years (female): 187
Intervention	None

* Defined as having non-missing haemoglobin, ferritin, and C-reactive protein.

2.9.4 ENSANUT

The Encuesta Nacional de Salud y Nutrición (ENSANUT) survey is a national cross-sectional population health survey, conducted in Ecuador between 2011 and 2013. (7) The objectives were to assess the health and nutritional status of the population from 0 to 59 years. The ENSANUT survey encompassed approximately 20,000 homes nationwide. It employed ten questionnaires tailored to family size, with around 6,000 homes revisited the next day for additional surveys, blood samples.

**Topic**	**Encuesta Nacional de Salud y Nutrición**
Location	Ecuador
Study type	Cross-sectional
Primary purpose	To provide an overview of maternal and child reproductive health, chronic non-communicable diseases, nutritional status, food consumption patterns, micronutrient levels, participation in food supplementation programs, prophylactic supplementation, physical activity, access to healthcare services, and health expenditure among the Ecuadorian population aged 0–59 years.
Date range	July 2012 – February 2013
Age range	0 months -59 years, with blood samples collected in individuals 6 months – 59 years
In/exclusion and/or Sampling strategy	Sampling strategy: The sample design is probabilistic, employing a stratified, three-stage, and conglomerate approach. Rural and urban areas are distinguished as strata, based on local population characteristics. Each province is further divided into rural and urban strata, and two exclusively urban strata are designated for Quito and Guayaquil, resulting in a total of 50 domains. In the initial stage, 64 census tracts were selected within each province, encompassing both rural and urban areas, with proportional probability to size determined by the number of occupied homes. Within each selected sector, 19 homes were preselected, leading to a final selection of 12 homes. Subsequently, in each chosen home, a woman of childbearing age and one individual from each age group were randomly selected, with adjustments made based on survey completion. The surveys included general information

Topic	Encuesta Nacional de Salud y Nutrición
	for all household members, as well as anthropometric measurements, while a subsample of 50% underwent specific measurements of biochemical indicators and consumer surveys.
Sample size	92502 individuals, with 21482 completing the biochemistry survey
Sample size eligible for analysis*	6-23 months: 787<br>24-59 months: 1,260
Intervention	None

* Defined as having non-missing haemoglobin, ferritin, and C-reactive protein.

2.9.5 Generation R

The Generation R Study is a population-based prospective cohort study spanning from fetal life to young adulthood, conducted in Rotterdam, the Netherlands. (8) Its primary aim is to discern both environmental and genetic factors influencing growth, development, and health throughout this life stage. Eligible mothers were invited to participate in the study at routine prenatal visits, and comprehensive assessments were were conducted during early, mid-, and late pregnancy, depending on gestational age at enrolment. A subset of mothers were also recruited at birth. The children constitute a birth cohort and the study plans to follow them until young adulthood. Ultimately, the insights derived from the Generation R Study are intended to inform the formulation of strategies aimed at enhancing health and healthcare for pregnant women and children.

Topic	Generation R
Location	The Netherlands
Study type	Prospective population-based longitudinal cohort study
Primary purpose	To identify early environmental and genetic causes of normal and abnormal growth, development and health from foetal life until young adulthood
Date range	Enrolment of mothers (i.e. pregnant women + women enrolled at birth) between April 2002 and January 2006.
Age range	Women of reproductive age
In/exclusion and/or Sampling strategy	Inclusion criteria mother: Mother resident in study area at delivery between April 2002 and January 2006; During the whole of pregnancy up to and including birth with delivery date
Sample size	8880 pregnant women (6748 early-, 1857 mid-, and 275 late-pregnancy)
Sample size eligible for analysis*	Trimester 1: 5,054<br>Trimester 2: 2,194<br>Trimester 3: 239
Intervention	None

* Defined as having non-missing haemoglobin.

2.9.6 HSE

The Health Survey for England (HSE) has been conducted yearly since 1991. (9, 16) HSE is cross-sectional survey of a nationally representative sample of adults and children living in private households in England. Each year, core modules of questions and measurements are supplemented by population boosts and/or or additional topics. Participants are visited by an interviewer who collects demographic and socioeconomic data; health and health-related behaviours; measures height and weight; and requests consent for data linkage. A nurse then visits participants who consent, to take additional measurements (eg, waist circumference, blood pressure), collect biological samples (eg, blood) and record information on medication use. Of all available years since 1991, only years 1998, (17) 2006, (18,

19) and 2009 (20, 21) included data on haemoglobin, ferritin, and C-reactive protein in the general population. In 1998 and 2006 the additional focus was on cardiovascular diseases, and in 2009 on kidney diseases.

**Topic**	**Health Survey for England, 1998**
Location	England
Study type	Cross-sectional survey
Primary purpose	To measure health and health-related behaviours in adults and children living in private households in England
Time period	January 1998 to December 1998
Dates of fieldwork	January 1998 to April 1999
Age range	Adults (aged 16 and over) and children (aged 2-15 years)
In/exclusion and/or Sampling strategy	Multi-stage stratified random probability sample of households
Sample size	19,654
Sample size eligible for analysis*	18-65 years (men): 3,835<br>18-65 years (female): 4,189
Intervention	None
Version of data set	7 April 2010 (2nd Edition)

* Defined as having non-missing haemoglobin, ferritin, and C-reactive protein.

**Topic**	**Health Survey for England, 2006**
Location	England
Study type	Cross-sectional survey
Primary purpose	To measure health and health-related behaviours in adults and children living in private households in England.
Time period	January 2006 to December 2006
Date of fieldwork	January 2006 to May 2007
Age range	Adults (aged 16 and over) and children (aged 0-15 years). A boost sample of children aged 2-15 was also included.
In/exclusion and/or Sampling strategy	Multi-stage stratified random probability sample of households
Sample size	21,399
Sample size eligible for analysis*	18-65 years (men): 2,569<br>18-65 years (female): 3,026
Intervention	None
Version of data set	21 July 2011 (4th Edition)

* Defined as having non-missing haemoglobin, ferritin, and C-reactive protein.

**Topic**	**Health Survey for England, 2009**
Location	England
Study type	Cross-sectional survey
Primary purpose	To measure health and health-related behaviours in adults and children living in private households in England.
Time period	January 2009 to December 2009
Date of fieldwork	January 2009 to April 2010
Age range	Adults (aged 16 and over) and children (aged 0-15 years)
In/exclusion and/or Sampling strategy	Multi-stage stratified random probability sample of households
Sample size	8606

Sample size eligible for analysis*	18-65 years (men): 804<br>18-65 years (female): 892
Intervention	None
Version of data set	21 January 2015 (3rd Edition)

* Defined as having non-missing haemoglobin, ferritin, and C-reactive protein.

2.9.7 NHANES

The National Health and Nutrition Examination Survey (NHANES) (10, 22) has been conducted in the United States on a continuous basis and are grouped into NHANES cycles: NHANES II (1976-1980); NHANES III phase 1 (1988-1991); NHANES III phase 2 (1991-1994); and continuous two-year cycles beginning with 1999-2000 and continuing to the present. The surveys collect data on the health and nutritional status of the civilian noninstitutionalized population of the United States, using a combination of interviews, physical examinations, and laboratory analysis of biological specimens. Of all years available since 1999, years 2011-2012 and 2013-2014 did not have ferritin and C-reactive protein available and the two-year cycle 2019-2020 was affected by the COVID-19 pandemic.

<table>
<tr><th>Topic</th><th>National Health and Nutrition Examination Survey</th></tr>
<tr><td>Location</td><td>United States</td></tr>
<tr><td>Study type</td><td>Survey</td></tr>
<tr><td>Primary purpose</td><td>To assess the health and nutritional status of adults and children in the United States</td></tr>
<tr><td>Date range</td><td>1999-2000; (23) 2001-2002; (24) 2003-2004; (25) 2005-2006; (26) 2007-2008; (27) 2009-2010; (28) 2015-2016; (29) 2017-2018 (30)</td></tr>
<tr><td>Age range</td><td>All ages. Laboratory measurements were not obtained in all ages and sexes and varied between surveys.</td></tr>
<tr><td>In/exclusion and/or Sampling strategy</td><td>A stratified, multistage probability sample of the civilian noninstitutionalized U.S. population</td></tr>
<tr><td>Sample size</td><td>1999-2000: 9,965; 2001-2002: 11,039; 2003-2004: 10,122; 2005-2006: 10,348; 2007-2008: 10,149; 2009-2010: 10,537; 2015-2016: 9,971; 2017-2018: 9,254</td></tr>
<tr><td>Sample size eligible for analysis*</td><td>
<table>
<tr><th></th><th>18-65y Men</th><th>18-65y Women</th><th>6-23m</th><th>24-59m</th><th>5-11y</th><th>12-17y Male</th><th>12-17y Female</th></tr>
<tr><td>1999-2000</td><td>1,673</td><td>1,944</td><td>0</td><td rowspan="8">2,559</td><td rowspan="8">3,030</td><td>836</td><td>793</td></tr>
<tr><td>2001-2002</td><td>1,973</td><td>2,160</td><td>0</td><td>820</td><td>879</td></tr>
<tr><td>2003-2004</td><td>0</td><td>1,455</td><td rowspan="6">530</td><td>0</td><td>730</td></tr>
<tr><td>2005-2006</td><td>0</td><td>1,606</td><td>0</td><td>732</td></tr>
<tr><td>2007-2008</td><td>0</td><td>1,434</td><td>0</td><td>383</td></tr>
<tr><td>2009-2010</td><td>0</td><td>1,703</td><td>0</td><td>422</td></tr>
<tr><td>2015-2016</td><td>0</td><td>1,485</td><td>0</td><td>420</td></tr>
<tr><td>2017-2018</td><td>1,854</td><td>2,059</td><td>383</td><td>366</td></tr>
</table>
</td></tr>
<tr><td>Intervention</td><td>None</td></tr>
</table>

* Defined as having non-missing haemoglobin, ferritin, and C-reactive protein. y=year, m=month.

2.9.8 TARGet Kids!

The Applied Research Group for Kids (TARGet Kids!) is an ongoing longitudinal study spanning from birth to adolescence, aiming to longitudinally assess the long-term health and development of Canadian children while leveraging the country's extensive primary care practice model. (11), (31) The research focuses on identifying factors that may impact children's health, growth, and development, contributing valuable insights to primary health care improvement. Children are enrolled during routine well-child visits, and data collection during each visit includes

demographic information, physical measurements, lifestyle factors, child behavior and developmental screening, and a blood sample. These data are collected twice a year until age 2 and annually until age 10. (11, 32)

**Topic**	**TARGet Kids!**
Location	Canada
Study type	Open longitudinal cohort study of healthy children
Primary purpose	To link early life exposures to health problems including obesity, micronutrient deficiencies and developmental problems. The overarching goal is to improve the health of Canadians by optimizing growth and developmental trajectories through preventive interventions in early childhood.
Date range	Started in June 2008. There is no official end date, children will be followed-up as long as they are attending their annual check-ups with their current physician and until they are adolescents. The date range in the data shared for the purpose of this manuscript is January 2010 to December 2019.
Age range	Birth till adolescence
In/exclusion and/or Sampling strategy	Inclusion criteria:<br>Families with children under 6 years of age who are healthy as reported by their parents (including children with asthma<br><br>Exclusion criteria:<br>Children with associated health conditions affecting growth (e.g. failure to thrive, cystic fibrosis), children with any acute or chronic conditions (other than asthma and high functioning autism), children with severe developmental delay and families who are unable to communicate in English
Sample size	Over 11, 000 participants
Sample size eligible for analysis*	6-23 months: 1,671<br>24-59 months: 2,050<br>5-11 years: 979
Intervention	None

* Defined as having non-missing haemoglobin, ferritin, and C-reactive protein at the first study visit.

2.10 Statistical Analyses

Individual demographic, clinical, laboratory and hematology data were accessible to specific authors for all data sources as outlined in the contributions statement. Individual data of national population health surveys HSE (web URL: beta.ukdataservice.ac.uk/datacatalogue/series/series?id=2000021), NHANES (web URL: cdc.gov/nchs/nhanes), CHNS (web URL: cpc.unc.edu/projects/china), and ENSANUT (web URL: salud.gob.ec/encuesta-nacional-de-salud-y-nutricion-ensanut) were downloaded from the relevant websites. Individual data of the randomized controlled BRISC trial (web URL: wehi.edu.au/people/sant-rayn-pasricha/trials/brisc) and the prospective population-based longitudinal cohort study TARGet Kids! (Web URL: targetkids.ca) were obtained from the study investigators. Individual data of AHS (web URL: abs.gov.au/ausstats/abs@.nsf/mf/4363.0.55.001), consisting of National Health Survey (NHS) and National Nutrition and Physical Activity Survey (NNPAS), was only accessible by the joint first authors through the Australian Bureau of Statistics (ABS) DataLab environment. Analysis output (e.g., centiles) could be retrieved outside of the secure DataLab for public use only after clearance for release from the ABS. Finally, individual data of the prospective population-based longitudinal cohort Generation R study (web URL: generationr.nl) was accessible to the Generation R investigators while analysis output (e.g., centiles) was available to the authors. Further citation details on HSE, NHANES, and AHS are provided in the table below.

Data Source	Citation
HSE	– *Survey 1998*: National Centre for Social Research, University College London. Department of Epidemiology and Public Health. (2010). Health Survey for England, 1998. [data collection]. 5th Release. UK Data Service. SN: 4150, DOI: http://doi.org/10.5255/UKDA-SN-4150-1<br>– *Survey 2006*: National Centre for Social Research, University College London, Department of Epidemiology and Public Health. (2011). Health Survey for England, 2006. [data collection]. 4th Edition. UK Data Service. SN: 5809, DOI: http://doi.org/10.5255/UKDA-SN-5809-1<br>– *Survey 2009*: University College London, Department of Epidemiology and Public Health, National Centre for Social Research. (2015). Health Survey for England, 2009. [data collection]. 3rd Edition. UK Data Service. SN: 6732, DOI: http://doi.org/10.5255/UKDA-SN-6732-2
NHANES	Centers for Disease Control and Prevention (CDC). National Center for Health Statistics (NCHS). National Health and Nutrition Examination Survey Data. Hyattsville, MD: U.S. Department of Health and Human Services, Centers for Disease Control and Prevention, [appropriate year] [web URL] as per below:<br>– [1999-2000] [wwwn.cdc.gov/nchs/nhanes/continuousnhanes/default.aspx?BeginYear=1999]<br>– [2001-2002] [wwwn.cdc.gov/nchs/nhanes/continuousnhanes/default.aspx?BeginYear=2001]<br>– [2003-2004] [wwwn.cdc.gov/nchs/nhanes/continuousnhanes/default.aspx?BeginYear=2003]<br>– [2005-2006] [wwwn.cdc.gov/nchs/nhanes/continuousnhanes/default.aspx?BeginYear=2005]<br>– [2007-2008] [wwwn.cdc.gov/nchs/nhanes/continuousnhanes/default.aspx?BeginYear=2007]<br>– [2009-2010] [wwwn.cdc.gov/nchs/nhanes/continuousnhanes/default.aspx?BeginYear=2009]<br>– [2015-2016] [wwwn.cdc.gov/nchs/nhanes/continuousnhanes/default.aspx?BeginYear=2015]<br>– [2017-2018] [wwwn.cdc.gov/nchs/nhanes/continuousnhanes/default.aspx?BeginYear=2017]
AHS	– Australian Bureau of Statistics (2011-12) National Health Survey, Australia [DataLab] (accessed April 2022-23)<br>– Australian Bureau of Statistics (2012-13) National Nutrition and Physical Activity Survey, Australia [DataLab] (accessed April 2022-23)

HSE = Health Survey for England; NHANES = National Health and Nutrition Examination Survey; AHS = Australian Health Survey (consisting of National Health Survey and National Nutrition and Physical Activity Survey).

In the absence of person identifiable data, it was assumed that each participant of the HSE, NHANES, and AHS national population health surveys represents a unique individual.

2.10.1 General Principles

All analyses used haemoglobin values as provided by the data source and were reported in g/L. The estimates of the 2·5th and 5th centiles along with confidence intervals were obtained for the healthy reference sample for each data source. The Clinical & Laboratory Standards Institute recommends two-sided 90% confidence intervals be calculated for each reference limit. (33)

With the exception of TARGet Kids!, a single haemoglobin value was available by age-group and sex or trimester of pregnancy. The TARGet Kids! study was a longitudinal cohort study with participants followed between birth to adolescence. Children under 6 years of age were eligible to be included in the study if reported healthy by their parents (including children with asthma). Given that health was central to the estimation of the centiles, only the haemoglobin measurement from the first study visit per participant was used to estimate the centile.

For each healthy reference sample, the assumption of normality was checked by examining histograms, Q-Q plots comparing the theoretical quantiles of the normal distribution to the sample quantiles, skewness and kurtosis. All analyses were run with and without outliers. Outliers were identified using Tukey's method. (34, 35) For discrete threshold estimation, outlying values were haemoglobin values < 25th haemoglobin centile – 3 × interquartile range (IQR) and haemoglobin values > 75th haemoglobin centile + 3 × IQR, whereby IQR is the difference between the 75th and 25th centiles. For continuous threshold estimation, haemoglobin in the above description was replaced by the

residuals from multivariable fractional polynomial regression of haemoglobin on age after adjusting for sex. Results for the discrete and continuous thresholds are presented excluding outlying values only.

2.10.2 Sample Size

For each data source, the sample size of the healthy reference sample was determined by the number of participants who fulfilled the criteria of an apparently healthy individual. For children in the age-groups of 6-23 months, 24-59 months, and 5-11 years, the available data or all NHANES survey cycles were pooled due to the small sample size by survey cycle. For the Australian NHS and NNPAS, no centiles could be extracted if the sample size was less than 200 ($2{\cdot}5^{th}$ centile) or 100 (5th centile) as per ABS requirements for output clearance. For adolescents 12-17 years, NHS and NNPAS were pooled to meet this sample size requirement for output clearance. For all other data sources, centiles were not obtained if the sample size was less than 20. No hypothesis testing was undertaken.

2.10.3 Survey Weighting and Design

In national population health surveys (NHANES, HSE, CHNS, ENSANUT), we aimed to account for the sampling weights and sampling design during the analysis. Accounting for the survey design (i.e., cluster and/or stratum to which the individual belongs) is required to correctly estimate standard errors and for correct inference. The weighting adjusts the results from a sample survey to infer results for the total target population. To do this, a 'weight' is allocated to each sample unit (person or household), indicating how many population units are represented by the sample unit. In general, the weights account for selection and non-response bias. In the table below, the design, cluster (primary sampling unit) and strata, and weight variables used in centile estimation described later are provided.

Design and weight variables used in centile estimation for each national population health survey.

Variable Type[1]	Variable Name[3]	Description
NHANES (Survey 1999-2000, 2001-02, 2003-04, 2005-06, 2007-08, 2009-10, 2015-16, 2017-18)		
Cluster/PSU[2]	sdmvpsu	County
Strata	sdmvstra	Geography (e.g., census region), metropolitan statistical area status, and various population demographics.
Weights[5]	wtmec2yr	Generated for individuals who had physical examinations and laboratory tests in mobile examination centers (MEC).
HSE (Survey 2006 and 2009; no design and weight variables applicable for 1998)		
Cluster/PSU	psu	Postcode sectors
Strata	cluster	Local authority and socio-economic group
Weights	wt_blood	Generated for individuals aged 16 and over who had a nurse visit and were eligible for a blood sample.
CHNS (Survey 2009)		
Cluster/PSU	commid	Community/county
Strata	N/A[4]	Information unavailable/not released to the public.
Weights	N/A	Information unavailable/not released to the public.
ENSANUT (Survey 2011-13)		
Cluster/PSU	idsector	Census tracts
Strata	area	Rural and urban areas
Weights	pw	Household expansion factor/weight

[1]Survey – National population health survey; [2]PSU – Primary sampling unit; [3]Variable Name – Name of variable in dataset; [4]N/A – Not available [5]For each NHANES survey cycle, the weight variables were implemented following the Weighting Module (web URL: wwwn.cdc.gov/nchs/nhanes/tutorials/weighting.aspx) and Variance Estimation Module (web URL: wwwn.cdc.gov/nchs/nhanes/tutorials/varianceestimation.aspx).

As described in Section 2.10.2, for children in the age-groups of 6-23 months, 24-59 months, and 5-11 years, NHANES survey cycles were combined due to the small sample size by survey cycle. The weights for the combined NHANES surveys were derived following the "Constructing Weights for Combined NHANES Survey Cycles" section of the Weighting Module (web URL: wwwn.cdc.gov/nchs/nhanes/tutorials/weighting.aspx). Specifically:

- 6-23 months: The 6 survey cycles from 2003-2018 were combined. The new multi-year sample weight for the combined 6 survey cycles was computed by dividing the 2-year sample weights for each cycle (wtmec2yr) by the number of two-year cycles in the analysis, i.e., 6.
- 24-59 months and 5-11 years: The 8 survey cycles from 1999-2018 were combined. As instructed on the NHANES website, the 4-year weight (wtmec4yr) was used to combine surveys for 1999-2002 and the 2-year weight (wtmec2yr) was used to combine each additional survey cycle. The formulas used to derive the new multi-year sample weight (wtmec16yr) for the combined 8 survey cycles were:
    - wtmec16yr = wtmec4yr × (2/8), if survey cycle in (1999-2000, 2001-2002)
    - wtmec16yr = wtmec2yr × (1/8), if survey cycle in (2003-2004, 2005-2006, 2007-2009, 2009-2010, 2015-2016, 2017-2018)

Summary statistics of participant characteristics (Section 3) and violations of exclusion criteria (Section 4) were presented unweighted for the national population health surveys.

2.10.4 Discrete Thresholds

The estimates of the 2·5th and 5th discrete centiles along with confidence intervals were obtained for each data source by age-group (6-23 months, 24-59 months, 5-11 years), age-group and sex (12-17 years, 18-65 years), or trimester of pregnancy (pregnant women [18-45 years]).

Simple Random Samples

The parametric theoretical centile of a Gaussian distribution, with corresponding 90% confidence interval, was used to estimate the centile. (36) In addition, we explored a Box-Cox transformation to the reference distribution prior to calculating the theoretical centile and corresponding confidence interval. (37) The simulation study conducted in Daly et al., (36) found that the parametric approach provided the least biased and most precise estimates for normally distributed reference distributions compared to the other methods examined (non-parametric and robust). Since the reference distribution appeared reasonably normal, the parametric method without Box-Cox transformation was utilized as the preferred method instead of non-parametric and robust methods.

Complex Surveys

For the national population health surveys NHANES, HSE, CHNS, and ENSANUT both unweighted and survey-weighted centile estimates were calculated. Unweighted centile estimates were calculated using the parametric theoretical centile. Survey-weighted centile estimates were calculated using survey-weighted quantile regression. The implementation of survey-weighted quantile regression proceeded the same way as described in Section 2.10.5, except

the survey-weighted quantile regression model only included a single intercept term and was performed using the rq function in R's quantreg package with the default model fitting algorithm (i.e., modified version of the Barrodale and Roberts (br) algorithm for l_1-regression). Standard errors for the intercept term were derived from 1000 replicate weights generated using Canty and Davidson's bootstrap and two-sided 90% confidence intervals derived assuming a normal approximation. (38) The selection of 1000 bootstrap replicates follow recommendations provided in section "3.4. Bootstrap" of Canty and Davison (38) and sections "19.2 Standard error estimation" and "19.3 Percentile estimation" of Efron and Tibshriani 1994. (39) Specifically for NHANES, the 90% confidence intervals for the survey weighted centile estimates were obtained as follows:

- The as.svrepdesign function was applied to the svydesign object created for a NHANES survey cycle to generate 1000 bootstrap replicate weights using the Canty and Davison bootstrap method. (38)
- The withReplicates function available in R's survey package was then used to perform weighted quantile regression using the bootstrap replicate weights generated by as.svrepdesign and to produce a replicate-based estimate of the standard error. Further details regarding the standard error calculation can be found in "Complex Surveys: A Guide to Analysis using R . Thomas Lumley", (41) sections "2.3.1 Specifying replicate weights to R" and section "2.3.2 Creating replicate weights in R".
- Only the standard error (not a confidence interval) is produced by the withReplicates function, and a normal approximation was used to derive approximate 90% confidence intervals (sections 2.8 & 2.15 of Cochran 1977) (42) as follows: estimate from weighted quantile regression +/- critical value for a 90% confidence interval (=1.64) × replicate-based standard error.

The unweighted centile estimate was obtained to explore the sensitivity of the results to sample size. In addition, quantile regression makes no assumptions about the distribution of the residuals. (43)

Centile estimation from complex survey data using the survey-weighted approach implemented by R's svyquantile function was also explored. The 90% confidence intervals for the survey weighted centile estimates were calculated using Woodruff's method, (40) and involves calculating the quantile from the empirical cumulative distribution function (ECDF), estimating a confidence interval for the proportion of values below the quantile, and then transforming that interval using the ECDF. For small centiles (e.g., 2·5th, and occasionally 5th) and small to moderate sample sizes, the lower limit of the 90% confidence interval could not be calculated using Woodruff's method. Since this problem was not observed for quantile regression, it was considered the preferred method to obtain the survey-weighted centile estimate.

Pooling

Centile estimates (discrete thresholds) pooled across all data sources using the combined healthy reference sample were calculated using fixed effect and random effects meta-analyses. The fixed effect approach assumes that the variation in the centile estimates is due to chance alone, and the pooled centile estimate is a weighted average of the centile estimates with weights equal to the square of the inverse of the standard error of the estimate. The random

effects approach assumes that the variation in the centile estimates is due to more than chance alone, and that between study differences are also contributing. Like the fixed effects approach, the pooled centile estimate from the random effects approach is a weighted average of the centile estimates, but with weights equal to inverse of the total variation (between study variation + within study/residual variation). The fixed and random effects meta-analyses were performed in R using the metafor package. (44)

2.10.5 Continuous Thresholds

The estimates of the 2·5th and 5th continuous centiles along with confidence intervals were obtained for all data sources combined (excluding NHS and NNPAS) by age-group (6--59 months, 5-11 years, 12-17 years, 18-65 years) (and sex if applicable). NHS and NNPAS data could not be included because individual data was only accessible via the ABS secure DataLab. No continuous thresholds were obtained for pregnant women. Centile estimates (continuous thresholds) were obtained using the method described below. No survey-weighting was applied to the national population health surveys.

The method proposed in Hoq et al. 2019 (45) was used to estimate age-specific continuous haemoglobin thresholds. In brief, Hoq's method implements the following steps:

1. Identify the best fitting multivariable fractional polynomial (MFP) model for the mean values of haemoglobin using Royston's method (8). (46) The procedure was implemented using R's mfp function (Benner 2005 and Sauerbrei et al. 2006). (47, 48) Fractional polynomial models of degree 2 (degrees of freedom 4) were considered for age. The set of powers available in the mfp function is {-2, -1, -0.5, 0, 0.5, 1, 2, 3,} where a power of 0 denotes the log transformation of a continuous covariates; non-zero values denote a power transformation of the following form $\beta_1 age^p$, where β_1 denotes a regression coefficient, p denotes a power selected from the set of available power values. Fractional polynomials of degree 2 allow age to be modelled by two terms and include the formulations presented below whereby β_1 and β_2 are regression coefficients, p_1 and p_2 are powers that can take values in the set of available powers.

$$\beta_1 age^{p_1} + \beta_2 age^{p_2}, \text{if } p_1 \neq p_2$$
$$\beta_1 age^{p_1} + \beta_2 age^{p_1} \log(age), \text{if } p_1 = p_2$$

A closed testing selection procedure is used to choose among the 44 possible fractional polynomial models (8 degree 1 models and 36 degree 2 models) and is implemented by R's mfp function. As originally described in Ambler and Royston 2001, (49) the closed testing selection procedure maintains approximately the correct Type I error rate for each covariate tested. A nominal level of significance of 0.05 was selected. In the closed testing procedure, an approximate P-value calculation based on assuming the difference of the deviance (-2 × log-likelihood) between two models has a chi-squared distribution is used to compare models.

2. Likelihood ratio test for an interaction between sex and the fractional polynomial representation of age in the model identified in step 1. Include the age-by-sex interaction term(s) in the model if they are statistically significant at the nominal level of significance of 0·05.
3. Quantile regression was used. The terms included in the quantile regression model were constrained to be the same as those included in the MFP model of the mean (steps 1 and 2). Age-dependent haemoglobin predictions were obtained from the quantile regression model and two-sided 90% bootstrap centile confidence intervals by using the percentile bootstrap method implemented by the predict command available in R's quantreg package. (50, 51) 1000 bootstrap samples were generated.

The residuals from the MFP were examined to assess the fit of the MPF model for the mean haemoglobin values and to identify outliers using Tukey's method. (35) The results from quantile regression modelling were presented in plots showing how the predicted centiles change with age (and by sex if applicable), superimposed onto a scatter plot of haemoglobin versus age (in years or months).

Provided in the table below are the resulting best fitting equations to the pooled haemoglobin levels (g/L) with outliers excluded (outlier detection described above and in Section 2.10.1 General Principles). For age groups 12-17 years and 18-65 years, the test for an interaction between sex and the fractional polynomial representation of age were found to be statistically significant. The continuous thresholds predicted from these models agree with the trends in the observed levels for these age groups, which exhibit different trends in observed levels with age for males and females.

Best fitting equations to the pooled haemoglobin levels (g/L) with outliers excluded.

Age Group	Equation for 5th centile*
6-59 months	$120.29 - 5.63 \times \left(\frac{age}{100}\right)^{-0.5} - 0.87 \times sex$
5-11 years	$108.06 + 7.84 \times \left(\frac{age}{100}\right) - 0.71 \times sex$
12-17 years	$81.80 + 26.67 \times \left(\frac{age}{100}\right) + 34.57 \times sex - 24.91 \times \left(\frac{age}{100}\right) \times sex$
18-65 years	$130.69 + 1.23 \times \left(\frac{age}{100}\right)^{-1} - 14.49 \times sex - 0.54 \times \left(\frac{age}{100}\right)^{-1} \times sex$

*Age in months for age groups 6-59 months, 5-11 years and 12-17 years. Age in years for 18-65 years. Automatic scaling of age by R's mfp function (i.e., dividing by 100) is performed to avoid numerical problems caused by cubic, squared, and reciprocal terms. Replace sex with "1" if subject is female. Otherwise, replace sex with "0" if subject is male.

2.11 Genetic Analyses

We summarised three existing large-scale, multi-ancestry GWAS summary statistics associated with haemoglobin concentration trait. (52-54) Chen and Genes & Health analyzed single nucleotide polymorphisms (SNPs), whilst Wheeler considered structural variations (SVs). In each study, individuals were classified as being genetically similar to one of the five "super-populations" defined as part of the 1000 Genomes study: European (EUR), East Asian (EAS), African (AFR), Hispanic/Latino, and South Asian (SAS), allowing for assessment of the effects of haemoglobin-associated variants on haemoglobin concentrations across ancestry groups. Details are given in Table 7.1.

Population-specific effect size and allele frequencies were extracted for all variants significantly associated with haemoglobin, in one or more ancestral population. To account for linkage disequilibrium (LD), we sought to select one representative SNP per genetic region for our summaries.

Chen reported independent variants associated with haemoglobin concentrations defined using iterative conditional analyses approach, so we extracted their predefined lists of independent associated SNPs. For the Genes & Health study results, we performed LD clumping with an $r^2<0{\cdot}1$ and a clumping window size of 500 kb to identify independent signals. We examined the overlap of independent signals from the Genes & Health study and Chen analyses, with signals defined as overlapping where the identified representative SNPs from each study were in linkage disequilibrium, with $r^2>0{\cdot}1$. Where signals were overlapping, we selected the top SNP defined in Chen as the representative SNP for that signal. Both utilized GWAS applied rank-based inverse rank normal transformation (IRNT) to the haemoglobin concentration measurements, prior to association with genetic variants. Effect sizes stated in the text are as reported in terms of the IRNT trait. To provide a better clinical interpretation of the reported effect size estimates, on the plots we also include a scale giving approximate effects in terms of units of haemoglobin measurement (in g/L). We used the UK Biobank (UKBB) population-based cohort (Application Number: 36610; Data-Field 30020) for these approximations by multiplying the IRNT effect size by the standard deviation of haemoglobin concentration in the UKBB cohort. We also considered structural variants identified in Wheeler. (53) We extracted ancestry specific frequencies for the identified SVs, but effect estimates were only available from a combined ancestry analysis. This study performed LD and conditional analyses for trait-associated SVs to previous reported GWAS variants to determine whether these SVs were being tagged by the GWAS SNPs.

We accessed gene-based summary statistics from the AstraZeneca PheWAS Portal. (55) Gene-phenotype associations were tested with multiple collapsing models. (55) We extracted gene associations of haemoglobin concentrations using the collapsing model Ptv5pcnt (defined as protein-truncating variants; PTVs, with MAF ≤ 5% both within the UKBB cohort and gnomAD). A gene with a p-value less than a Bonferroni corrected threshold of 0·05 divided by the 18,762 genes (55) tested ($P < 2{\cdot}665 \times 10^{-6}$) was considered significant. For significant genes, we further restricted to those identified as clinically relevant causes of rare anaemia from the Genomics England PanelApp 'Green' gene list (Version 3.0) (56) and investigated the frequency distributions of predicted loss-of-function (pLoF) variants in different populations of gnomAD. (57, 58) For each gene, we also estimated a cumulative frequency of pLoF variants, within each population, as the sum of alternate allele count for each variant, divided by the mean number of genotypes available across all variants in the gene.

2.12 Software

Reference samples were derived using Stata version 16.1, (59) R version 4.2.0 (Generation R), (60) or Stata version 17.1 (NHS and NNPAS). (61) Estimation of the haemoglobin thresholds was performed using R version 4.1.1, (62) R version 4.2.0 (Generation R), (60) or Stata version 17.1 (NHS and NNPAS). (61) An overview of the R packages

used as part of the discrete and continuous threshold estimation as well as the pooling are listed in the table below. In Stata, the command *centile* with option normal was used to obtain the discrete thresholds.

Overview of R packages used as part of the threshold estimation.

Package	Citation
tidyverse	Wickham et al., (2019). Welcome to the tidyverse. Journal of Open Source Software, 4(43), 1686. https://doi.org/10.21105/joss.01686
magrittr	Stefan Milton Bache and Hadley Wickham (2022). magrittr: A Forward-Pipe Operator for R. R package version 2.0.3. https://CRAN.R-project.org/package=magrittr
haven	Hadley Wickham, Evan Miller and Danny Smith (2022). haven: Import and Export 'SPSS', 'Stata' and 'SAS' Files. R package version 2.5.0. https://CRAN.R-project.org/package=haven
ggpubr	Alboukadel Kassambara (2020). ggpubr: 'ggplot2' Based Publication Ready Plots. R package version 0.4.0. https://CRAN.R-project.org/package=ggpubr
cowplot	Claus O. Wilke (2020). cowplot: Streamlined Plot Theme and Plot Annotations for 'ggplot2'. R package version 1.1.1. https://CRAN.R-project.org/package=cowplot
MKinfer	Kohl M (2020). _MKinfer: Inferential Statistics_. R package version 0.6 http://www.stamats.de
quantreg	Roger Koenker (2022). quantreg: Quantile Regression. R package version 5.88. https://CRAN.R-project.org/package=quantreg
survey	T. Lumley (2004) Analysis of complex survey samples. Journal of Statistical Software 9(1): 1-19 T. Lumley (2010) Complex Surveys: A Guide to Analysis Using R. John Wiley and Sons.
jtools	Long JA (2020). _jtools: Analysis and Presentation of Social Scientific Data_. R package version 2.1.0. https://cran.r-project.org/package=jtools
moments	Lukasz Komsta and Frederick Novomestky (2015). Moments: Moments, cumulants, skewness, kurtosis and related tests. R package version 0.14. https://CRAN.R-project.org/package=moments
kableExtra	Hao Zhu (2021). kableExtra: Construct Complex Table with 'kable' and Pipe Syntax. R package version 1.3.4. https://CRAN.R-project.org/package=kableExtra
mfp	Gareth Ambler and Axel Benner (2022). mfp: Multivariable Fractional Polynomials. R package version 1.5.2.2. https://CRAN.R-project.org/package=mfp
lmtest	Achim Zeileis, Torsten Hothorn (2002). Diagnostic Checking in Regression Relationships. R News 2(3), 7-10. https://CRAN.R-project.org/doc/Rnews/
referenceIntervals	Daniel Finnegan (2020). referenceIntervals: Reference Intervals. R package version 1.2.0. https://CRAN.R-project.org/package=referenceIntervals
car	John Fox and Sanford Weisberg (2019). An R Companion to Applied Regression, Third Edition. Thousand Oaks CA: Sage. https://socialsciences.mcmaster.ca/jfox/Books/Companion/
Ecfun	Spencer Graves (2021). Ecfun: Functions for 'Ecdat'. R package version 0.2-5. https://CRAN.R-project.org/package=Ecfun
moments	Lukasz Komsta and Frederick Novomestky (2015). moments: Moments, cumulants, skewness, kurtosis and related tests. R package version 0.14. https://CRAN.R-project.org/package=moments
nortest	Juergen Gross and Uwe Ligges (2015). nortest: Tests for Normality. R package version 1.0-4. https://CRAN.R-project.org/package=nortest
metafor	Viechtbauer, W. (2010). Conducting meta-analyses in R with the metafor package. Journal of Statistical Software, 36(3), 1-48. https://doi.org/10.18637/jss.v036.i03
readxl	Wickham H, Bryan J (2022). _readxl: Read Excel Files_. R package version 1.4.0. https://CRAN.R-project.org/package=readxl
gridExtra	Auguie B (2017). _gridExtra: Miscellaneous Functions for "Grid" Graphics_. R package version 2.3, https://CRAN.R-project.org/package=gridExtra
expss	Demin G (2023). _expss: Tables, Labels and Some Useful Functions from Spreadsheets and 'SPSS' Statistics_. R package version 0.11.6, https://CRAN.R-project.org/package=expss
officer	Gohel D (2023). _officer: Manipulation of Microsoft Word and PowerPoint Documents_. R package version 0.6.2, https://CRAN.R-project.org/package=officer
flextable	Gohel D, Skintzos P (2023). _flextable: Functions for Tabular Reporting_. R package version 0.9.2, https://CRAN.R-project.org/package=flextable
finalfit	Harrison E, Drake T, Pius R (2023). _finalfit: Quickly Create Elegant Regression Results Tables and Plots when Modelling_. R package version 1.0.7, https://CRAN.R-project.org/package=finalfit
labelled	Larmarange J (2023). _labelled: Manipulating Labelled Data_. R package version 2.12.0, https://CRAN.R-project.org/package=labelled
foreign	R Core Team (2022). _foreign: Read Data Stored by 'Minitab', 'S', 'SAS', 'SPSS', 'Stata', 'Systat', 'Weka', 'dBase', ..._. R package version 0.8-84, https://CRAN.R-project.org/package=foreign
gtsummary	Sjoberg DD, Whiting K, Curry M, Lavery JA, Larmarange J. Reproducible summary tables with the gtsummary package. The R Journal 2021;13:570–80. https://doi.org/10.32614/RJ-2021-053.

Genetic analyses was performed in R version 4.1.3 (60) using R packages tidyverse (version 1.3.2), data.table (version 1.14.2), ieugwasr (version 0.1.5), ggplot2 (version 3.4.1), ggrepel (version 0.9.1), UpSetR (version 1.4.0), (63) and ComplexHeatmap (version 2.13.1).(64)

2.13 Ethics Approvals

Each individual data source obtained ethics approval before conduct of the study/survey, as outlined in the table below.

Data Source	Ethics
TARGet Kids!	The study was approved by the Research Ethics Board of The Hospital for Sick Children, Toronto, Ontario, Canada.
Generation R	The general design, all research aims and the specific measurements in the Generation R Study have been approved by the Medical Ethical Committee of Erasmus MC, University Medical Center Rotterdam, The Netherlands.
AHS	The AHS biomedical measures component forms the National Health Measures Survey (NHMS). Ethics approval for the NHMS was granted by the Australian Government Department of Health and Ageing Departmental Ethics Committee in February 2011.
NHANES	The NHANES protocol was developed and reviewed to be in compliance with the Department of Health and Human Services (HHS) Policy for Protection of Human Research Subjects (45 CFR part 46), guidance/45cfr46.html. It was approved by the National Center for Health Statistics (NCHS), United States, Research Ethics Review Board (ERB) and underwent annual review.
HSE	Ethical approval was obtained from the London Multi-centre Research Ethics Committee, England.
BRISC	The trial was approved by the Melbourne Health Human Research Ethics Committee, Melbourne, Victoria, Australia (2016.269); the Ethical Review Committee of icddr,b (PR-16063); and the Directorate General of Drug Administration, Ministry of Health and Family Welfare, Dhaka, Bangladesh.
ENSANUT	This survey was run with oversight from the National Institute of Statistics and Censuses, Ecuador.
CHNS	This is an international collaborative project between the Carolina Population Center at the University of North Carolina at Chapel Hill (United States); and the Chinese Center for Disease Control and Prevention (China).

TARGet Kids! = The Applied Research Group for Kids; AHS = Australian Health Survey (consisting of National Health Survey and National Nutrition and Physical Activity Survey); NHANES = National Health and Nutrition Examination Survey; HSE = Health Survey for England; BRISC = Benefits and Risks of Iron intervention in Children; ENSANUT = Encuesta Nacional de Salud y Nutrición; CHNS = China Health and Nutrition Survey.

3. Demographics

3.1 Demographics Adults (18-65 years) males

Table 3.1.1 US NHANES 1999-2000: Participant characteristics (Males) (18-65 years).

	Overall sample N=1,673	Excluded sample N=1,491 (89·1%)	Reference sample[1] N=182 (10·9%)
Age (years)	38·9 (15·0)	40·2 (14·8)	28·2 (12·1)
Sex			
Male	1,673/1,673 (100·0%)	1,491/1,491 (100·0%)	182/182 (100·0%)
BMI (kg/m^2)	27.4 (5.6)	27.8 (5.7)	24.1 (3.1)
Poverty	287/1,475 (19·5%)	253/1,322 (19·1%)	34/153 (22·2%)
Race and Hispanic Origin			
Mexican American	521/1,673 (31·1%)	434/1,491 (29·1%)	87/182 (47·8%)
Other Hispanic	95/1,673 (5·7%)	84/1,491 (5·6%)	11/182 (6·0%)
Non-Hispanic White	662/1,673 (39·6%)	610/1,491 (40·9%)	52/182 (28·6%)
Non-Hispanic Black	332/1,673 (19·8%)	305/1,491 (20·5%)	27/182 (14·8%)
Other Race - Including Multi-Racial	63/1,673 (3·8%)	58/1,491 (3·9%)	5/182 (2·7%)
Haemoglobin (g/L)	154.2 (11.0)	154.0 (11.2)	155.7 (9.2)
Anaemia (Haemoglobin<130g/L)			
No	1,637/1,673 (97·8%)	1,456/1,491 (97·7%)	181/182 (99·5%)
Yes	36/1,673 (2·2%)	35/1,491 (2·3%)	1/182 (0·5%)
Iron deficiency (Ferritin<15ug/L)			
No	1,658/1,673 (99·1%)	1,476/1,491 (99·0%)	182/182 (100·0%)
Yes	15/1,673 (0·9%)	15/1,491 (1·0%)	0/182 (0·0%)
Inflammation (C-Reactive Protein>5mg/L)			
No	1,421/1,673 (84·9%)	1,239/1,491 (83·1%)	182/182 (100·0%)
Yes	252/1,673 (15·1%)	252/1,491 (16·9%)	0/182 (0·0%)

US = United States, NHANES = National Health and Nutrition Examination Survey, BMI = Body Mass Index, SD = Standard Deviation.
Data are presented as unweighted mean (SD) for continuous measures, and unweighted n/total (%) for categorical measures based on participants with non-missing haemoglobin, ferritin, and C-reactive protein.
[1] Ferritin≥15 and ≤200ug/L and C-reactive protein≤5mg/L.

Table 3.1.2 US NHANES 2001-2002: Participant characteristics (Males) (18-65 years).

	Overall sample N=1,973	Excluded sample N=1,733 (87·8%)	Reference sample[1] N=240 (12·2%)
Age (years)	38·6 (14·5)	40·0 (14·3)	29·2 (11·8)
Sex			
Male	1,973/1,973 (100·0%)	1,733/1,733 (100·0%)	240/240 (100·0%)
BMI (kg/m^2)	27·5 (5·8)	27·9 (5·9)	24·3 (2·9)
Poverty	315/1,856 (17·0%)	265/1,630 (16·3%)	50/226 (22·1%)
Race and Hispanic Origin			
Mexican American	497/1,973 (25·2%)	397/1,733 (22·9%)	100/240 (41·7%)
Other Hispanic	89/1,973 (4·5%)	75/1,733 (4·3%)	14/240 (5·8%)
Non-Hispanic White	898/1,973 (45·5%)	817/1,733 (47·1%)	81/240 (33·8%)
Non-Hispanic Black	415/1,973 (21·0%)	378/1,733 (21·8%)	37/240 (15·4%)
Other Race - Including Multi-Racial	74/1,973 (3·8%)	66/1,733 (3·8%)	8/240 (3·3%)
Haemoglobin (g/L)	154·1 (11·4)	153·9 (11·7)	155·6 (9·1)
Anaemia (Haemoglobin<130g/L)			
No	1,919/1,973 (97·3%)	1,679/1,733 (96·9%)	240/240 (100·0%)
Yes	54/1,973 (2·7%)	54/1,733 (3·1%)	0/240 (0·0%)
Iron deficiency (Ferritin<15ug/L)			
No	1,939/1,973 (98·3%)	1,699/1,733 (98·0%)	240/240 (100·0%)
Yes	34/1,973 (1·7%)	34/1,733 (2·0%)	0/240 (0·0%)
Inflammation (C-Reactive Protein>5mg/L)			
No	1,678/1,973 (85·0%)	1,438/1,733 (83·0%)	240/240 (100·0%)
Yes	295/1,973 (15·0%)	295/1,733 (17·0%)	0/240 (0·0%)

US = United States, NHANES = National Health and Nutrition Examination Survey, BMI = Body Mass Index, SD = Standard Deviation.

Data are presented as unweighted mean (SD) for continuous measures, and unweighted n/total (%) for categorical measures based on participants with non-missing haemoglobin, ferritin, and C-reactive protein.

[1] Ferritin≥15 and ≤200ug/L and C-reactive protein≤5mg/L.

Table 3.1.3 US NHANES 2017-2018: Participant characteristics (Males) (18-65 years).

	**Overall sample N=1,854**	**Excluded sample N=1,705 (92·0%)**	**Reference sample[1] N=149 (8·0%)**
Age (years)	42·7 (14·7)	43·7 (14·5)	31·4 (12·0)
Sex			
Male	1,854/1,854 (100·0%)	1,705/1,705 (100·0%)	149/149 (100·0%)
BMI (kg/m^2)	29·4 (6·7)	29·8 (6·8)	24·9 (3·1)
Poverty	318/1,620 (19·6%)	289/1,493 (19·4%)	29/127 (22·8%)
Race and Hispanic Origin			
Mexican American	302/1,854 (16·3%)	271/1,705 (15·9%)	31/149 (20·8%)
Other Hispanic	177/1,854 (9·5%)	156/1,705 (9·1%)	21/149 (14·1%)
Non-Hispanic White	567/1,854 (30·6%)	542/1,705 (31·8%)	25/149 (16·8%)
Non-Hispanic Black	408/1,854 (22·0%)	379/1,705 (22·2%)	29/149 (19·5%)
Non-Hispanic Asian	287/1,854 (15·5%)	246/1,705 (14·4%)	41/149 (27·5%)
Other Race - Including Multi-Racial	113/1,854 (6·1%)	111/1,705 (6·5%)	2/149 (1·3%)
Haemoglobin (g/L)	150·3 (12·2)	150·2 (12·3)	152·0 (10·4)
Anaemia (Haemoglobin<130g/L)			
No	1,777/1,854 (95·8%)	1,632/1,705 (95·7%)	145/149 (97·3%)
Yes	77/1,854 (4·2%)	73/1,705 (4·3%)	4/149 (2·7%)
Iron deficiency (Ferritin<15ug/L)			
No	1,826/1,854 (98·5%)	1,677/1,705 (98·4%)	149/149 (100·0%)
Yes	28/1,854 (1·5%)	28/1,705 (1·6%)	0/149 (0·0%)
Inflammation (C-Reactive Protein>5mg/L)			
No	1,537/1,854 (82·9%)	1,388/1,705 (81·4%)	149/149 (100·0%)
Yes	317/1,854 (17·1%)	317/1,705 (18·6%)	0/149 (0·0%)

US = United States, NHANES = National Health and Nutrition Examination Survey, BMI = Body Mass Index, SD = Standard Deviation.

Data are presented as unweighted mean (SD) for continuous measures, and unweighted n/total (%) for categorical measures based on participants with non-missing haemoglobin, ferritin, and C-reactive protein.

[1] Ferritin≥15 and ≤200ug/L and C-reactive protein≤5mg/L.

Table 3.1.4 England HSE 1998: Participant characteristics (Males) (18-65 years).

	**Overall sample N=3,835**	**Excluded sample N=3,268 (85·2%)**	**Reference sample[1] N=567 (14·8%)**
Age (years)	42·3 (12·5)	42·8 (12·6)	39·7 (11·5)
Sex			
Male	3,835/3,835 (100·0%)	3,268/3,268 (100·0%)	567/567 (100·0%)
BMI (kg/m^2)	26·6 (4·0)	26·8 (4·1)	25·4 (2·5)
Income			
bottom quintile (≤£7,186)	414/3,408 (12·1%)	363/2,900 (12·5%)	51/508 (10·0%)
2nd quintile (>£7,186 ≤ £10,834)	406/3,408 (11·9%)	349/2,900 (12·0%)	57/508 (11·2%)
3rd quintile (>£10,834 ≤ £17,890)	809/3,408 (23·7%)	684/2,900 (23·6%)	125/508 (24·6%)
4th quintile (>£17,890 ≤ £27,705)	858/3,408 (25·2%)	739/2,900 (25·5%)	119/508 (23·4%)
highest quintile (>£27,705)	921/3,408 (27·0%)	765/2,900 (26·4%)	156/508 (30·7%)
Ethnic group			
White	3,608/3,833 (94·1%)	3,094/3,266 (94·7%)	514/567 (90·7%)
Black - caribbean	33/3,833 (0·9%)	24/3,266 (0·7%)	9/567 (1·6%)
Black - african	24/3,833 (0·6%)	20/3,266 (0·6%)	4/567 (0·7%)
Black - other black groups	8/3,833 (0·2%)	8/3,266 (0·2%)	0/567 (0·0%)
Indian	63/3,833 (1·6%)	44/3,266 (1·3%)	19/567 (3·4%)
Pakistani	33/3,833 (0·9%)	26/3,266 (0·8%)	7/567 (1·2%)
Bangladeshi	15/3,833 (0·4%)	11/3,266 (0·3%)	4/567 (0·7%)
Chinese	9/3,833 (0·2%)	9/3,266 (0·3%)	0/567 (0·0%)
None of these	40/3,833 (1·0%)	30/3,266 (0·9%)	10/567 (1·8%)
Haemoglobin (g/L)	148·3 (10·1)	148·4 (10·4)	147·6 (8·4)
Anaemia (Haemoglobin<130g/L)			
No	3,725/3,835 (97·1%)	3,163/3,268 (96·8%)	562/567 (99·1%)
Yes	110/3,835 (2·9%)	105/3,268 (3·2%)	5/567 (0·9%)
Iron deficiency (Ferritin<15ug/L)			
No	3,769/3,835 (98·3%)	3,202/3,268 (98·0%)	567/567 (100·0%)
Yes	66/3,835 (1·7%)	66/3,268 (2·0%)	0/567 (0·0%)
Inflammation (C-Reactive Protein>5mg/L)			
No	3,437/3,835 (89·6%)	2,870/3,268 (87·8%)	567/567 (100·0%)
Yes	398/3,835 (10·4%)	398/3,268 (12·2%)	0/567 (0·0%)

HSE = Health Survey for England, BMI = Body Mass Index, SD = Standard Deviation.
Data are presented as unweighted mean (SD) for continuous measures, and unweighted n/total (%) for categorical measures based on participants with non-missing haemoglobin, ferritin, and C-reactive protein.
[1] Ferritin≥15 and ≤200ug/L and C-reactive protein≤5mg/L.

Table 3.1.5 England HSE 2006: Participant characteristics (Males) (18-65 years).

	**Overall sample N=2,569**	**Excluded sample N=2,195 (85·4%)**	**Reference sample[1] N=374 (14·6%)**
Age (years)	44·6 (12·6)	45·3 (12·6)	40·8 (12·2)
Sex			
Male	2,569/2,569 (100·0%)	2,195/2,195 (100·0%)	374/374 (100·0%)
BMI (kg/m^2)	27·5 (4·4)	27·9 (4·5)	25·5 (2·6)
Income			
bottom quintile (<£10,598)	259/2,196 (11·8%)	238/1,888 (12·6%)	21/308 (6·8%)
2nd quintile (≥£10,598 < £16,852)	261/2,196 (11·9%)	232/1,888 (12·3%)	29/308 (9·4%)
3rd quintile (≥£16,852< £25,114)	432/2,196 (19·7%)	386/1,888 (20·4%)	46/308 (14·9%)
4th quintile (≥£25,114< £40,373)	604/2,196 (27·5%)	501/1,888 (26·5%)	103/308 (33·4%)
highest quintile (≥£40,373)	640/2,196 (29·1%)	531/1,888 (28·1%)	109/308 (35·4%)
Ethnic group			
White	2,378/2,568 (92·6%)	2,059/2,194 (93·8%)	319/374 (85·3%)
Mixed	21/2,568 (0·8%)	14/2,194 (0·6%)	7/374 (1·9%)
Asian or Asian British	109/2,568 (4·2%)	76/2,194 (3·5%)	33/374 (8·8%)
Black or Black British	43/2,568 (1·7%)	32/2,194 (1·5%)	11/374 (2·9%)
Chinese or other ethnic group	17/2,568 (0·7%)	13/2,194 (0·6%)	4/374 (1·1%)
Haemoglobin (g/L)	150·5 (10·0)	150·7 (10·2)	149·8 (8·4)
Anaemia (Haemoglobin<130g/L)			
No	2,522/2,569 (98·2%)	2,153/2,195 (98·1%)	369/374 (98·7%)
Yes	47/2,569 (1·8%)	42/2,195 (1·9%)	5/374 (1·3%)
Iron deficiency (Ferritin<15ug/L)			
No	2,552/2,569 (99·3%)	2,178/2,195 (99·2%)	374/374 (100·0%)
Yes	17/2,569 (0·7%)	17/2,195 (0·8%)	0/374 (0·0%)
Inflammation (C-Reactive Protein>5mg/L)			
No	2,269/2,569 (88·3%)	1,895/2,195 (86·3%)	374/374 (100·0%)
Yes	300/2,569 (11·7%)	300/2,195 (13·7%)	0/374 (0·0%)

HSE = Health Survey for England, BMI = Body Mass Index, SD = Standard Deviation.
Data are presented as unweighted mean (SD) for continuous measures, and unweighted n/total (%) for categorical measures based on participants with non-missing haemoglobin, ferritin, and C-reactive protein.
[1] Ferritin≥15 and ≤200ug/L and C-reactive protein≤5mg/L.

Table 3.1.6 England HSE 2009: Participant characteristics (Males) (18-65 years).

	**Overall sample N=804**	**Excluded sample N=686 (85·3%)**	**Reference sample[1] N=118 (14·7%)**
Age (years)	44·5 (13·0)	45·6 (12·8)	38·1 (12·6)
Sex			
Male	804/804 (100·0%)	686/686 (100·0%)	118/118 (100·0%)
BMI (kg/m^2)	27·5 (4·4)	27·9 (4·5)	24·7 (2·9)
Income			
bottom quintile (≤£10,655·74)	84/693 (12·1%)	79/584 (13·5%)	5/109 (4·6%)
2nd quintile (>£10,655·74 ≤£16,900·00)	89/693 (12·8%)	75/584 (12·8%)	14/109 (12·8%)
3rd quintile (>£16,900·00 ≤£26,787·88)	115/693 (16·6%)	92/584 (15·8%)	23/109 (21·1%)
4th quintile (>£26,787·88 ≤£41,864·41)	197/693 (28·4%)	167/584 (28·6%)	30/109 (27·5%)
highest quintile (>£41,864·41)	208/693 (30·0%)	171/584 (29·3%)	37/109 (33·9%)
Ethnic group			
White - British	699/804 (86·9%)	599/686 (87·3%)	100/118 (84·7%)
White - Irish	5/804 (0·6%)	5/686 (0·7%)	0/118 (0·0%)
Any other white background	22/804 (2·7%)	20/686 (2·9%)	2/118 (1·7%)
Mixed - White and Black Caribbean	4/804 (0·5%)	3/686 (0·4%)	1/118 (0·8%)
Mixed - White and Black African	2/804 (0·2%)	1/686 (0·1%)	1/118 (0·8%)
Mixed - White and Asian	1/804 (0·1%)	1/686 (0·1%)	0/118 (0·0%)
Any other mixed background	8/804 (1·0%)	6/686 (0·9%)	2/118 (1·7%)
Asian or Asian British - Indian	11/804 (1·4%)	11/686 (1·6%)	0/118 (0·0%)
Asian or Asian British - Pakistani	11/804 (1·4%)	8/686 (1·2%)	3/118 (2·5%)
Asian or Asian British - Bangladeshi	4/804 (0·5%)	3/686 (0·4%)	1/118 (0·8%)
Any other Asian/Asian British background	9/804 (1·1%)	7/686 (1·0%)	2/118 (1·7%)
Black or Black British - Caribbean	7/804 (0·9%)	6/686 (0·9%)	1/118 (0·8%)
Black or Black British - African	16/804 (2·0%)	12/686 (1·7%)	4/118 (3·4%)
Any other Black/Black British background	1/804 (0·1%)	0/686 (0·0%)	1/118 (0·8%)
Chinese	1/804 (0·1%)	1/686 (0·1%)	0/118 (0·0%)
Any other	3/804 (0·4%)	3/686 (0·4%)	0/118 (0·0%)
Haemoglobin (g/L)	149·9 (9·6)	150·2 (9·7)	148·3 (9·2)
Anaemia (Haemoglobin<130g/L)			
No	791/804 (98·4%)	674/686 (98·3%)	117/118 (99·2%)
Yes	13/804 (1·6%)	12/686 (1·7%)	1/118 (0·8%)
Iron deficiency (Ferritin<15ug/L)			
No	801/804 (99·6%)	683/686 (99·6%)	118/118 (100·0%)
Yes	3/804 (0·4%)	3/686 (0·4%)	0/118 (0·0%)
Inflammation (C-Reactive Protein>5mg/L)			
No	728/804 (90·5%)	610/686 (88·9%)	118/118 (100·0%)
Yes	76/804 (9·5%)	76/686 (11·1%)	0/118 (0·0%)

HSE = Health Survey for England, BMI = Body Mass Index, SD = Standard Deviation.
Data are presented as unweighted mean (SD) for continuous measures, and unweighted n/total (%) for categorical measures based on participants with non-missing haemoglobin, ferritin, and C-reactive protein.
[1] Ferritin≥15 and ≤200ug/L and C-reactive protein≤5mg/L.

Table 3.1.7 CHN CHNS 2009: Participant characteristics (Males) (18-65 years).

	**Overall sample N=3,352**	**Excluded sample N=3,107 (92·7%)**	**Reference sample[1] N=245 (7·3%)**
Age (years)	45·7 (12·1)	46·3 (11·8)	37·2 (12·9)
Sex			
Male	3,352/3,352 (100·0%)	3,107/3,107 (100·0%)	245/245 (100·0%)
BMI (kg/m^2)	23·5 (3·4)	23·5 (3·4)	22·9 (2·7)
Nationality			
Han	2,978/3,340 (89·2%)	2,748/3,095 (88·8%)	230/245 (93·9%)
Mongolian	4/3,340 (0·1%)	4/3,095 (0·1%)	0/245 (0·0%)
Hui	10/3,340 (0·3%)	9/3,095 (0·3%)	1/245 (0·4%)
Miao	91/3,340 (2·7%)	90/3,095 (2·9%)	1/245 (0·4%)
Zhuang	22/3,340 (0·7%)	20/3,095 (0·6%)	2/245 (0·8%)
Buyi	69/3,340 (2·1%)	69/3,095 (2·2%)	0/245 (0·0%)
Korean	3/3,340 (0·1%)	3/3,095 (0·1%)	0/245 (0·0%)
Man	88/3,340 (2·6%)	79/3,095 (2·6%)	9/245 (3·7%)
Dong	1/3,340 (0·0%)	1/3,095 (0·0%)	0/245 (0·0%)
Tujia	44/3,340 (1·3%)	42/3,095 (1·4%)	2/245 (0·8%)
Other	30/3,340 (0·9%)	30/3,095 (1·0%)	0/245 (0·0%)
Haemoglobin (g/L)	153·2 (17·3)	153·1 (17·5)	153·4 (15·6)
Anaemia (Haemoglobin<130g/L)			
No	3,252/3,352 (97·0%)	3,008/3,107 (96·8%)	244/245 (99·6%)
Yes	100/3,352 (3·0%)	99/3,107 (3·2%)	1/245 (0·4%)
Iron deficiency (Ferritin<15ug/L)			
No	3,294/3,352 (98·3%)	3,049/3,107 (98·1%)	245/245 (100·0%)
Yes	58/3,352 (1·7%)	58/3,107 (1·9%)	0/245 (0·0%)
Inflammation (C-Reactive Protein>5mg/L)			
No	3,058/3,352 (91·2%)	2,813/3,107 (90·5%)	245/245 (100·0%)
Yes	294/3,352 (8·8%)	294/3,107 (9·5%)	0/245 (0·0%)

CHN = China, CHNS = China Health and Nutrition Survey, BMI = Body Mass Index, SD = Standard Deviation. Data are presented as unweighted mean (SD) for continuous measures, and unweighted n/total (%) for categorical measures based on participants with non-missing haemoglobin, ferritin, and C-reactive protein.

[1] Ferritin≥15 and ≤200ug/L and C-reactive protein≤5mg/L.

Table 3.1.8 AUS NHS 2011-2012: Participant characteristics (Males) (18-65 years).

	**Overall sample N=1,912**	**Excluded sample N=1,699 (88·9%)**	**Reference sample[1] N=213 (11·1%)**
Age (years)	46·2 (12·6)	47·5 (12·2)	36·6 (11·6)
Sex			
Male	1,912/1,912 (100·0%)	1,699/1,699 (100·0%)	213/213 (100·0%)
BMI (kg/m^2)*	28·2 (5·0)	28·6 (5·0)	24·7 (2·6)
Equivalized income of household			
bottom quintile	196/1,735 (11·3%)	181/1,546 (11·7%)	15/189 (7·9%)
2nd quintile	209/1,735 (12·0%)	188/1,546 (12·2%)	21/189 (11·1%)
3rd quintile	367/1,735 (21·2%)	320/1,546 (20·7%)	47/189 (24·9%)
4th quintile	447/1,735 (25·8%)	400/1,546 (25·9%)	47/189 (24·9%)
highest quintile	516/1,735 (29·7%)	457/1,546 (29·6%)	59/189 (31·2%)
Ancestry (multiple response)			
Oceanian and Antarctican	455/1,912 (23·8%)	411/1,699 (24·2%)	44/213 (20·7%)
North-West European	1,025/1,912 (53·6%)	930/1,699 (54·7%)	95/213 (44·6%)
Southern and Eastern European	174/1,912 (9·1%)	159/1,699 (9·4%)	15/213 (7·0%)
South-East Asian	42/1,912 (2·2%)	32/1,699 (1·9%)	10/213 (4·7%)
North-East Asian	60/1,912 (3·1%)	44/1,699 (2·6%)	16/213 (7·5%)
Southern and Central Asian	78/1,912 (4·1%)	56/1,699 (3·3%)	22/213 (10·3%)
Other[†]	78/1,912 (4·1%)	67/1,699 (3·9%)	11/213 (5·2%)
Haemoglobin (g/L)	152·6 (10·5)	152·9 (10·6)	150·3 (8·9)
Anaemia (Haemoglobin<130g/L)			
No	1,884/1,912 (98·5%)	Not displayed[§]	Not displayed[§]
Yes	28/1,912 (1·5%)	Not displayed[§]	<10/213 (<4·7%)
Iron deficiency (Ferritin<15ug/L)			
No	1,895/1,912 (99·1%)	Not displayed[§]	Not displayed[§]
Yes	17/1,912 (0·9%)	Not displayed[§]	<10/213 (<4·7%)
Inflammation (C-Reactive Protein>5mg/L)			
No	1,713/1,912 (89·6%)	Not displayed[§]	Not displayed[§]
Yes	199/1,912 (10·4%)	Not displayed[§]	<10/213 (<4·7%)

AUS = Australia, NHS = National Health Survey, BMI = Body Mass Index, SD = Standard Deviation.
Data are presented as unweighted mean (SD) for continuous measures, and unweighted n/total (%) for categorical measures based on participants with non-missing haemoglobin, ferritin, and C-reactive protein.
*BMI is missing for 53 participants.
[†] Other includes North African and Middle Eastern, American, Sub-Saharan African, Not stated, inadequately described.
[§]Not displayed due to Australian Bureau of Statistics requirements for output clearance.
[1] Ferritin≥15 and ≤200ug/L and C-reactive protein≤5mg/L.

Table 3.1.9 AUS NNPAS 2011-2012: Participant characteristics (Males) (18-65 years).

	**Overall sample N=1,277**	**Excluded sample N=983 (77·0%)**	**Reference sample[1] N=294 (23·0%)**
Age (years)	45·4 (12·8)	47·6 (11·9)	37·7 (12·8)
Sex			
Male	1,277/1,277 (100·0%)	983/983 (100·0%)	294/294 (100·0%)
BMI (kg/m^2)*	27·9 (4·7)	28·6 (4·8)	25·2 (2·7)
Equivalized income of household			
bottom quintile	152/1,218 (12·5%)	124/947 (13·1%)	28/271 (10·3%)
2nd quintile	160/1,218 (13·1%)	129/947 (13·6%)	31/271 (11·4%)
3rd quintile	245/1,218 (20·1%)	184/947 (19·4%)	61/271 (22·5%)
4th quintile	326/1,218 (26·8%)	243/947 (25·7%)	83/271 (30·6%)
highest quintile	335/1,218 (27·5%)	267/947 (28·2%)	68/271 (25·1%)
Country of birth			
Australia	891/1,277 (69·8%)	699/983 (71·1%)	192/294 (65·3%)
United Kingdom	94/1,277 (7·4%)	71/983 (7·2%)	23/294 (7·8%)
Other[†]	292/1,277 (22·9%)	213/983 (21·7%)	79/294 (26·9%)
Haemoglobin (g/L)	152·3 (10·2)	152·5 (10·5)	151·3 (9·3)
Anaemia (Haemoglobin<120g/L)			
No	1,252/1,277 (98·0%)	Not displayed[§]	Not displayed[§]
Yes	25/1,277 (2·0%)	Not displayed[§]	<10/294 (<3·4%)
Iron deficiency (Ferritin<15ug/L)			
No	1,261/1,277 (98·7%)	Not displayed[§]	Not displayed[§]
Yes	16/1,277 (1·3%)	Not displayed[§]	<10/294 (<3·4%)
Inflammation (C-Reactive Protein>5mg/L)			
No	1,145/1,277 (89·7%)	Not displayed[§]	Not displayed[§]
Yes	132/1,277 (10·3%)	Not displayed[§]	<10/294 (<3·4%)

AUS = Australia, NNPAS = National Nutrition and Physical Activity Survey, BMI = Body Mass Index, SD = Standard Deviation.

Data are presented as unweighted mean (SD) for continuous measures, and unweighted n/total (%) for categorical measures based on participants with non-missing haemoglobin, ferritin, and C-reactive protein.

*BMI is missing for 43 participants.

[†] Other: New Zealand, Italy, Viet Nam, China (excl. SARs and Taiwan Province), Greece, Germany, Philippines, India, Oceania and Antarctica (excl. Australia and New Zealand), North-West Europe (excl. United Kingdom and Germany), Southern and Eastern Europe (excl. Italy and Greece), North Africa and the Middle East, South-East Asia (excl. Viet Nam and Philippines), North-East Asia (excl. China), Southern & Central Asia (excl. India), Americas, Sub-Saharan Africa.

[§] Not displayed due to Australian Bureau of Statistics requirements for output clearance.

[1] Ferritin≥15 and ≤200ug/L and C-reactive protein≤5mg/L.

3.2 Demographics Adults (18-65 years) females

Table 3.2.1 US NHANES 1999-2000: Participant characteristics (Females) (18-65 years).

	**Overall sample N=1,944**	**Excluded sample N=1,825 (93·9%)**	**Reference sample[1] N=119 (6·1%)**
Age (years)	37·8 (14·6)	38·3 (14·6)	29·4 (10·8)
Sex			
Female	1,944/1,944 (100·0%)	1,825/1,825 (100·0%)	119/119 (100·0%)
BMI (kg/m^2)	28·7 (7·2)	29·1 (7·2)	23·7 (2·9)
Poverty	416/1,674 (24·9%)	388/1,570 (24·7%)	28/104 (26·9%)
Race and Hispanic Origin			
Mexican American	622/1,944 (32·0%)	568/1,825 (31·1%)	54/119 (45·4%)
Other Hispanic	148/1,944 (7·6%)	136/1,825 (7·5%)	12/119 (10·1%)
Non-Hispanic White	724/1,944 (37·2%)	691/1,825 (37·9%)	33/119 (27·7%)
Non-Hispanic Black	382/1,944 (19·7%)	368/1,825 (20·2%)	14/119 (11·8%)
Other Race - Including Multi-Racial	68/1,944 (3·5%)	62/1,825 (3·4%)	6/119 (5·0%)
Haemoglobin (g/L)	133·1 (12·0)	132·9 (12·2)	136·9 (8·6)
Anaemia (Haemoglobin<120g/L)			
No	1,783/1,944 (91·7%)	1,669/1,825 (91·5%)	114/119 (95·8%)
Yes	161/1,944 (8·3%)	156/1,825 (8·5%)	5/119 (4·2%)
Iron deficiency (Ferritin<15ug/L)			
No	1,561/1,944 (80·3%)	1,442/1,825 (79·0%)	119/119 (100·0%)
Yes	383/1,944 (19·7%)	383/1,825 (21·0%)	0/119 (0·0%)
Inflammation (C-Reactive Protein>5mg/L)			
No	1,273/1,944 (65·5%)	1,154/1,825 (63·2%)	119/119 (100·0%)
Yes	671/1,944 (34·5%)	671/1,825 (36·8%)	0/119 (0·0%)

US = United States, NHANES = National Health and Nutrition Examination Survey, BMI = Body Mass Index, SD = Standard Deviation.
Data are presented as unweighted mean (SD) for continuous measures, and unweighted n/total (%) for categorical measures based on participants with non-missing haemoglobin, ferritin, and C-reactive protein.
[1] Ferritin≥15 and ≤150ug/L and C-reactive protein≤5mg/L.

Table 3.2.2 US NHANES 2001-2002: Participant characteristics (Females) (18-65 years).

	Overall sample N=2,160	Excluded sample N=2,052 (95·0%)	Reference sample[1] N=108 (5·0%)
Age (years)	37·5 (14·1)	37·9 (14·2)	30·0 (10·8)
Sex			
Female	2,160/2,160 (100·0%)	2,052/2,052 (100·0%)	108/108 (100·0%)
BMI (kg/m^2)	28·3 (7·0)	28·6 (7·1)	24·2 (2·8)
Poverty	430/2,026 (21·2%)	401/1,925 (20·8%)	29/101 (28·7%)
Race and Hispanic Origin			
Mexican American	526/2,160 (24·4%)	489/2,052 (23·8%)	37/108 (34·3%)
Other Hispanic	97/2,160 (4·5%)	86/2,052 (4·2%)	11/108 (10·2%)
Non-Hispanic White	1,001/2,160 (46·3%)	966/2,052 (47·1%)	35/108 (32·4%)
Non-Hispanic Black	445/2,160 (20·6%)	428/2,052 (20·9%)	17/108 (15·7%)
Other Race - Including Multi-Racial	91/2,160 (4·2%)	83/2,052 (4·0%)	8/108 (7·4%)
Haemoglobin (g/L)	132·7 (12·9)	132·5 (13·0)	136·5 (9·9)
Anaemia (Haemoglobin<120g/L)			
No	1,946/2,160 (90·1%)	1,843/2,052 (89·8%)	103/108 (95·4%)
Yes	214/2,160 (9·9%)	209/2,052 (10·2%)	5/108 (4·6%)
Iron deficiency (Ferritin<15ug/L)			
No	1,668/2,160 (77·2%)	1,560/2,052 (76·0%)	108/108 (100·0%)
Yes	492/2,160 (22·8%)	492/2,052 (24·0%)	0/108 (0·0%)
Inflammation (C-Reactive Protein>5mg/L)			
No	1,475/2,160 (68·3%)	1,367/2,052 (66·6%)	108/108 (100·0%)
Yes	685/2,160 (31·7%)	685/2,052 (33·4%)	0/108 (0·0%)

US = United States, NHANES = National Health and Nutrition Examination Survey, BMI = Body Mass Index, SD = Standard Deviation.

Data are presented as unweighted mean (SD) for continuous measures, and unweighted n/total (%) for categorical measures based on participants with non-missing haemoglobin, ferritin, and C-reactive protein.

[1] Ferritin≥15 and ≤150ug/L and C-reactive protein≤5mg/L.

Table 3.2.3 US NHANES 2003-2004: Participant characteristics (Females) (18-65 years).

	**Overall sample N=1,455**	**Excluded sample N=1,337 (91·9%)**	**Reference sample[1] N=118 (8·1%)**
Age (years)	30·9 (9·8)	31·2 (9·8)	27·5 (9·2)
Sex			
Female	1,455/1,455 (100·0%)	1,337/1,337 (100·0%)	118/118 (100·0%)
BMI (kg/m^2)	28·3 (7·4)	28·7 (7·5)	23·6 (3·0)
Poverty	403/1,381 (29·2%)	371/1,272 (29·2%)	32/109 (29·4%)
Race and Hispanic Origin			
Mexican American	336/1,455 (23·1%)	295/1,337 (22·1%)	41/118 (34·7%)
Other Hispanic	54/1,455 (3·7%)	46/1,337 (3·4%)	8/118 (6·8%)
Non-Hispanic White	648/1,455 (44·5%)	615/1,337 (46·0%)	33/118 (28·0%)
Non-Hispanic Black	360/1,455 (24·7%)	334/1,337 (25·0%)	26/118 (22·0%)
Other Race - Including Multi-Racial	57/1,455 (3·9%)	47/1,337 (3·5%)	10/118 (8·5%)
Haemoglobin (g/L)	133·2 (12·4)	132·9 (12·6)	136·4 (8·9)
Anaemia (Haemoglobin<120g/L)			
No	1,334/1,455 (91·7%)	1,218/1,337 (91·1%)	116/118 (98·3%)
Yes	121/1,455 (8·3%)	119/1,337 (8·9%)	2/118 (1·7%)
Iron deficiency (Ferritin<15ug/L)			
No	1,204/1,455 (82·7%)	1,086/1,337 (81·2%)	118/118 (100·0%)
Yes	251/1,455 (17·3%)	251/1,337 (18·8%)	0/118 (0·0%)
Inflammation (C-Reactive Protein>5mg/L)			
No	986/1,455 (67·8%)	868/1,337 (64·9%)	118/118 (100·0%)
Yes	469/1,455 (32·2%)	469/1,337 (35·1%)	0/118 (0·0%)

US = United States, NHANES = National Health and Nutrition Examination Survey, BMI = Body Mass Index, SD = Standard Deviation.

Data are presented as unweighted mean (SD) for continuous measures, and unweighted n/total (%) for categorical measures based on participants with non-missing haemoglobin, ferritin, and C-reactive protein. No ferritin was measured in participants more than 49 years.

[1] Ferritin≥15 and ≤150ug/L and C-reactive protein≤5mg/L.

Table 3.2.4 US NHANES 2005-2006: Participant characteristics (Females) (18-65 years).

	**Overall sample N=1,606**	**Excluded sample N=1,473 (91·7%)**	**Reference sample[1] N=133 (8·3%)**
Age (years)	30·7 (9·5)	31·0 (9·5)	28·1 (9·2)
Sex			
Female	1,606/1,606 (100·0%)	1,473/1,473 (100·0%)	133/133 (100·0%)
BMI (kg/m^2)	28·6 (7·4)	29·0 (7·5)	23·9 (3·2)
Poverty	392/1,539 (25·5%)	357/1,414 (25·2%)	35/125 (28·0%)
Race and Hispanic Origin			
Mexican American	416/1,606 (25·9%)	366/1,473 (24·8%)	50/133 (37·6%)
Other Hispanic	64/1,606 (4·0%)	58/1,473 (3·9%)	6/133 (4·5%)
Non-Hispanic White	638/1,606 (39·7%)	606/1,473 (41·1%)	32/133 (24·1%)
Non-Hispanic Black	395/1,606 (24·6%)	364/1,473 (24·7%)	31/133 (23·3%)
Other Race - Including Multi-Racial	93/1,606 (5·8%)	79/1,473 (5·4%)	14/133 (10·5%)
Haemoglobin (g/L)	131·3 (12·5)	130·9 (12·8)	135·5 (8·5)
Anaemia (Haemoglobin<120g/L)			
No	1,451/1,606 (90·3%)	1,320/1,473 (89·6%)	131/133 (98·5%)
Yes	155/1,606 (9·7%)	153/1,473 (10·4%)	2/133 (1·5%)
Iron deficiency (Ferritin<15ug/L)			
No	1,291/1,606 (80·4%)	1,158/1,473 (78·6%)	133/133 (100·0%)
Yes	315/1,606 (19·6%)	315/1,473 (21·4%)	0/133 (0·0%)
Inflammation (C-Reactive Protein>5mg/L)			
No	1,074/1,606 (66·9%)	941/1,473 (63·9%)	133/133 (100·0%)
Yes	532/1,606 (33·1%)	532/1,473 (36·1%)	0/133 (0·0%)

US = United States, NHANES = National Health and Nutrition Examination Survey, BMI = Body Mass Index, SD = Standard Deviation.

Data are presented as unweighted mean (SD) for continuous measures, and unweighted n/total (%) for categorical measures based on participants with non-missing haemoglobin, ferritin, and C-reactive protein. No ferritin was measured in participants more than 49 years.

[1] Ferritin≥15 and ≤150ug/L and C-reactive protein≤5mg/L.

Table 3.2.5 US NHANES 2007-2008: Participant characteristics (Females) (18-65 years).

	Overall sample N=1,434	Excluded sample N=1,297 (90·4%)	Reference sample[1] N=137 (9·6%)
Age (years)	33·8 (9·3)	34·1 (9·3)	30·8 (9·2)
Sex			
Female	1,434/1,434 (100·0%)	1,297/1,297 (100·0%)	137/137 (100·0%)
BMI (kg/m^2)	28·6 (7·6)	29·0 (7·7)	24·1 (2·9)
Poverty	364/1,328 (27·4%)	338/1,207 (28·0%)	26/121 (21·5%)
Race and Hispanic Origin			
Mexican American	304/1,434 (21·2%)	259/1,297 (20·0%)	45/137 (32·8%)
Other Hispanic	197/1,434 (13·7%)	167/1,297 (12·9%)	30/137 (21·9%)
Non-Hispanic White	582/1,434 (40·6%)	553/1,297 (42·6%)	29/137 (21·2%)
Non-Hispanic Black	289/1,434 (20·2%)	267/1,297 (20·6%)	22/137 (16·1%)
Other Race - Including Multi-Racial	62/1,434 (4·3%)	51/1,297 (3·9%)	11/137 (8·0%)
Haemoglobin (g/L)	132·3 (12·6)	132·2 (13·0)	133·2 (8·5)
Anaemia (Haemoglobin<120g/L)			
No	1,273/1,434 (88·8%)	1,140/1,297 (87·9%)	133/137 (97·1%)
Yes	161/1,434 (11·2%)	157/1,297 (12·1%)	4/137 (2·9%)
Iron deficiency (Ferritin<15ug/L)			
No	1,177/1,434 (82·1%)	1,040/1,297 (80·2%)	137/137 (100·0%)
Yes	257/1,434 (17·9%)	257/1,297 (19·8%)	0/137 (0·0%)
Inflammation (C-Reactive Protein>5mg/L)			
No	1,068/1,434 (74·5%)	931/1,297 (71·8%)	137/137 (100·0%)
Yes	366/1,434 (25·5%)	366/1,297 (28·2%)	0/137 (0·0%)

US = United States, NHANES = National Health and Nutrition Examination Survey, BMI = Body Mass Index, SD = Standard Deviation.

Data are presented as unweighted mean (SD) for continuous measures, and unweighted n/total (%) for categorical measures based on participants with non-missing haemoglobin, ferritin, and C-reactive protein. No ferritin was measured in participants more than 49 years.

[1] Ferritin≥15 and ≤150ug/L and C-reactive protein≤5mg/L.

Table 3.2.6 US NHANES 2009-2010: Participant characteristics (Females) (18-65 years).

	Overall sample N=1,703	Excluded sample N=1,536 (90·2%)	Reference sample[1] N=167 (9·8%)
Age (years)	33·5 (9·5)	33·8 (9·4)	30·7 (9·2)
Sex			
Female	1,703/1,703 (100·0%)	1,536/1,536 (100·0%)	167/167 (100·0%)
BMI (kg/m^2)	28·8 (8·0)	29·4 (8·2)	24·0 (3·0)
Poverty	475/1,557 (30·5%)	438/1,416 (30·9%)	37/141 (26·2%)
Race and Hispanic Origin			
Mexican American	346/1,703 (20·3%)	299/1,536 (19·5%)	47/167 (28·1%)
Other Hispanic	201/1,703 (11·8%)	175/1,536 (11·4%)	26/167 (15·6%)
Non-Hispanic White	738/1,703 (43·3%)	685/1,536 (44·6%)	53/167 (31·7%)
Non-Hispanic Black	303/1,703 (17·8%)	285/1,536 (18·6%)	18/167 (10·8%)
Other Race - Including Multi-Racial	115/1,703 (6·8%)	92/1,536 (6·0%)	23/167 (13·8%)
Haemoglobin (g/L)	131·9 (12·0)	131·7 (12·3)	133·8 (8·6)
Anaemia (Haemoglobin<120g/L)			
No	1,504/1,703 (88·3%)	1,346/1,536 (87·6%)	158/167 (94·6%)
Yes	199/1,703 (11·7%)	190/1,536 (12·4%)	9/167 (5·4%)
Iron deficiency (Ferritin<15ug/L)			
No	1,411/1,703 (82·9%)	1,244/1,536 (81·0%)	167/167 (100·0%)
Yes	292/1,703 (17·1%)	292/1,536 (19·0%)	0/167 (0·0%)
Inflammation (C-Reactive Protein>5mg/L)			
No	1,267/1,703 (74·4%)	1,100/1,536 (71·6%)	167/167 (100·0%)
Yes	436/1,703 (25·6%)	436/1,536 (28·4%)	0/167 (0·0%)

US = United States, NHANES = National Health and Nutrition Examination Survey, BMI = Body Mass Index, SD = Standard Deviation.

Data are presented as unweighted mean (SD) for continuous measures, and unweighted n/total (%) for categorical measures based on participants with non-missing haemoglobin, ferritin, and C-reactive protein. No ferritin was measured in participants more than 49 years.

[1] Ferritin≥15 and ≤150ug/L and C-reactive protein≤5mg/L.

Table 3.2.7 US NHANES 2015-2016: Participant characteristics (Females) (18-65 years).

	**Overall sample N=1,485**	**Excluded sample N=1,317 (88·7%)**	**Reference sample[1] N=168 (11·3%)**
Age (years)	33·5 (9·2)	33·7 (9·3)	31·7 (8·6)
Sex			
Female	1,485/1,485 (100·0%)	1,317/1,317 (100·0%)	168/168 (100·0%)
BMI (kg/m^2)	29·7 (8·1)	30·4 (8·3)	24·1 (3·2)
Poverty	331/1,360 (24·3%)	297/1,212 (24·5%)	34/148 (23·0%)
Race and Hispanic Origin			
Mexican American	289/1,485 (19·5%)	246/1,317 (18·7%)	43/168 (25·6%)
Other Hispanic	194/1,485 (13·1%)	169/1,317 (12·8%)	25/168 (14·9%)
Non-Hispanic White	407/1,485 (27·4%)	378/1,317 (28·7%)	29/168 (17·3%)
Non-Hispanic Black	339/1,485 (22·8%)	323/1,317 (24·5%)	16/168 (9·5%)
Non-Hispanic Asian	194/1,485 (13·1%)	142/1,317 (10·8%)	52/168 (31·0%)
Other Race - Including Multi-Racial	62/1,485 (4·2%)	59/1,317 (4·5%)	3/168 (1·8%)
Haemoglobin (g/L)	128·8 (13·0)	128·3 (13·3)	132·8 (8·4)
Anaemia (Haemoglobin<120g/L)			
No	1,227/1,485 (82·6%)	1,067/1,317 (81·0%)	160/168 (95·2%)
Yes	258/1,485 (17·4%)	250/1,317 (19·0%)	8/168 (4·8%)
Iron deficiency (Ferritin<15ug/L)			
No	1,198/1,485 (80·7%)	1,030/1,317 (78·2%)	168/168 (100·0%)
Yes	287/1,485 (19·3%)	287/1,317 (21·8%)	0/168 (0·0%)
Inflammation (C-Reactive Protein>5mg/L)			
No	1,083/1,485 (72·9%)	915/1,317 (69·5%)	168/168 (100·0%)
Yes	402/1,485 (27·1%)	402/1,317 (30·5%)	0/168 (0·0%)

US = United States, NHANES = National Health and Nutrition Examination Survey, BMI = Body Mass Index, SD = Standard Deviation.
Data are presented as unweighted mean (SD) for continuous measures, and unweighted n/total (%) for categorical measures based on participants with non-missing haemoglobin, ferritin, and C-reactive protein. No ferritin was measured in participants more than 49 years.
[1] Ferritin≥15 and ≤150ug/L and C-reactive protein≤5mg/L.

Table 3.2.8 US NHANES 2017-2018: Participant characteristics (Females) (18-65 years).

	Overall sample N=2,059	Excluded sample N=1,916 (93·1%)	Reference sample[1] N=143 (6·9%)
Age (years)	42·4 (14·3)	43·0 (14·3)	34·6 (12·0)
Sex			
Female	2,059/2,059 (100·0%)	1,916/1,916 (100·0%)	143/143 (100·0%)
BMI (kg/m^2)	30·3 (8·4)	30·8 (8·5)	24·0 (3·0)
Poverty	412/1,790 (23·0%)	383/1,672 (22·9%)	29/118 (24·6%)
Race and Hispanic Origin			
Mexican American	318/2,059 (15·4%)	295/1,916 (15·4%)	23/143 (16·1%)
Other Hispanic	223/2,059 (10·8%)	201/1,916 (10·5%)	22/143 (15·4%)
Non-Hispanic White	616/2,059 (29·9%)	591/1,916 (30·8%)	25/143 (17·5%)
Non-Hispanic Black	486/2,059 (23·6%)	469/1,916 (24·5%)	17/143 (11·9%)
Non-Hispanic Asian	307/2,059 (14·9%)	254/1,916 (13·3%)	53/143 (37·1%)
Other Race - Including Multi-Racial	109/2,059 (5·3%)	106/1,916 (5·5%)	3/143 (2·1%)
Haemoglobin (g/L)	132·0 (13·0)	131·8 (13·3)	134·6 (7·9)
Anaemia (Haemoglobin<120g/L)			
No	1,803/2,059 (87·6%)	1,665/1,916 (86·9%)	138/143 (96·5%)
Yes	256/2,059 (12·4%)	251/1,916 (13·1%)	5/143 (3·5%)
Iron deficiency (Ferritin<15ug/L)			
No	1,812/2,059 (88·0%)	1,669/1,916 (87·1%)	143/143 (100·0%)
Yes	247/2,059 (12·0%)	247/1,916 (12·9%)	0/143 (0·0%)
Inflammation (C-Reactive Protein>5mg/L)			
No	1,471/2,059 (71·4%)	1,328/1,916 (69·3%)	143/143 (100·0%)
Yes	588/2,059 (28·6%)	588/1,916 (30·7%)	0/143 (0·0%)

US = United States, NHANES = National Health and Nutrition Examination Survey, BMI = Body Mass Index, SD = Standard Deviation.
Data are presented as unweighted mean (SD) for continuous measures, and unweighted n/total (%) for categorical measures based on participants with non-missing haemoglobin, ferritin, and C-reactive protein.
[1] Ferritin≥15 and ≤150ug/L and C-reactive protein≤5mg/L.

Table 3.2.9 England HSE 1998: Participant characteristics (Females) (18-65 years).

	Overall sample N=4,189	Excluded sample N=3,613 (86·2%)	Reference sample[1] N=576 (13·8%)
Age (years)	42·5 (12·5)	42·8 (12·6)	40·6 (11·8)
Sex			
Female	4,189/4,189 (100·0%)	3,613/3,613 (100·0%)	576/576 (100·0%)
BMI (kg/m^2)	26·3 (5·2)	26·6 (5·4)	24·2 (2·7)
Income			
bottom quintile (≤£7,186)	604/3,699 (16·3%)	553/3,194 (17·3%)	51/505 (10·1%)
2nd quintile (>£7,186 ≤ £10,834)	492/3,699 (13·3%)	449/3,194 (14·1%)	43/505 (8·5%)
3rd quintile (>£10,834 ≤ £17,890)	905/3,699 (24·5%)	778/3,194 (24·4%)	127/505 (25·1%)
4th quintile (>£17,890 ≤ £27,705)	865/3,699 (23·4%)	733/3,194 (22·9%)	132/505 (26·1%)
highest quintile (>£27,705)	833/3,699 (22·5%)	681/3,194 (21·3%)	152/505 (30·1%)
Ethnic group			
White	3,957/4,184 (94·6%)	3,421/3,608 (94·8%)	536/576 (93·1%)
Black - Caribbean	24/4,184 (0·6%)	20/3,608 (0·6%)	4/576 (0·7%)
Black - African	35/4,184 (0·8%)	29/3,608 (0·8%)	6/576 (1·0%)
Black - other black groups	10/4,184 (0·2%)	10/3,608 (0·3%)	0/576 (0·0%)
Indian	67/4,184 (1·6%)	57/3,608 (1·6%)	10/576 (1·7%)
Pakistani	28/4,184 (0·7%)	24/3,608 (0·7%)	4/576 (0·7%)
Bangladeshi	11/4,184 (0·3%)	9/3,608 (0·2%)	2/576 (0·3%)
Chinese	8/4,184 (0·2%)	7/3,608 (0·2%)	1/576 (0·2%)
None of these	44/4,184 (1·1%)	31/3,608 (0·9%)	13/576 (2·3%)
Haemoglobin (g/L)	131·7 (10·4)	131·8 (10·6)	131·1 (8·4)
Anaemia (Haemoglobin<120g/L)			
No	3,782/4,189 (90·3%)	3,248/3,613 (89·9%)	534/576 (92·7%)
Yes	407/4,189 (9·7%)	365/3,613 (10·1%)	42/576 (7·3%)
Iron deficiency (Ferritin<15ug/L)			
No	3,673/4,189 (87·7%)	3,097/3,613 (85·7%)	576/576 (100·0%)
Yes	516/4,189 (12·3%)	516/3,613 (14·3%)	0/576 (0·0%)
Inflammation (C-Reactive Protein>5mg/L)			
No	3,472/4,189 (82·9%)	2,896/3,613 (80·2%)	576/576 (100·0%)
Yes	717/4,189 (17·1%)	717/3,613 (19·8%)	0/576 (0·0%)

HSE = Health Survey for England, BMI = Body Mass Index, SD = Standard Deviation.
Data are presented as unweighted mean (SD) for continuous measures, and unweighted n/total (%) for categorical measures based on participants with non-missing haemoglobin, ferritin, and C-reactive protein.
[1] Ferritin≥15 and ≤150ug/L and C-reactive protein≤5mg/L.

Table 3.2.10 England HSE 2006: Participant characteristics (Females) (18-65 years).

	Overall sample N=3,026	Excluded sample N=2,511 (83·0%)	Reference sample[1] N=515 (17·0%)
Age (years)	44·5 (12·4)	45·0 (12·4)	42·1 (11·8)
Sex			
Female	3,026/3,026 (100·0%)	2,511/2,511 (100·0%)	515/515 (100·0%)
BMI (kg/m^2)	26·7 (5·4)	27·2 (5·7)	24·2 (2·8)
Income			
bottom quintile (<£10,598)	380/2,591 (14·7%)	342/2,149 (15·9%)	38/442 (8·6%)
2nd quintile (≥£10,598< £16,852)	404/2,591 (15·6%)	351/2,149 (16·3%)	53/442 (12·0%)
3rd quintile (≥£16,852< £25,114)	537/2,591 (20·7%)	456/2,149 (21·2%)	81/442 (18·3%)
4th quintile (≥£25,114< £40,373)	638/2,591 (24·6%)	524/2,149 (24·4%)	114/442 (25·8%)
highest quintile (≥£40,373)	632/2,591 (24·4%)	476/2,149 (22·1%)	156/442 (35·3%)
Ethnic group			
White	2,792/3,025 (92·3%)	2,336/2,510 (93·1%)	456/515 (88·5%)
Mixed	31/3,025 (1·0%)	27/2,510 (1·1%)	4/515 (0·8%)
Asian or Asian British	123/3,025 (4·1%)	92/2,510 (3·7%)	31/515 (6·0%)
Black or Black British	52/3,025 (1·7%)	40/2,510 (1·6%)	12/515 (2·3%)
Chinese or other ethnic group	27/3,025 (0·9%)	15/2,510 (0·6%)	12/515 (2·3%)
Haemoglobin (g/L)	134·0 (10·2)	134·2 (10·6)	133·1 (8·3)
Anaemia (Haemoglobin<120g/L)			
No	2,819/3,026 (93·2%)	2,328/2,511 (92·7%)	491/515 (95·3%)
Yes	207/3,026 (6·8%)	183/2,511 (7·3%)	24/515 (4·7%)
Iron deficiency (Ferritin<15ug/L)			
No	2,762/3,026 (91·3%)	2,247/2,511 (89·5%)	515/515 (100·0%)
Yes	264/3,026 (8·7%)	264/2,511 (10·5%)	0/515 (0·0%)
Inflammation (C-Reactive Protein>5mg/L)			
No	2,513/3,026 (83·0%)	1,998/2,511 (79·6%)	515/515 (100·0%)
Yes	513/3,026 (17·0%)	513/2,511 (20·4%)	0/515 (0·0%)

HSE = Health Survey for England, BMI = Body Mass Index, SD = Standard Deviation.
Data are presented as unweighted mean (SD) for continuous measures, and unweighted n/total (%) for categorical measures based on participants with non-missing haemoglobin, ferritin, and C-reactive protein.
[1] Ferritin≥15 and ≤150ug/L and C-reactive protein≤5mg/L.

Table 3.2.11 England HSE 2009: Participant characteristics (Females) (18-65 years).

	**Overall sample N=892**	**Excluded sample N=750 (84·1%)**	**Reference sample[1] N=142 (15·9%)**
Age (years)	44·9 (12·5)	45·5 (12·5)	41·9 (11·8)
Sex			
Female	892/892 (100·0%)	750/750 (100·0%)	142/142 (100·0%)
BMI (kg/m^2)	27·3 (5·7)	27·8 (6·0)	24·4 (2·9)
Income			
bottom quintile (≤£10,655·74)	110/768 (14·3%)	101/646 (15·6%)	9/122 (7·4%)
2nd quintile (>£10,655·74 ≤£16,900·00)	135/768 (17·6%)	119/646 (18·4%)	16/122 (13·1%)
3rd quintile (>£16,900·00 ≤£26,787·88)	140/768 (18·2%)	123/646 (19·0%)	17/122 (13·9%)
4th quintile (>£26,787·88 ≤£41,864·41)	192/768 (25·0%)	155/646 (24·0%)	37/122 (30·3%)
highest quintile (>£41,864·41)	191/768 (24·9%)	148/646 (22·9%)	43/122 (35·2%)
Ethnic group			
White - British	781/891 (87·7%)	672/750 (89·6%)	109/141 (77·3%)
White - Irish	8/891 (0·9%)	5/750 (0·7%)	3/141 (2·1%)
Any other white background	36/891 (4·0%)	27/750 (3·6%)	9/141 (6·4%)
Mixed - White and Black Caribbean	1/891 (0·1%)	1/750 (0·1%)	0/141 (0·0%)
Mixed - White and Black African	2/891 (0·2%)	1/750 (0·1%)	1/141 (0·7%)
Mixed - White and Asian	2/891 (0·2%)	1/750 (0·1%)	1/141 (0·7%)
Any other mixed background	4/891 (0·4%)	4/750 (0·5%)	0/141 (0·0%)
Asian or Asian British - Indian	9/891 (1·0%)	8/750 (1·1%)	1/141 (0·7%)
Asian or Asian British - Pakistani	11/891 (1·2%)	10/750 (1·3%)	1/141 (0·7%)
Asian or Asian British - Bangladeshi	5/891 (0·6%)	4/750 (0·5%)	1/141 (0·7%)
Any other Asian/Asian British background	7/891 (0·8%)	2/750 (0·3%)	5/141 (3·5%)
Black or Black British - Caribbean	5/891 (0·6%)	3/750 (0·4%)	2/141 (1·4%)
Black or Black British - African	14/891 (1·6%)	8/750 (1·1%)	6/141 (4·3%)
Any other Black/Black British background	1/891 (0·1%)	1/750 (0·1%)	0/141 (0·0%)
Chinese	1/891 (0·1%)	1/750 (0·1%)	0/141 (0·0%)
Any other	4/891 (0·4%)	2/750 (0·3%)	2/141 (1·4%)
Haemoglobin (g/L)	132·9 (10·3)	132·8 (10·4)	133·7 (9·6)
Anaemia (Haemoglobin<120g/L)			
No	823/892 (92·3%)	683/750 (91·1%)	140/142 (98·6%)
Yes	69/892 (7·7%)	67/750 (8·9%)	2/142 (1·4%)
Iron deficiency (Ferritin<15ug/L)			
No	809/892 (90·7%)	667/750 (88·9%)	142/142 (100·0%)
Yes	83/892 (9·3%)	83/750 (11·1%)	0/142 (0·0%)
Inflammation (C-Reactive Protein>5mg/L)			
No	734/892 (82·3%)	592/750 (78·9%)	142/142 (100·0%)
Yes	158/892 (17·7%)	158/750 (21·1%)	0/142 (0·0%)

HSE = Health Survey for England, BMI = Body Mass Index, SD = Standard Deviation.
Data are presented as unweighted mean (SD) for continuous measures, and unweighted n/total (%) for categorical measures based on participants with non-missing haemoglobin, ferritin, and C-reactive protein.
[1] Ferritin≥15 and ≤150ug/L and C-reactive protein≤5mg/L.

Table 3.2.12 CHN CHNS 2009: Participant characteristics (Females) (18-65 years).

	**Overall sample N=3,820**	**Excluded sample N=3,254 (85·2%)**	**Reference sample[1] N=566 (14·8%)**
Age (years)	45·6 (11·8)	47·1 (11·4)	37·2 (10·7)
Sex			
Female	3,820/3,820 (100·0%)	3,254/3,254 (100·0%)	566/566 (100·0%)
BMI (kg/m^2)	23·4 (3·5)	23·5 (3·6)	22·9 (2·6)
Nationality			
Han	3,374/3,808 (88·6%)	2,856/3,246 (88·0%)	518/562 (92·2%)
Mongolian	4/3,808 (0·1%)	4/3,246 (0·1%)	0/562 (0·0%)
Hui	7/3,808 (0·2%)	4/3,246 (0·1%)	3/562 (0·5%)
Miao	121/3,808 (3·2%)	112/3,246 (3·5%)	9/562 (1·6%)
Zhuang	28/3,808 (0·7%)	26/3,246 (0·8%)	2/562 (0·4%)
Buyi	68/3,808 (1·8%)	68/3,246 (2·1%)	0/562 (0·0%)
Korean	3/3,808 (0·1%)	2/3,246 (0·1%)	1/562 (0·2%)
Man	117/3,808 (3·1%)	97/3,246 (3·0%)	20/562 (3·6%)
Tujia	43/3,808 (1·1%)	41/3,246 (1·3%)	2/562 (0·4%)
Other	43/3,808 (1·1%)	36/3,246 (1·1%)	7/562 (1·2%)
Haemoglobin (g/L)	132·4 (17·9)	132·1 (18·2)	134·3 (16·0)
Anaemia (Haemoglobin<120g/L)			
No	2,213/3,820 (57·9%)	1,859/3,254 (57·1%)	354/566 (62·5%)
Yes	1,607/3,820 (42·1%)	1,395/3,254 (42·9%)	212/566 (37·5%)
Iron deficiency (Ferritin<15ug/L)			
No	3,145/3,820 (82·3%)	2,579/3,254 (79·3%)	566/566 (100·0%)
Yes	675/3,820 (17·7%)	675/3,254 (20·7%)	0/566 (0·0%)
Inflammation (C-Reactive Protein>5mg/L)			
No	3,538/3,820 (92·6%)	2,972/3,254 (91·3%)	566/566 (100·0%)
Yes	282/3,820 (7·4%)	282/3,254 (8·7%)	0/566 (0·0%)

CHN = China, CHNS = China Health and Nutrition Survey, BMI = Body Mass Index, SD = Standard Deviation. Data are presented as unweighted mean (SD) for continuous measures, and unweighted n/total (%) for categorical measures based on participants with non-missing haemoglobin, ferritin, and C-reactive protein.

[1] Ferritin≥15 and ≤150ug/L and C-reactive protein≤5mg/L.

Table 3.2.13 AUS NHS 2011-2012: Participant characteristics (Females) (18-65 years).

	Overall sample N=2,480	Excluded sample N=2,198 (88·6%)	Reference sample N=282 (11·4%)
Age (years)	45·3 (12·6)	46·2 (12·5)	38·3 (11·0)
Sex			
Female	2,480/2,480 (100·0%)	2,198/2,198 (100·0%)	282/282 (100·0%)
BMI (kg/m^2)*	27·5 (6·3)	28·0 (6·5)	23·7 (2·7)
Equivalized income of household			
bottom quintile	270/2,121 (12·7%)	251/1,883 (13·3%)	19/238 (8·0%)
2nd quintile	352/2,121 (16·6%)	317/1,883 (16·8%)	35/238 (14·7%)
3rd quintile	487/2,121 (23·0%)	430/1,883 (22·8%)	57/238 (23·9%)
4th quintile	521/2,121 (24·6%)	450/1,883 (23·9%)	71/238 (29·8%)
highest quintile	491/2,121 (23·1%)	435/1,883 (23·1%)	56/238 (23·5%)
Ancestry (multiple response)			
Oceanian and Antarctican	621/2,480 (25·0%)	565/2,198 (25·7%)	56/282 (19·9%)
North-West European	1,326/2,480 (53·5%)	1,206/2,198 (54·9%)	120/282 (42·6%)
Southern and Eastern European	217/2,480 (8·8%)	185/2,198 (8·4%)	32/282 (11·3%)
South-East Asian	73/2,480 (2·9%)	57/2,198 (2·6%)	16/282 (5·7%)
North-East Asian	63/2,480 (2·5%)	43/2,198 (2·0%)	20/282 (7·1%)
Southern and Central Asian	74/2,480 (3·0%)	58/2,198 (2·6%)	16/282 (5·7%)
Other[†]	106/2,480 (4·3%)	84/2,198 (3·8%)	22/282 (7·8%)
Haemoglobin (g/L)	135·5 (9·9)	135·7 (10·1)	134·7 (8·6)
Anaemia (Haemoglobin<120g/L)			
No	2,354/2,480 (94·9%)	Not displayed[§]	Not displayed[§]
Yes	126/2,480 (5·1%)	Not displayed[§]	<10/282 (<3·5%)
Iron deficiency (Ferritin<15ug/L)			
No	2,306/2,480 (93·0%)	Not displayed[§]	Not displayed[§]
Yes	174/2,480 (7·0%)	Not displayed[§]	<10/282 (<3·5%)
Inflammation (C-Reactive Protein>5mg/L)			
No	2,010/2,480 (81·0%)	Not displayed[§]	Not displayed[§]
Yes	470/2,480 (19·0%)	Not displayed[§]	<10/282 (<3·5%)

AUS = Australia, NHS= National Health Survey, BMI = Body Mass Index, SD = Standard Deviation.
Data are presented as unweighted mean (SD) for continuous measures, and unweighted n/total (%) for categorical measures based on participants with non-missing haemoglobin, ferritin, and C-reactive protein.
*BMI is missing for 176 participants.
[†] Other includes North African and Middle Eastern, American, Sub-Saharan African, Not stated, inadequately described.
[§] Not displayed due to Australian Bureau of Statistics requirements for output clearance.
[1] Ferritin≥15 and ≤150ug/L and C-reactive protein≤5mg/L.

Table 3.2.14 AUS NNPAS 2011-2012: Participant characteristics (Females) (18-65 years).

	**Overall sample N=1,593**	**Excluded sample N=1,114 (69·9%)**	**Reference sample N=479 (30·1%)**
Age (years)	44·6 (12·7)	46·2 (12·7)	41·0 (11·9)
Sex			
Female	1,593/1,593 (100·0%)	1,114/1,114 (100·0%)	479/479 (100·0%)
BMI (kg/m^2)*	27·3 (6·2)	28·9 (6·6)	23·8 (2·9)
Equivalized income of household			
bottom quintile	245/1,504 (16·3%)	189/1,057 (17·9%)	56/447 (12·5%)
2nd quintile	252/1,504 (16·8%)	192/1,057 (18·2%)	60/447 (13·4%)
3rd quintile	307/1,504 (20·4%)	211/1,057 (20·0%)	96/447 (21·5%)
4th quintile	379/1,504 (25·2%)	247/1,057 (23·4%)	132/447 (29·5%)
highest quintile	321/1,504 (21·3%)	218/1,057 (20·6%)	103/447 (23·0%)
Country of birth			
Australia	1,140/1,593 (71·6%)	835/1,114 (75·0%)	305/479 (63·7%)
United Kingdom	109/1,593 (6·8%)	66/1,114 (5·9%)	43/479 (9·0%)
Other[†]	344/1,593 (21·6%)	213/1,114 (19·1%)	131/479 (27·3%)
Haemoglobin (g/L)	135·2 (9·6)	135·4 (10·0)	134·6 (8·4)
Anaemia (Haemoglobin<130g/L)	1,512/1,593 (94·9%)	1,052/1,114 (94·4%)	460/479 (96·0%)
No	81/1,593 (5·1%)	62/1,114 (5·6%)	19/479 (4·0%)
Yes			
Iron deficiency (Ferritin<15ug/L)	1,495/1,593 (93·8%)	Not displayed[§]	Not displayed[§]
No	98/1,593 (6·2%)	Not displayed[§]	<10/479 (<2·1%)
Yes			
Inflammation (C-Reactive Protein>5mg/L)	1,288/1,593 (80·9%)	Not displayed[§]	Not displayed[§]
No	305/1,593 (19·1%)	Not displayed[§]	<10/479 (<2·1%)

AUS = Australia, NNPAS= National Nutrition and Physical Activity Survey, BMI = Body Mass Index, SD = Standard Deviation.

Data are presented as unweighted mean (SD) for continuous measures, and unweighted n/total (%) for categorical measures based on participants with non-missing haemoglobin, ferritin, and C-reactive protein.

*BMI is missing for 95 participants.

[†] Other: New Zealand, Italy, Viet Nam, China (excl. SARs and Taiwan Province), Greece, Germany, Philippines, India, Oceania and Antarctica (excl. Australia and New Zealand), North-West Europe (excl. United Kingdom and Germany), Southern and Eastern Europe (excl. Italy and Greece), North Africa and the Middle East, South-East Asia (excl. Viet Nam and Philippines), North-East Asia (excl. China), Southern & Central Asia (excl. India), Americas, Sub-Saharan Africa.

[§] Not displayed due to Australian Bureau of Statistics requirements for output clearance.

[1] Ferritin≥15 and ≤150ug/L and C-reactive protein≤5mg/L.

3.3 Demographics Children (6-23 months)

Table 3.3.1 CAN TARGet Kids! 2010-2019: Participant characteristics (6-23 months).

	**Overall sample N=1,671**	**Excluded sample N=1,248 (74·7%)**	**Reference sample[1] N=423 (25·3%)**
Age (months)	13·5 (4·2)	13·8 (4·3)	12·9 (3·9)
Sex			
Male	906/1,671 (54·2%)	699/1,248 (56·0%)	207/423 (48·9%)
Female	765/1,671 (45·8%)	549/1,248 (44·0%)	216/423 (51·1%)
Maternal ethnicity			
European	934/1,458 (64·1%)	694/1,093 (63·5%)	240/365 (65·8%)
East Asian	83/1,458 (5·7%)	62/1,093 (5·7%)	21/365 (5·8%)
South Asian	119/1,458 (8·2%)	93/1,093 (8·5%)	26/365 (7·1%)
Southeast Asian	45/1,458 (3·1%)	37/1,093 (3·4%)	8/365 (2·2%)
Arab	30/1,458 (2·1%)	20/1,093 (1·8%)	10/365 (2·7%)
African	94/1,458 (6·4%)	75/1,093 (6·9%)	19/365 (5·2%)
Latin American	45/1,458 (3·1%)	30/1,093 (2·7%)	15/365 (4·1%)
Mixed Ethnicity	102/1,458 (7·0%)	76/1,093 (7·0%)	26/365 (7·1%)
Other	6/1,458 (0·4%)	6/1,093 (0·5%)	0/365 (0·0%)
Paternal ethnicity			
European	932/1,429 (65·2%)	691/1,070 (64·6%)	241/359 (67·1%)
East Asian	55/1,429 (3·8%)	43/1,070 (4·0%)	12/359 (3·3%)
South Asian	119/1,429 (8·3%)	92/1,070 (8·6%)	27/359 (7·5%)
Southeast Asian	35/1,429 (2·4%)	27/1,070 (2·5%)	8/359 (2·2%)
Arab	34/1,429 (2·4%)	28/1,070 (2·6%)	6/359 (1·7%)
African	117/1,429 (8·2%)	95/1,070 (8·9%)	22/359 (6·1%)
Latin American	44/1,429 (3·1%)	29/1,070 (2·7%)	15/359 (4·2%)
Mixed Ethnicity	86/1,429 (6·0%)	58/1,070 (5·4%)	28/359 (7·8%)
Other	7/1,429 (0·5%)	7/1,070 (0·7%)	0/359 (0·0%)
Haemoglobin (g/L)	117·2 (9·6)	116·9 (10·0)	117·9 (8·1)
Anaemia (Haemoglobin<110g/L)			
No	1,348/1,671 (80·7%)	991/1,248 (79·4%)	357/423 (84·4%)
Yes	323/1,671 (19·3%)	257/1,248 (20·6%)	66/423 (15·6%)
Iron deficiency (Ferritin<12ug/L)			
No	1,459/1,671 (87·3%)	1,036/1,248 (83·0%)	423/423 (100·0%)
Yes	212/1,671 (12·7%)	212/1,248 (17·0%)	0/423 (0·0%)
Inflammation (C-Reactive Protein>5mg/L)			
No	1,568/1,671 (93·8%)	1,145/1,248 (91·7%)	423/423 (100·0%)
Yes	103/1,671 (6·2%)	103/1,248 (8·3%)	0/423 (0·0%)

CAN = Canada, TARGet Kids! = The Applied Research Group for Kids, BMI = Body Mass Index, SD = Standard Deviation.
Data are presented as mean (SD) for continuous measures, and n/total (%) for categorical measures based on participants with non-missing haemoglobin, ferritin, and C-reactive protein. Body mass index not presented, z-scores were used as exclusion criteria.
[1] Ferritin≥12ug/L and C-reactive protein≤5mg/L.

Table 3.3.2 BAN BRISC 2017-2020: Participant characteristics (11 months).

	Overall sample N=1,365	Excluded sample N=1,110 (81·3%)	Reference sample[1] N=255 (18·7%)
Age (months)	11·1 (0·3)	11·1 (0·3)	11·0 (0·3)
Sex			
Female	689/1,365 (50·5%)	536/1,110 (48·3%)	153/255 (60·0%)
Male	676/1,365 (49·5%)	574/1,110 (51·7%)	102/255 (40·0%)
Wealth index			
Quintile 1 (relative poorest)	298/1,364 (21·8%)	244/1,109 (22·0%)	54/255 (21·2%)
Quintile 2	267/1,364 (19·6%)	227/1,109 (20·5%)	40/255 (15·7%)
Quintile 3 (relative middle)	264/1,364 (19·4%)	209/1,109 (18·8%)	55/255 (21·6%)
Quintile 4	274/1,364 (20·1%)	225/1,109 (20·3%)	49/255 (19·2%)
Quintile 5 (relative wealthiest)	261/1,364 (19·1%)	204/1,109 (18·4%)	57/255 (22·4%)
Country			
Bangladesh	1,365/1,365 (100·0%)	1,110/1,110 (100·0%)	255/255 (100·0%)
Haemoglobin (g/L)	115·1 (9·3)	114·8 (9·5)	116·5 (8·3)
Anaemia (Haemoglobin<110g/L)			
No	1,031/1,365 (75·5%)	826/1,110 (74·4%)	205/255 (80·4%)
Yes	334/1,365 (24·5%)	284/1,110 (25·6%)	50/255 (19·6%)
Iron deficiency (Ferritin<12ug/L)			
No	1,203/1,365 (88·1%)	948/1,110 (85·4%)	255/255 (100·0%)
Yes	162/1,365 (11·9%)	162/1,110 (14·6%)	0/255 (0·0%)
Inflammation (C-Reactive Protein>5mg/L)			
No	1,220/1,365 (89·4%)	965/1,110 (86·9%)	255/255 (100·0%)
Yes	145/1,365 (10·6%)	145/1,110 (13·1%)	0/255 (0·0%)

BAN = Bangladesh, BRISC = Benefits and Risks of Iron intervention in Children, SD = Standard Deviation. Data are presented as mean (SD) for continuous measures, and n/total (%) for categorical measures based on participants with non-missing haemoglobin, ferritin, and C-reactive protein. Body mass index not presented, z-scores were used as exclusion criteria.

[1] Ferritin≥12ug/L and C-reactive protein≤5mg/L.

Table 3.3.3 ECU ENSANUT 2011-2013: Participant characteristics (6-23 months).

	**Overall sample N=787**	**Excluded sample N=692 (87·9%)**	**Reference sample[1] N=95 (12·1%)**
Age (months)	14·3 (5·1)	14·3 (5·1)	14·1 (5·3)
Sex			
Male	395/787 (50·2%)	347/692 (50·1%)	48/95 (50·5%)
Female	392/787 (49·8%)	345/692 (49·9%)	47/95 (49·5%)
Economic quintiles			
Quintile 1 (relative poorest)	239/787 (30·4%)	215/692 (31·1%)	24/95 (25·3%)
Quintile 2	176/787 (22·4%)	148/692 (21·4%)	28/95 (29·5%)
Quintile 3 (relative middle)	157/787 (19·9%)	133/692 (19·2%)	24/95 (25·3%)
Quintile 4	126/787 (16·0%)	117/692 (16·9%)	9/95 (9·5%)
Quintile 5 (relative wealthiest)	89/787 (11·3%)	79/692 (11·4%)	10/95 (10·5%)
Ethnicity (self-reported)			
Afro-Ecuadorian	32/787 (4·1%)	28/692 (4·0%)	4/95 (4·2%)
Indigenous	78/787 (9·9%)	70/692 (10·1%)	8/95 (8·4%)
Montubio	34/787 (4·3%)	31/692 (4·5%)	3/95 (3·2%)
Rest of the Population	643/787 (81·7%)	563/692 (81·4%)	80/95 (84·2%)
Haemoglobin (g/L)	114·3 (12·7)	114·4 (13·1)	113·7 (9·1)
Anaemia (Haemoglobin<110g/L)			
No	521/787 (66·2%)	461/692 (66·6%)	60/95 (63·2%)
Yes	266/787 (33·8%)	231/692 (33·4%)	35/95 (36·8%)
Iron deficiency (Ferritin<12ug/L)			
No	648/787 (82·3%)	553/692 (79·9%)	95/95 (100·0%)
Yes	139/787 (17·7%)	139/692 (20·1%)	0/95 (0·0%)
Inflammation (C-Reactive Protein>5mg/L)			
No	682/787 (86·7%)	587/692 (84·8%)	95/95 (100·0%)
Yes	105/787 (13·3%)	105/692 (15·2%)	0/95 (0·0%)

ECU = Ecuador, ENSANUT = Encuesta National de Salud y Nutricion, SD = Standard Deviation.
Data are presented as unweighted mean (SD) for continuous measures, and unweighted n/total (%) for categorical measures based on participants with non-missing haemoglobin, ferritin, and C-reactive protein. No information on body mass index was available.
[1] Ferritin≥12ug/L and C-reactive protein≤5mg/L.

Table 3.3.4 US NHANES 2003-2018: Participant characteristics (12-23 months).

	Overall sample N=530	Excluded sample N=379 (71·5%)	Reference sample[1] N=151 (28·5%)
Age (months)	18·2 (3·2)	18·1 (3·2)	18·5 (3·3)
Sex			
Male	279/530 (52·6%)	213/379 (56·2%)	66/151 (43·7%)
Female	251/530 (47·4%)	166/379 (43·8%)	85/151 (56·3%)
Poverty	198/496 (39·9%)	147/358 (41·1%)	51/138 (37·0%)
Race and Hispanic Origin			
Mexican American	139/530 (26·2%)	100/379 (26·4%)	39/151 (25·8%)
Other Hispanic	38/530 (7·2%)	28/379 (7·4%)	10/151 (6·6%)
Non-Hispanic White	171/530 (32·3%)	126/379 (33·2%)	45/151 (29·8%)
Non-Hispanic Black	136/530 (25·7%)	93/379 (24·5%)	43/151 (28·5%)
Other Race - Including Multi-Racial	46/530 (8·7%)	32/379 (8·4%)	14/151 (9·3%)
Haemoglobin (g/L)	122·7 (8·2)	122·4 (8·4)	123·6 (7·5)
Anaemia (Haemoglobin<110g/L)			
No	500/530 (94·3%)	354/379 (93·4%)	146/151 (96·7%)
Yes	30/530 (5·7%)	25/379 (6·6%)	5/151 (3·3%)
Iron deficiency (Ferritin<12ug/L)			
No	461/530 (87·0%)	310/379 (81·8%)	151/151 (100·0%)
Yes	69/530 (13·0%)	69/379 (18·2%)	0/151 (0·0%)
Inflammation (C-Reactive Protein>5mg/L)			
No	487/530 (91·9%)	336/379 (88·7%)	151/151 (100·0%)
Yes	43/530 (8·1%)	43/379 (11·3%)	0/151 (0·0%)

US = United States, NHANES = National Health and Nutrition Examination Survey, SD = Standard Deviation. Data are presented as unweighted mean (SD) for continuous measures, and unweighted n/total (%) for categorical measures based on participants with non-missing haemoglobin, ferritin, and C-reactive protein. Data was combined from 1999-2018. No ferritin was measured in participants in survey years 1999-2000, 2001-2002, 2007-2008, 2009-2010, 2011-2012, 2013-2014. No information on body mass index was available.

[1] Ferritin≥12ug/L and C-reactive protein≤5mg/L.

3.4 Demographics Children (24-59 months)

Table 3.4.1 CAN TARGet Kids! 2010-2019: Participant characteristics (24-59 months).

	**Overall sample N=2,050**	**Excluded sample N=1,293 (63·1%)**	**Reference sample[1] N=757 (36·9%)**
Age (months)	38·0 (10·6)	37·5 (10·9)	38·8 (10·0)
Sex			
Male	1,075/2,050 (52·4%)	688/1,293 (53·2%)	387/757 (51·1%)
Female	975/2,050 (47·6%)	605/1,293 (46·8%)	370/757 (48·9%)
BMI (kg/m^2)	16·0 (1·5)	16·1 (1·7)	15·9 (1·2)
Maternal ethnicity			
European	1,110/1,802 (61·6%)	663/1,155 (57·4%)	447/647 (69·1%)
East Asian	112/1,802 (6·2%)	77/1,155 (6·7%)	35/647 (5·4%)
South Asian	197/1,802 (10·9%)	150/1,155 (13·0%)	47/647 (7·3%)
Southeast Asian	79/1,802 (4·4%)	52/1,155 (4·5%)	27/647 (4·2%)
Arab	35/1,802 (1·9%)	23/1,155 (2·0%)	12/647 (1·9%)
African	106/1,802 (5·9%)	77/1,155 (6·7%)	29/647 (4·5%)
Latin American	56/1,802 (3·1%)	40/1,155 (3·5%)	16/647 (2·5%)
Mixed Ethnicity	100/1,802 (5·5%)	69/1,155 (6·0%)	31/647 (4·8%)
Other	7/1,802 (0·4%)	4/1,155 (0·3%)	3/647 (0·5%)
Paternal ethnicity			
European	1,132/1,782 (63·5%)	678/1,142 (59·4%)	454/640 (70·9%)
East Asian	79/1,782 (4·4%)	53/1,142 (4·6%)	26/640 (4·1%)
South Asian	201/1,782 (11·3%)	153/1,142 (13·4%)	48/640 (7·5%)
Southeast Asian	57/1,782 (3·2%)	37/1,142 (3·2%)	20/640 (3·1%)
Arab	44/1,782 (2·5%)	30/1,142 (2·6%)	14/640 (2·2%)
African	131/1,782 (7·4%)	91/1,142 (8·0%)	40/640 (6·3%)
Latin American	46/1,782 (2·6%)	32/1,142 (2·8%)	14/640 (2·2%)
Mixed Ethnicity	85/1,782 (4·8%)	62/1,142 (5·4%)	23/640 (3·6%)
Other	7/1,782 (0·4%)	6/1,142 (0·5%)	1/640 (0·2%)
Haemoglobin (g/L)	120·2 (8·1)	119·7 (8·4)	121·0 (7·3)
Anaemia (Haemoglobin<110g/L)			
No	1,889/2,050 (92·1%)	1,172/1,293 (90·6%)	717/757 (94·7%)
Yes	161/2,050 (7·9%)	121/1,293 (9·4%)	40/757 (5·3%)
Iron deficiency (Ferritin<12ug/L)			
No	1,911/2,050 (93·2%)	1,154/1,293 (89·2%)	757/757 (100·0%)
Yes	139/2,050 (6·8%)	139/1,293 (10·8%)	0/757 (0·0%)
Inflammation (C-Reactive Protein>5mg/L)			
No	1,906/2,050 (93·0%)	1,149/1,293 (88·9%)	757/757 (100·0%)
Yes	144/2,050 (7·0%)	144/1,293 (11·1%)	0/757 (0·0%)

CAN = Canada, TARGet Kids! = The Applied Research Group for Kids, BMI = Body Mass Index, SD = Standard Deviation.

Data are presented as mean (SD) for continuous measures, and n/total (%) for categorical measures based on participants with non-missing haemoglobin, ferritin, and C-reactive protein.

[1] Ferritin≥12ug/L and C-reactive protein≤5mg/L.

Table 3.4.2 ECU ENSANUT 2011-2013: Participant characteristics (24-59 months).

	Overall sample N=1,260	Excluded sample N=1,080 (85·7%)	Reference sample[1] N=180 (14·3%)
Age (months)	39·6 (10·4)	39·6 (10·6)	39·6 (9·7)
Sex			
Male	644/1,260 (51·1%)	556/1,080 (51·5%)	88/180 (48·9%)
Female	616/1,260 (48·9%)	524/1,080 (48·5%)	92/180 (51·1%)
Economic quintiles			
Quintile 1 (relative poorest)	362/1,260 (28·7%)	319/1,080 (29·5%)	43/180 (23·9%)
Quintile 2	309/1,260 (24·5%)	270/1,080 (25·0%)	39/180 (21·7%)
Quintile 3 (relative middle)	263/1,260 (20·9%)	220/1,080 (20·4%)	43/180 (23·9%)
Quintile 4	192/1,260 (15·2%)	159/1,080 (14·7%)	33/180 (18·3%)
Quintile 5 (relative wealthiest)	134/1,260 (10·6%)	112/1,080 (10·4%)	22/180 (12·2%)
Ethnicity (self-reported)			
Afro-Ecuadorian	41/1,260 (3·3%)	32/1,080 (3·0%)	9/180 (5·0%)
Indigenous	124/1,260 (9·8%)	112/1,080 (10·4%)	12/180 (6·7%)
Montubio	64/1,260 (5·1%)	56/1,080 (5·2%)	8/180 (4·4%)
Rest of the Population	1,031/1,260 (81·8%)	880/1,080 (81·5%)	151/180 (83·9%)
Haemoglobin (g/L)	125·0 (11·0)	125·5 (11·3)	121·7 (8·3)
Anaemia (Haemoglobin<110g/L)			
No	1,189/1,260 (94·4%)	1,020/1,080 (94·4%)	169/180 (93·9%)
Yes	71/1,260 (5·6%)	60/1,080 (5·6%)	11/180 (6·1%)
Iron deficiency (Ferritin<12ug/L)			
No	1,210/1,260 (96·0%)	1,030/1,080 (95·4%)	180/180 (100·0%)
Yes	50/1,260 (4·0%)	50/1,080 (4·6%)	0/180 (0·0%)
Inflammation (C-Reactive Protein>5mg/L)			
No	1,112/1,260 (88·3%)	932/1,080 (86·3%)	180/180 (100·0%)
Yes	148/1,260 (11·7%)	148/1,080 (13·7%)	0/180 (0·0%)

ECU = Ecuador, ENSANUT = Encuesta National de Salud y Nutricion, SD = Standard Deviation.
Data are presented as unweighted mean (SD) for continuous measures, and unweighted n/total (%) for categorical measures based on participants with non-missing haemoglobin, ferritin, and C-reactive protein.
[1] Ferritin≥12ug/L and C-reactive protein≤5mg/L.

Table 3.4.3 US NHANES 1999-2018: Participant characteristics (24-59 months).

	Overall sample N=2,559	Excluded sample N=1,622 (63·4%)	Reference sample[1] N=937 (36·6%)
Age (months)	44·5 (9·6)	44·1 (9·7)	45·1 (9·5)
Sex			
Male	1,334/2,559 (52·1%)	869/1,622 (53·6%)	465/937 (49·6%)
Female	1,225/2,559 (47·9%)	753/1,622 (46·4%)	472/937 (50·4%)
BMI (kg/m^2)	16·4 (1·8)	16·7 (2·1)	16·0 (1·1)
Poverty	902/2,370 (38·1%)	585/1,500 (39·0%)	317/870 (36·4%)
Race and Hispanic Origin			
Mexican American	700/2,559 (27·4%)	476/1,622 (29·3%)	224/937 (23·9%)
Other Hispanic	210/2,559 (8·2%)	142/1,622 (8·8%)	68/937 (7·3%)
Non-Hispanic White	784/2,559 (30·6%)	488/1,622 (30·1%)	296/937 (31·6%)
Non-Hispanic Black	645/2,559 (25·2%)	389/1,622 (24·0%)	256/937 (27·3%)
Other Race - Including Multi-Racial	220/2,559 (8·6%)	127/1,622 (7·8%)	93/937 (9·9%)
Haemoglobin (g/L)	125·3 (8·2)	124·9 (8·5)	125·8 (7·8)
Anaemia (Haemoglobin<110g/L)			
No	2,492/2,559 (97·4%)	1,570/1,622 (96·8%)	922/937 (98·4%)
Yes	67/2,559 (2·6%)	52/1,622 (3·2%)	15/937 (1·6%)
Iron deficiency (Ferritin<12ug/L)			
No	2,365/2,559 (92·4%)	1,428/1,622 (88·0%)	937/937 (100·0%)
Yes	194/2,559 (7·6%)	194/1,622 (12·0%)	0/937 (0·0%)
Inflammation (C-Reactive Protein>5mg/L)			
No	2,390/2,559 (93·4%)	1,453/1,622 (89·6%)	937/937 (100·0%)
Yes	169/2,559 (6·6%)	169/1,622 (10·4%)	0/937 (0·0%)

US = United States, NHANES = National Health and Nutrition Examination Survey, BMI = Body Mass Index, SD = Standard Deviation.
Data are presented as unweighted mean (SD) for continuous measures, and unweighted n/total (%) for categorical measures based on participants with non-missing haemoglobin, ferritin, and C-reactive protein. Data was combined from 1999-2018. No ferritin was measured in participants less than 3 years in survey years 1999-2000, 2001-2002, 2007-2008 and 2009-2010. No ferritin was measured in participants in survey years 2011-2012 and 2013-2014.
[1] Ferritin≥12ug/L and C-reactive protein≤5mg/L.

3.5 Demographics Children (5-11 years)

Table 3.5.1 CAN TARGet Kids! 2010-2019: Participant characteristics (5-11 years).

	**Overall sample N=979**	**Excluded sample N=587 (60·0%)**	**Reference sample[1] N=392 (40·0%)**
Age (months)	6·2 (1·4)	6·3 (1·5)	6·2 (1·3)
Sex			
Male	528/979 (53·9%)	329/587 (56·0%)	199/392 (50·8%)
Female	451/979 (46·1%)	258/587 (44·0%)	193/392 (49·2%)
BMI (kg/m^2)	15·9 (2·1)	16·2 (2·4)	15·5 (1·4)
Maternal ethnicity			
European	587/923 (63·6%)	343/555 (61·8%)	244/368 (66·3%)
East Asian	81/923 (8·8%)	46/555 (8·3%)	35/368 (9·5%)
South Asian	70/923 (7·6%)	50/555 (9·0%)	20/368 (5·4%)
Southeast Asian	33/923 (3·6%)	19/555 (3·4%)	14/368 (3·8%)
Arab	16/923 (1·7%)	10/555 (1·8%)	6/368 (1·6%)
African	46/923 (5·0%)	34/555 (6·1%)	12/368 (3·3%)
Latin American	35/923 (3·8%)	24/555 (4·3%)	11/368 (3·0%)
Mixed Ethnicity	53/923 (5·7%)	28/555 (5·0%)	25/368 (6·8%)
Other	2/923 (0·2%)	1/555 (0·2%)	1/368 (0·3%)
Paternal ethnicity			
European	582/888 (65·5%)	342/535 (63·9%)	240/353 (68·0%)
East Asian	42/888 (4·7%)	25/535 (4·7%)	17/353 (4·8%)
South Asian	81/888 (9·1%)	52/535 (9·7%)	29/353 (8·2%)
Southeast Asian	20/888 (2·3%)	9/535 (1·7%)	11/353 (3·1%)
Arab	15/888 (1·7%)	9/535 (1·7%)	6/353 (1·7%)
African	61/888 (6·9%)	43/535 (8·0%)	18/353 (5·1%)
Latin American	30/888 (3·4%)	19/535 (3·6%)	11/353 (3·1%)
Mixed Ethnicity	55/888 (6·2%)	35/535 (6·5%)	20/353 (5·7%)
Other	2/888 (0·2%)	1/535 (0·2%)	1/353 (0·3%)
Haemoglobin (g/L)	124·1 (7·9)	124·2 (8·4)	123·9 (7·2)
Anaemia (Haemoglobin<115g/L)			
No	874/979 (89·3%)	514/587 (87·6%)	360/392 (91·8%)
Yes	105/979 (10·7%)	73/587 (12·4%)	32/392 (8·2%)
Iron deficiency (Ferritin<15ug/L)			
No	926/979 (94·6%)	534/587 (91·0%)	392/392 (100·0%)
Yes	53/979 (5·4%)	53/587 (9·0%)	0/392 (0·0%)
Inflammation (C-Reactive Protein>5mg/L)			
No	934/979 (95·4%)	542/587 (92·3%)	392/392 (100·0%)
Yes	45/979 (4·6%)	45/587 (7·7%)	0/392 (0·0%)

CAN = Canada, TARGet Kids! = The Applied Research Group for Kids, BMI = Body Mass Index, SD = Standard Deviation.

Data are presented as mean (SD) for continuous measures, and n/total (%) for categorical measures based on participants with non-missing haemoglobin, ferritin, and C-reactive protein.

[1] Ferritin≥15ug/L and C-reactive protein≤5mg/L.

Table 3.5.2 US NHANES 1999-2018: Participant characteristics (5-11 years).

	**Overall sample N=3,030**	**Excluded sample N=1,829 (60·4%)**	**Reference sample[1] N=1,201 (39·6%)**
Age (years)	7·7 (2·2)	7·7 (2·2)	7·7 (2·2)
Sex			
Male	1,531/3,030 (50·5%)	947/1,829 (51·8%)	584/1,201 (48·6%)
Female	1,499/3,030 (49·5%)	882/1,829 (48·2%)	617/1,201 (51·4%)
BMI (kg/m^2)	17·8 (3·7)	18·5 (4·3)	16·6 (2·1)
Poverty	1,024/2,778 (36·9%)	594/1,680 (35·4%)	430/1,098 (39·2%)
Race and Hispanic Origin			
Mexican American	973/3,030 (32·1%)	581/1,829 (31·8%)	392/1,201 (32·6%)
Other Hispanic	166/3,030 (5·5%)	111/1,829 (6·1%)	55/1,201 (4·6%)
Non-Hispanic White	818/3,030 (27·0%)	500/1,829 (27·3%)	318/1,201 (26·5%)
Non-Hispanic Black	910/3,030 (30·0%)	530/1,829 (29·0%)	380/1,201 (31·6%)
Other Race - Including Multi-Racial	163/3,030 (5·4%)	107/1,829 (5·9%)	56/1,201 (4·7%)
Haemoglobin (g/L)	130·6 (9·0)	130·4 (9·0)	130·8 (9·1)
Anaemia (Haemoglobin<115g/L)			
No	2,944/3,030 (97·2%)	1,778/1,829 (97·2%)	1,166/1,201 (97·1%)
Yes	86/3,030 (2·8%)	51/1,829 (2·8%)	35/1,201 (2·9%)
Iron deficiency (Ferritin<15ug/L)			
No	2,798/3,030 (92·3%)	1,597/1,829 (87·3%)	1,201/1,201 (100·0%)
Yes	232/3,030 (7·7%)	232/1,829 (12·7%)	0/1,201 (0·0%)
Inflammation (C-Reactive Protein>5mg/L)			
No	2,824/3,030 (93·2%)	1,623/1,829 (88·7%)	1,201/1,201 (100·0%)
Yes	206/3,030 (6·8%)	206/1,829 (11·3%)	0/1,201 (0·0%)

US = United States, NHANES = National Health and Nutrition Examination Survey, BMI = Body Mass Index, SD = Standard Deviation.

Data are presented as unweighted mean (SD) for continuous measures, and unweighted n/total (%) for categorical measures based on participants with non-missing haemoglobin, ferritin, and C-reactive protein. Data was combined from 1999-2018. No ferritin was measured in participants 6 to 11 years in survey years 2003-2004, 2005-2006, 2007-2008, 2009-2010, 2015-2016, 2017-2018. No ferritin was measured in participants in survey years 2011-2012 and 2013-2014.

[1] Ferritin≥15ug/L and C-reactive protein≤5mg/L.

Table 3.5.3 CHN CHNS 2009: Participant characteristics (5-11 years).

	**Overall sample N=404**	**Excluded sample N=155 (38·4%)**	**Reference sample[1] N=249 (61·6%)**
Age (years)	9·0 (1·5)	9·0 (1·6)	9·0 (1·5)
Sex			
Male	227/404 (56·2%)	84/155 (54·2%)	143/249 (57·4%)
Female	177/404 (43·8%)	71/155 (45·8%)	106/249 (42·6%)
BMI (kg/m^2)	16·3 (2·9)	16·7 (3·9)	16·1 (2·0)
Nationality			
Han	338/403 (83·9%)	112/155 (72·3%)	226/248 (91·1%)
Hui	1/403 (0·2%)	0/155 (0·0%)	1/248 (0·4%)
Miao	16/403 (4·0%)	8/155 (5·2%)	8/248 (3·2%)
Zhuang	5/403 (1·2%)	1/155 (0·6%)	4/248 (1·6%)
Buyi	11/403 (2·7%)	11/155 (7·1%)	0/248 (0·0%)
Korean	2/403 (0·5%)	1/155 (0·6%)	1/248 (0·4%)
Man	7/403 (1·7%)	2/155 (1·3%)	5/248 (2·0%)
Tujia	19/403 (4·7%)	19/155 (12·3%)	0/248 (0·0%)
Other	4/403 (1·0%)	1/155 (0·6%)	3/248 (1·2%)
Haemoglobin (g/L)	133·9 (15·2)	133·1 (13·8)	134·3 (16·1)
Anaemia (Haemoglobin<120g/L)			
No	380/404 (94·1%)	150/155 (96·8%)	230/249 (92·4%)
Yes	24/404 (5·9%)	5/155 (3·2%)	19/249 (7·6%)
Iron deficiency (Ferritin<15ug/L)			
No	392/404 (97·0%)	143/155 (92·3%)	249/249 (100·0%)
Yes	12/404 (3·0%)	12/155 (7·7%)	0/249 (0·0%)
Inflammation (C-Reactive Protein>5mg/L)			
No	383/404 (94·8%)	134/155 (86·5%)	249/249 (100·0%)
Yes	21/404 (5·2%)	21/155 (13·5%)	0/249 (0·0%)

CHN = China, CHNS = China Health and Nutrition Survey, BMI = Body Mass Index, SD = Standard Deviation. Data are presented as unweighted mean (SD) for continuous measures, and unweighted n/total (%) for categorical measures based on participants with non-missing haemoglobin, ferritin, and C-reactive protein.

[1] Ferritin≥15ug/L and C-reactive protein≤5mg/L.

3.6 Demographics Adolescents (12-17 years) males

Table 3.6.1 US NHANES 1999-2000: Participant characteristics (Males) (12-17 years).

	**Overall sample N=836**	**Excluded sample N=502 (60·0%)**	**Reference sample[1] N=334 (40·0%)**
Age (years)	15·0 (1·8)	14·9 (1·7)	15·1 (1·9)
Sex			
Male	836/836 (100·0%)	502/502 (100·0%)	334/334 (100·0%)
BMI (kg/m^2)	23·2 (5·8)	24·7 (6·7)	21·0 (3·1)
Poverty	269/730 (36·8%)	148/433 (34·2%)	121/297 (40·7%)
Race and Hispanic Origin			
Mexican American	371/836 (44·4%)	225/502 (44·8%)	146/334 (43·7%)
Other Hispanic	38/836 (4·5%)	22/502 (4·4%)	16/334 (4·8%)
Non-Hispanic White	159/836 (19·0%)	102/502 (20·3%)	57/334 (17·1%)
Non-Hispanic Black	243/836 (29·1%)	140/502 (27·9%)	103/334 (30·8%)
Other Race - Including Multi-Racial	25/836 (3·0%)	13/502 (2·6%)	12/334 (3·6%)
Haemoglobin (g/L)	146·5 (11·7)	145·8 (11·5)	147·6 (12·0)
Anaemia (Haemoglobin<120g/L [<15 years] or 130g/L [≥15 years])			
No	818/836 (97·8%)	490/502 (97·6%)	328/334 (98·2%)
Yes	18/836 (2·2%)	12/502 (2·4%)	6/334 (1·8%)
Iron deficiency (Ferritin<15ug/L)			
No	791/836 (94·6%)	457/502 (91·0%)	334/334 (100·0%)
Yes	45/836 (5·4%)	45/502 (9·0%)	0/334 (0·0%)
Inflammation (C-Reactive Protein>5mg/L)			
No	785/836 (93·9%)	451/502 (89·8%)	334/334 (100·0%)
Yes	51/836 (6·1%)	51/502 (10·2%)	0/334 (0·0%)

US = United States, NHANES = National Health and Nutrition Examination Survey, BMI = Body Mass Index, SD = Standard Deviation.
Data are presented as unweighted mean (SD) for continuous measures, and unweighted n/total (%) for categorical measures based on participants with non-missing haemoglobin, ferritin, and C-reactive protein.
[1] Ferritin≥15ug/L and C-reactive protein≤5mg/L.

Table 3.6.2 US NHANES 2001-2002: Participant characteristics (Males) (12-17 years).

	Overall sample N=820	Excluded sample N=529 (64·5%)	Reference sample[1] N=291 (35·5%)
Age (years)	15·1 (1·8)	15·2 (1·8)	15·1 (1·7)
Sex			
Male	820/820 (100·0%)	529/529 (100·0%)	291/291 (100·0%)
BMI (kg/m^2)	23·0 (5·5)	24·2 (6·1)	20·9 (3·2)
Poverty	206/760 (27·1%)	132/491 (26·9%)	74/269 (27·5%)
Race and Hispanic Origin			
Mexican American	246/820 (30·0%)	155/529 (29·3%)	91/291 (31·3%)
Other Hispanic	37/820 (4·5%)	22/529 (4·2%)	15/291 (5·2%)
Non-Hispanic White	243/820 (29·6%)	167/529 (31·6%)	76/291 (26·1%)
Non-Hispanic Black	258/820 (31·5%)	163/529 (30·8%)	95/291 (32·6%)
Other Race - Including Multi-Racial	36/820 (4·4%)	22/529 (4·2%)	14/291 (4·8%)
Haemoglobin (g/L)	147·1 (12·3)	146·9 (12·6)	147·7 (11·8)
Anaemia (Haemoglobin<120g/L [<15 years] or 130g/L [≥15 years])			
No	804/820 (98·0%)	515/529 (97·4%)	289/291 (99·3%)
Yes	16/820 (2·0%)	14/529 (2·6%)	2/291 (0·7%)
Iron deficiency (Ferritin<15ug/L)			
No	765/820 (93·3%)	474/529 (89·6%)	291/291 (100·0%)
Yes	55/820 (6·7%)	55/529 (10·4%)	0/291 (0·0%)
Inflammation (C-Reactive Protein>5mg/L)			
No	766/820 (93·4%)	475/529 (89·8%)	291/291 (100·0%)
Yes	54/820 (6·6%)	54/529 (10·2%)	0/291 (0·0%)

US = United States, NHANES = National Health and Nutrition Examination Survey, BMI = Body Mass Index, SD = Standard Deviation

Data are presented as unweighted mean (SD) for continuous measures, and unweighted n/total (%) for categorical measures based on participants with non-missing haemoglobin, ferritin, and C-reactive protein.

[1] Ferritin≥15ug/L and C-reactive protein≤5mg/L.

Table 3.6.3 US NHANES 2017-2018: Participant characteristics (Males) (12-17 years).

	Overall sample N=383	Excluded sample N=242 (63·2%)	Reference sample[1] N=141 (36·8%)
Age (years)	15·0 (1·8)	15·1 (1·8)	14·9 (1·8)
Sex			
Male	383/383 (100·0%)	242/242 (100·0%)	141/141 (100·0%)
BMI (kg/m^2)	23·5 (6·1)	25·3 (6·7)	20·5 (3·0)
Poverty	80/346 (23·1%)	48/219 (21·9%)	32/127 (25·2%)
Race and Hispanic Origin			
Mexican American	59/383 (15·4%)	42/242 (17·4%)	17/141 (12·1%)
Other Hispanic	29/383 (7·6%)	15/242 (6·2%)	14/141 (9·9%)
Non-Hispanic White	124/383 (32·4%)	82/242 (33·9%)	42/141 (29·8%)
Non-Hispanic Black	80/383 (20·9%)	46/242 (19·0%)	34/141 (24·1%)
Non-Hispanic Asian	51/383 (13·3%)	30/242 (12·4%)	21/141 (14·9%)
Other Race - Including Multi-Racial	40/383 (10·4%)	27/242 (11·2%)	13/141 (9·2%)
Haemoglobin (g/L)	145·2 (12·2)	145·4 (13·3)	144·8 (10·3)
Anaemia (Haemoglobin<120g/L [<15 years] or 130g/L [≥15 years])			
No	376/383 (98·2%)	236/242 (97·5%)	140/141 (99·3%)
Yes	7/383 (1·8%)	6/242 (2·5%)	1/141 (0·7%)
Iron deficiency (Ferritin<15ug/L)			
No	367/383 (95·8%)	226/242 (93·4%)	141/141 (100·0%)
Yes	16/383 (4·2%)	16/242 (6·6%)	0/141 (0·0%)
Inflammation (C-Reactive Protein>5mg/L)			
No	351/383 (91·6%)	210/242 (86·8%)	141/141 (100·0%)
Yes	32/383 (8·4%)	32/242 (13·2%)	0/141 (0·0%)

US = United States, NHANES = National Health and Nutrition Examination Survey, BMI = Body Mass Index, SD = Standard Deviation.

Data are presented as unweighted mean (SD) for continuous measures, and unweighted n/total (%) for categorical measures based on participants with non-missing haemoglobin, ferritin, and C-reactive protein.

[1] Ferritin≥15ug/L and C-reactive protein≤5mg/L.

Table 3.6.4 AUS NHS and NNPAS 2011-2012: Participant characteristics (Males) (12-17 years).

	**Overall sample N=358**	**Excluded sample N=151 (42·2%)**	**Reference sample N=207 (57·8%)**
Age (years)	14·6 (1·7)	14·6 (1·6)	14·6 (1·7)
Sex			
Male	358/358 (100·0%)	151/151 (100·0%)	207/207 (100·0%)
BMI (kg/m^2)*	21·5 (3·7)	22·3 (4·6)	20·9 (2·9)
Equivalized income of household			
bottom quintile	36/312 (11·5%)	11/133 (8·3%)	25/179 (14·0%)
2nd quintile	63/312 (20·2%)	28/133 (21·1%)	35/179 (19·6%)
3rd quintile	84/312 (26·9%)	40/133 (30·1%)	44/179 (24·6%)
4th quintile	86/312 (27·6%)	36/133 (27·1%)	50/179 (27·9%)
highest quintile	43/312 (13·8%)	18/133 (13·5%)	25/179 (14·0%)
Haemoglobin (g/L)	147·7 (11·1)	147·5 (10·2)	147·8 (11·7)
Anaemia (Haemoglobin<120g/L [<15 years] or 130g/L [≥15 years])			
No	Not displayed†	Not displayed†	Not displayed†
Yes	<10/358 (<2·8%)	Not displayed†	Not displayed†
Iron deficiency (Ferritin<15ug/L)			
No	Not displayed†	Not displayed†	Not displayed†
Yes	<10/358 (<2·8%)	Not displayed†	Not displayed†
Inflammation (C-Reactive Protein>5mg/L)			
No	333/358 (93·0%)	Not displayed†	Not displayed†
Yes	25/358 (7·0%)	Not displayed†	<10/207 (<4·8%)

AUS = Australia, NHS = National Health Survey, NNPAS = National Nutrition and Physical Activity Survey, BMI = Body Mass Index, SD = Standard Deviation.

Data are presented as unweighted mean (SD) for continuous measures, and unweighted n/total (%) for categorical measures based on participants with non-missing haemoglobin, ferritin, and C-reactive protein. Data was combined from NHS and NNPAS 2011-2012.

*BMI is missing for 21 participants.

† Not presented due to Australian Bureau of Statistics requirements for output clearance.

[1] Ferritin≥15ug/L and C-reactive protein≤5mg/L.

Table 3.6.5 CHN CHNS 2009: Participant characteristics (Males) (12-17 years).

	Overall sample N=223	Excluded sample N=93 (41·7%)	Reference sample[1] N=130 (58·3%)
Age (years)	14·1 (1·7)	14·1 (1·6)	14·1 (1·7)
Sex			
Male	223/223 (100·0%)	93/93 (100·0%)	130/130 (100·0%)
BMI (kg/m^2)	19·0 (3·5)	19·0 (4·5)	19·1 (2·6)
Nationality			
Han	192/223 (86·1%)	70/93 (75·3%)	122/130 (93·8%)
Vaguer	1/223 (0·4%)	1/93 (1·1%)	0/130 (0·0%)
Miao	4/223 (1·8%)	4/93 (4·3%)	0/130 (0·0%)
Zhuang	3/223 (1·3%)	1/93 (1·1%)	2/130 (1·5%)
Buyi	8/223 (3·6%)	8/93 (8·6%)	0/130 (0·0%)
Man	4/223 (1·8%)	1/93 (1·1%)	3/130 (2·3%)
Tujia	8/223 (3·6%)	7/93 (7·5%)	1/130 (0·8%)
Other	3/223 (1·3%)	1/93 (1·1%)	2/130 (1·5%)
Haemoglobin (g/L)	146·3 (15·0)	145·5 (16·8)	146·8 (13·7)
Anaemia (Haemoglobin<120g/L [<15 years] or 130g/L [>=15 years])			
No	211/223 (94·6%)	86/93 (92·5%)	125/130 (96·2%)
Yes	12/223 (5·4%)	7/93 (7·5%)	5/130 (3·8%)
Iron deficiency (Ferritin<15ug/L)			
No	213/223 (95·5%)	83/93 (89·2%)	130/130 (100·0%)
Yes	10/223 (4·5%)	10/93 (10·8%)	0/130 (0·0%)
Inflammation (C-Reactive Protein>5mg/L)			
No	208/223 (93·3%)	78/93 (83·9%)	130/130 (100·0%)
Yes	15/223 (6·7%)	15/93 (16·1%)	0/130 (0·0%)

CHN = China, CHNS = China Health and Nutrition Survey, BMI = Body Mass Index, SD = Standard Deviation.
Data are presented as unweighted mean (SD) for continuous measures, and unweighted n/total (%) for categorical measures based on participants with non-missing haemoglobin, ferritin, and C-reactive protein.
[1] Ferritin≥15ug/L and C-reactive protein≤5mg/L.

3.7 Demographics Adolescents (12-17 years) females

Table 3.7.1 US NHANES 1999-2000: Participant characteristics (Females) (12-17 years).

	**Overall sample N=793**	**Excluded sample N=560 (70·6%)**	**Reference sample[1] N=233 (29·4%)**
Age (years)	14·9 (1·7)	15·0 (1·8)	14·6 (1·7)
Sex			
Female	793/793 (100·0%)	560/560 (100·0%)	233/233 (100·0%)
BMI (kg/m^2)	23·9 (5·7)	24·9 (6·2)	21·4 (2·9)
Poverty	255/701 (36·4%)	181/497 (36·4%)	74/204 (36·3%)
Race and Hispanic Origin			
Mexican American	332/793 (41·9%)	241/560 (43·0%)	91/233 (39·1%)
Other Hispanic	36/793 (4·5%)	27/560 (4·8%)	9/233 (3·9%)
Non-Hispanic White	160/793 (20·2%)	112/560 (20·0%)	48/233 (20·6%)
Non-Hispanic Black	231/793 (29·1%)	157/560 (28·0%)	74/233 (31·8%)
Other Race - Including Multi-Racial	34/793 (4·3%)	23/560 (4·1%)	11/233 (4·7%)
Haemoglobin (g/L)	133·3 (9·5)	132·9 (10·0)	134·4 (8·3)
Anaemia (Haemoglobin<120g/L)			
No	736/793 (92·8%)	518/560 (92·5%)	218/233 (93·6%)
Yes	57/793 (7·2%)	42/560 (7·5%)	15/233 (6·4%)
Iron deficiency (Ferritin<15ug/L)			
No	632/793 (79·7%)	399/560 (71·3%)	233/233 (100·0%)
Yes	161/793 (20·3%)	161/560 (28·7%)	0/233 (0·0%)
Inflammation (C-Reactive Protein>5mg/L)			
No	717/793 (90·4%)	484/560 (86·4%)	233/233 (100·0%)
Yes	76/793 (9·6%)	76/560 (13·6%)	0/233 (0·0%)

US = United States, NHANES = National Health and Nutrition Examination Survey, BMI = Body Mass Index, SD = Standard Deviation.

Data are presented as unweighted mean (SD) for continuous measures, and unweighted n/total (%) for categorical measures based on participants with non-missing haemoglobin, ferritin, and C-reactive protein.

[1] Ferritin≥15ug/L and C-reactive protein≤5mg/L.

Table 3.7.2 US NHANES 2001-2002: Participant characteristics (Females) (12-17 years).

	**Overall sample N=879**	**Excluded sample N=634 (72·1%)**	**Reference sample[1] N=245 (27·9%)**
Age (years)	15·0 (1·8)	15·1 (1·8)	14·6 (1·7)
Sex			
Female	879/879 (100·0%)	634/634 (100·0%)	245/245 (100·0%)
BMI (kg/m^2)	23·3 (5·6)	24·2 (6·1)	21·0 (3·0)
Poverty	227/841 (27·0%)	166/609 (27·3%)	61/232 (26·3%)
Race and Hispanic Origin			
Mexican American	287/879 (32·7%)	206/634 (32·5%)	81/245 (33·1%)
Other Hispanic	44/879 (5·0%)	29/634 (4·6%)	15/245 (6·1%)
Non-Hispanic White	262/879 (29·8%)	185/634 (29·2%)	77/245 (31·4%)
Non-Hispanic Black	260/879 (29·6%)	197/634 (31·1%)	63/245 (25·7%)
Other Race - Including Multi-Racial	26/879 (3·0%)	17/634 (2·7%)	9/245 (3·7%)
Haemoglobin (g/L)	133·1 (10·8)	132·3 (11·1)	135·3 (9·7)
Anaemia (Haemoglobin<120g/L)			
No	801/879 (91·1%)	567/634 (89·4%)	234/245 (95·5%)
Yes	78/879 (8·9%)	67/634 (10·6%)	11/245 (4·5%)
Iron deficiency (Ferritin<15ug/L)			
No	664/879 (75·5%)	419/634 (66·1%)	245/245 (100·0%)
Yes	215/879 (24·5%)	215/634 (33·9%)	0/245 (0·0%)
Inflammation (C-Reactive Protein>5mg/L)			
No	822/879 (93·5%)	577/634 (91·0%)	245/245 (100·0%)
Yes	57/879 (6·5%)	57/634 (9·0%)	0/245 (0·0%)

US = United States, NHANES = National Health and Nutrition Examination Survey, BMI = Body Mass Index, SD = Standard Deviation.

Data are presented as unweighted mean (SD) for continuous measures, and unweighted n/total (%) for categorical measures based on participants with non-missing haemoglobin, ferritin, and C-reactive protein.

[1] Ferritin≥15ug/L and C-reactive protein≤5mg/L.

Table 3.7.3 US NHANES 2003-2004: Participant characteristics (Females) (12-17 years).

	Overall sample N=730	Excluded sample N=487 (66·7%)	Reference sample[1] N=243 (33·3%)
Age (years)	15·0 (1·8)	15·2 (1·8)	14·6 (1·7)
Sex			
Female	730/730 (100·0%)	487/487 (100·0%)	243/243 (100·0%)
BMI (kg/m^2)	23·6 (5·9)	24·7 (6·6)	21·5 (2·9)
Poverty	229/706 (32·4%)	153/470 (32·6%)	76/236 (32·2%)
Race and Hispanic Origin			
Mexican American	227/730 (31·1%)	141/487 (29·0%)	86/243 (35·4%)
Other Hispanic	21/730 (2·9%)	18/487 (3·7%)	3/243 (1·2%)
Non-Hispanic White	191/730 (26·2%)	138/487 (28·3%)	53/243 (21·8%)
Non-Hispanic Black	258/730 (35·3%)	170/487 (34·9%)	88/243 (36·2%)
Other Race - Including Multi-Racial	33/730 (4·5%)	20/487 (4·1%)	13/243 (5·3%)
Haemoglobin (g/L)	133·7 (10·3)	132·9 (10·6)	135·3 (9·5)
Anaemia (Haemoglobin<120g/L)			
No	669/730 (91·6%)	439/487 (90·1%)	230/243 (94·7%)
Yes	61/730 (8·4%)	48/487 (9·9%)	13/243 (5·3%)
Iron deficiency (Ferritin<15ug/L)			
No	602/730 (82·5%)	359/487 (73·7%)	243/243 (100·0%)
Yes	128/730 (17·5%)	128/487 (26·3%)	0/243 (0·0%)
Inflammation (C-Reactive Protein>5mg/L)			
No	660/730 (90·4%)	417/487 (85·6%)	243/243 (100·0%)
Yes	70/730 (9·6%)	70/487 (14·4%)	0/243 (0·0%)

US = United States, NHANES = National Health and Nutrition Examination Survey, BMI = Body Mass Index, SD = Standard Deviation.
Data are presented as unweighted mean (SD) for continuous measures, and unweighted n/total (%) for categorical measures based on participants with non-missing haemoglobin, ferritin, and C-reactive protein.
[1] Ferritin≥15ug/L and C-reactive protein≤5mg/L.

Table 3.7.4 US NHANES 2005-2006: Participant characteristics (Females) (12-17 years).

	**Overall sample N=732**	**Excluded sample N=512 (69·9%)**	**Reference sample[1] N=220 (30.1%)**
Age (years)	15·1 (1·7)	15·2 (1·7)	14·7 (1·7)
Sex			
Female	732/732 (100·0%)	512/512 (100·0%)	220/220 (100·0%)
BMI (kg/m^2)	24·2 (6·2)	25·4 (6·9)	21·5 (2·9)
Poverty	199/707 (28·1%)	145/496 (29·2%)	54/211 (25·6%)
Race and Hispanic Origin			
Mexican American	255/732 (34·8%)	176/512 (34·4%)	79/220 (35·9%)
Other Hispanic	19/732 (2·6%)	14/512 (2·7%)	5/220 (2·3%)
Non-Hispanic White	192/732 (26·2%)	143/512 (27·9%)	49/220 (22·3%)
Non-Hispanic Black	233/732 (31·8%)	157/512 (30·7%)	76/220 (34·5%)
Other Race - Including Multi-Racial	33/732 (4·5%)	22/512 (4·3%)	11/220 (5·0%)
Haemoglobin (g/L)	133·5 (10·2)	132·9 (10·5)	134·7 (9·3)
Anaemia (Haemoglobin<120g/L)			
No	675/732 (92·2%)	466/512 (91·0%)	209/220 (95·0%)
Yes	57/732 (7·8%)	46/512 (9·0%)	11/220 (5·0%)
Iron deficiency (Ferritin<15ug/L)			
No	620/732 (84·7%)	400/512 (78·1%)	220/220 (100·0%)
Yes	112/732 (15·3%)	112/512 (21·9%)	0/220 (0·0%)
Inflammation (C-Reactive Protein>5mg/L)			
No	663/732 (90·6%)	443/512 (86·5%)	220/220 (100·0%)
Yes	69/732 (9·4%)	69/512 (13·5%)	0/220 (0·0%)

US = United States, NHANES = National Health and Nutrition Examination Survey, BMI = Body Mass Index, SD = Standard Deviation.

Data are presented as unweighted mean (SD) for continuous measures, and unweighted n/total (%) for categorical measures based on participants with non-missing haemoglobin, ferritin, and C-reactive protein.

[1] Ferritin≥15ug/L and C-reactive protein≤5mg/L.

Table 3.7.5 US NHANES 2007-2008: Participant characteristics (Females) (12-17 years).

	**Overall sample N=383**	**Excluded sample N=264 (68·9%)**	**Reference sample[1] N=119 (31·1%)**
Age (years)	15·0 (1·7)	15·2 (1·7)	14·7 (1·6)
Sex			
Female	383/383 (100·0%)	264/264 (100·0%)	119/119 (100·0%)
BMI (kg/m^2)	24·1 (5·9)	25·3 (6·4)	21·4 (3·1)
Poverty	107/359 (29·8%)	72/248 (29·0%)	35/111 (31·5%)
Race and Hispanic Origin			
Mexican American	92/383 (24·0%)	67/264 (25·4%)	25/119 (21·0%)
Other Hispanic	52/383 (13·6%)	38/264 (14·4%)	14/119 (11·8%)
Non-Hispanic White	122/383 (31·9%)	86/264 (32·6%)	36/119 (30·3%)
Non-Hispanic Black	103/383 (26·9%)	64/264 (24·2%)	39/119 (32·8%)
Other Race - Including Multi-Racial	14/383 (3·7%)	9/264 (3·4%)	5/119 (4·2%)
Haemoglobin (g/L)	131·8 (11·3)	131·0 (11·4)	133·4 (10·9)
Anaemia (Haemoglobin<120g/L)			
No	346/383 (90·3%)	232/264 (87·9%)	114/119 (95·8%)
Yes	37/383 (9·7%)	32/264 (12·1%)	5/119 (4·2%)
Iron deficiency (Ferritin<15ug/L)			
No	323/383 (84·3%)	204/264 (77·3%)	119/119 (100·0%)
Yes	60/383 (15·7%)	60/264 (22·7%)	0/119 (0·0%)
Inflammation (C-Reactive Protein>5mg/L)			
No	350/383 (91·4%)	231/264 (87·5%)	119/119 (100·0%)
Yes	33/383 (8·6%)	33/264 (12·5%)	0/119 (0·0%)

US = United States, NHANES = National Health and Nutrition Examination Survey, BMI = Body Mass Index, SD = Standard Deviation.

Data are presented as unweighted mean (SD) for continuous measures, and unweighted n/total (%) for categorical measures based on participants with non-missing haemoglobin, ferritin, and C-reactive protein.

[1] Ferritin≥15ug/L and C-reactive protein≤5mg/L.

Table 3.7.5 US NHANES 2009-2010: Participant characteristics (Females) (12-17 years).

	Overall sample N=422	Excluded sample N=275 (65·2%)	Reference sample[1] N=147 (34·8%)
Age (years)	15·1 (1·8)	15·2 (1·7)	14·8 (1·8)
Sex			
Female	422/422 (100·0%)	275/275 (100·0%)	147/147 (100·0%)
BMI (kg/m^2)	23·9 (5·4)	25·2 (6·0)	21·3 (2·9)
Poverty	102/382 (26·7%)	76/255 (29·8%)	26/127 (20·5%)
Race and Hispanic Origin			
Mexican American	122/422 (28·9%)	74/275 (26·9%)	48/147 (32·7%)
Other Hispanic	51/422 (12·1%)	34/275 (12·4%)	17/147 (11·6%)
Non-Hispanic White	129/422 (30·6%)	81/275 (29·5%)	48/147 (32·7%)
Non-Hispanic Black	89/422 (21·1%)	67/275 (24·4%)	22/147 (15·0%)
Other Race - Including Multi-Racial	31/422 (7·3%)	19/275 (6·9%)	12/147 (8·2%)
Haemoglobin (g/L)	122/422 (28·9%)	74/275 (26·9%)	48/147 (32·7%)
Anaemia (Haemoglobin<120g/L)			
No	378/422 (89·6%)	239/275 (86·9%)	139/147 (94·6%)
Yes	44/422 (10·4%)	36/275 (13·1%)	8/147 (5·4%)
Iron deficiency (Ferritin<15ug/L)			
No	337/422 (79·9%)	190/275 (69·1%)	147/147 (100·0%)
Yes	85/422 (20·1%)	85/275 (30·9%)	0/147 (0·0%)
Inflammation (C-Reactive Protein>5mg/L)			
No	403/422 (95·5%)	256/275 (93·1%)	147/147 (100·0%)
Yes	19/422 (4·5%)	19/275 (6·9%)	0/147 (0·0%)

US = United States, NHANES = National Health and Nutrition Examination Survey, BMI = Body Mass Index, SD = Standard Deviation.

Data are presented as unweighted mean (SD) for continuous measures, and unweighted n/total (%) for categorical measures based on participants with non-missing haemoglobin, ferritin, and C-reactive protein.

[1] Ferritin≥15ug/L and C-reactive protein≤5mg/L.

Table 3.7.6 US NHANES 2015-2016: Participant characteristics (Females) (12-17 years).

	**Overall sample N=420**	**Excluded sample N=266 (63.3%)**	**Reference sample[1] N=154 (36·7%)**
Age (years)	15·1 (1·7)	15·3 (1·8)	14·8 (1·7)
Sex			
Female	420/420 (100·0%)	266/266 (100·0%)	154/154 (100·0%)
BMI (kg/m^2)	24·6 (5·9)	26·0 (6·6)	22·1 (3·3)
Poverty	102/387 (26·4%)	64/244 (26·2%)	38/143 (26·6%)
Race and Hispanic Origin			
Mexican American	105/420 (25·0%)	68/266 (25·6%)	37/154 (24·0%)
Other Hispanic	53/420 (12·6%)	34/266 (12·8%)	19/154 (12·3%)
Non-Hispanic White	113/420 (26·9%)	71/266 (26·7%)	42/154 (27·3%)
Non-Hispanic Black	85/420 (20·2%)	58/266 (21·8%)	27/154 (17·5%)
Non-Hispanic Asian	39/420 (9·3%)	19/266 (7·1%)	20/154 (13·0%)
Other Race - Including Multi-Racial	25/420 (6·0%)	16/266 (6·0%)	9/154 (5·8%)
Haemoglobin (g/L)	130·4 (11·1)	128·0 (11·7)	134·7 (8·5)
Anaemia (Haemoglobin<120g/L)			
No	368/420 (87·6%)	220/266 (82·7%)	148/154 (96·1%)
Yes	52/420 (12·4%)	46/266 (17·3%)	6/154 (3·9%)
Iron deficiency (Ferritin<15ug/L)			
No	332/420 (79·0%)	178/266 (66·9%)	154/154 (100·0%)
Yes	88/420 (21·0%)	88/266 (33·1%)	0/154 (0·0%)
Inflammation (C-Reactive Protein>5mg/L)			
No	394/420 (93·8%)	240/266 (90·2%)	154/154 (100·0%)
Yes	26/420 (6·2%)	26/266 (9·8%)	0/154 (0·0%)

US = United States, NHANES = National Health and Nutrition Examination Survey, BMI = Body Mass Index, SD = Standard Deviation.

Data are presented as unweighted mean (SD) for continuous measures, and unweighted n/total (%) for categorical measures based on participants with non-missing haemoglobin, ferritin, and C-reactive protein.

[1] Ferritin≥15ug/L and C-reactive protein≤5mg/L.

Table 3.7.7 US NHANES 2017-2018: Participant characteristics (Females) (12-17 years).

	**Overall sample N=366**	**Excluded sample N=257 (70.2%)**	**Reference sample[1] N=109 (29·8%)**
Age (years)	15·1 (1·8)	15·2 (1·7)	14·8 (1·9)
Sex			
Female	366/366 (100·0%)	257/257 (100·0%)	109/109 (100·0%)
BMI (kg/m^2)	24·8 (6·4)	26·1 (6·9)	21·7 (3·4)
Poverty	81/324 (25·0%)	64/229 (27·9%)	17/95 (17·9%)
Race and Hispanic Origin			
Mexican American	80/366 (21·9%)	52/257 (20·2%)	28/109 (25·7%)
Other Hispanic	26/366 (7·1%)	17/257 (6·6%)	9/109 (8·3%)
Non-Hispanic White	104/366 (28·4%)	78/257 (30·4%)	26/109 (23·9%)
Non-Hispanic Black	86/366 (23·5%)	63/257 (24·5%)	23/109 (21·1%)
Non-Hispanic Asian	43/366 (11·7%)	27/257 (10·5%)	16/109 (14·7%)
Other Race - Including Multi-Racial	27/366 (7·4%)	20/257 (7·8%)	7/109 (6·4%)
Haemoglobin (g/L)	130·2 (11·2)	128·5 (12·1)	134·2 (7·4)
Anaemia (Haemoglobin<120g/L)			
No	315/366 (86·1%)	209/257 (81·3%)	106/109 (97·2%)
Yes	51/366 (13·9%)	48/257 (18·7%)	3/109 (2·8%)
Iron deficiency (Ferritin<15ug/L)			
No	287/366 (78·4%)	178/257 (69·3%)	109/109 (100·0%)
Yes	79/366 (21·6%)	79/257 (30·7%)	0/109 (0·0%)
Inflammation (C-Reactive Protein>5mg/L)			
No	339/366 (92·6%)	230/257 (89·5%)	109/109 (100·0%)
Yes	27/366 (7·4%)	27/257 (10·5%)	0/109 (0·0%)

US = United States, NHANES = National Health and Nutrition Examination Survey, BMI = Body Mass Index, SD = Standard Deviation.
Data are presented as unweighted mean (SD) for continuous measures, and unweighted n/total (%) for categorical measures based on participants with non-missing haemoglobin, ferritin, and C-reactive protein.
[1] Ferritin≥15ug/L and C-reactive protein≤5mg/L.

Table 3.7.8 AUS NHS and NNPAS 2011-2012: Participant characteristics (Females) (12-17 years).

	**Overall sample N=376**	**Excluded sample N=203 (54·0%)**	**Reference sample[1] N=173 (46·0%)**
Age (years)	14·7 (1·7)	15·0 (1·7)	14·4 (1·6)
Sex			
Female	376/376 (100·0%)	203/203 (100·0%)	173/173 (100·0%)
BMI (kg/m^2)*	22·3 (4·4)	22·9 (5·2)	21·6 (3·1)
Equivalized income of household			
bottom quintile	43/320 (13·4%)	21/169 (12·4%)	22/151 (14·6%)
2nd quintile	67/320 (20·9%)	38/169 (22·5%)	29/151 (19·2%)
3rd quintile	82/320 (25·6%)	47/169 (27·8%)	35/151 (23·2%)
4th quintile	81/320 (25·3%)	39/169 (23·1%)	42/151 (27·8%)
highest quintile	47/320 (14·7%)	24/169 (14·2%)	23/151 (15·2%)
Haemoglobin (g/L)	134·9 (8·7)	133·7 (9·2)	136·3 (7·9)
Anaemia (Haemoglobin<120g/L)			
No	360/376 (95·7%)	Not displayed†	Not displayed†
Yes	16/376 (4·3%)	Not displayed†	<10/173 (<5·8%)
Iron deficiency (Ferritin<15ug/L)			
No	329/376 (87·5%)	Not displayed†	Not displayed†
Yes	47/376 (12·5%)	Not displayed†	<10/173 (<5·8%)
Inflammation (C-Reactive Protein>5mg/L)			
No	358/376 (95·2%)	Not displayed†	Not displayed†
Yes	18/376 (4·8%)	Not displayed†	<10/173 (<5·8%)

AUS = Australia, NHS = National Health Survey, NNPAS = National Nutrition and Physical Activity Survey, BMI = Body Mass Index, SD = Standard Deviation.

Data are presented as unweighted mean (SD) for continuous measures, and unweighted n/total (%) for categorical measures based on participants with non-missing haemoglobin, ferritin, and C-reactive protein. Data was combined from NHS and NNPAS 2011-2012.

* BMI is missing for 25 participants.

† Not presented due to Australian Bureau of Statistics requirements for output clearance.

[1] Ferritin≥15ug/L and C-reactive protein≤5mg/L.

Table 3.7.9 CHN CHNS 2009: Participant characteristics (Females) (12-17 years).

	Overall sample N=187	Excluded sample N=85 (45·5%)	Reference sample[1] N=102 (54·5%)
Age (years)	13·9 (1·5)	13·8 (1·4)	14·0 (1·6)
Sex			
Female	187/187 (100·0%)	85/85 (100·0%)	102/102 (100·0%)
BMI (kg/m^2)	19·0 (2·8)	18·7 (2·9)	19·1 (2·8)
Nationality			
Han	154/186 (82·8%)	62/85 (72·9%)	92/101 (91·1%)
Hui	1/186 (0·5%)	0/85 (0·0%)	1/101 (1·0%)
Miao	4/186 (2·2%)	3/85 (3·5%)	1/101 (1·0%)
Zhuang	4/186 (2·2%)	1/85 (1·2%)	3/101 (3·0%)
Buyi	12/186 (6·5%)	12/85 (14·1%)	0/101 (0·0%)
Man	6/186 (3·2%)	2/85 (2·4%)	4/101 (4·0%)
Tujia	4/186 (2·2%)	4/85 (4·7%)	0/101 (0·0%)
Other	1/186 (0·5%)	1/85 (1·2%)	0/101 (0·0%)
Haemoglobin (g/L)	134·7 (15·9)	132·4 (16·3)	136·7 (15·3)
Anaemia (Haemoglobin<120g/L)			
No	172/187 (92·0%)	76/85 (89·4%)	96/102 (94·1%)
Yes	15/187 (8·0%)	9/85 (10·6%)	6/102 (5·9%)
Iron deficiency (Ferritin<15ug/L)			
No	157/187 (84·0%)	55/85 (64·7%)	102/102 (100·0%)
Yes	30/187 (16·0%)	30/85 (35·3%)	0/102 (0·0%)
Inflammation (C-Reactive Protein>5mg/L)			
No	179/187 (95·7%)	77/85 (90·6%)	102/102 (100·0%)
Yes	8/187 (4·3%)	8/85 (9·4%)	0/102 (0·0%)

CHN = China, CHNS = China Health and Nutrition Survey, BMI = Body Mass Index, SD = Standard Deviation. Data are presented as unweighted mean (SD) for continuous measures, and unweighted n/total (%) for categorical measures based on participants with non-missing haemoglobin, ferritin, and C-reactive protein.

[1] Ferritin≥15ug/L and C-reactive protein≤5mg/L.

3.8 Demographics Pregnant women

Table 3.8.1 NL Generation R: Participant characteristics (Trimester 1) (18-45 years).

	**Overall sample N=5,054**	**Excluded sample N=4,279 (84·7%)**	**Reference sample [1] N=775 (15·3%)**
Age (years)	29·8 (4·9)	29·6 (5·0)	30·8 (4·5)
BMI* (kg/m^2)	24·5 (4·4)	24·8 (4·6)	22·8 (2·4)
Nationality			
Dutch	2,469/4,844 (51·0%)	1,992/4,070 (48·9%)	477/774 (61·6%)
Other†	2,375/4,844 (49·0%)	2,078/4,070 (51·1%)	297/774 (38·4%)
Haemoglobin (g/L)	123·4 (9·2)	123·4 (9·3)	123·8 (8·7)
Anaemia (Haemoglobin<110g/L)	372/5,054 (7·4%)	327/4,279 (7·6%)	45/775 (5·8%)
Iron deficiency (Ferritin<15ug/L)*	366/4,785 (7·6%)	366/4,010 (9·1%)	0/775 (0%)
Inflammation (C-Reactive Protein>5mg/L)*	2,136/4,737 (45·1%)	2,136/3,962 (53·9%)	0/775 (0%)

NL = The Netherlands, BMI = Body Mass Index, SD = Standard Deviation.
Data are presented as mean (SD) for continuous measures, and n/total (%) for categorical measures based on participants with non-missing haemoglobin in first trimester of pregnancy.
* BMI, iron deficiency, and inflammation in the first trimester of pregnancy.
† Other includes Indonesian, Cape Verde, Moroccan, Dutch Antilles, Surinamese, Turkish, African, American, western, American, non-western, Asian, western, Asian, non-western, European, Oceania.
[1] Ferritin≥15 and ≤150ug/L and C-reactive protein≤5mg/L (both first trimester of pregnancy). If a measurement of one or more of the healthy criteria is missing, then the participant is excluded from the reference sample.

Table 3.8.2 NL Generation R: Participant characteristics (Trimester 2) (18-45 years).

	**Overall sample**<br>**N=2,194**	**Excluded sample**<br>**N=2,083 (94·9%)**	**Reference sample** [1]<br>**N=111 (5·1%)**
Age (years)	29·9 (5·6)	29·8 (5·6)	31·5 (4·6)
BMI* (kg/m^2)	24·7 (4·5)	25·0 (4·7)	22·7 (2·4)
Nationality			
Dutch	886/2,029 (43·7%)	813/1,918 (42·4%)	73/111 (65·8%)
Other†	1,143/2,029 (56·3%)	1,105/1,918 (57·6%)	38/111 (34·5%)
Haemoglobin (g/L)	116·3 (9·7)	116·2 (9·7)	118·4 (7·6)
Anaemia (Haemoglobin<110g/L)	549/2,149 (25·0%)	535/2,083 (25·7%)	14/111 (12·6%)
Iron deficiency (Ferritin<15ug/L)*	45/658 (6·8%)	45/547 (8·2%)	0/111 (0%)
Inflammation (C-Reactive Protein>5mg/L)*	276/649 (42·5%)	276/538 (51·3%)	0/111 (0%)

NL = The Netherlands, BMI = Body Mass Index, SD = Standard Deviation.
Data are presented as mean (SD) for continuous measures, and n/total (%) for categorical measures based on participants with non-missing haemoglobin in second trimester of pregnancy.
* BMI, iron deficiency, and inflammation in the first trimester of pregnancy. Approx. 75% of the participants in their second trimester of pregnancy did not have a first trimester blood sample.
† Other includes Indonesian, Cape Verde, Moroccan, Dutch Antilles, Surinamese, Turkish, African, American-western, American-non-western, Asian-western, Asian- non-western, European, Oceania.
[1] Ferritin≥15 and ≤150ug/L and C-reactive protein≤5mg/L (both first trimester for pregnancy). If a measurement of one or more of the healthy criteria is missing, then the participant is excluded from the reference sample.

Table 3.8.3 NL Generation R: Participant characteristics (Trimester 3) (18-45 years).

There were 239 participants in the overall sample. No participant characteristics presented because of the reference sample size (N=1).

4. Detailed Individual Exclusions

4.1 Detailed Individual Exclusions Adults (18-65 years)

Figure 4.1.1 Summary of exclusions of special interest from overall sample in males (18-65 years).

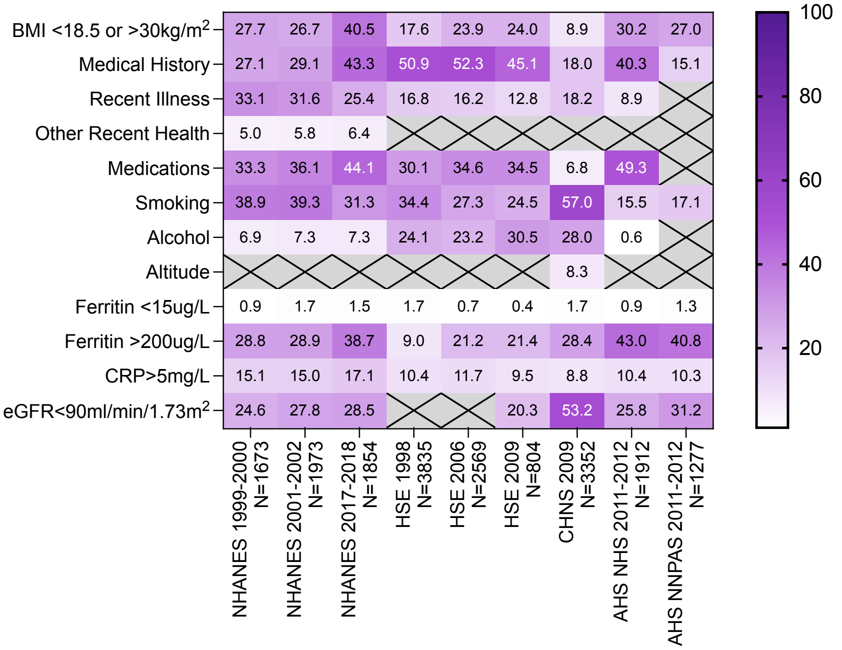

This represents the percentage of the total data set in each survey excluded by individual or grouped criteria.
Data sources consisted of NHANES, HSE, CHNS, and AHS. BMI=Body Mass Index; CRP=C-Reactive Protein; eGFR=estimated Glomerular Filtration Rate.

Figure 4.1.2 Summary of exclusions of special interest from overall sample in non-pregnant females (18-65 years).

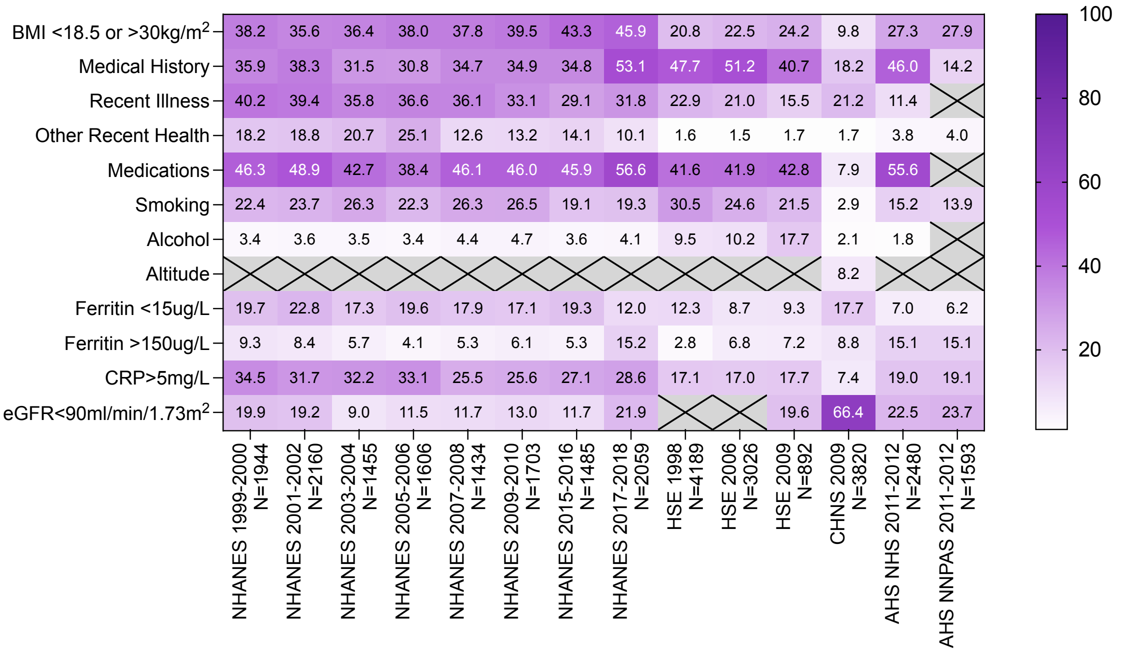

This represents the percentage of the total data set in each survey excluded by individual or grouped criteria.
Data sources consisted of NHANES, HSE, CHNS, and AHS. BMI=Body Mass Index; CRP=C-Reactive Protein; eGFR=estimated Glomerular Filtration Rate.

Table 4.1.1 US NHANES 1999-2000: Participant characteristics of violations (18-65 years).

	Overall sample	
	**Male N=1,673**	**Female N=1,944**
Reference sample[1]		
No	1,491/1,673 (89·1%)	1,825/1,944 (93·9%)
Yes	182/1,673 (10·9%)	119/1,944 (6·1%)
VIOLATION: BMI<18.5 or >30 kg/m^2		
No	1,197/1,673 (71·5%)	1,195/1,944 (61·5%)
Yes	463/1,673 (27·7%)	742/1,944 (38·2%)
No data	13/1,673 (0·8%)	7/1,944 (0·4%)
VIOLATION: Doctor told you have diabetes		
No	1,563/1,673 (93·4%)	1,840/1,944 (94·7%)
Yes	108/1,673 (6·5%)	104/1,944 (5·3%)
No data	2/1,673 (0·1%)	0/1,944 (0·0%)
VIOLATION: Ever told you had weak/failing kidneys?		
No	1,390/1,673 (83·1%)	1,642/1,944 (84·5%)
Yes	33/1,673 (2·0%)	45/1,944 (2·3%)
No data	5/1,673 (0·3%)	0/1,944 (0·0%)
Missing	245/1,673 (14·6%)	257/1,944 (13·2%)
VIOLATION: Still have asthma		
No	12/1,673 (0·7%)	15/1,944 (0·8%)
Yes	22/1,673 (1·3%)	24/1,944 (1·2%)
Missing	1,639/1,673 (98·0%)	1,905/1,944 (98·0%)
VIOLATION: Taking treatment for anaemia/past 3 months		
No	1,662/1,673 (99·3%)	1,829/1,944 (94·1%)
Yes	11/1,673 (0·7%)	114/1,944 (5·9%)
No data	0/1,673 (0·0%)	1/1,944 (0·1%)
VIOLATION: Doctor ever said you had arthritis		
No	1,230/1,673 (73·5%)	1,375/1,944 (70·7%)
Yes	197/1,673 (11·8%)	311/1,944 (16·0%)
No data	1/1,673 (0·1%)	1/1,944 (0·1%)
Missing	245/1,673 (14·6%)	257/1,944 (13·2%)
VIOLATION: Ever told had congestive heart failure		
No	1,401/1,673 (83·7%)	1,665/1,944 (85·6%)
Yes	25/1,673 (1·5%)	20/1,944 (1·0%)
No data	2/1,673 (0·1%)	2/1,944 (0·1%)
Missing	245/1,673 (14·6%)	257/1,944 (13·2%)
VIOLATION: Ever told you had coronary heart disease		
No	1,384/1,673 (82·7%)	1,662/1,944 (85·5%)
Yes	44/1,673 (2·6%)	20/1,944 (1·0%)
No data	0/1,673 (0·0%)	5/1,944 (0·3%)
Missing	245/1,673 (14·6%)	257/1,944 (13·2%)
VIOLATION: Ever told you had angina/angina pectoris		
No	1,395/1,673 (83·4%)	1,652/1,944 (85·0%)
Yes	31/1,673 (1·9%)	26/1,944 (1·3%)
No data	2/1,673 (0·1%)	9/1,944 (0·5%)
Missing	245/1,673 (14·6%)	257/1,944 (13·2%)
VIOLATION: Ever told you had heart attack		
No	1,370/1,673 (81·9%)	1,664/1,944 (85·6%)
Yes	57/1,673 (3·4%)	22/1,944 (1·1%)
No data	1/1,673 (0·1%)	1/1,944 (0·1%)
Missing	245/1,673 (14·6%)	257/1,944 (13·2%)
VIOLATION: Ever told you had a stroke		
No	1,401/1,673 (83·7%)	1,666/1,944 (85·7%)
Yes	27/1,673 (1·6%)	21/1,944 (1·1%)
Missing	245/1,673 (14·6%)	257/1,944 (13·2%)
VIOLATION: Do you still have thyroid problem		
No	4/1,673 (0·2%)	48/1,944 (2·5%)
Yes	18/1,673 (1·1%)	72/1,944 (3·7%)
No data	0/1,673 (0·0%)	7/1,944 (0·4%)
Missing	1,651/1,673 (98·7%)	1,817/1,944 (93·5%)
VIOLATION: Ever told you had emphysema		
No	1,416/1,673 (84·6%)	1,676/1,944 (86·2%)
Yes	10/1,673 (0·6%)	11/1,944 (0·6%)
No data	2/1,673 (0·1%)	0/1,944 (0·0%)
Missing	245/1,673 (14·6%)	257/1,944 (13·2%)
VIOLATION: Ever told you had chronic bronchitis		
No	1,378/1,673 (82·4%)	1,552/1,944 (79·8%)

	Overall sample	
	Male N=1,673	Female N=1,944
Yes	48/1,673 (2·9%)	135/1,944 (6·9%)
No data	2/1,673 (0·1%)	0/1,944 (0·0%)
Missing	245/1,673 (14·6%)	257/1,944 (13·2%)
VIOLATION: Ever told you had any liver condition		
No	1,368/1,673 (81·8%)	1,642/1,944 (84·5%)
Yes	58/1,673 (3·5%)	41/1,944 (2·1%)
No data	2/1,673 (0·1%)	4/1,944 (0·2%)
Missing	245/1,673 (14·6%)	257/1,944 (13·2%)
VIOLATION: Ever told you had cancer or malignancy		
No	1,387/1,673 (82·9%)	1,611/1,944 (82·9%)
Yes	40/1,673 (2·4%)	75/1,944 (3·9%)
No data	1/1,673 (0·1%)	1/1,944 (0·1%)
Missing	245/1,673 (14·6%)	257/1,944 (13·2%)
VIOLATION: Overnight hospital patient in last year		
No	1,542/1,673 (92·2%)	1,685/1,944 (86·7%)
Yes	128/1,673 (7·7%)	256/1,944 (13·2%)
No data	3/1,673 (0·2%)	3/1,944 (0·2%)
VIOLATION: SP have head cold or chest cold?		
No	1,264/1,673 (75·6%)	1,398/1,944 (71·9%)
Yes	333/1,673 (19·9%)	438/1,944 (22·5%)
No data	76/1,673 (4·5%)	108/1,944 (5·6%)
VIOLATION: SP have stomach or intestinal illness?		
No	1,462/1,673 (87·4%)	1,643/1,944 (84·5%)
Yes	133/1,673 (7·9%)	191/1,944 (9·8%)
No data	78/1,673 (4·7%)	110/1,944 (5·7%)
VIOLATION: SP have flu, pneumonia, ear infection?		
No	1,497/1,673 (89·5%)	1,730/1,944 (89·0%)
Yes	97/1,673 (5·8%)	103/1,944 (5·3%)
No data	79/1,673 (4·7%)	111/1,944 (5·7%)
VIOLATION: SP donated blood in past 12 months		
No	1,507/1,673 (90·1%)	1,726/1,944 (88·8%)
Yes	84/1,673 (5·0%)	106/1,944 (5·5%)
No data	82/1,673 (4·9%)	112/1,944 (5·8%)
VIOLATION: Are you pregnant now?		
No	0/1,673 (0·0%)	489/1,944 (25·2%)
Yes	0/1,673 (0·0%)	227/1,944 (11·7%)
No data	0/1,673 (0·0%)	610/1,944 (31·4%)
Missing	1,673/1,673 (100·0%)	618/1,944 (31·8%)
VIOLATION: Pregnancy status at exam		
No	0/1,673 (0·0%)	1,379/1,944 (70·9%)
Yes	0/1,673 (0·0%)	265/1,944 (13·6%)
No data	0/1,673 (0·0%)	78/1,944 (4·0%)
Missing	1,673/1,673 (100·0%)	222/1,944 (11·4%)
VIOLATION: Urine Pregnancy Result		
No	0/1,673 (0·0%)	1,441/1,944 (74·1%)
Yes	0/1,673 (0·0%)	263/1,944 (13·5%)
No data	0/1,673 (0·0%)	18/1,944 (0·9%)
Missing	1,673/1,673 (100·0%)	222/1,944 (11·4%)
VIOLATION: Taken prescription medicine, past month		
No	1,113/1,673 (66·5%)	1,041/1,944 (53·5%)
Yes	557/1,673 (33·3%)	901/1,944 (46·3%)
No data	3/1,673 (0·2%)	2/1,944 (0·1%)
VIOLATION: Do you now smoke cigarettes?		
No	390/1,673 (23·3%)	308/1,944 (15·8%)
Yes	420/1,673 (25·1%)	325/1,944 (16·7%)
Missing	863/1,673 (51·6%)	1,311/1,944 (67·4%)
VIOLATION: Smoked tobacco last 5 days?		
No	986/1,673 (58·9%)	1,420/1,944 (73·0%)
Yes	603/1,673 (36·0%)	409/1,944 (21·0%)
No data	84/1,673 (5·0%)	115/1,944 (5·9%)
VIOLATION: Do you now smoke a pipe?		
No	120/1,673 (7·2%)	3/1,944 (0·2%)
Yes	4/1,673 (0·2%)	0/1,944 (0·0%)
Missing	1,549/1,673 (92·6%)	1,941/1,944 (99·8%)
VIOLATION: Do you now smoke a cigar?		
No	82/1,673 (4·9%)	4/1,944 (0·2%)
Yes	64/1,673 (3·8%)	6/1,944 (0·3%)

	Overall sample	
	**Male** N=1,673	**Female** N=1,944
No data	1/1,673 (0·1%)	0/1,944 (0·0%)
Missing	1,526/1,673 (91·2%)	1,934/1,944 (99·5%)
VIOLATION: Do you now use snuff?		
No	48/1,673 (2·9%)	3/1,944 (0·2%)
Yes	44/1,673 (2·6%)	3/1,944 (0·2%)
Missing	1,581/1,673 (94·5%)	1,938/1,944 (99·7%)
VIOLATION: Do you now chew tobacco?		
No	63/1,673 (3·8%)	2/1,944 (0·1%)
Yes	14/1,673 (0·8%)	1/1,944 (0·1%)
Missing	1,596/1,673 (95·4%)	1,941/1,944 (99·8%)
VIOLATION: Excessive alcohol intake		
No	307/1,673 (18·4%)	590/1,944 (30·3%)
Yes	116/1,673 (6·9%)	67/1,944 (3·4%)
No data	64/1,673 (3·8%)	105/1,944 (5·4%)
Missing	1,186/1,673 (70·9%)	1,182/1,944 (60·8%)
VIOLATION: Ferritin<15ug/L or Ferritin>150/200ug/L (F/M)		
No	1,176/1,673 (70·3%)	1,380/1,944 (71·0%)
Yes	497/1,673 (29·7%)	564/1,944 (29·0%)
VIOLATION: C-Reactive Protein>5mg/L		
No	1,421/1,673 (84·9%)	1,273/1,944 (65·5%)
Yes	252/1,673 (15·1%)	671/1,944 (34·5%)
VIOLATION: eGFR<90 ml/min/1.73m^2		
No	1,252/1,673 (74·8%)	1,549/1,944 (79·7%)
Yes	411/1,673 (24·6%)	387/1,944 (19·9%)
No data	10/1,673 (0·6%)	8/1,944 (0·4%)

US = United States, NHANES = National Health and Nutrition Examination Survey, BMI = Body Mass Index, SP = Survey Participant, F = Female, M = Male.

Data are presented as unweighted n/total (%) based on participants with non-missing haemoglobin, ferritin, and C-reactive protein.

No data: No answer/refused, Don't know; Missing: Not applicable, Not asked. If a measurement of one or more of the healthy criteria is missing, then the participant is included in the reference sample.

[1] Males: ferritin≥15 and ≤200ug/L and C-reactive protein≤5mg/L; females: ferritin≥15 and ≤150ug/L and C-reactive protein≤5mg/L.

Table 4.1.2 US NHANES 2001-2002: Participant characteristics of violations (18-65 years).

	Overall sample	
	Male N=1,973	Female N=2,160
Reference sample[1]		
No	1,733/1,973 (87·8%)	2,052/2,160 (95·0%)
Yes	240/1,973 (12·2%)	108/2,160 (5·0%)
VIOLATION: BMI<18.5 or >30 kg/m^2		
No	1,390/1,973 (70·5%)	1,327/2,160 (61·4%)
Yes	526/1,973 (26·7%)	768/2,160 (35·6%)
No data	57/1,973 (2·9%)	65/2,160 (3·0%)
VIOLATION: Doctor told you have diabetes		
No	1,840/1,973 (93·3%)	2,031/2,160 (94·0%)
Yes	132/1,973 (6·7%)	129/2,160 (6·0%)
No data	1/1,973 (0·1%)	0/2,160 (0·0%)
VIOLATION: Ever told you had weak/failing kidneys?		
No	1,659/1,973 (84·1%)	1,881/2,160 (87·1%)
Yes	33/1,973 (1·7%)	31/2,160 (1·4%)
No data	2/1,973 (0·1%)	4/2,160 (0·2%)
Missing	279/1,973 (14·1%)	244/2,160 (11·3%)
VIOLATION: Still have asthma		
No	83/1,973 (4·2%)	97/2,160 (4·5%)
Yes	104/1,973 (5·3%)	169/2,160 (7·8%)
No data	4/1,973 (0·2%)	4/2,160 (0·2%)
Missing	1,782/1,973 (90·3%)	1,890/2,160 (87·5%)
VIOLATION: Taking treatment for anaemia/past 3 months		
No	1,958/1,973 (99·2%)	2,040/2,160 (94·4%)
Yes	15/1,973 (0·8%)	119/2,160 (5·5%)
No data	0/1,973 (0·0%)	1/2,160 (0·0%)
VIOLATION: Doctor ever said you had arthritis		
No	1,445/1,973 (73·2%)	1,570/2,160 (72·7%)
Yes	248/1,973 (12·6%)	345/2,160 (16·0%)
No data	1/1,973 (0·1%)	1/2,160 (0·0%)
Missing	279/1,973 (14·1%)	244/2,160 (11·3%)
VIOLATION: Ever told had congestive heart failure		
No	1,659/1,973 (84·1%)	1,896/2,160 (87·8%)
Yes	32/1,973 (1·6%)	19/2,160 (0·9%)
No data	3/1,973 (0·2%)	1/2,160 (0·0%)
Missing	279/1,973 (14·1%)	244/2,160 (11·3%)
VIOLATION: Ever told you had coronary heart disease		
No	1,638/1,973 (83·0%)	1,890/2,160 (87·5%)
Yes	52/1,973 (2·6%)	26/2,160 (1·2%)
No data	4/1,973 (0·2%)	0/2,160 (0·0%)
Missing	279/1,973 (14·1%)	244/2,160 (11·3%)
VIOLATION: Ever told you had angina/angina pectoris		
No	1,657/1,973 (84·0%)	1,877/2,160 (86·9%)
Yes	34/1,973 (1·7%)	35/2,160 (1·6%)
No data	3/1,973 (0·2%)	4/2,160 (0·2%)
Missing	279/1,973 (14·1%)	244/2,160 (11·3%)
VIOLATION: Ever told you had heart attack		
No	1,645/1,973 (83·4%)	1,892/2,160 (87·6%)
Yes	48/1,973 (2·4%)	24/2,160 (1·1%)
No data	1/1,973 (0·1%)	0/2,160 (0·0%)
Missing	279/1,973 (14·1%)	244/2,160 (11·3%)
VIOLATION: Ever told you had a stroke		
No	1,672/1,973 (84·7%)	1,886/2,160 (87·3%)
Yes	21/1,973 (1·1%)	29/2,160 (1·3%)
No data	1/1,973 (0·1%)	1/2,160 (0·0%)
Missing	279/1,973 (14·1%)	244/2,160 (11·3%)
VIOLATION: Do you still have thyroid problem		
No	10/1,973 (0·5%)	56/2,160 (2·6%)
Yes	21/1,973 (1·1%)	104/2,160 (4·8%)
No data	2/1,973 (0·1%)	6/2,160 (0·3%)
Missing	1,940/1,973 (98·3%)	1,994/2,160 (92·3%)
VIOLATION: Ever told you had emphysema		
No	1,677/1,973 (85·0%)	1,904/2,160 (88·1%)
Yes	17/1,973 (0·9%)	12/2,160 (0·6%)
Missing	279/1,973 (14·1%)	244/2,160 (11·3%)
VIOLATION: Ever told you had chronic bronchitis		

	Overall sample	
	**Male N=1,973**	**Female N=2,160**
No	1,626/1,973 (82·4%)	1,788/2,160 (82·8%)
Yes	62/1,973 (3·1%)	122/2,160 (5·6%)
No data	6/1,973 (0·3%)	6/2,160 (0·3%)
Missing	279/1,973 (14·1%)	244/2,160 (11·3%)
VIOLATION: Ever told you had any liver condition		
No	1,621/1,973 (82·2%)	1,876/2,160 (86·9%)
Yes	70/1,973 (3·5%)	39/2,160 (1·8%)
No data	3/1,973 (0·2%)	1/2,160 (0·0%)
Missing	279/1,973 (14·1%)	244/2,160 (11·3%)
VIOLATION: Ever told you had cancer or malignancy		
No	1,631/1,973 (82·7%)	1,802/2,160 (83·4%)
Yes	63/1,973 (3·2%)	110/2,160 (5·1%)
No data	0/1,973 (0·0%)	4/2,160 (0·2%)
Missing	279/1,973 (14·1%)	244/2,160 (11·3%)
VIOLATION: Overnight hospital patient in last year		
No	1,839/1,973 (93·2%)	1,846/2,160 (85·5%)
Yes	134/1,973 (6·8%)	314/2,160 (14·5%)
VIOLATION: SP have head cold or chest cold?		
No	1,464/1,973 (74·2%)	1,510/2,160 (69·9%)
Yes	401/1,973 (20·3%)	466/2,160 (21·6%)
No data	108/1,973 (5·5%)	184/2,160 (8·5%)
VIOLATION: SP have stomach or intestinal illness?		
No	1,704/1,973 (86·4%)	1,773/2,160 (82·1%)
Yes	161/1,973 (8·2%)	203/2,160 (9·4%)
No data	108/1,973 (5·5%)	184/2,160 (8·5%)
VIOLATION: SP have flu, pneumonia, ear infection?		
No	1,791/1,973 (90·8%)	1,853/2,160 (85·8%)
Yes	73/1,973 (3·7%)	121/2,160 (5·6%)
No data	109/1,973 (5·5%)	186/2,160 (8·6%)
VIOLATION: SP donated blood in past 12 months		
No	1,750/1,973 (88·7%)	1,865/2,160 (86·3%)
Yes	114/1,973 (5·8%)	111/2,160 (5·1%)
No data	109/1,973 (5·5%)	184/2,160 (8·5%)
VIOLATION: Are you pregnant now?		
No	0/1,973 (0·0%)	625/2,160 (28·9%)
Yes	0/1,973 (0·0%)	243/2,160 (11·3%)
No data	0/1,973 (0·0%)	1,118/2,160 (51·8%)
Missing	1,973/1,973 (100·0%)	174/2,160 (8·1%)
VIOLATION: Pregnancy status at exam		
No	0/1,973 (0·0%)	1,556/2,160 (72·0%)
Yes	0/1,973 (0·0%)	305/2,160 (14·1%)
No data	0/1,973 (0·0%)	84/2,160 (3·9%)
Missing	1,973/1,973 (100·0%)	215/2,160 (10·0%)
VIOLATION: Urine Pregnancy Result		
No	0/1,973 (0·0%)	1,621/2,160 (75·0%)
Yes	0/1,973 (0·0%)	301/2,160 (13·9%)
No data	0/1,973 (0·0%)	23/2,160 (1·1%)
Missing	1,973/1,973 (100·0%)	215/2,160 (10·0%)
VIOLATION: Taken prescription medicine, past month		
No	1,260/1,973 (63·9%)	1,103/2,160 (51·1%)
Yes	713/1,973 (36·1%)	1,057/2,160 (48·9%)
VIOLATION: Do you now smoke cigarettes?		
No	447/1,973 (22·7%)	343/2,160 (15·9%)
Yes	539/1,973 (27·3%)	415/2,160 (19·2%)
Missing	987/1,973 (50·0%)	1,402/2,160 (64·9%)
VIOLATION: Smoked tobacco last 5 days?		
No	1,160/1,973 (58·8%)	1,511/2,160 (70·0%)
Yes	703/1,973 (35·6%)	461/2,160 (21·3%)
No data	110/1,973 (5·6%)	188/2,160 (8·7%)
VIOLATION: Do you now smoke a pipe?		
No	122/1,973 (6·2%)	6/2,160 (0·3%)
Yes	16/1,973 (0·8%)	1/2,160 (0·0%)
Missing	1,835/1,973 (93·0%)	2,153/2,160 (99·7%)
VIOLATION: Do you now smoke cigars?		
No	87/1,973 (4·4%)	9/2,160 (0·4%)
Yes	67/1,973 (3·4%)	4/2,160 (0·2%)
Missing	1,819/1,973 (92·2%)	2,147/2,160 (99·4%)

	Overall sample	
	**Male N=1,973**	**Female N=2,160**
VIOLATION: Do you now use snuff?		
No	75/1,973 (3·8%)	2/2,160 (0·1%)
Yes	43/1,973 (2·2%)	0/2,160 (0·0%)
Missing	1,855/1,973 (94·0%)	2,158/2,160 (99·9%)
VIOLATION: Do you now chew tobacco?		
No	78/1,973 (4·0%)	5/2,160 (0·2%)
Yes	25/1,973 (1·3%)	2/2,160 (0·1%)
Missing	1,870/1,973 (94·8%)	2,153/2,160 (99·7%)
VIOLATION: Excessive alcohol intake		
No	368/1,973 (18·7%)	661/2,160 (30·6%)
Yes	144/1,973 (7·3%)	78/2,160 (3·6%)
No data	88/1,973 (4·5%)	172/2,160 (8·0%)
Missing	1,373/1,973 (69·6%)	1,249/2,160 (57·8%)
VIOLATION: Ferritin<15ug/L or Ferritin>150/200ug/L (F/M)		
No	1,370/1,973 (69·4%)	1,486/2,160 (68·8%)
Yes	603/1,973 (30·6%)	674/2,160 (31·2%)
VIOLATION: C-Reactive Protein>5mg/L		
No	1,678/1,973 (85·0%)	1,475/2,160 (68·3%)
Yes	295/1,973 (15·0%)	685/2,160 (31·7%)
VIOLATION: eGFR<90 ml/min/1.73m^2		
No	1,419/1,973 (71·9%)	1,726/2,160 (79·9%)
Yes	548/1,973 (27·8%)	414/2,160 (19·2%)
No data	6/1,973 (0·3%)	20/2,160 (0·9%)

US = United States, NHANES = National Health and Nutrition Examination Survey, BMI = Body Mass Index, SP = Survey Participant, F = Female, M = Male.
Data are presented as unweighted n/total (%) based on participants with non-missing haemoglobin, ferritin, and C-reactive protein.
No data: No answer/refused, Don't know; Missing: Not applicable, Not asked. If a measurement of one or more of the healthy criteria is missing, then the participant is included in the reference sample.
[1] Males: ferritin≥15 and ≤200ug/L and C-reactive protein≤5mg/L; females: ferritin≥15 and ≤150ug/L and C-reactive protein≤5mg/L.

Table 4.1.3 US NHANES 2003-2004: Participant characteristics of violations (Females) (18-65 years).

	Overall sample<br>Female<br>N=1,455
Reference sample[1]	
No	1,337/1,455 (91·9%)
Yes	118/1,455 (8·1%)
VIOLATION: BMI<18.5 or >30 kg/m^2	
No	903/1,455 (62·1%)
Yes	530/1,455 (36·4%)
No data	22/1,455 (1·5%)
VIOLATION: Doctor told you have diabetes	
No	1,417/1,455 (97·4%)
Yes	37/1,455 (2·5%)
No data	1/1,455 (0·1%)
VIOLATION: Ever told you had weak/failing kidneys?	
No	1,166/1,455 (80·1%)
Yes	16/1,455 (1·1%)
No data	5/1,455 (0·3%)
Missing	268/1,455 (18·4%)
VIOLATION: Still have asthma	
No	79/1,455 (5·4%)
Yes	143/1,455 (9·8%)
No data	4/1,455 (0·3%)
Missing	1,229/1,455 (84·5%)
VIOLATION: Taking treatment for anaemia/past 3 months	
No	1,378/1,455 (94·7%)
Yes	76/1,455 (5·2%)
No data	1/1,455 (0·1%)
VIOLATION: Doctor ever said you had arthritis	
No	1,048/1,455 (72·0%)
Yes	136/1,455 (9·3%)
No data	3/1,455 (0·2%)
Missing	268/1,455 (18·4%)
VIOLATION: Ever told had congestive heart failure	
No	1,180/1,455 (81·1%)
Yes	5/1,455 (0·3%)
No data	2/1,455 (0·1%)
Missing	268/1,455 (18·4%)
VIOLATION: Ever told you had coronary heart disease	
No	1,180/1,455 (81·1%)
Yes	5/1,455 (0·3%)
No data	2/1,455 (0·1%)
Missing	268/1,455 (18·4%)
VIOLATION: Ever told you had angina/angina pectoris	
No	1,176/1,455 (80·8%)
Yes	9/1,455 (0·6%)
No data	2/1,455 (0·1%)
Missing	268/1,455 (18·4%)
VIOLATION: Ever told you had heart attack	
No	1,178/1,455 (81·0%)
Yes	8/1,455 (0·5%)
No data	1/1,455 (0·1%)
Missing	268/1,455 (18·4%)
VIOLATION: Ever told you had a stroke	
No	1,175/1,455 (80·8%)
Yes	11/1,455 (0·8%)
No data	1/1,455 (0·1%)
Missing	268/1,455 (18·4%)
VIOLATION: Do you still have thyroid problem	
No	21/1,455 (1·4%)
Yes	77/1,455 (5·3%)
No data	6/1,455 (0·4%)
Missing	1,351/1,455 (92·9%)
VIOLATION: Ever told you had emphysema	
No	1,179/1,455 (81·0%)
Yes	7/1,455 (0·5%)
No data	1/1,455 (0·1%)
Missing	268/1,455 (18·4%)

	Overall sample<br>Female<br>N=1,455
VIOLATION: Ever told you had chronic bronchitis	
No	1,109/1,455 (76·2%)
Yes	76/1,455 (5·2%)
No data	2/1,455 (0·1%)
Missing	268/1,455 (18·4%)
VIOLATION: Ever told you had any liver condition	
No	1,163/1,455 (79·9%)
Yes	21/1,455 (1·4%)
No data	3/1,455 (0·2%)
Missing	268/1,455 (18·4%)
VIOLATION: Ever told you had cancer or malignancy	
No	1,141/1,455 (78·4%)
Yes	41/1,455 (2·8%)
No data	5/1,455 (0·3%)
Missing	268/1,455 (18·4%)
VIOLATION: Overnight hospital patient in last year	
No	1,269/1,455 (87·2%)
Yes	186/1,455 (12·8%)
VIOLATION: SP have head cold or chest cold?	
No	1,040/1,455 (71·5%)
Yes	272/1,455 (18·7%)
No data	143/1,455 (9·8%)
VIOLATION: SP have stomach or intestinal illness?	
No	1,188/1,455 (81·6%)
Yes	124/1,455 (8·5%)
No data	143/1,455 (9·8%)
VIOLATION: SP have flu, pneumonia, ear infection?	
No	1,237/1,455 (85·0%)
Yes	73/1,455 (5·0%)
No data	145/1,455 (10·0%)
VIOLATION: SP donated blood in past 12 months	
No	1,229/1,455 (84·5%)
Yes	81/1,455 (5·6%)
No data	145/1,455 (10·0%)
VIOLATION: Are you pregnant now?	
No	645/1,455 (44·3%)
Yes	198/1,455 (13·6%)
No data	612/1,455 (42·1%)
VIOLATION: Pregnancy status at exam	
No	1,188/1,455 (81·6%)
Yes	230/1,455 (15·8%)
No data	37/1,455 (2·5%)
VIOLATION: Urine Pregnancy Result	
No	1,217/1,455 (83·6%)
Yes	228/1,455 (15·7%)
No data	10/1,455 (0·7%)
VIOLATION: Taken prescription medicine, past month	
No	831/1,455 (57·1%)
Yes	622/1,455 (42·7%)
No data	2/1,455 (0·1%)
VIOLATION: Do you now smoke cigarettes?	
No	170/1,455 (11·7%)
Yes	291/1,455 (20·0%)
Missing	994/1,455 (68·3%)
VIOLATION: Smoked tobacco last 5 days?	
No	974/1,455 (66·9%)
Yes	335/1,455 (23·0%)
No data	146/1,455 (10·0%)
VIOLATION: Do you now smoke a pipe?	
No	3/1,455 (0·2%)
Missing	1,452/1,455 (99·8%)
VIOLATION: Do you now smoke cigars?	
No	3/1,455 (0·2%)
Yes	9/1,455 (0·6%)
Missing	1,443/1,455 (99·2%)
VIOLATION: Do you now use snuff?	
No	3/1,455 (0·2%)

	Overall sample Female N=1,455
Yes	1/1,455 (0·1%)
Missing	1,451/1,455 (99·7%)
VIOLATION: Do you now chew tobacco?	
Missing	1,455/1,455 (100·0%)
VIOLATION: Excessive alcohol intake	
No	354/1,455 (24·3%)
Yes	51/1,455 (3·5%)
No data	131/1,455 (9·0%)
Missing	919/1,455 (63·2%)
VIOLATION: Ferritin<15ug/L or Ferritin>150ug/L	
No	1,120/1,455 (77·0%)
Yes	335/1,455 (23·0%)
VIOLATION: C-Reactive Protein>5mg/L	
No	986/1,455 (67·8%)
Yes	469/1,455 (32·2%)
VIOLATION: eGFR<90 ml/min/1.73m^2	
No	1,315/1,455 (90·4%)
Yes	131/1,455 (9·0%)
No data	9/1,455 (0·6%)

US = United States, NHANES = National Health and Nutrition Examination Survey, BMI = Body Mass Index, SP = Survey Participant.
Data are presented as unweighted n/total (%) based on participants with non-missing haemoglobin, ferritin, and C-reactive protein. No ferritin was measured in participants more than 49 years. No ferritin was collected in males.
No data: No answer/refused, Don't know; Missing: Not applicable, Not asked. If a measurement of one or more of the healthy criteria is missing, then the participant is included in the reference sample.
[1] Ferritin≥15 and ≤150ug/L and C-reactive protein≤5mg/L.

Table 4.1.4 US NHANES 2005-2006: Participant characteristics of violations (Females) (18-65 years).

	Overall sample Female N=1,606
Reference sample[1]	
No	1,473/1,606 (91·7%)
Yes	133/1,606 (8·3%)
VIOLATION: BMI<18.5 or >30 kg/m^2	
No	984/1,606 (61·3%)
Yes	611/1,606 (38·0%)
No data	11/1,606 (0·7%)
VIOLATION: Doctor told you have diabetes	
No	1,555/1,606 (96·8%)
Yes	50/1,606 (3·1%)
No data	1/1,606 (0·1%)
VIOLATION: Ever told you had weak/failing kidneys?	
No	1,313/1,606 (81·8%)
Yes	29/1,606 (1·8%)
No data	5/1,606 (0·3%)
Missing	259/1,606 (16·1%)
VIOLATION: Still have asthma	
No	86/1,606 (5·4%)
Yes	153/1,606 (9·5%)
No data	4/1,606 (0·2%)
Missing	1,363/1,606 (84·9%)
VIOLATION: Taking treatment for anaemia/past 3 months	
No	1,515/1,606 (94·3%)
Yes	90/1,606 (5·6%)
No data	1/1,606 (0·1%)
VIOLATION: Doctor ever said you had arthritis	
No	1,202/1,606 (74·8%)
Yes	143/1,606 (8·9%)
No data	2/1,606 (0·1%)
Missing	259/1,606 (16·1%)
VIOLATION: Ever told had congestive heart failure	
No	1,337/1,606 (83·3%)
Yes	8/1,606 (0·5%)
No data	2/1,606 (0·1%)
Missing	259/1,606 (16·1%)
VIOLATION: Ever told you had coronary heart disease	
No	1,338/1,606 (83·3%)
Yes	7/1,606 (0·4%)
No data	2/1,606 (0·1%)
Missing	259/1,606 (16·1%)
VIOLATION: Ever told you had angina/angina pectoris	
No	1,336/1,606 (83·2%)
Yes	8/1,606 (0·5%)
No data	3/1,606 (0·2%)
Missing	259/1,606 (16·1%)
VIOLATION: Ever told you had heart attack	
No	1,339/1,606 (83·4%)
Yes	7/1,606 (0·4%)
No data	1/1,606 (0·1%)
Missing	259/1,606 (16·1%)
VIOLATION: Ever told you had a stroke	
No	1,331/1,606 (82·9%)
Yes	16/1,606 (1·0%)
Missing	259/1,606 (16·1%)
VIOLATION: Do you still have thyroid problem	
No	23/1,606 (1·4%)
Yes	71/1,606 (4·4%)
No data	4/1,606 (0·2%)
Missing	1,508/1,606 (93·9%)
VIOLATION: Ever told you had emphysema	
No	1,342/1,606 (83·6%)
Yes	5/1,606 (0·3%)
Missing	259/1,606 (16·1%)
VIOLATION: Ever told you had chronic bronchitis	
No	1,264/1,606 (78·7%)

	Overall sample<br>Female<br>N=1,606
Yes	80/1,606 (5·0%)
No data	3/1,606 (0·2%)
Missing	259/1,606 (16·1%)
VIOLATION: Ever told you had any liver condition	
No	1,316/1,606 (81·9%)
Yes	30/1,606 (1·9%)
No data	1/1,606 (0·1%)
Missing	259/1,606 (16·1%)
VIOLATION: Ever told you had cancer or malignancy	
No	1,296/1,606 (80·7%)
Yes	51/1,606 (3·2%)
Missing	259/1,606 (16·1%)
VIOLATION: Overnight hospital patient in last year	
No	1,401/1,606 (87·2%)
Yes	205/1,606 (12·8%)
VIOLATION: SP have head cold or chest cold?	
No	1,170/1,606 (72·9%)
Yes	309/1,606 (19·2%)
No data	127/1,606 (7·9%)
VIOLATION: SP have stomach or intestinal illness?	
No	1,321/1,606 (82·3%)
Yes	160/1,606 (10·0%)
No data	125/1,606 (7·8%)
VIOLATION: SP have flu, pneumonia, ear infection?	
No	1,405/1,606 (87·5%)
Yes	76/1,606 (4·7%)
No data	125/1,606 (7·8%)
VIOLATION: SP donated blood in past 12 months	
No	1,404/1,606 (87·4%)
Yes	77/1,606 (4·8%)
No data	125/1,606 (7·8%)
VIOLATION: Are you pregnant now?	
No	711/1,606 (44·3%)
Yes	299/1,606 (18·6%)
No data	596/1,606 (37·1%)
VIOLATION: Pregnancy status at exam	
No	1,229/1,606 (76·5%)
Yes	334/1,606 (20·8%)
No data	43/1,606 (2·7%)
VIOLATION: Urine Pregnancy Result	
No	1,259/1,606 (78·4%)
Yes	331/1,606 (20·6%)
No data	16/1,606 (1·0%)
VIOLATION: Taken prescription medicine, past month	
No	989/1,606 (61·6%)
Yes	616/1,606 (38·4%)
No data	1/1,606 (0·1%)
VIOLATION: Do you now smoke cigarettes?	
No	214/1,606 (13·3%)
Yes	270/1,606 (16·8%)
Missing	1,122/1,606 (69·9%)
VIOLATION: Smoked tobacco last 5 days?	
No	1,152/1,606 (71·7%)
Yes	321/1,606 (20·0%)
No data	133/1,606 (8·3%)
VIOLATION: Excessive alcohol intake	
No	423/1,606 (26·3%)
Yes	54/1,606 (3·4%)
No data	116/1,606 (7·2%)
Missing	1,013/1,606 (63·1%)
VIOLATION: Ferritin<15ug/L or Ferritin>150ug/L	
No	1,226/1,606 (76·3%)
Yes	380/1,606 (23·7%)
VIOLATION: C-Reactive Protein>5mg/L	
No	1,074/1,606 (66·9%)
Yes	532/1,606 (33·1%)
VIOLATION: eGFR<90 ml/min/1.73m^2	

	Overall sample Female N=1,606
No	1,414/1,606 (88·0%)
Yes	185/1,606 (11·5%)
No data	7/1,606 (0·4%)

US = United States, NHANES = National Health and Nutrition Examination Survey, BMI = Body Mass Index, SP = Survey Participant.
Data are presented as unweighted n/total (%) based on participants with non-missing haemoglobin, ferritin, and C-reactive protein. No ferritin was measured in participants more than 49 years. No ferritin was collected in males.
No data: No answer/refused, Don't know; Missing: Not applicable, Not asked. If a measurement of one or more of the healthy criteria is missing, then the participant is included in the reference sample.
[1] Ferritin≥15 and ≤150ug/L and C-reactive protein≤5mg/L.

Table 4.1.5 US NHANES 2007-2008: Participant characteristics of violations (Females) (18-65 years).

	Overall sample<br>Female<br>N=1,434
Reference sample[1]	
No	1,297/1,434 (90·4%)
Yes	137/1,434 (9·6%)
VIOLATION: BMI<18.5 or >30 kg/m^2	
No	871/1,434 (60·7%)
Yes	542/1,434 (37·8%)
No data	21/1,434 (1·5%)
VIOLATION: Doctor told you have diabetes	
No	1,376/1,434 (96·0%)
Yes	57/1,434 (4·0%)
No data	1/1,434 (0·1%)
VIOLATION: Ever told you had weak/failing kidneys?	
No	1,294/1,434 (90·2%)
Yes	29/1,434 (2·0%)
Missing	111/1,434 (7·7%)
VIOLATION: Still have asthma	
No	82/1,434 (5·7%)
Yes	128/1,434 (8·9%)
No data	7/1,434 (0·5%)
Missing	1,217/1,434 (84·9%)
VIOLATION: Taking treatment for anaemia/past 3 months	
No	1,355/1,434 (94·5%)
Yes	79/1,434 (5·5%)
VIOLATION: Doctor ever said you had arthritis	
No	1,139/1,434 (79·4%)
Yes	183/1,434 (12·8%)
No data	1/1,434 (0·1%)
Missing	111/1,434 (7·7%)
VIOLATION: Ever told had congestive heart failure	
No	1,313/1,434 (91·6%)
Yes	9/1,434 (0·6%)
No data	1/1,434 (0·1%)
Missing	111/1,434 (7·7%)
VIOLATION: Ever told you had coronary heart disease	
No	1,318/1,434 (91·9%)
Yes	3/1,434 (0·2%)
No data	2/1,434 (0·1%)
Missing	111/1,434 (7·7%)
VIOLATION: Ever told you had angina/angina pectoris	
No	1,317/1,434 (91·8%)
Yes	4/1,434 (0·3%)
No data	2/1,434 (0·1%)
Missing	111/1,434 (7·7%)
VIOLATION: Ever told you had heart attack	
No	1,309/1,434 (91·3%)
Yes	13/1,434 (0·9%)
No data	1/1,434 (0·1%)
Missing	111/1,434 (7·7%)
VIOLATION: Ever told you had a stroke	
No	1,306/1,434 (91·1%)
Yes	14/1,434 (1·0%)
No data	3/1,434 (0·2%)
Missing	111/1,434 (7·7%)
VIOLATION: Do you still have thyroid problem	
No	30/1,434 (2·1%)
Yes	77/1,434 (5·4%)
No data	5/1,434 (0·3%)
Missing	1,322/1,434 (92·2%)
VIOLATION: Ever told you had emphysema	
No	1,311/1,434 (91·4%)
Yes	11/1,434 (0·8%)
No data	1/1,434 (0·1%)
Missing	111/1,434 (7·7%)
VIOLATION: Ever told you had chronic bronchitis	
No	1,241/1,434 (86·5%)

	Overall sample<br>Female<br>N=1,434
Yes	81/1,434 (5·6%)
No data	1/1,434 (0·1%)
Missing	111/1,434 (7·7%)
VIOLATION: Ever told you had any liver condition	
No	1,277/1,434 (89·1%)
Yes	46/1,434 (3·2%)
Missing	111/1,434 (7·7%)
VIOLATION: Ever told you had cancer or malignancy	
No	1,262/1,434 (88·0%)
Yes	59/1,434 (4·1%)
No data	2/1,434 (0·1%)
Missing	111/1,434 (7·7%)
VIOLATION: Doctor ever told you that you had gout?	
No	1,311/1,434 (91·4%)
Yes	10/1,434 (0·7%)
No data	2/1,434 (0·1%)
Missing	111/1,434 (7·7%)
VIOLATION: Overnight hospital patient in last year	
No	1,247/1,434 (87·0%)
Yes	187/1,434 (13·0%)
VIOLATION: SP have head cold or chest cold?	
No	1,051/1,434 (73·3%)
Yes	259/1,434 (18·1%)
No data	124/1,434 (8·6%)
VIOLATION: SP have stomach or intestinal illness?	
No	1,142/1,434 (79·6%)
Yes	171/1,434 (11·9%)
No data	121/1,434 (8·4%)
VIOLATION: SP have flu, pneumonia, ear infection?	
No	1,238/1,434 (86·3%)
Yes	74/1,434 (5·2%)
No data	122/1,434 (8·5%)
VIOLATION: SP donated blood in past 12 months	
No	1,251/1,434 (87·2%)
Yes	62/1,434 (4·3%)
No data	121/1,434 (8·4%)
VIOLATION: Are you pregnant now?	
No	680/1,434 (47·4%)
Yes	40/1,434 (2·8%)
No data	369/1,434 (25·7%)
Missing	345/1,434 (24·1%)
VIOLATION: Pregnancy status at exam	
No	1,020/1,434 (71·1%)
Yes	49/1,434 (3·4%)
No data	20/1,434 (1·4%)
Missing	345/1,434 (24·1%)
VIOLATION: Urine Pregnancy Result	
No	1,028/1,434 (71·7%)
Yes	49/1,434 (3·4%)
No data	12/1,434 (0·8%)
Missing	345/1,434 (24·1%)
VIOLATION: How many months ago have baby?	
No	44/1,434 (3·1%)
Yes	78/1,434 (5·4%)
No data	3/1,434 (0·2%)
Missing	1,309/1,434 (91·3%)
VIOLATION: Taken prescription medicine, past month	
No	798/1,434 (55·6%)
Yes	636/1,434 (44·4%)
VIOLATION: For anaemia, such as low iron	
Yes	51/1,434 (3·6%)
No data	1,383/1,434 (96·4%)
VIOLATION: Do you now smoke cigarettes?	
No	166/1,434 (11·6%)
Yes	317/1,434 (22·1%)
Missing	951/1,434 (66·3%)
VIOLATION: Smoked tobacco last 5 days?	

	Overall sample<br>Female<br>N=1,434
No	974/1,434 (67·9%)
Yes	339/1,434 (23·6%)
No data	121/1,434 (8·4%)
VIOLATION: Excessive alcohol intake	
No	364/1,434 (25·4%)
Yes	63/1,434 (4·4%)
No data	110/1,434 (7·7%)
Missing	897/1,434 (62·6%)
VIOLATION: Ferritin<15ug/L or Ferritin>150ug/L	
No	1,102/1,434 (76·8%)
Yes	332/1,434 (23·2%)
VIOLATION: C-Reactive Protein>5mg/L	
No	1,068/1,434 (74·5%)
Yes	366/1,434 (25·5%)
VIOLATION: eGFR<90 ml/min/1.73m^2	
No	1,261/1,434 (87·9%)
Yes	168/1,434 (11·7%)
No data	5/1,434 (0·3%)

US = United States, NHANES = National Health and Nutrition Examination Survey, BMI = Body Mass Index, SP = Survey Participant.
Data are presented as unweighted n/total (%) based on participants with non-missing haemoglobin, ferritin, and C-reactive protein. No ferritin was measured in participants more than 49 years. No ferritin was collected in males.
No data: No answer/refused, Don't know; Missing: Not applicable, Not asked. If a measurement of one or more of the healthy criteria is missing, then the participant is included in the reference sample.
[1] Ferritin≥15 and ≤150ug/L and C-reactive protein≤5mg/L.

Table 4.1.6 US NHANES 2009-2010: Participant characteristics of violations (Females) (18-65 years).

	Overall sample Female N=1,703
Reference sample[1]	
No	1,536/1,703 (90·2%)
Yes	167/1,703 (9·8%)
VIOLATION: BMI<18.5 or >30 kg/m^2	
No	1,020/1,703 (59·9%)
Yes	672/1,703 (39·5%)
No data	11/1,703 (0·6%)
VIOLATION: Doctor told you have diabetes	
No	1,647/1,703 (96·7%)
Yes	54/1,703 (3·2%)
No data	2/1,703 (0·1%)
VIOLATION: Ever told you had weak/failing kidneys?	
No	1,553/1,703 (91·2%)
Yes	22/1,703 (1·3%)
No data	2/1,703 (0·1%)
Missing	126/1,703 (7·4%)
VIOLATION: Still have asthma	
No	114/1,703 (6·7%)
Yes	169/1,703 (9·9%)
No data	6/1,703 (0·4%)
Missing	1,414/1,703 (83·0%)
VIOLATION: Taking treatment for anaemia/past 3 months	
No	1,606/1,703 (94·3%)
Yes	97/1,703 (5·7%)
VIOLATION: Doctor ever said you had arthritis	
No	1,390/1,703 (81·6%)
Yes	185/1,703 (10·9%)
No data	2/1,703 (0·1%)
Missing	126/1,703 (7·4%)
VIOLATION: Ever told had congestive heart failure	
No	1,567/1,703 (92·0%)
Yes	7/1,703 (0·4%)
No data	3/1,703 (0·2%)
Missing	126/1,703 (7·4%)
VIOLATION: Ever told you had coronary heart disease	
No	1,570/1,703 (92·2%)
Yes	4/1,703 (0·2%)
No data	3/1,703 (0·2%)
Missing	126/1,703 (7·4%)
VIOLATION: Ever told you had angina/angina pectoris	
No	1,569/1,703 (92·1%)
Yes	6/1,703 (0·4%)
No data	2/1,703 (0·1%)
Missing	126/1,703 (7·4%)
VIOLATION: Ever told you had heart attack	
No	1,559/1,703 (91·5%)
Yes	13/1,703 (0·8%)
No data	5/1,703 (0·3%)
Missing	126/1,703 (7·4%)
VIOLATION: Ever told you had a stroke	
No	1,557/1,703 (91·4%)
Yes	20/1,703 (1·2%)
Missing	126/1,703 (7·4%)
VIOLATION: Do you still have thyroid problem	
No	23/1,703 (1·4%)
Yes	97/1,703 (5·7%)
No data	7/1,703 (0·4%)
Missing	1,576/1,703 (92·5%)
VIOLATION: Ever told you had emphysema	
No	1,563/1,703 (91·8%)
Yes	14/1,703 (0·8%)
Missing	126/1,703 (7·4%)
VIOLATION: Ever told you had chronic bronchitis	
No	1,500/1,703 (88·1%)
Yes	76/1,703 (4·5%)

	Overall sample<br>Female<br>N=1,703
No data	1/1,703 (0·1%)
Missing	126/1,703 (7·4%)
VIOLATION: Ever told you had any liver condition	
No	1,542/1,703 (90·5%)
Yes	34/1,703 (2·0%)
No data	1/1,703 (0·1%)
Missing	126/1,703 (7·4%)
VIOLATION: Ever told you had cancer or malignancy	
No	1,503/1,703 (88·3%)
Yes	74/1,703 (4·3%)
Missing	126/1,703 (7·4%)
VIOLATION: Doctor ever told you that you had gout?	
No	1,570/1,703 (92·2%)
Yes	7/1,703 (0·4%)
Missing	126/1,703 (7·4%)
VIOLATION: Overnight hospital patient in last year	
No	1,460/1,703 (85·7%)
Yes	243/1,703 (14·3%)
VIOLATION: SP have head cold or chest cold?	
No	1,175/1,703 (69·0%)
Yes	280/1,703 (16·4%)
No data	248/1,703 (14·6%)
VIOLATION: SP have stomach or intestinal illness?	
No	1,348/1,703 (79·2%)
Yes	108/1,703 (6·3%)
No data	247/1,703 (14·5%)
VIOLATION: SP have flu, pneumonia, ear infection?	
No	1,384/1,703 (81·3%)
Yes	67/1,703 (3·9%)
No data	252/1,703 (14·8%)
VIOLATION: SP donated blood in past 12 months	
No	1,368/1,703 (80·3%)
Yes	88/1,703 (5·2%)
No data	247/1,703 (14·5%)
VIOLATION: Are you pregnant now?	
No	713/1,703 (41·9%)
Yes	44/1,703 (2·6%)
No data	549/1,703 (32·2%)
Missing	397/1,703 (23·3%)
VIOLATION: Pregnancy status at exam	
No	1,212/1,703 (71·2%)
Yes	64/1,703 (3·8%)
No data	30/1,703 (1·8%)
Missing	397/1,703 (23·3%)
VIOLATION: Urine Pregnancy Result	
No	1,232/1,703 (72·3%)
Yes	64/1,703 (3·8%)
No data	10/1,703 (0·6%)
Missing	397/1,703 (23·3%)
VIOLATION: How many months ago have baby?	
No	58/1,703 (3·4%)
Yes	80/1,703 (4·7%)
Missing	1,565/1,703 (91·9%)
VIOLATION: Taken prescription medicine, past month	
No	942/1,703 (55·3%)
Yes	761/1,703 (44·7%)
VIOLATION: For anaemia, such as low iron	
Yes	53/1,703 (3·1%)
No data	1,650/1,703 (96·9%)
VIOLATION: Do you now smoke cigarettes?	
No	187/1,703 (11·0%)
Yes	397/1,703 (23·3%)
Missing	1,119/1,703 (65·7%)
VIOLATION: Smoked tobacco last 5 days?	
No	1,072/1,703 (62·9%)
Yes	382/1,703 (22·4%)
No data	249/1,703 (14·6%)

	Overall sample<br>Female<br>N=1,703
VIOLATION: Excessive alcohol intake	
No	370/1,703 (21·7%)
Yes	80/1,703 (4·7%)
No data	234/1,703 (13·7%)
Missing	1,019/1,703 (59·8%)
VIOLATION: Ferritin<15ug/L or Ferritin>150ug/L (F/M)	
No	1,308/1,703 (76·8%)
Yes	395/1,703 (23·2%)
VIOLATION: C-Reactive Protein>5mg/L	
No	1,267/1,703 (74·4%)
Yes	436/1,703 (25·6%)
VIOLATION: eGFR<90 ml/min/1.73m^2	
No	1,465/1,703 (86·0%)
Yes	222/1,703 (13·0%)
No data	16/1,703 (0·9%)

US = United States, NHANES = National Health and Nutrition Examination Survey, BMI = Body Mass Index, SP = Survey Participant.
Data are presented as unweighted n/total (%) based on participants with non-missing haemoglobin, ferritin, and C-reactive protein. No ferritin was measured in participants more than 49 years. No ferritin was collected in males.
No data: No answer/refused, Don't know; Missing: Not applicable, Not asked. If a measurement of one or more of the healthy criteria is missing, then the participant is included in the reference sample.
[1] Ferritin≥15 and ≤150ug/L and C-reactive protein≤5mg/L.

Table 4.1.7 US NHANES 2015-2016: Participant characteristics of violations (Females) (18-65 years).

	Overall sample Female N=1,485
Reference sample[1]	
No	1,317/1,485 (88·7%)
Yes	168/1,485 (11·3%)
VIOLATION: BMI<18.5 or >30 kg/m^2	
No	830/1,485 (55·9%)
Yes	643/1,485 (43·3%)
No data	12/1,485 (0·8%)
VIOLATION: Doctor told you have diabetes	
No	1,403/1,485 (94·5%)
Yes	81/1,485 (5·5%)
No data	1/1,485 (0·1%)
VIOLATION: Ever told you had weak/failing kidneys?	
No	1,352/1,485 (91·0%)
Yes	23/1,485 (1·5%)
No data	1/1,485 (0·1%)
Missing	109/1,485 (7·3%)
VIOLATION: Still have asthma	
No	91/1,485 (6·1%)
Yes	157/1,485 (10·6%)
No data	4/1,485 (0·3%)
Missing	1,233/1,485 (83·0%)
VIOLATION: Taking treatment for anaemia/past 3 months	
No	1,384/1,485 (93·2%)
Yes	99/1,485 (6·7%)
No data	2/1,485 (0·1%)
VIOLATION: Doctor ever said you had arthritis	
No	1,209/1,485 (81·4%)
Yes	165/1,485 (11·1%)
No data	2/1,485 (0·1%)
Missing	109/1,485 (7·3%)
VIOLATION: Ever told had congestive heart failure	
No	1,369/1,485 (92·2%)
Yes	7/1,485 (0·5%)
Missing	109/1,485 (7·3%)
VIOLATION: Ever told you had coronary heart disease	
No	1,369/1,485 (92·2%)
Yes	6/1,485 (0·4%)
No data	1/1,485 (0·1%)
Missing	109/1,485 (7·3%)
VIOLATION: Ever told you had angina/angina pectoris	
No	1,366/1,485 (92·0%)
Yes	9/1,485 (0·6%)
No data	1/1,485 (0·1%)
Missing	109/1,485 (7·3%)
VIOLATION: Ever told you had heart attack	
No	1,365/1,485 (91·9%)
Yes	11/1,485 (0·7%)
Missing	109/1,485 (7·3%)
VIOLATION: Ever told you had a stroke	
No	1,364/1,485 (91·9%)
Yes	12/1,485 (0·8%)
Missing	109/1,485 (7·3%)
VIOLATION: Do you still have thyroid problem	
No	29/1,485 (2·0%)
Yes	81/1,485 (5·5%)
No data	13/1,485 (0·9%)
Missing	1,362/1,485 (91·7%)
VIOLATION: Ever told you had emphysema	
No	1,370/1,485 (92·3%)
Yes	6/1,485 (0·4%)
Missing	109/1,485 (7·3%)
VIOLATION: Ever told you had chronic bronchitis	
No	1,305/1,485 (87·9%)
Yes	67/1,485 (4·5%)
No data	4/1,485 (0·3%)

	Overall sample<br>Female<br>N=1,485
Missing	109/1,485 (7·3%)
VIOLATION: Ever told you had any liver condition	
No	1,340/1,485 (90·2%)
Yes	32/1,485 (2·2%)
No data	4/1,485 (0·3%)
Missing	109/1,485 (7·3%)
VIOLATION: Ever told you had COPD?	
No	1,363/1,485 (91·8%)
Yes	13/1,485 (0·9%)
Missing	109/1,485 (7·3%)
VIOLATION: Ever been told you have jaundice?	
No	1,452/1,485 (97·8%)
Yes	31/1,485 (2·1%)
No data	2/1,485 (0·1%)
VIOLATION: Ever told you had cancer or malignancy	
No	1,336/1,485 (90·0%)
Yes	40/1,485 (2·7%)
Missing	109/1,485 (7·3%)
VIOLATION: Ever been told you have jaundice?	
No	1,452/1,485 (97·8%)
Yes	31/1,485 (2·1%)
No data	2/1,485 (0·1%)
VIOLATION: Overnight hospital patient in last year	
No	1,318/1,485 (88·8%)
Yes	167/1,485 (11·2%)
VIOLATION: SP have head cold or chest cold?	
No	1,062/1,485 (71·5%)
Yes	230/1,485 (15·5%)
No data	193/1,485 (13·0%)
VIOLATION: SP have stomach or intestinal illness?	
No	1,177/1,485 (79·3%)
Yes	114/1,485 (7·7%)
No data	194/1,485 (13·1%)
VIOLATION: SP have flu, pneumonia, ear infection?	
No	1,248/1,485 (84·0%)
Yes	43/1,485 (2·9%)
No data	194/1,485 (13·1%)
VIOLATION: SP donated blood in past 12 months	
No	1,216/1,485 (81·9%)
Yes	76/1,485 (5·1%)
No data	193/1,485 (13·0%)
VIOLATION: Are you pregnant now?	
No	611/1,485 (41·1%)
Yes	46/1,485 (3·1%)
No data	59/1,485 (4·0%)
Missing	769/1,485 (51·8%)
VIOLATION: Pregnancy status at exam	
No	1,060/1,485 (71·4%)
Yes	60/1,485 (4·0%)
No data	28/1,485 (1·9%)
Missing	337/1,485 (22·7%)
VIOLATION: Urine Pregnancy Result	
No	1,081/1,485 (72·8%)
Yes	59/1,485 (4·0%)
No data	8/1,485 (0·5%)
Missing	337/1,485 (22·7%)
VIOLATION: How many months ago have baby?	
No	82/1,485 (5·5%)
Yes	84/1,485 (5·7%)
No data	982/1,485 (66·1%)
Missing	337/1,485 (22·7%)
VIOLATION: Taken prescription medicine, past month	
No	840/1,485 (56·6%)
Yes	644/1,485 (43·4%)
No data	1/1,485 (0·1%)
VIOLATION: For anaemia, such as low iron	
Yes	71/1,485 (4·8%)

	Overall sample Female N=1,485
No data	1,414/1,485 (95·2%)
VIOLATION: Do you now smoke cigarettes?	
No	149/1,485 (10·0%)
Yes	220/1,485 (14·8%)
Missing	1,116/1,485 (75·2%)
VIOLATION: Smoked tobacco last 5 days?	
No	1,049/1,485 (70·6%)
Yes	243/1,485 (16·4%)
No data	193/1,485 (13·0%)
VIOLATION: Do you now smoke cigarettes?	
No	401/1,485 (27·0%)
Yes	17/1,485 (1·1%)
Missing	1,067/1,485 (71·9%)
VIOLATION: Excessive alcohol intake	
No	419/1,485 (28·2%)
Yes	54/1,485 (3·6%)
No data	194/1,485 (13·1%)
Missing	818/1,485 (55·1%)
VIOLATION: Ferritin<15ug/L or Ferritin>150ug/L (F/M)	
No	1,120/1,485 (75·4%)
Yes	365/1,485 (24·6%)
VIOLATION: C-Reactive Protein>5mg/L	
No	1,083/1,485 (72·9%)
Yes	402/1,485 (27·1%)
VIOLATION: eGFR<90 ml/min/1.73m^2	
No	1,309/1,485 (88·1%)
Yes	174/1,485 (11·7%)
No data	2/1,485 (0·1%)

US = United States, NHANES = National Health and Nutrition Examination Survey, BMI = Body Mass Index, SP = Survey Participant.
Data are presented as unweighted n/total (%) based on participants with non-missing haemoglobin, ferritin, and C-reactive protein. No ferritin was measured in participants more than 49 years. No ferritin was collected in males. No data: No answer/refused, Don't know; Missing: Not applicable, Not asked. If a measurement of one or more of the healthy criteria is missing, then the participant is included in the reference sample.
[1] Ferritin≥15 and ≤150ug/L and C-reactive protein≤5mg/L.

Table 4.1.8 US NHANES 2017-2018: Participant characteristics of violations (18-65 years).

	Overall sample	
	**Male N=1,854**	**Female N=2,059**
Reference sample[1]		
No	1,705/1,854 (92·0%)	1,916/2,059 (93·1%)
Yes	149/1,854 (8·0%)	143/2,059 (6·9%)
VIOLATION: BMI<18.5 or >30 kg/m^2		
No	1,080/1,854 (58·3%)	1,094/2,059 (53·1%)
Yes	750/1,854 (40·5%)	946/2,059 (45·9%)
No data	24/1,854 (1·3%)	19/2,059 (0·9%)
VIOLATION: Doctor told you have diabetes		
No	1,626/1,854 (87·7%)	1,856/2,059 (90·1%)
Yes	227/1,854 (12·2%)	201/2,059 (9·8%)
No data	1/1,854 (0·1%)	2/2,059 (0·1%)
VIOLATION: Ever told you had weak/failing kidneys?		
No	1,693/1,854 (91·3%)	1,888/2,059 (91·7%)
Yes	45/1,854 (2·4%)	48/2,059 (2·3%)
No data	1/1,854 (0·1%)	1/2,059 (0·0%)
Missing	115/1,854 (6·2%)	122/2,059 (5·9%)
VIOLATION: Still have asthma		
No	125/1,854 (6·7%)	103/2,059 (5·0%)
Yes	140/1,854 (7·6%)	245/2,059 (11·9%)
No data	8/1,854 (0·4%)	5/2,059 (0·2%)
Missing	1,581/1,854 (85·3%)	1,706/2,059 (82·9%)
VIOLATION: Taking treatment for anaemia/past 3 months		
No	1,835/1,854 (99·0%)	1,891/2,059 (91·8%)
Yes	17/1,854 (0·9%)	166/2,059 (8·1%)
No data	2/1,854 (0·1%)	2/2,059 (0·1%)
VIOLATION: Doctor ever said you had arthritis		
No	1,389/1,854 (74·9%)	1,449/2,059 (70·4%)
Yes	346/1,854 (18·7%)	483/2,059 (23·5%)
No data	4/1,854 (0·2%)	5/2,059 (0·2%)
Missing	115/1,854 (6·2%)	122/2,059 (5·9%)
VIOLATION: Ever told had congestive heart failure		
No	1,692/1,854 (91·3%)	1,911/2,059 (92·8%)
Yes	43/1,854 (2·3%)	23/2,059 (1·1%)
No data	4/1,854 (0·2%)	3/2,059 (0·1%)
Missing	115/1,854 (6·2%)	122/2,059 (5·9%)
VIOLATION: Ever told you had coronary heart disease		
No	1,677/1,854 (90·5%)	1,912/2,059 (92·9%)
Yes	58/1,854 (3·1%)	24/2,059 (1·2%)
No data	4/1,854 (0·2%)	1/2,059 (0·0%)
Missing	115/1,854 (6·2%)	122/2,059 (5·9%)
VIOLATION: Ever told you had angina/angina pectoris		
No	1,698/1,854 (91·6%)	1,898/2,059 (92·2%)
Yes	34/1,854 (1·8%)	33/2,059 (1·6%)
No data	7/1,854 (0·4%)	6/2,059 (0·3%)
Missing	115/1,854 (6·2%)	122/2,059 (5·9%)
VIOLATION: Ever told you had heart attack		
No	1,665/1,854 (89·8%)	1,899/2,059 (92·2%)
Yes	71/1,854 (3·8%)	34/2,059 (1·7%)
No data	3/1,854 (0·2%)	4/2,059 (0·2%)
Missing	115/1,854 (6·2%)	122/2,059 (5·9%)
VIOLATION: Ever told you had a stroke		
No	1,683/1,854 (90·8%)	1,877/2,059 (91·2%)
Yes	54/1,854 (2·9%)	57/2,059 (2·8%)
No data	2/1,854 (0·1%)	3/2,059 (0·1%)
Missing	115/1,854 (6·2%)	122/2,059 (5·9%)
VIOLATION: Do you still have thyroid problem		
No	16/1,854 (0·9%)	54/2,059 (2·6%)
Yes	53/1,854 (2·9%)	200/2,059 (9·7%)
No data	3/1,854 (0·2%)	11/2,059 (0·5%)
Missing	1,782/1,854 (96·1%)	1,794/2,059 (87·1%)
VIOLATION: Ever told you had emphysema		
No	1,713/1,854 (92·4%)	1,917/2,059 (93·1%)
Yes	24/1,854 (1·3%)	17/2,059 (0·8%)
No data	2/1,854 (0·1%)	3/2,059 (0·1%)
Missing	115/1,854 (6·2%)	122/2,059 (5·9%)

	Overall sample	
	Male<br>N=1,854	Female<br>N=2,059
VIOLATION: Ever told you had chronic bronchitis		
No	1,643/1,854 (88·6%)	1,809/2,059 (87·9%)
Yes	96/1,854 (5·2%)	125/2,059 (6·1%)
No data	0/1,854 (0·0%)	3/2,059 (0·1%)
Missing	115/1,854 (6·2%)	122/2,059 (5·9%)
VIOLATION: Ever told you had any liver condition		
No	1,633/1,854 (88·1%)	1,849/2,059 (89·8%)
Yes	103/1,854 (5·6%)	82/2,059 (4·0%)
No data	3/1,854 (0·2%)	6/2,059 (0·3%)
Missing	115/1,854 (6·2%)	122/2,059 (5·9%)
VIOLATION: Ever told you had cancer or malignancy		
No	1,668/1,854 (90·0%)	1,806/2,059 (87·7%)
Yes	71/1,854 (3·8%)	129/2,059 (6·3%)
No data	0/1,854 (0·0%)	2/2,059 (0·1%)
Missing	115/1,854 (6·2%)	122/2,059 (5·9%)
VIOLATION: Ever been told you have jaundice?		
No	1,798/1,854 (97·0%)	2,013/2,059 (97·8%)
Yes	53/1,854 (2·9%)	46/2,059 (2·2%)
No data	3/1,854 (0·2%)	0/2,059 (0·0%)
VIOLATION: Ever told you had COPD?		
No	1,680/1,854 (90·6%)	1,869/2,059 (90·8%)
Yes	57/1,854 (3·1%)	67/2,059 (3·3%)
No data	2/1,854 (0·1%)	1/2,059 (0·0%)
Missing	115/1,854 (6·2%)	122/2,059 (5·9%)
VIOLATION: Has DR ever said you have gallstones		
No	1,661/1,854 (89·6%)	1,666/2,059 (80·9%)
Yes	75/1,854 (4·0%)	265/2,059 (12·9%)
No data	3/1,854 (0·2%)	6/2,059 (0·3%)
Missing	115/1,854 (6·2%)	122/2,059 (5·9%)
VIOLATION: Overnight hospital patient in last year		
No	1,721/1,854 (92·8%)	1,848/2,059 (89·8%)
Yes	133/1,854 (7·2%)	211/2,059 (10·2%)
VIOLATION: SP have head cold or chest cold?		
No	1,485/1,854 (80·1%)	1,541/2,059 (74·8%)
Yes	273/1,854 (14·7%)	359/2,059 (17·4%)
No data	96/1,854 (5·2%)	159/2,059 (7·7%)
VIOLATION: SP have stomach or intestinal illness?		
No	1,641/1,854 (88·5%)	1,746/2,059 (84·8%)
Yes	120/1,854 (6·5%)	160/2,059 (7·8%)
No data	93/1,854 (5·0%)	153/2,059 (7·4%)
VIOLATION: SP have flu, pneumonia, ear infection?		
No	1,692/1,854 (91·3%)	1,814/2,059 (88·1%)
Yes	71/1,854 (3·8%)	93/2,059 (4·5%)
No data	91/1,854 (4·9%)	152/2,059 (7·4%)
VIOLATION: SP donated blood in past 12 months		
No	1,644/1,854 (88·7%)	1,820/2,059 (88·4%)
Yes	118/1,854 (6·4%)	87/2,059 (4·2%)
No data	92/1,854 (5·0%)	152/2,059 (7·4%)
VIOLATION: Are you pregnant now?		
No	0/1,854 (0·0%)	579/2,059 (28·1%)
Yes	0/1,854 (0·0%)	37/2,059 (1·8%)
No data	0/1,854 (0·0%)	48/2,059 (2·3%)
Missing	1,854/1,854 (100·0%)	1,395/2,059 (67·8%)
VIOLATION: Pregnancy status at exam		
No	0/1,854 (0·0%)	911/2,059 (44·2%)
Yes	0/1,854 (0·0%)	47/2,059 (2·3%)
No data	0/1,854 (0·0%)	22/2,059 (1·1%)
Missing	1,854/1,854 (100·0%)	1,079/2,059 (52·4%)
VIOLATION: Urine Pregnancy Result		
No	0/1,854 (0·0%)	927/2,059 (45·0%)
Yes	0/1,854 (0·0%)	47/2,059 (2·3%)
No data	0/1,854 (0·0%)	6/2,059 (0·3%)
Missing	1,854/1,854 (100·0%)	1,079/2,059 (52·4%)
VIOLATION: How many months ago have baby?		
No	0/1,854 (0·0%)	904/2,059 (43·9%)
Yes	0/1,854 (0·0%)	76/2,059 (3·7%)
Missing	1,854/1,854 (100·0%)	1,079/2,059 (52·4%)

	Overall sample	
	**Male**<br>**N=1,854**	**Female**<br>**N=2,059**
VIOLATION: Taken prescription medicine, past month		
No	1,039/1,854 (56·0%)	938/2,059 (45·6%)
Yes	812/1,854 (43·8%)	1,117/2,059 (54·2%)
No data	3/1,854 (0·2%)	4/2,059 (0·2%)
VIOLATION: For anaemia, such as low iron		
No	1,829/1,854 (98·7%)	1,930/2,059 (93·7%)
Yes	25/1,854 (1·3%)	129/2,059 (6·3%)
VIOLATION: Do you now smoke cigarettes?		
No	432/1,854 (23·3%)	284/2,059 (13·8%)
Yes	443/1,854 (23·9%)	329/2,059 (16·0%)
Missing	979/1,854 (52·8%)	1,446/2,059 (70·2%)
VIOLATION: Smoked tobacco last 5 days?		
No	1,230/1,854 (66·3%)	1,539/2,059 (74·7%)
Yes	533/1,854 (28·7%)	367/2,059 (17·8%)
No data	91/1,854 (4·9%)	153/2,059 (7·4%)
VIOLATION: Excessive alcohol intake		
No	414/1,854 (22·3%)	548/2,059 (26·6%)
Yes	136/1,854 (7·3%)	84/2,059 (4·1%)
No data	94/1,854 (5·1%)	156/2,059 (7·6%)
Missing	1,210/1,854 (65·3%)	1,271/2,059 (61·7%)
VIOLATION: Ferritin<15ug/L or Ferritin>150/200ug/L (F/M)		
No	1,108/1,854 (59·8%)	1,499/2,059 (72·8%)
Yes	746/1,854 (40·2%)	560/2,059 (27·2%)
VIOLATION: C-Reactive Protein>5mg/L		
No	1,537/1,854 (82·9%)	1,471/2,059 (71·4%)
Yes	317/1,854 (17·1%)	588/2,059 (28·6%)
VIOLATION: eGFR<90 ml/min/1.73m^2		
No	1,322/1,854 (71·3%)	1,606/2,059 (78·0%)
Yes	529/1,854 (28·5%)	450/2,059 (21·9%)
No data	3/1,854 (0·2%)	3/2,059 (0·1%)

US = United States, NHANES = National Health and Nutrition Examination Survey, BMI = Body Mass Index, SP = Survey Participant, F = Female, M = Male.
Data are presented as unweighted n/total (%) based on participants with non-missing haemoglobin, ferritin, and C-reactive protein.
No data: No answer/refused, Don't know; Missing: Not applicable, Not asked. If a measurement of one or more of the healthy criteria is missing, then the participant is included in the reference sample.
[1] Males: ferritin≥15 and ≤200ug/L and C-reactive protein≤5mg/L; females: ferritin≥15 and ≤150ug/L and C-reactive protein≤5mg/L.

Table 4.1.9 England HSE 1998: Participant characteristics of violations (18-65 years).

	Overall sample	
	**Male N=3,835**	**Female N=4,189**
Reference sample[1]		
No	3,268/3,835 (85·2%)	3,613/4,189 (86·2%)
Yes	567/3,835 (14·8%)	576/4,189 (13·8%)
VIOLATION: BMI<18.5 or >30 kg/m[2]		
No	3,009/3,835 (78·5%)	3,120/4,189 (74·5%)
Yes	675/3,835 (17·6%)	871/4,189 (20·8%)
No data	151/3,835 (3·9%)	198/4,189 (4·7%)
VIOLATION: whether has clotting disorder		
No	3,806/3,835 (99·2%)	4,169/4,189 (99·5%)
Yes	29/3,835 (0·8%)	20/4,189 (0·5%)
VIOLATION: (d) had cardiovascular condition		
No	2,946/3,835 (76·8%)	3,330/4,189 (79·5%)
Yes	885/3,835 (23·1%)	855/4,189 (20·4%)
No data	4/3,835 (0·1%)	4/4,189 (0·1%)
VIOLATION: (d) had cvd: excludes those with high bp		
No	3,408/3,835 (88·9%)	3,791/4,189 (90·5%)
Yes	424/3,835 (11·1%)	394/4,189 (9·4%)
No data	3/3,835 (0·1%)	4/4,189 (0·1%)
VIOLATION: (d) had ihd (angina or heart attack)		
No	3,686/3,835 (96·1%)	4,117/4,189 (98·3%)
Yes	148/3,835 (3·9%)	71/4,189 (1·7%)
No data	1/3,835 (0·0%)	1/4,189 (0·0%)
VIOLATION: (d) had cvd (angina, heart attack or stroke)		
No	3,660/3,835 (95·4%)	4,096/4,189 (97·8%)
Yes	174/3,835 (4·5%)	92/4,189 (2·2%)
No data	1/3,835 (0·0%)	1/4,189 (0·0%)
VIOLATION: (d) angina or mi (rose angina qure)		
No	3,474/3,835 (90·6%)	3,905/4,189 (93·2%)
Yes	354/3,835 (9·2%)	282/4,189 (6·7%)
No data	7/3,835 (0·2%)	2/4,189 (0·0%)
VIOLATION: (d) angina symptoms (rose angina qure)		
No	3,762/3,835 (98·1%)	4,087/4,189 (97·6%)
Yes	66/3,835 (1·7%)	101/4,189 (2·4%)
No data	7/3,835 (0·2%)	1/4,189 (0·0%)
VIOLATION: (d) possible infarction (rose angina qure)		
No	3,525/3,835 (91·9%)	3,987/4,189 (95·2%)
Yes	310/3,835 (8·1%)	201/4,189 (4·8%)
No data	0/3,835 (0·0%)	1/4,189 (0·0%)
VIOLATION: (d) doctor diagnosed angina		
No	3,728/3,835 (97·2%)	4,130/4,189 (98·6%)
Yes	107/3,835 (2·8%)	59/4,189 (1·4%)
VIOLATION: (d) doctor diagnosed high blood pressure (excluding pregnant)		
No	3,209/3,835 (83·7%)	3,599/4,189 (85·9%)
Yes	625/3,835 (16·3%)	590/4,189 (14·1%)
No data	1/3,835 (0·0%)	0/4,189 (0·0%)
VIOLATION: (d) doctor diagnosed heart attack		
No	3,745/3,835 (97·7%)	4,163/4,189 (99·4%)
Yes	89/3,835 (2·3%)	25/4,189 (0·6%)
No data	1/3,835 (0·0%)	1/4,189 (0·0%)
VIOLATION: (d) doctor diagnosed stroke		
No	3,796/3,835 (99·0%)	4,162/4,189 (99·4%)
Yes	39/3,835 (1·0%)	27/4,189 (0·6%)
VIOLATION: (d) doctor diagnosed diabetes (excluding pregnant)		
No	3,742/3,835 (97·6%)	4,129/4,189 (98·6%)
Yes	93/3,835 (2·4%)	60/4,189 (1·4%)
VIOLATION: (d) doctor diagnosed heart murmur (excluding pregnant)		
No	3,738/3,835 (97·5%)	4,052/4,189 (96·7%)
Yes	96/3,835 (2·5%)	136/4,189 (3·2%)
No data	1/3,835 (0·0%)	1/4,189 (0·0%)
VIOLATION: (d) doctor diagnosed irregular heart rhythm		
No	3,682/3,835 (96·0%)	4,029/4,189 (96·2%)
Yes	151/3,835 (3·9%)	159/4,189 (3·8%)
No data	2/3,835 (0·1%)	1/4,189 (0·0%)
VIOLATION: (d) doctor diagnosed other heart condition		
No	3,796/3,835 (99·0%)	4,151/4,189 (99·1%)

	Overall sample	
	Male<br>N=3,835	Female<br>N=4,189
Yes	39/3,835 (1·0%)	37/4,189 (0·9%)
No data	0/3,835 (0·0%)	1/4,189 (0·0%)
VIOLATION: surgery for heart condition		
No	3,785/3,835 (98·7%)	4,166/4,189 (99·5%)
Yes	50/3,835 (1·3%)	23/4,189 (0·5%)
VIOLATION: any surgery for heart murmur		
No	3,829/3,835 (99·8%)	4,185/4,189 (99·9%)
Yes	6/3,835 (0·2%)	4/4,189 (0·1%)
VIOLATION: (d) i infectious disease		
No	3,825/3,835 (99·7%)	4,182/4,189 (99·8%)
Yes	8/3,835 (0·2%)	6/4,189 (0·1%)
No data	2/3,835 (0·1%)	1/4,189 (0·0%)
VIOLATION: (d) ii neoplasms & benign growths		
No	3,809/3,835 (99·3%)	4,129/4,189 (98·6%)
Yes	24/3,835 (0·6%)	59/4,189 (1·4%)
No data	2/3,835 (0·1%)	1/4,189 (0·0%)
VIOLATION: (d) iii endocrine & metabolic		
No	3,696/3,835 (96·4%)	4,006/4,189 (95·6%)
Yes	137/3,835 (3·6%)	182/4,189 (4·3%)
No data	2/3,835 (0·1%)	1/4,189 (0·0%)
VIOLATION: (d) iv blood and related organs		
No	3,822/3,835 (99·7%)	4,168/4,189 (99·5%)
Yes	11/3,835 (0·3%)	20/4,189 (0·5%)
No data	2/3,835 (0·1%)	1/4,189 (0·0%)
VIOLATION: (d) v mental disorders		
No	3,757/3,835 (98·0%)	4,066/4,189 (97·1%)
Yes	76/3,835 (2·0%)	122/4,189 (2·9%)
No data	2/3,835 (0·1%)	1/4,189 (0·0%)
VIOLATION: (d) vi nervous system		
No	3,737/3,835 (97·4%)	4,031/4,189 (96·2%)
Yes	96/3,835 (2·5%)	157/4,189 (3·7%)
No data	2/3,835 (0·1%)	1/4,189 (0·0%)
VIOLATION: (d) vi eye complaints		
No	3,778/3,835 (98·5%)	4,143/4,189 (98·9%)
Yes	55/3,835 (1·4%)	45/4,189 (1·1%)
No data	2/3,835 (0·1%)	1/4,189 (0·0%)
VIOLATION: (d) vi ear complaints		
No	3,725/3,835 (97·1%)	4,126/4,189 (98·5%)
Yes	108/3,835 (2·8%)	62/4,189 (1·5%)
No data	2/3,835 (0·1%)	1/4,189 (0·0%)
VIOLATION: (d) vii heart and circulatory system		
No	3,566/3,835 (93·0%)	3,949/4,189 (94·3%)
Yes	267/3,835 (7·0%)	239/4,189 (5·7%)
No data	2/3,835 (0·1%)	1/4,189 (0·0%)
VIOLATION: (d) respiratory system		
No	3,488/3,835 (91·0%)	3,825/4,189 (91·3%)
Yes	345/3,835 (9·0%)	363/4,189 (8·7%)
No data	2/3,835 (0·1%)	1/4,189 (0·0%)
VIOLATION: (d) ix digestive system		
No	3,657/3,835 (95·4%)	4,005/4,189 (95·6%)
Yes	176/3,835 (4·6%)	183/4,189 (4·4%)
No data	2/3,835 (0·1%)	1/4,189 (0·0%)
VIOLATION: (d) x genito-urinary system		
No	3,777/3,835 (98·5%)	4,089/4,189 (97·6%)
Yes	56/3,835 (1·5%)	99/4,189 (2·4%)
No data	2/3,835 (0·1%)	1/4,189 (0·0%)
VIOLATION: (d) xii skin complaints		
No	3,758/3,835 (98·0%)	4,120/4,189 (98·4%)
Yes	75/3,835 (2·0%)	68/4,189 (1·6%)
No data	2/3,835 (0·1%)	1/4,189 (0·0%)
VIOLATION: (d) xiii musculoskeletal system		
No	3,150/3,835 (82·1%)	3,504/4,189 (83·6%)
Yes	683/3,835 (17·8%)	684/4,189 (16·3%)
No data	2/3,835 (0·1%)	1/4,189 (0·0%)
VIOLATION: (d) other complaints		
No	3,830/3,835 (99·9%)	4,186/4,189 (99·9%)
Yes	3/3,835 (0·1%)	2/4,189 (0·0%)

	Overall sample	
	Male<br>N=3,835	Female<br>N=4,189
No data	2/3,835 (0·1%)	1/4,189 (0·0%)
VIOLATION: (d) long standing illness		
No	2,306/3,835 (60·1%)	2,586/4,189 (61·7%)
Yes	1,527/3,835 (39·8%)	1,602/4,189 (38·2%)
No data	2/3,835 (0·1%)	1/4,189 (0·0%)
VIOLATION: (d) acute sickness last 2 weeks		
No	3,343/3,835 (87·2%)	3,483/4,189 (83·1%)
Yes	491/3,835 (12·8%)	705/4,189 (16·8%)
No data	1/3,835 (0·0%)	1/4,189 (0·0%)
VIOLATION: been an inpatient in hospital in last 12 months		
No	3,604/3,835 (94·0%)	3,829/4,189 (91·4%)
Yes	228/3,835 (5·9%)	359/4,189 (8·6%)
No data	3/3,835 (0·1%)	1/4,189 (0·0%)
VIOLATION: whether currently pregnant 16 plus		
No	3,835/3,835 (100·0%)	4,182/4,189 (99·8%)
No data	0/3,835 (0·0%)	7/4,189 (0·2%)
VIOLATION: pregnant in the last 12 months		
No	3,835/3,835 (100·0%)	4,129/4,189 (98·6%)
Yes	0/3,835 (0·0%)	59/4,189 (1·4%)
No data	0/3,835 (0·0%)	1/4,189 (0·0%)
VIOLATION: (d) whether taking medication - excluding contraceptives only		
No	2,680/3,835 (69·9%)	2,489/4,189 (59·4%)
Yes	1,155/3,835 (30·1%)	1,700/4,189 (40·6%)
VIOLATION: Do you currently inject insulin for diabetes?		
No	3,808/3,835 (99·3%)	4,171/4,189 (99·6%)
Yes	27/3,835 (0·7%)	18/4,189 (0·4%)
VIOLATION: Are you currently taking any medicines, tablets or pills		
No	3,793/3,835 (98·9%)	4,157/4,189 (99·2%)
Yes	42/3,835 (1·1%)	32/4,189 (0·8%)
VIOLATION: whether still on hrt		
No	3,835/3,835 (100·0%)	3,664/4,189 (87·5%)
Yes	0/3,835 (0·0%)	520/4,189 (12·4%)
No data	0/3,835 (0·0%)	5/4,189 (0·1%)
VIOLATION: (d) diuretics (blood pressure)		
No	3,768/3,835 (98·3%)	4,057/4,189 (96·8%)
Yes	67/3,835 (1·7%)	132/4,189 (3·2%)
VIOLATION: (d) beta blockers (blood pressure fibrinogen)		
No	3,731/3,835 (97·3%)	4,074/4,189 (97·3%)
Yes	104/3,835 (2·7%)	115/4,189 (2·7%)
VIOLATION: (d) ace inhibitors (blood pressure)		
No	3,729/3,835 (97·2%)	4,127/4,189 (98·5%)
Yes	106/3,835 (2·8%)	62/4,189 (1·5%)
VIOLATION: (d) calcium blockers (blood pressure)		
No	3,739/3,835 (97·5%)	4,125/4,189 (98·5%)
Yes	96/3,835 (2·5%)	64/4,189 (1·5%)
VIOLATION: (d) other drugs affecting bp		
No	3,820/3,835 (99·6%)	4,174/4,189 (99·6%)
Yes	15/3,835 (0·4%)	15/4,189 (0·4%)
VIOLATION: (d) lipid lowering (cholesterol fibrinogen)		
No	3,750/3,835 (97·8%)	4,134/4,189 (98·7%)
Yes	85/3,835 (2·2%)	55/4,189 (1·3%)
VIOLATION: (d) iron deficiency (haemoglobin ferritin)		
No	3,835/3,835 (100·0%)	4,189/4,189 (100·0%)
VIOLATION: (d) whether taking drugs affecting blood pressure		
No	3,559/3,835 (92·8%)	3,897/4,189 (93·0%)
Yes	276/3,835 (7·2%)	292/4,189 (7·0%)
VIOLATION: (d) whether taking drugs prescribed for blood pressure		
No	3,632/3,835 (94·7%)	3,983/4,189 (95·1%)
Yes	203/3,835 (5·3%)	206/4,189 (4·9%)
VIOLATION: (d) units drunk on heaviest day in last 7		
No	2,911/3,835 (75·9%)	3,792/4,189 (90·5%)
Yes	888/3,835 (23·2%)	381/4,189 (9·1%)
No data	36/3,835 (0·9%)	16/4,189 (0·4%)
VIOLATION: (d) cigarette smoking status – never/ ex-regular/ ex-occasional/ current		
No	2,687/3,835 (70·1%)	2,956/4,189 (70·6%)
Yes	1,145/3,835 (29·9%)	1,232/4,189 (29·4%)

	Overall sample	
	Male<br>N=3,835	Female<br>N=4,189
No data	3/3,835 (0·1%)	1/4,189 (0·0%)
VIOLATION: (d) number of cigarettes smoke a day - including non-smokers		
No	2,705/3,835 (70·5%)	2,978/4,189 (71·1%)
Yes	1,125/3,835 (29·3%)	1,207/4,189 (28·8%)
No data	5/3,835 (0·1%)	4/4,189 (0·1%)
VIOLATION: currently smokes cigarettes		
No	2,704/3,835 (70·5%)	2,964/4,189 (70·8%)
Yes	1,131/3,835 (29·5%)	1,225/4,189 (29·2%)
VIOLATION: currently smokes cigars		
No	3,700/3,835 (96·5%)	4,180/4,189 (99·8%)
Yes	135/3,835 (3·5%)	9/4,189 (0·2%)
VIOLATION: currently smokes pipe		
No	3,797/3,835 (99·0%)	4,189/4,189 (100·0%)
Yes	38/3,835 (1·0%)	0/4,189 (0·0%)
VIOLATION: Ferritin<15ug/L or Ferritin>150/200ug/L (F/M)		
No	3,423/3,835 (89·3%)	3,558/4,189 (84·9%)
Yes	412/3,835 (10·7%)	631/4,189 (15·1%)
VIOLATION: C-Reactive Protein>5mg/L		
No	3,437/3,835 (89·6%)	3,472/4,189 (82·9%)
Yes	398/3,835 (10·4%)	717/4,189 (17·1%)

HSE = Health Survey for England, BMI = Body Mass Index, F = Female, M = Male.
Data are presented as unweighted n/total (%) based on participants with non-missing haemoglobin, ferritin, and C-reactive protein.
No data: No answer/refused, Don't know, Not applicable, Not obtained. If a measurement of one or more of the healthy criteria is missing, then the participant is included in the reference sample.
[1] Males: ferritin≥15 and ≤200ug/L and C-reactive protein≤5mg/L; females: ferritin≥15 and ≤150ug/L and C-reactive protein≤5mg/L.

Table 4.1.10 England HSE 2006: Participant characteristics of violations (18-65 years).

	Overall sample	
	**Male N=2,569**	**Female N=3,026**
Reference sample[1]		
No	2,195/2,569 (85·4%)	2,511/3,026 (83·0%)
Yes	374/2,569 (14·6%)	515/3,026 (17·0%)
VIOLATION: BMI<18.5 or >30 kg/m^2		
No	1,820/2,569 (70·8%)	2,162/3,026 (71·4%)
Yes	614/2,569 (23·9%)	682/3,026 (22·5%)
No data	135/2,569 (5·3%)	182/3,026 (6·0%)
VIOLATION: whether has clotting disorder		
No	2,569/2,569 (100·0%)	3,026/3,026 (100·0%)
VIOLATION: (d) had cardiovascular condition		
No	1,843/2,569 (71·7%)	2,269/3,026 (75·0%)
Yes	725/2,569 (28·2%)	752/3,026 (24·9%)
No data	1/2,569 (0·0%)	5/3,026 (0·2%)
VIOLATION: (d) had cvd: excludes those with high bp		
No	2,267/2,569 (88·2%)	2,706/3,026 (89·4%)
Yes	301/2,569 (11·7%)	317/3,026 (10·5%)
No data	1/2,569 (0·0%)	3/3,026 (0·1%)
VIOLATION: (d) had ihd (angina or heart attack)		
No	2,486/2,569 (96·8%)	3,001/3,026 (99·2%)
Yes	83/2,569 (3·2%)	25/3,026 (0·8%)
VIOLATION: (d) had cvd (angina, heart attack or stroke)		
No	2,477/2,569 (96·4%)	2,982/3,026 (98·5%)
Yes	92/2,569 (3·6%)	44/3,026 (1·5%)
VIOLATION: (d) angina or mi (rose angina qure)		
No	2,330/2,569 (90·7%)	2,835/3,026 (93·7%)
Yes	237/2,569 (9·2%)	187/3,026 (6·2%)
No data	2/2,569 (0·1%)	4/3,026 (0·1%)
VIOLATION: (d) angina symptoms (rose angina qure)		
No	2,532/2,569 (98·6%)	2,962/3,026 (97·9%)
Yes	36/2,569 (1·4%)	60/3,026 (2·0%)
No data	1/2,569 (0·0%)	4/3,026 (0·1%)
VIOLATION: (d) possible infarction (rose angina qure)		
No	2,353/2,569 (91·6%)	2,884/3,026 (95·3%)
Yes	215/2,569 (8·4%)	142/3,026 (4·7%)
No data	1/2,569 (0·0%)	0/3,026 (0·0%)
VIOLATION: (d) doctor diagnosed angina		
No	2,509/2,569 (97·7%)	3,007/3,026 (99·4%)
Yes	60/2,569 (2·3%)	19/3,026 (0·6%)
VIOLATION: (d) doctor diagnosed high blood pressure (excluding pregnant)		
No	2,006/2,569 (78·1%)	2,453/3,026 (81·1%)
Yes	563/2,569 (21·9%)	571/3,026 (18·9%)
No data	0/2,569 (0·0%)	2/3,026 (0·1%)
VIOLATION: (d) doctor diagnosed heart attack		
No	2,523/2,569 (98·2%)	3,012/3,026 (99·5%)
Yes	46/2,569 (1·8%)	14/3,026 (0·5%)
VIOLATION: (d) doctor diagnosed stroke		
No	2,554/2,569 (99·4%)	3,003/3,026 (99·2%)
Yes	15/2,569 (0·6%)	23/3,026 (0·8%)
VIOLATION: (d) doctor diagnosed diabetes (excluding pregnant)		
No	2,471/2,569 (96·2%)	2,965/3,026 (98·0%)
Yes	98/2,569 (3·8%)	61/3,026 (2·0%)
VIOLATION: (d) doctor diagnosed heart murmur (excluding pregnant)		
No	2,521/2,569 (98·1%)	2,929/3,026 (96·8%)
Yes	48/2,569 (1·9%)	94/3,026 (3·1%)
No data	0/2,569 (0·0%)	3/3,026 (0·1%)
VIOLATION: (d) doctor diagnosed irregular heart rhythm		
No	2,464/2,569 (95·9%)	2,891/3,026 (95·5%)
Yes	104/2,569 (4·0%)	135/3,026 (4·5%)
No data	1/2,569 (0·0%)	0/3,026 (0·0%)
VIOLATION: (d) doctor diagnosed other heart condition		
No	2,543/2,569 (99·0%)	2,990/3,026 (98·8%)
Yes	26/2,569 (1·0%)	36/3,026 (1·2%)
VIOLATION: surgery for heart condition		
No	2,522/2,569 (98·2%)	3,010/3,026 (99·5%)
Yes	47/2,569 (1·8%)	16/3,026 (0·5%)

	Overall sample	
	**Male** N=2,569	**Female** N=3,026
VIOLATION: any surgery for heart murmur		
No	2,567/2,569 (99·9%)	3,024/3,026 (99·9%)
Yes	2/2,569 (0·1%)	2/3,026 (0·1%)
VIOLATION: (d) i infectious disease		
No	2,565/2,569 (99·8%)	3,021/3,026 (99·8%)
Yes	3/2,569 (0·1%)	4/3,026 (0·1%)
No data	1/2,569 (0·0%)	1/3,026 (0·0%)
VIOLATION: (d) ii neoplasms & benign growths		
No	2,543/2,569 (99·0%)	2,985/3,026 (98·6%)
Yes	25/2,569 (1·0%)	40/3,026 (1·3%)
No data	1/2,569 (0·0%)	1/3,026 (0·0%)
VIOLATION: (d) iii endocrine & metabolic		
No	2,436/2,569 (94·8%)	2,853/3,026 (94·3%)
Yes	132/2,569 (5·1%)	172/3,026 (5·7%)
No data	1/2,569 (0·0%)	1/3,026 (0·0%)
VIOLATION: (d) iv blood and related organs		
No	2,562/2,569 (99·7%)	2,999/3,026 (99·1%)
Yes	6/2,569 (0·2%)	26/3,026 (0·9%)
No data	1/2,569 (0·0%)	1/3,026 (0·0%)
VIOLATION: (d) v mental disorders		
No	2,486/2,569 (96·8%)	2,887/3,026 (95·4%)
Yes	82/2,569 (3·2%)	138/3,026 (4·6%)
No data	1/2,569 (0·0%)	1/3,026 (0·0%)
VIOLATION: (d) vi nervous system		
No	2,491/2,569 (97·0%)	2,893/3,026 (95·6%)
Yes	77/2,569 (3·0%)	132/3,026 (4·4%)
No data	1/2,569 (0·0%)	1/3,026 (0·0%)
VIOLATION: (d) vi eye complaints		
No	2,530/2,569 (98·5%)	2,995/3,026 (99·0%)
Yes	38/2,569 (1·5%)	30/3,026 (1·0%)
No data	1/2,569 (0·0%)	1/3,026 (0·0%)
VIOLATION: (d) vi ear complaints		
No	2,519/2,569 (98·1%)	2,977/3,026 (98·4%)
Yes	49/2,569 (1·9%)	48/3,026 (1·6%)
No data	1/2,569 (0·0%)	1/3,026 (0·0%)
VIOLATION: (d) vii heart and circulatory system		
No	2,351/2,569 (91·5%)	2,797/3,026 (92·4%)
Yes	217/2,569 (8·4%)	228/3,026 (7·5%)
No data	1/2,569 (0·0%)	1/3,026 (0·0%)
VIOLATION: (d) respiratory system		
No	2,359/2,569 (91·8%)	2,757/3,026 (91·1%)
Yes	209/2,569 (8·1%)	268/3,026 (8·9%)
No data	1/2,569 (0·0%)	1/3,026 (0·0%)
VIOLATION: (d) ix digestive system		
No	2,453/2,569 (95·5%)	2,886/3,026 (95·4%)
Yes	115/2,569 (4·5%)	139/3,026 (4·6%)
No data	1/2,569 (0·0%)	1/3,026 (0·0%)
VIOLATION: (d) x genito-urinary system		
No	2,530/2,569 (98·5%)	2,945/3,026 (97·3%)
Yes	38/2,569 (1·5%)	80/3,026 (2·6%)
No data	1/2,569 (0·0%)	1/3,026 (0·0%)
VIOLATION: (d) xii skin complaints		
No	2,524/2,569 (98·2%)	2,970/3,026 (98·1%)
Yes	44/2,569 (1·7%)	55/3,026 (1·8%)
No data	1/2,569 (0·0%)	1/3,026 (0·0%)
VIOLATION: (d) xiii musculoskeletal system		
No	2,116/2,569 (82·4%)	2,540/3,026 (83·9%)
Yes	452/2,569 (17·6%)	485/3,026 (16·0%)
No data	1/2,569 (0·0%)	1/3,026 (0·0%)
VIOLATION: (d) other complaints		
No	2,564/2,569 (99·8%)	3,014/3,026 (99·6%)
Yes	4/2,569 (0·2%)	11/3,026 (0·4%)
No data	1/2,569 (0·0%)	1/3,026 (0·0%)
VIOLATION: (d) long standing illness		
No	1,550/2,569 (60·3%)	1,790/3,026 (59·2%)
Yes	1,018/2,569 (39·6%)	1,235/3,026 (40·8%)
No data	1/2,569 (0·0%)	1/3,026 (0·0%)

	Overall sample	
	**Male** N=2,569	**Female** N=3,026
VIOLATION: been an inpatient in hospital in last 12 months		
No	2,498/2,569 (97·2%)	2,889/3,026 (95·5%)
Yes	71/2,569 (2·8%)	137/3,026 (4·5%)
VIOLATION: (d) acute sickness last 2 weeks		
No	2,202/2,569 (85·7%)	2,477/3,026 (81·9%)
Yes	367/2,569 (14·3%)	548/3,026 (18·1%)
No data	0/2,569 (0·0%)	1/3,026 (0·0%)
VIOLATION: how many times fractured bones in the last 12 months		
No	2,559/2,569 (99·6%)	3,019/3,026 (99·8%)
Yes	10/2,569 (0·4%)	7/3,026 (0·2%)
VIOLATION: whether currently pregnant 16 plus		
No	2,569/2,569 (100·0%)	3,026/3,026 (100·0%)
VIOLATION: pregnant in the last 12months		
No	2,569/2,569 (100·0%)	2,982/3,026 (98·5%)
Yes	0/2,569 (0·0%)	43/3,026 (1·4%)
No data	0/2,569 (0·0%)	1/3,026 (0·0%)
VIOLATION: (d) whether taking medication - excluding contraceptives only		
No	1,681/2,569 (65·4%)	1,777/3,026 (58·7%)
Yes	888/2,569 (34·6%)	1,249/3,026 (41·3%)
VIOLATION: Do you currently inject insulin for diabetes?		
No	2,538/2,569 (98·8%)	3,005/3,026 (99·3%)
Yes	31/2,569 (1·2%)	21/3,026 (0·7%)
VIOLATION: Are you currently taking any medicines, tablets or pills		
No	2,504/2,569 (97·5%)	2,988/3,026 (98·7%)
Yes	65/2,569 (2·5%)	38/3,026 (1·3%)
VIOLATION: whether still on hrt		
No	2,569/2,569 (100·0%)	2,860/3,026 (94·5%)
Yes	0/2,569 (0·0%)	165/3,026 (5·5%)
No data	0/2,569 (0·0%)	1/3,026 (0·0%)
VIOLATION: (d) diuretics (blood pressure)		
No	2,487/2,569 (96·8%)	2,900/3,026 (95·8%)
Yes	82/2,569 (3·2%)	126/3,026 (4·2%)
VIOLATION: (d) beta blockers (blood pressure fibrinogen)		
No	2,460/2,569 (95·8%)	2,910/3,026 (96·2%)
Yes	109/2,569 (4·2%)	116/3,026 (3·8%)
VIOLATION: (d) ace inhibitors (blood pressure)		
No	2,385/2,569 (92·8%)	2,885/3,026 (95·3%)
Yes	184/2,569 (7·2%)	141/3,026 (4·7%)
VIOLATION: (d) calcium blockers (blood pressure)		
No	2,479/2,569 (96·5%)	2,949/3,026 (97·5%)
Yes	90/2,569 (3·5%)	77/3,026 (2·5%)
VIOLATION: (d) other drugs affecting bp		
No	2,541/2,569 (98·9%)	3,004/3,026 (99·3%)
Yes	28/2,569 (1·1%)	22/3,026 (0·7%)
VIOLATION: (d) lipid lowering (cholesterol fibrinogen)		
No	2,352/2,569 (91·6%)	2,887/3,026 (95·4%)
Yes	217/2,569 (8·4%)	139/3,026 (4·6%)
VIOLATION: (d) iron deficiency (haemoglobin ferritin)		
No	2,569/2,569 (100·0%)	3,026/3,026 (100·0%)
VIOLATION: (d) whether taking drugs affecting blood pressure		
No	2,268/2,569 (88·3%)	2,730/3,026 (90·2%)
Yes	301/2,569 (11·7%)	296/3,026 (9·8%)
VIOLATION: (d) whether taking drugs prescribed for blood pressure		
No	2,342/2,569 (91·2%)	2,785/3,026 (92·0%)
Yes	227/2,569 (8·8%)	241/3,026 (8·0%)
VIOLATION: are you taking statins (drugs to lower cholesterol)		
No	2,558/2,569 (99·6%)	3,013/3,026 (99·6%)
Yes	11/2,569 (0·4%)	13/3,026 (0·4%)
VIOLATION: (d) units drunk on heaviest day in last 7		
No	1,973/2,569 (76·8%)	2,717/3,026 (89·8%)
Yes	593/2,569 (23·1%)	307/3,026 (10·1%)
No data	3/2,569 (0·1%)	2/3,026 (0·1%)
VIOLATION: (d) cigarette smoking status – never/ ex-regular/ ex-occasional/ current		
No	1,956/2,569 (76·1%)	2,315/3,026 (76·5%)
Yes	613/2,569 (23·9%)	709/3,026 (23·4%)
No data	0/2,569 (0·0%)	2/3,026 (0·1%)

	Overall sample	
	**Male** N=2,569	**Female** N=3,026
VIOLATION: (d) number of cigarettes smoke a day - including non-smokers		
No	1,974/2,569 (76·8%)	2,334/3,026 (77·1%)
Yes	590/2,569 (23·0%)	689/3,026 (22·8%)
No data	5/2,569 (0·2%)	3/3,026 (0·1%)
VIOLATION: currently smokes cigarettes		
No	1,959/2,569 (76·3%)	2,332/3,026 (77·1%)
Yes	610/2,569 (23·7%)	694/3,026 (22·9%)
VIOLATION: currently smokes cigars		
No	2,522/2,569 (98·2%)	3,024/3,026 (99·9%)
Yes	47/2,569 (1·8%)	2/3,026 (0·1%)
VIOLATION: currently smokes pipe		
No	2,558/2,569 (99·6%)	3,026/3,026 (100·0%)
Yes	11/2,569 (0·4%)	0/3,026 (0·0%)
VIOLATION: Ferritin<15ug/L or Ferritin>150/200ug/L (F/M)		
No	2,007/2,569 (78·1%)	2,556/3,026 (84·5%)
Yes	562/2,569 (21·9%)	470/3,026 (15·5%)
VIOLATION: C-Reactive Protein>5mg/L		
No	2,269/2,569 (88·3%)	2,513/3,026 (83·0%)
Yes	300/2,569 (11·7%)	513/3,026 (17·0%)

HSE = Health Survey for England, BMI = Body Mass Index, F = Female, M = Male.
Data are presented as unweighted n/total (%) based on participants with non-missing haemoglobin, ferritin, and C-reactive protein.
No data: No answer/refused, Don't know, Not applicable, Not obtained. If a measurement of one or more of the healthy criteria is missing, then the participant is included in the reference sample.
[1] Males: ferritin≥15 and ≤200ug/L and C-reactive protein≤5mg/L; females: ferritin≥15 and ≤150ug/L and C-reactive protein≤5mg/L.

Table 4.1.11 England HSE 2009: Participant characteristics of violations (18-65 years).

	Overall sample	
	**Male N=804**	**Female N=892**
Reference sample[1]		
No	686/804 (85·3%)	750/892 (84·1%)
Yes	118/804 (14·7%)	142/892 (15·9%)
VIOLATION: BMI<18.5 or >30 kg/m^2		
No	557/804 (69·3%)	624/892 (70·0%)
Yes	193/804 (24·0%)	216/892 (24·2%)
No data	54/804 (6·7%)	52/892 (5·8%)
VIOLATION: whether has clotting disorder		
No	804/804 (100·0%)	892/892 (100·0%)
VIOLATION: (d) doctor diagnosed high blood pressure (excluding pregnant)		
No	634/804 (78·9%)	764/892 (85·7%)
Yes	170/804 (21·1%)	128/892 (14·3%)
VIOLATION: (d) doctor diagnosed diabetes (excluding pregnant)		
No	776/804 (96·5%)	871/892 (97·6%)
Yes	28/804 (3·5%)	21/892 (2·4%)
VIOLATION: (d) i infectious disease		
No	803/804 (99·9%)	891/892 (99·9%)
Yes	0/804 (0·0%)	1/892 (0·1%)
No data	1/804 (0·1%)	0/892 (0·0%)
VIOLATION: (d) ii neoplasms & benign growths		
No	795/804 (98·9%)	883/892 (99·0%)
Yes	8/804 (1·0%)	9/892 (1·0%)
No data	1/804 (0·1%)	0/892 (0·0%)
VIOLATION: (d) iii endocrine & metabolic		
No	760/804 (94·5%)	828/892 (92·8%)
Yes	43/804 (5·3%)	64/892 (7·2%)
No data	1/804 (0·1%)	0/892 (0·0%)
VIOLATION: (d) iv blood and related organs		
No	802/804 (99·8%)	885/892 (99·2%)
Yes	1/804 (0·1%)	7/892 (0·8%)
No data	1/804 (0·1%)	0/892 (0·0%)
VIOLATION: (d) v mental disorders		
No	777/804 (96·6%)	855/892 (95·9%)
Yes	26/804 (3·2%)	37/892 (4·1%)
No data	1/804 (0·1%)	0/892 (0·0%)
VIOLATION: (d) vi nervous system		
No	781/804 (97·1%)	848/892 (95·1%)
Yes	22/804 (2·7%)	44/892 (4·9%)
No data	1/804 (0·1%)	0/892 (0·0%)
VIOLATION: (d) vi eye complaints		
No	794/804 (98·8%)	885/892 (99·2%)
Yes	9/804 (1·1%)	7/892 (0·8%)
No data	1/804 (0·1%)	0/892 (0·0%)
VIOLATION: (d) vi ear complaints		
No	793/804 (98·6%)	881/892 (98·8%)
Yes	10/804 (1·2%)	11/892 (1·2%)
No data	1/804 (0·1%)	0/892 (0·0%)
VIOLATION: (d) vii heart and circulatory system		
No	720/804 (89·6%)	840/892 (94·2%)
Yes	83/804 (10·3%)	52/892 (5·8%)
No data	1/804 (0·1%)	0/892 (0·0%)
VIOLATION: (d) respiratory system		
No	755/804 (93·9%)	828/892 (92·8%)
Yes	48/804 (6·0%)	64/892 (7·2%)
No data	1/804 (0·1%)	0/892 (0·0%)
VIOLATION: (d) ix digestive system		
No	772/804 (96·0%)	849/892 (95·2%)
Yes	31/804 (3·9%)	43/892 (4·8%)
No data	1/804 (0·1%)	0/892 (0·0%)
VIOLATION: (d) x genito-urinary system		
No	794/804 (98·8%)	872/892 (97·8%)
Yes	9/804 (1·1%)	20/892 (2·2%)
No data	1/804 (0·1%)	0/892 (0·0%)
VIOLATION: (d) xii skin complaints		
No	790/804 (98·3%)	880/892 (98·7%)

	Overall sample	
	Male<br>N=804	Female<br>N=892
Yes	13/804 (1·6%)	12/892 (1·3%)
No data	1/804 (0·1%)	0/892 (0·0%)
VIOLATION: (d) xiii musculoskeletal system		
No	684/804 (85·1%)	767/892 (86·0%)
Yes	119/804 (14·8%)	125/892 (14·0%)
No data	1/804 (0·1%)	0/892 (0·0%)
VIOLATION: (d) other complaints		
No	800/804 (99·5%)	891/892 (99·9%)
Yes	3/804 (0·4%)	1/892 (0·1%)
No data	1/804 (0·1%)	0/892 (0·0%)
VIOLATION: (d) long standing illness		
No	509/804 (63·3%)	572/892 (64·1%)
Yes	294/804 (36·6%)	320/892 (35·9%)
No data	1/804 (0·1%)	0/892 (0·0%)
VIOLATION: (d) acute sickness last 2 weeks		
No	701/804 (87·2%)	754/892 (84·5%)
Yes	103/804 (12·8%)	138/892 (15·5%)
VIOLATION: (d) whether taking medication - excluding contraceptives only		
No	530/804 (65·9%)	511/892 (57·3%)
Yes	274/804 (34·1%)	381/892 (42·7%)
VIOLATION: Do you currently inject insulin for diabetes?		
No	796/804 (99·0%)	885/892 (99·2%)
Yes	8/804 (1·0%)	7/892 (0·8%)
VIOLATION: Are you currently taking any medicines, tablets or pills		
No	784/804 (97·5%)	878/892 (98·4%)
Yes	20/804 (2·5%)	14/892 (1·6%)
VIOLATION: (d) diuretics (blood pressure)		
No	773/804 (96·1%)	867/892 (97·2%)
Yes	31/804 (3·9%)	25/892 (2·8%)
VIOLATION: (d) beta blockers (blood pressure fibrinogen)		
No	777/804 (96·6%)	869/892 (97·4%)
Yes	27/804 (3·4%)	23/892 (2·6%)
VIOLATION: (d) ace inhibitors (blood pressure)		
No	730/804 (90·8%)	845/892 (94·7%)
Yes	74/804 (9·2%)	47/892 (5·3%)
VIOLATION: (d) calcium blockers (blood pressure)		
No	762/804 (94·8%)	881/892 (98·8%)
Yes	42/804 (5·2%)	11/892 (1·2%)
VIOLATION: (d) other drugs affecting bp		
No	795/804 (98·9%)	887/892 (99·4%)
Yes	9/804 (1·1%)	5/892 (0·6%)
VIOLATION: (d) lipid lowering (cholesterol fibrinogen)		
No	729/804 (90·7%)	847/892 (95·0%)
Yes	75/804 (9·3%)	45/892 (5·0%)
VIOLATION: (d) iron deficiency (haemoglobin ferritin)		
No	804/804 (100·0%)	892/892 (100·0%)
VIOLATION: (d) whether taking drugs affecting blood pressure		
No	689/804 (85·7%)	814/892 (91·3%)
Yes	115/804 (14·3%)	78/892 (8·7%)
VIOLATION: (d) whether taking drugs prescribed for blood pressure		
No	714/804 (88·8%)	827/892 (92·7%)
Yes	90/804 (11·2%)	65/892 (7·3%)
VIOLATION: are you taking statins (drugs to lower cholesterol)		
No	797/804 (99·1%)	885/892 (99·2%)
Yes	7/804 (0·9%)	7/892 (0·8%)
VIOLATION: (D) NEW Units drunk on heaviest day in last 7 (16yrs+)		
No	559/804 (69·5%)	734/892 (82·3%)
Yes	243/804 (30·2%)	154/892 (17·3%)
No data	2/804 (0·2%)	4/892 (0·4%)
VIOLATION: (d) cigarette smoking status – never/ ex-regular/ ex-occasional/ current		
No	628/804 (78·1%)	707/892 (79·3%)
Yes	176/804 (21·9%)	185/892 (20·7%)
VIOLATION: (d) number of cigarettes smoke a day - including non-smokers		
No	631/804 (78·5%)	709/892 (79·5%)
Yes	172/804 (21·4%)	183/892 (20·5%)
No data	1/804 (0·1%)	0/892 (0·0%)
VIOLATION: currently smokes cigarettes		

	Overall sample	
	**Male N=804**	**Female N=892**
No	627/804 (78·0%)	709/892 (79·5%)
Yes	177/804 (22·0%)	183/892 (20·5%)
VIOLATION: currently smokes cigars		
No	786/804 (97·8%)	892/892 (100·0%)
Yes	18/804 (2·2%)	0/892 (0·0%)
VIOLATION: currently smokes pipe		
No	803/804 (99·9%)	892/892 (100·0%)
Yes	1/804 (0·1%)	0/892 (0·0%)
VIOLATION: Ferritin<15ug/L or Ferritin>150/200ug/L (F/M)		
No	629/804 (78·2%)	745/892 (83·5%)
Yes	175/804 (21·8%)	147/892 (16·5%)
VIOLATION: C-Reactive Protein>5mg/L		
No	728/804 (90·5%)	734/892 (82·3%)
Yes	76/804 (9·5%)	158/892 (17·7%)
VIOLATION: eGFR<90 ml/min/1.73m^2		
No	606/804 (75·4%)	677/892 (75·9%)
Yes	163/804 (20·3%)	175/892 (19·6%)
No data	35/804 (4·4%)	40/892 (4·5%)

HSE = Health Survey for England, BMI = Body Mass Index, F = Female, M = Male.
Data are presented as unweighted n/total (%) based on participants with non-missing haemoglobin, ferritin, and C-reactive protein.
No data: No answer/refused, Don't know, Not applicable, Not obtained. If a measurement of one or more of the healthy criteria is missing, then the participant is included in the reference sample.
[1] Males: ferritin≥15 and ≤200ug/L and C-reactive protein≤5mg/L; females: ferritin≥15 and ≤150ug/L and C-reactive protein≤5mg/L.

Table 4.1.12 CHN CHNS 2009: Participant characteristics of violations (18-65 years).

	Overall sample	
	**Male N=3,352**	**Female N=3,820**
Reference sample[1]		
No	3,107/3,352 (92·7%)	3,254/3,820 (85·2%)
Yes	245/3,352 (7·3%)	566/3,820 (14·8%)
VIOLATION: BMI<18.5 or >30 kg/m^2		
No	2,967/3,352 (88·5%)	3,382/3,820 (88·5%)
Yes	298/3,352 (8·9%)	376/3,820 (9·8%)
Missing	87/3,352 (2·6%)	62/3,820 (1·6%)
VIOLATION: Illness/Injury - Infectious/parasitic disease		
No	342/3,352 (10·2%)	483/3,820 (12·6%)
Yes	2/3,352 (0·1%)	1/3,820 (0·0%)
Missing	3,008/3,352 (89·7%)	3,336/3,820 (87·3%)
VIOLATION: Illness/Injury - Heart disease		
No	331/3,352 (9·9%)	459/3,820 (12·0%)
Yes	13/3,352 (0·4%)	25/3,820 (0·7%)
Missing	3,008/3,352 (89·7%)	3,336/3,820 (87·3%)
VIOLATION: Illness/Injury - Tumor		
No	342/3,352 (10·2%)	482/3,820 (12·6%)
Yes	2/3,352 (0·1%)	2/3,820 (0·1%)
Missing	3,008/3,352 (89·7%)	3,336/3,820 (87·3%)
VIOLATION: Illness/Injury - Respiratory disease		
No	244/3,352 (7·3%)	355/3,820 (9·3%)
Yes	100/3,352 (3·0%)	129/3,820 (3·4%)
Missing	3,008/3,352 (89·7%)	3,336/3,820 (87·3%)
VIOLATION: Illness/Injury - Injury		
No	340/3,352 (10·1%)	477/3,820 (12·5%)
Yes	4/3,352 (0·1%)	7/3,820 (0·2%)
Missing	3,008/3,352 (89·7%)	3,336/3,820 (87·3%)
VIOLATION: Illness/Injury - Alcohol poisoning		
No	343/3,352 (10·2%)	484/3,820 (12·7%)
Yes	1/3,352 (0·0%)	0/3,820 (0·0%)
Missing	3,008/3,352 (89·7%)	3,336/3,820 (87·3%)
VIOLATION: Illness/Injury - Endocrine disorder		
No	337/3,352 (10·1%)	477/3,820 (12·5%)
Yes	7/3,352 (0·2%)	7/3,820 (0·2%)
Missing	3,008/3,352 (89·7%)	3,336/3,820 (87·3%)
VIOLATION: Illness/Injury - Hematological disease		
No	341/3,352 (10·2%)	479/3,820 (12·5%)
Yes	3/3,352 (0·1%)	5/3,820 (0·1%)
Missing	3,008/3,352 (89·7%)	3,336/3,820 (87·3%)
VIOLATION: Illness/Injury - Mental/psychiatric disorder		
No	342/3,352 (10·2%)	482/3,820 (12·6%)
Yes	2/3,352 (0·1%)	2/3,820 (0·1%)
Missing	3,008/3,352 (89·7%)	3,336/3,820 (87·3%)
VIOLATION: Illness/Injury - Neurological disorder		
No	330/3,352 (9·8%)	466/3,820 (12·2%)
Yes	14/3,352 (0·4%)	18/3,820 (0·5%)
Missing	3,008/3,352 (89·7%)	3,336/3,820 (87·3%)
VIOLATION: Illness/Injury - Eye/ear/nose/throat/teeth disease		
No	337/3,352 (10·1%)	469/3,820 (12·3%)
Yes	7/3,352 (0·2%)	15/3,820 (0·4%)
Missing	3,008/3,352 (89·7%)	3,336/3,820 (87·3%)
VIOLATION: Illness/Injury - Digestive disease		
No	296/3,352 (8·8%)	440/3,820 (11·5%)
Yes	48/3,352 (1·4%)	44/3,820 (1·2%)
Missing	3,008/3,352 (89·7%)	3,336/3,820 (87·3%)
VIOLATION: Illness/Injury - Urinary disease		
No	335/3,352 (10·0%)	476/3,820 (12·5%)
Yes	9/3,352 (0·3%)	8/3,820 (0·2%)
Missing	3,008/3,352 (89·7%)	3,336/3,820 (87·3%)
VIOLATION: Illness/Injury - Sexual dysfunction		
No	343/3,352 (10·2%)	484/3,820 (12·7%)
Yes	1/3,352 (0·0%)	0/3,820 (0·0%)
Missing	3,008/3,352 (89·7%)	3,336/3,820 (87·3%)
VIOLATION: Illness/Injury - Obstetrical/gynecological disease		
No	344/3,352 (10·3%)	466/3,820 (12·2%)

	Overall sample	
	Male N=3,352	Female N=3,820
Yes	0/3,352 (0·0%)	18/3,820 (0·5%)
Missing	3,008/3,352 (89·7%)	3,336/3,820 (87·3%)
VIOLATION: Illness/Injury - Dermatological disease		
No	336/3,352 (10·0%)	477/3,820 (12·5%)
Yes	8/3,352 (0·2%)	7/3,820 (0·2%)
Missing	3,008/3,352 (89·7%)	3,336/3,820 (87·3%)
VIOLATION: Illness/Injury - Muscular/rheumatological disease		
No	328/3,352 (9·8%)	461/3,820 (12·1%)
Yes	16/3,352 (0·5%)	23/3,820 (0·6%)
Missing	3,008/3,352 (89·7%)	3,336/3,820 (87·3%)
VIOLATION: Diagnosed with high blood pressure		
No	3,013/3,352 (89·9%)	3,422/3,820 (89·6%)
Yes	317/3,352 (9·5%)	381/3,820 (10·0%)
No data	9/3,352 (0·3%)	7/3,820 (0·2%)
Missing	13/3,352 (0·4%)	10/3,820 (0·3%)
VIOLATION: Diagnosed with diabetes		
No	3,244/3,352 (96·8%)	3,735/3,820 (97·8%)
Yes	90/3,352 (2·7%)	67/3,820 (1·8%)
No data	5/3,352 (0·1%)	8/3,820 (0·2%)
Missing	13/3,352 (0·4%)	10/3,820 (0·3%)
VIOLATION: Diagnosed with myocardial infarction		
No	3,319/3,352 (99·0%)	3,777/3,820 (98·9%)
Yes	19/3,352 (0·6%)	26/3,820 (0·7%)
No data	1/3,352 (0·0%)	5/3,820 (0·1%)
Missing	13/3,352 (0·4%)	12/3,820 (0·3%)
VIOLATION: Diagnosed with apoplexy		
No	3,301/3,352 (98·5%)	3,786/3,820 (99·1%)
Yes	37/3,352 (1·1%)	18/3,820 (0·5%)
No data	1/3,352 (0·0%)	4/3,820 (0·1%)
Missing	13/3,352 (0·4%)	12/3,820 (0·3%)
VIOLATION: Dr. told you that you suffer from asthma		
No	3,306/3,352 (98·6%)	3,780/3,820 (99·0%)
Yes	30/3,352 (0·9%)	26/3,820 (0·7%)
No data	1/3,352 (0·0%)	2/3,820 (0·1%)
Missing	15/3,352 (0·4%)	12/3,820 (0·3%)
VIOLATION: Past 12 months - had whistling/wheezing in chest		
No	9/3,352 (0·3%)	10/3,820 (0·3%)
Yes	20/3,352 (0·6%)	15/3,820 (0·4%)
No data	0/3,352 (0·0%)	1/3,820 (0·0%)
Missing	3,323/3,352 (99·1%)	3,794/3,820 (99·3%)
VIOLATION: Last 4 weeks - been sick or injured		
No	2,932/3,352 (87·5%)	3,260/3,820 (85·3%)
Yes	400/3,352 (11·9%)	543/3,820 (14·2%)
No data	1/3,352 (0·0%)	1/3,820 (0·0%)
Missing	19/3,352 (0·6%)	16/3,820 (0·4%)
VIOLATION: Last 4 weeks - fever, sore throat, cough		
No	3,102/3,352 (92·5%)	3,534/3,820 (92·5%)
Yes	234/3,352 (7·0%)	270/3,820 (7·1%)
No data	0/3,352 (0·0%)	1/3,820 (0·0%)
Missing	16/3,352 (0·5%)	15/3,820 (0·4%)
VIOLATION: Last 4 weeks - diarrhea, stomachache		
No	3,285/3,352 (98·0%)	3,744/3,820 (98·0%)
Yes	50/3,352 (1·5%)	58/3,820 (1·5%)
No data	0/3,352 (0·0%)	1/3,820 (0·0%)
Missing	17/3,352 (0·5%)	17/3,820 (0·4%)
VIOLATION: Last 4 weeks - stomachache		
No	3,259/3,352 (97·2%)	3,712/3,820 (97·2%)
Yes	79/3,352 (2·4%)	95/3,820 (2·5%)
No data	1/3,352 (0·0%)	3/3,820 (0·1%)
Missing	13/3,352 (0·4%)	10/3,820 (0·3%)
VIOLATION: Last 4 weeks - asthma		
No	3,324/3,352 (99·2%)	3,788/3,820 (99·2%)
Yes	14/3,352 (0·4%)	20/3,820 (0·5%)
No data	1/3,352 (0·0%)	2/3,820 (0·1%)
Missing	13/3,352 (0·4%)	10/3,820 (0·3%)
VIOLATION: Last 4 weeks - headache, dizziness		
No	3,245/3,352 (96·8%)	3,588/3,820 (93·9%)

	Overall sample	
	Male<br>N=3,352	Female<br>N=3,820
Yes	91/3,352 (2·7%)	217/3,820 (5·7%)
No data	1/3,352 (0·0%)	3/3,820 (0·1%)
Missing	15/3,352 (0·4%)	12/3,820 (0·3%)
VIOLATION: Last 4 weeks - joint, muscle pain		
No	3,239/3,352 (96·6%)	3,603/3,820 (94·3%)
Yes	97/3,352 (2·9%)	203/3,820 (5·3%)
No data	1/3,352 (0·0%)	3/3,820 (0·1%)
Missing	15/3,352 (0·4%)	11/3,820 (0·3%)
VIOLATION: Last 4 weeks - rash, dermatitis		
No	3,319/3,352 (99·0%)	3,789/3,820 (99·2%)
Yes	16/3,352 (0·5%)	19/3,820 (0·5%)
No data	1/3,352 (0·0%)	1/3,820 (0·0%)
Missing	16/3,352 (0·5%)	11/3,820 (0·3%)
VIOLATION: Last 4 weeks - eye/ear disease		
No	3,322/3,352 (99·1%)	3,785/3,820 (99·1%)
Yes	13/3,352 (0·4%)	23/3,820 (0·6%)
No data	1/3,352 (0·0%)	1/3,820 (0·0%)
Missing	16/3,352 (0·5%)	11/3,820 (0·3%)
VIOLATION: Last 4 weeks - heart disease/chest pain		
No	3,309/3,352 (98·7%)	3,757/3,820 (98·4%)
Yes	26/3,352 (0·8%)	51/3,820 (1·3%)
No data	1/3,352 (0·0%)	1/3,820 (0·0%)
Missing	16/3,352 (0·5%)	11/3,820 (0·3%)
VIOLATION: Last 4 weeks - other infectious disease		
No	3,284/3,352 (98·0%)	3,752/3,820 (98·2%)
Yes	41/3,352 (1·2%)	41/3,820 (1·1%)
No data	2/3,352 (0·1%)	6/3,820 (0·2%)
Missing	25/3,352 (0·7%)	21/3,820 (0·5%)
VIOLATION: Last 4 weeks - noncommunicable disease		
No	3,196/3,352 (95·3%)	3,637/3,820 (95·2%)
Yes	129/3,352 (3·8%)	155/3,820 (4·1%)
No data	2/3,352 (0·1%)	7/3,820 (0·2%)
Missing	25/3,352 (0·7%)	21/3,820 (0·5%)
VIOLATION: Last 4 weeks - days hospitalised		
Yes	31/3,352 (0·9%)	27/3,820 (0·7%)
Missing	3,321/3,352 (99·1%)	3,793/3,820 (99·3%)
VIOLATION: Currently pregnant or don't know		
No	0/3,352 (0·0%)	2,124/3,820 (55·6%)
Yes	0/3,352 (0·0%)	64/3,820 (1·7%)
Missing	3,352/3,352 (100·0%)	1,632/3,820 (42·7%)
VIOLATION: # of months pregnant		
Yes	0/3,352 (0·0%)	62/3,820 (1·6%)
Missing	3,352/3,352 (100·0%)	3,758/3,820 (98·4%)
VIOLATION: Taking anti-hypertension drugs		
No	88/3,352 (2·6%)	80/3,820 (2·1%)
Yes	228/3,352 (6·8%)	300/3,820 (7·9%)
No data	0/3,352 (0·0%)	1/3,820 (0·0%)
Missing	3,036/3,352 (90·6%)	3,439/3,820 (90·0%)
VIOLATION: Province – Guizhou – Altitude		
No	3,073/3,352 (91·7%)	3,506/3,820 (91·8%)
Yes	279/3,352 (8·3%)	314/3,820 (8·2%)
VIOLATION: still smokes cigarettes		
No	172/3,352 (5·1%)	12/3,820 (0·3%)
Yes	1,909/3,352 (57·0%)	109/3,820 (2·9%)
No data	1,271/3,352 (37·9%)	3,699/3,820 (96·8%)
VIOLATION: Alcohol consumption almost every day		
No	1,521/3,352 (45·4%)	317/3,820 (8·3%)
Yes	605/3,352 (18·0%)	34/3,820 (0·9%)
Missing	1,226/3,352 (36·6%)	3,469/3,820 (90·8%)
VIOLATION (# beer bottles per week): >6 for women and >8 for men		
No	1,362/3,352 (40·6%)	210/3,820 (5·5%)
Yes	107/3,352 (3·2%)	9/3,820 (0·2%)
Missing	1,883/3,352 (56·2%)	3,601/3,820 (94·3%)
VIOLATION (# wine drinks per week): >6 for women and >8 for men		
No	224/3,352 (6·7%)	105/3,820 (2·7%)
Yes	56/3,352 (1·7%)	11/3,820 (0·3%)
Missing	3,072/3,352 (91·6%)	3,704/3,820 (97·0%)

	Overall sample	
	**Male** **N=3,352**	**Female** **N=3,820**
VIOLATION (# liquor drinks per week): >6 for women and >8 for men		
No	1,075/3,352 (32·1%)	134/3,820 (3·5%)
Yes	561/3,352 (16·7%)	36/3,820 (0·9%)
Missing	1,716/3,352 (51·2%)	3,650/3,820 (95·5%)
VIOLATION (# beer, wine, liquor): >6 for women and >8 for men		
No	1,357/3,352 (40·5%)	285/3,820 (7·5%)
Yes	759/3,352 (22·6%)	64/3,820 (1·7%)
Missing	1,236/3,352 (36·9%)	3,471/3,820 (90·9%)
VIOLATION: Ferritin<15ug/L or Ferritin>150/200ug/L (F/M)		
No	2,343/3,352 (69·9%)	2,808/3,820 (73·5%)
Yes	1,009/3,352 (30·1%)	1,012/3,820 (26·5%)
VIOLATION: C-Reactive Protein>5mg/L		
No	3,058/3,352 (91·2%)	3,538/3,820 (92·6%)
Yes	294/3,352 (8·8%)	282/3,820 (7·4%)
VIOLATION: eGFR<90 ml/min/1.73m^2		
No	1,570/3,352 (46·8%)	1,281/3,820 (33·5%)
Yes	1,782/3,352 (53·2%)	2,538/3,820 (66·4%)
Missing	0/3,352 (0·0%)	1/3,820 (0·0%)

CHN = China, CHNS = China Health and Nutrition Survey, BMI = Body Mass Index, F = Female, M = Male.
Data are presented as unweighted n/total (%) based on participants with non-missing haemoglobin, ferritin, and C-reactive protein.
No data: No answer/refused, Don't know; Missing: Not applicable, Not asked. If a measurement of one or more of the healthy criteria is missing, then the participant is included in the reference sample.
[1] Males: ferritin≥15 and ≤200ug/L and C-reactive protein≤5mg/L; females: ferritin≥15 and ≤150ug/L and C-reactive protein≤5mg/L.

Table 4.1.13 AUS NHS 2011-2012: Participant characteristics of violations (18-65 years).

	Overall sample	
	**Male N=1,912**	**Female N=2,480**
Reference sample[1]		
No	1,699/1,912 (88·9%)	2,198/2,480 (88·6%)
Yes	213/1,912 (11·1%)	282/2,480 (11·4%)
VIOLATION: BMI<18.5 or >30 kg/m^2		
No	1,282/1,912 (67·1%)	1,626/2,480 (65·6%)
Yes	577/1,912 (30·2%)	678/2,480 (27·3%)
Missing	53/1,912 (2·8%)	176/2,480 (7·1%)
VIOLATION: Co-morbidities		
No	1,143/1,912 (59·8%)	1,338/2,480 (54·0%)
Yes	769/1,912 (40·2%)	1,142/2,480 (46·0%)
VIOLATION: Dialysis		
No	Not displayed*	Not displayed*
Yes	<10/1,912 (<0·5%)	<10/2,480(<0·4%)
VIOLATION: Hospital last 12 months		
No	Not displayed*	2,460/2,480 (99·2%)
Yes	<10/1,912 (<0·5%)	20/2,480 (0·8%)
VIOLATION: Hospital last 2 weeks		
No	1,902/1,912 (99·5%)	2,457/2,480 (99·1%)
Yes	10/1,912 (0·5%)	23/2,480 (0·9%)
VIOLATION: Time off work or study due to illness		
No	1,445/1,912 (75·6%)	1,592/2,480 (64·2%)
Yes	162/1,912 (8·5%)	257/2,480 (10·4%)
Missing	305/1,912 (16·0%)	631/2,480 (25·4%)
VIOLATION: Time off work due to illness		
No	1,411/1,912 (73·8%)	1,540/2,480 (62·1%)
Yes	151/1,912 (7·9%)	233/2,480 (9·4%)
Missing	350/1,912 (18·3%)	707/2,480 (28·5%)
VIOLATION: Time off study due to illness		
No	181/1,912 (9·5%)	306/2,480 (12·3%)
Yes	15/1,912 (0·8%)	30/2,480 (1·2%)
Missing	1,716/1,912 (89·7%)	2,144/2,480 (86·5%)
VIOLATION: Medication		
No	969/1,912 (50·7%)	1,101/2,480 (44·4%)
Yes	943/1,912 (49·3%)	1,379/2,480 (55·6%)
VIOLATION: Breastfeeding		
No	Not applicable	2,388/2,480 (97·5%)
Yes	Not applicable	62/2,480 (2·5%)
VIOLATION: Pregnancy		
No	Not applicable	2,388/2,480 (98·7%)
Yes	Not applicable	32/2,480 (1·3%)
VIOLATION: Smoking		
No	1,615/1,912 (84·5%)	2,103/2,480 (84·8%)
Yes	297/1,912 (15·5%)	377/2,480 (15·2%)
VIOLATION: Alcohol		
No	1,365/1,912 (71·4%)	1,388/2,480 (56·0%)
Yes	12/1,912 (0·6%)	44/2,480 (1·8%)
Missing	535/1,912 (28·0%)	1,048/2,480 (42·3%)
VIOLATION: Ferritin<15ug/L or Ferritin>150/200ug/L (F/M)		
No	1,072/1,912 (56·1%)	1,933/2,480 (77·9%)
Yes	840/1,912 (43·9%)	547/2,480 (22·1%)
VIOLATION: C-Reactive Protein>5mg/L		
No	1,713/1,912 (89·6%)	2,010/2,480 (81·0%)
Yes	199/1,912 (10·4%)	470/2,480 (19·0%)
VIOLATION: eGFR<90 ml/min/1.73m^2		
No	1,416/1,910 (74·1%)	1,919/2,478 (77·4%)
Yes	494/1,910 (25·8%)	559/2,478 (22·5%)

AUS = Australia, NHS = National Health Survey, BMI = Body Mass Index, F = Female, M = Male.
Data are presented as unweighted n/total (%) based on participants with non-missing haemoglobin, ferritin, and C-reactive protein.
If a measurement of one or more of the healthy criteria is missing, then the participant is included in the reference sample.
[*] Not displayed due to Australian Bureau of Statistics requirements for output clearance.

[1] Males: ferritin$\geq$15 and $\leq$200ug/L and C-reactive protein$\leq$5mg/L; females: ferritin$\geq$15 and $\leq$150ug/L and C-reactive protein$\leq$5mg/L.

Table 4.1.14 AUS NNPAS 2011-2012: Participant characteristics of violations (18-65 years).

	Overall sample	
	**Male N=1,277**	**Female N=1,593**
Reference sample[1]		
No	983/1,277 (77·0%)	1,114/1,593 (69·9%)
Yes	294/1,277 (23·0%)	479/1,593 (30·1%)
VIOLATION: BMI<18.5 or >30 kg/m^2		
No	889/1,277 (69·6%)	1,053/1,593 (66·1%)
Yes	345/1,277 (27·0%)	445/1,593 (27·9%)
Missing	43/1,277 (3·4%)	95/1,593 (6·0%)
VIOLATION: Co-morbidities		
No	1,084/1,277 (84·9%)	1,367/1,593 (85·8%)
Yes	193/1,277 (15·1%)	226/1,593 (14·2%)
VIOLATION: Breastfeeding		
No	Not applicable	1,552/1,593 (97·4%)
Yes	Not applicable	41/1,593 (2·6%)
VIOLATION: Pregnancy		
No	Not applicable	1,571/1,593 (98·6%)
Yes	Not applicable	22/1,593 (1·4%)
VIOLATION: Smoking		
No	1,059/1,277 (82·9%)	1,371/1,593 (86·1%)
Yes	218/1,277 (17·1%)	222/1,593 (13·9%)
VIOLATION: Ferritin<15ug/L or Ferritin>150/200ug/L (F/M)		
No	739/1,277 (57·9%)	1,253/1,593 (78·7%)
Yes	538/1,277 (42·1%)	340/1,593 (21·3%)
VIOLATION: C-Reactive Protein>5mg/L		
No	1,145/1,277 (89·7%)	1,288/1,593 (80·9%)
Yes	132/1,277 (10·3%)	305/1,593 (19·1%)
VIOLATION: eGFR<90 ml/min/1.73m^2		
No	877/1,276 (68·7%)	1,216/1,593 (76·3%)
Yes	399/1,276 (31·2%)	377/1,593 (23·7%)

AUS = Australia, NNPAS = National Nutrition and Physical Activity Survey, BMI = Body Mass Index, F = Female, M = Male.

Data are presented as unweighted n/total (%) based on participants with non-missing haemoglobin, ferritin, and C-reactive protein.

If a measurement of one or more of the healthy criteria is missing, then the participant is included in the reference sample.

[1] Males: ferritin≥15 and ≤200ug/L and C-reactive protein≤5mg/L; females: ferritin≥15 and ≤150ug/L and C-reactive protein≤5mg/L.

4.2 Detailed Individual Exclusions Children (6-23 months)

Figure 4.2 Summary of exclusions of special interest from overall sample in children (6-23 months).

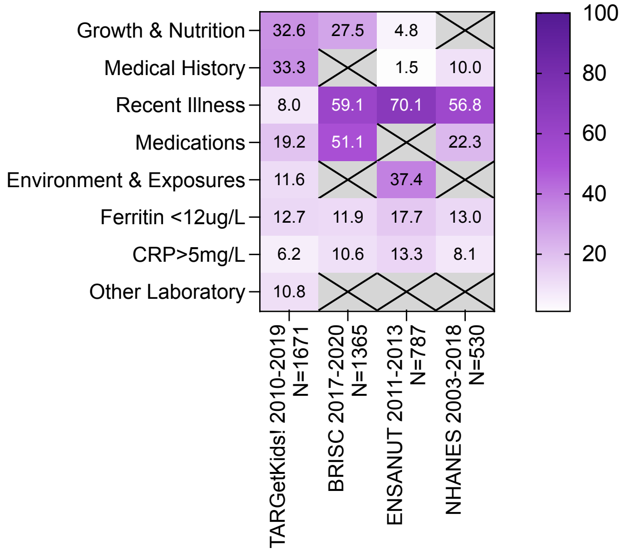

This represents the percentage of the total data set in each survey excluded by individual or grouped criteria. Data sources consisted of TARGet Kids!, BRISC, ENSANUT, and NHANES. CRP=C-Reactive Protein; eGFR=estimated Glomerular Filtration Rate.

Table 4.2.1 CAN TARGet Kids: Participant characteristics of violations (6-23 months).

	**Overall sample Male and Female N=1,671**
Reference sample[1]	
No	1,248/1,671 (74·7%)
Yes	423/1,671 (25·3%)
VIOLATION: Low birth weight (<2.5 kg) if age ≤24 months	
No	1,377/1,671 (82·4%)
Yes	209/1,671 (12·5%)
Missing	85/1,671 (5·1%)
VIOLATION: Weight z-score <-2 or >+2	
No	1,558/1,671 (93·2%)
Yes	85/1,671 (5·1%)
Missing	28/1,671 (1·7%)
VIOLATION: Length/height z-score <-2 or >+2	
No	1,421/1,671 (85·0%)
Yes	212/1,671 (12·7%)
Missing	38/1,671 (2·3%)
VIOLATION: Weight for length z-score <-2 or >+2	
No	1,521/1,671 (91·0%)
Yes	109/1,671 (6·5%)
Missing	41/1,671 (2·5%)
VIOLATION: BMI z-score <-2 or >+2	
No	1,507/1,671 (90·2%)
Yes	123/1,671 (7·4%)
Missing	41/1,671 (2·5%)
VIOLATION: Aged ≤12 months and >6 months exclusively breastfed	
No	446/1,671 (26·7%)
Yes	64/1,671 (3·8%)
Missing	1,161/1,671 (69·5%)
VIOLATION: Anaemia (mother)	
No	1,449/1,671 (86·7%)
Yes	110/1,671 (6·6%)
Missing	112/1,671 (6·7%)
VIOLATION: Birth (<37 weeks or > 42 weeks) if age ≤24 months	
No	1,263/1,671 (75·6%)
Yes	219/1,671 (13·1%)
Missing	189/1,671 (11·3%)
VIOLATION: Nursery if < 24 months	
No	433/1,671 (25·9%)
Yes	107/1,671 (6·4%)
Missing	1,131/1,671 (67·7%)
VIOLATION: Allergies	
No	1,076/1,671 (64·4%)
Yes	53/1,671 (3·2%)
Missing	542/1,671 (32·4%)
VIOLATION: Asthma	
No	1,414/1,671 (84·6%)
Yes	27/1,671 (1·6%)
Missing	230/1,671 (13·8%)
VIOLATION: Austism/PDD	
No	1,434/1,671 (85·8%)
Yes	3/1,671 (0·2%)
Missing	234/1,671 (14·0%)
VIOLATION: Development delay	
No	1,106/1,671 (66·2%)
Yes	13/1,671 (0·8%)
Missing	552/1,671 (33·0%)
VIOLATION: Diabetes	
No	1,434/1,671 (85·8%)
Yes	3/1,671 (0·2%)
Missing	234/1,671 (14·0%)
VIOLATION: At least one diagnosis	
No	1,172/1,671 (70·1%)
Yes	234/1,671 (14·0%)
Missing	265/1,671 (15·9%)
VIOLATION: Overweight	
No	1,114/1,671 (66·7%)

	Overall sample<br>Male and Female<br>N=1,671
Yes	3/1,671 (0·2%)
Missing	554/1,671 (33·2%)
VIOLATION: IBD	
No	333/1,671 (19·9%)
Missing	1,338/1,671 (80·1%)
VIOLATION: Cancer	
No	543/1,671 (32·5%)
Yes	1/1,671 (0·1%)
Missing	1,127/1,671 (67·4%)
VIOLATION: Other diagnosis	
No	1,138/1,671 (68·1%)
Yes	62/1,671 (3·7%)
Missing	471/1,671 (28·2%)
VIOLATION: Ill past month	
No	125/1,671 (7·5%)
Yes	134/1,671 (8·0%)
Missing	1,412/1,671 (84·5%)
VIOLATION: Regular prescribed medicine	
No	1,511/1,671 (90·4%)
Yes	68/1,671 (4·1%)
Missing	92/1,671 (5·5%)
VIOLATION: Prescribed medicine past 2 weeks	
No	301/1,671 (18·0%)
Yes	25/1,671 (1·5%)
Missing	1,345/1,671 (80·5%)
VIOLATION: Cold or flu medicine past month	
No	119/1,671 (7·1%)
Yes	46/1,671 (2·8%)
Missing	1,506/1,671 (90·1%)
VIOLATION: Other medicine past month	
No	97/1,671 (5·8%)
Yes	99/1,671 (5·9%)
Missing	1,475/1,671 (88·3%)
VIOLATION: Cold or flu medicine past 2 weeks	
No	277/1,671 (16·6%)
Yes	44/1,671 (2·6%)
Missing	1,350/1,671 (80·8%)
VIOLATION: Other medicine past 2 weeks	
No	273/1,671 (16·3%)
Yes	48/1,671 (2·9%)
Missing	1,350/1,671 (80·8%)
VIOLATION: Iron (e.g., Ferinsol, Palafer) intake	
No	973/1,671 (58·2%)
Yes	28/1,671 (1·7%)
Missing	670/1,671 (40·1%)
VIOLATION: Other iron intake	
No	328/1,671 (19·6%)
Yes	1/1,671 (0·1%)
Missing	1,342/1,671 (80·3%)
VIOLATION: Traditional remedies or cosmetics last 3 months	
No	246/1,671 (14·7%)
Yes	12/1,671 (0·7%)
Missing	1,413/1,671 (84·6%)
VIOLATION: Lead (household)	
No	232/1,671 (13·9%)
Yes	25/1,671 (1·5%)
Missing	1,414/1,671 (84·6%)
VIOLATION: Smoking (household)	
No	1,402/1,671 (83·9%)
Yes	178/1,671 (10·7%)
Missing	91/1,671 (5·4%)
VIOLATION: Ferritin<12ug/L	
No	1,459/1,671 (87·3%)
Yes	212/1,671 (12·7%)
VIOLATION: C-Reactive Protein>5mg/L	
No	1,568/1,671 (93·8%)
Yes	103/1,671 (6·2%)

	Overall sample<br>Male and Female<br>N=1,671
VIOLATION: Albumin<32g/L	
No	1,630/1,671 (97·5%)
Missing	41/1,671 (2·5%)
VIOLATION: Alkaline Phosphatase (age and sex dependent)	
No	1,533/1,671 (91·7%)
Yes	96/1,671 (5·7%)
Missing	42/1,671 (2·5%)
VIOLATION: ALT serum≥35U/L	
No	1,582/1,671 (94·7%)
Yes	88/1,671 (5·3%)
Missing	1/1,671 (0·1%)

CAN = Canada, TARGet Kids! = The Applied Research Group for Kids, BMI = Body Mass Index.
Data are presented as n/total (%) based on participants with non-missing haemoglobin, ferritin, and C-reactive protein.
Missing: No answer. If a measurement of one or more of the healthy criteria is missing, then the participant is included in the reference sample.
[1] Ferritin≥12ug/L and C-reactive protein≤5mg/L.

Table 4.2.2 BAN BRISC 2017-2020: Participant characteristics of violations (6-23 months).

	**Overall sample Male and Female N=1,365**
Reference sample[1]	
No	1,110/1,365 (81·3%)
Yes	255/1,365 (18·7%)
VIOLATION: Weight z-score <-2 or >+2	
No	1,235/1,365 (90·5%)
Yes	127/1,365 (9·3%)
Missing	3/1,365 (0·2%)
VIOLATION: Length/height z-score <-2 or >+2	
No	1,053/1,365 (77·1%)
Yes	309/1,365 (22·6%)
Missing	3/1,365 (0·2%)
VIOLATION: Weight for length z-score <-2 or >+2	
No	1,300/1,365 (95·2%)
Yes	62/1,365 (4·5%)
Missing	3/1,365 (0·2%)
VIOLATION: Any meds in the 4 weeks before midline visit	
No	806/1,365 (59·0%)
Yes	545/1,365 (39·9%)
Missing	14/1,365 (1·0%)
VIOLATION: Compliance<70% [Iron and MNP specific]	
No	1,120/1,365 (82·1%)
Yes	245/1,365 (17·9%)
VIOLATION: Any medical visit in the 4 weeks before midline visit	
No	783/1,365 (57·4%)
Yes	567/1,365 (41·5%)
Missing	15/1,365 (1·1%)
VIOLATION: Any infection in the 4 weeks before midline visit	
No	594/1,365 (43·5%)
Yes	759/1,365 (55·6%)
Missing	12/1,365 (0·9%)
VIOLATION: Ferritin<12ug/L	
No	1,203/1,365 (88·1%)
Yes	162/1,365 (11·9%)
VIOLATION: C-Reactive Protein>5mg/L	
No	1,220/1,365 (89·4%)
Yes	145/1,365 (10·6%)

BAN = Bangladesh, BRISC = Benefits and Risks of Iron intervention in Children.
Data are presented as n/total (%) based on participants with non-missing haemoglobin, ferritin, and C-reactive protein.
Missing: No data. If a measurement of one or more of the healthy criteria is missing, then the participant is included in the reference sample.
[1] Ferritin≥12ug/L and C-reactive protein≤5mg/L.

Table 4.2.3 ECU ENSANUT 2011-2013: Participant characteristics of violations (6-23 months).

	**Overall sample Male and Female N=787**
Reference sample[1]	95/787 (12·1%)
VIOLATION: Altitude > 750 meters	
No	493/787 (62·6%)
Yes	294/787 (37·4%)
VIOLATION: Weighed less than 5.5 pounds or 2.5 kilograms	
No	55/787 (7·0%)
Yes	13/787 (1·7%)
No data	124/787 (15·8%)
Missing	595/787 (75·6%)
VIOLATION: Birthweight less than 2500 grams	
No	445/787 (56·5%)
Yes	25/787 (3·2%)
Missing	317/787 (40·3%)
VIOLATION: Born ≥5 weeks early or late	
No	90/787 (11·4%)
Yes	12/787 (1·5%)
No data	5/787 (0·6%)
Missing	680/787 (86·4%)
VIOLATION: Health problems in the last 30 days	
No	265/787 (33·7%)
Yes	522/787 (66·3%)
VIOLATION: Hospitalisation in the last 30 days	
No	732/787 (93·0%)
Yes	55/787 (7·0%)
VIOLATION: Diarrhea in the last 2 weeks	
No	614/787 (78·0%)
Yes	163/787 (20·7%)
No data	1/787 (0·1%)
Missing	9/787 (1·1%)
VIOLATION: Cough, runny nose in the last 2 weeks	
No	424/787 (53·9%)
Yes	354/787 (45·0%)
Missing	9/787 (1·1%)
VIOLATION: Ferritin<12ug/L	
No	648/787 (82·3%)
Yes	139/787 (17·7%)
VIOLATION: C-Reactive Protein>5mg/L	
No	682/787 (86·7%)
Yes	105/787 (13·3%)

ECU = Ecuador, ENSANUT = Encuesta National de Salud y Nutricion.
Data are presented as unweighted n/total (%) based on participants with non-missing haemoglobin, ferritin, and C-reactive protein.
No data: No answer/refused, Don't know; Missing: Not applicable, Not asked. If a measurement of one or more of the healthy criteria is missing, then the participant is included in the reference sample.
[1] Ferritin≥12ug/L and C-reactive protein≤5mg/L.

Table 4.2.4 US NHANES 2003-2018: Participant characteristics of violations (12-23 months).

	**Overall sample Male and Female N=530**
Reference sample[1]	151/530 (28·5%)
VIOLATION: Doctor told you have diabetes	
No	529/530 (99·8%)
Yes	1/530 (0·2%)
VIOLATION: Still have asthma	
No	3/530 (0·6%)
Yes	38/530 (7·2%)
No data	1/530 (0·2%)
Missing	488/530 (92·1%)
VIOLATION: Taking treatment for anaemia/past 3 months	
No	515/530 (97·2%)
Yes	15/530 (2·8%)
VIOLATION: Overnight hospital patient in last year	
No	470/530 (88·7%)
Yes	60/530 (11·3%)
VIOLATION: SP have head cold or chest cold?	
No	290/530 (54·7%)
Yes	215/530 (40·6%)
No data	25/530 (4·7%)
VIOLATION: SP have stomach or intestinal illness?	
No	416/530 (78·5%)
Yes	87/530 (16·4%)
No data	27/530 (5·1%)
VIOLATION: SP have flu, pneumonia, ear infection?	
No	447/530 (84·3%)
Yes	54/530 (10·2%)
No data	29/530 (5·5%)
VIOLATION: Taken prescription medicine, past month	
No	414/530 (78·1%)
Yes	116/530 (21·9%)
VIOLATION: For anaemia, such as low iron	
No	97/530 (18·3%)
Yes	4/530 (0·8%)
No data	117/530 (22·1%)
Missing	312/530 (58·9%)
VIOLATION: Ferritin<12ug/L	
No	461/530 (87·0%)
Yes	69/530 (13·0%)
VIOLATION: C-Reactive Protein>5mg/L	
No	487/530 (91·9%)
Yes	43/530 (8·1%)

US = United States, NHANES = National Health and Nutrition Examination Survey, SP = Survey Participant. Data are presented as unweighted n/total (%) based on participants with non-missing haemoglobin, ferritin, and C-reactive protein. Data was combined from 1999-2018. No ferritin was measured in participants in survey years 1999-2000, 2001-2002, 2007-2008, 2009-2010, 2011-2012, 2013-2014. VIOLATION: For anaemia, such as low iron - only asked in survey years 2015-2016 and 2017-2018.

No data: No answer/refused, Don't know; Missing: Not applicable, Not asked. If a measurement of one or more of the healthy criteria is missing, then the participant is included in the reference sample.

[1] Ferritin≥12ug/L and C-reactive protein≤5mg/L.

4.3 Detailed Individual Exclusions Children (24-59 months)

Figure 4.3 Summary of exclusions of special interest from overall sample in children (24-59 months).

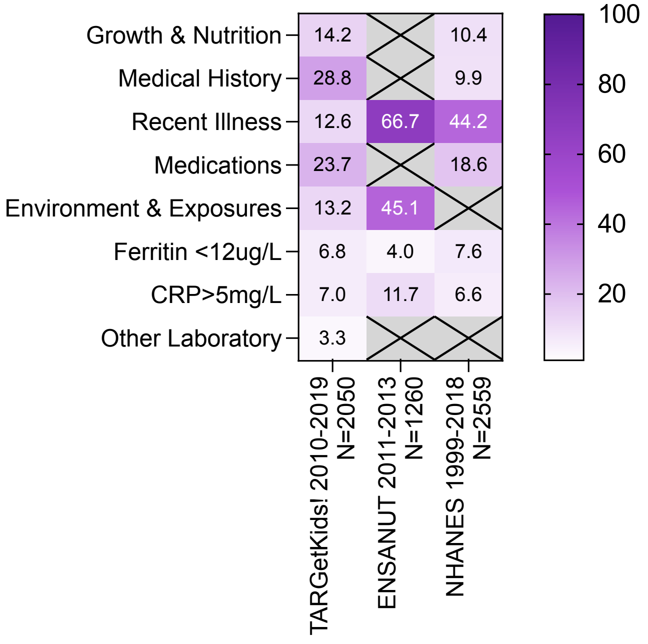

This represents the percentage of the total data set in each survey excluded by individual or grouped criteria. Data sources consisted of TARGet Kids!, ENSANUT, and NHANES. CRP=C-Reactive Protein.

Table 4.3.1 CAN TARGet Kids: Participant characteristics of violations (24-59 months).

	**Overall sample Male and Female N=2,050**
Reference sample[1]	
No	1,293/2,050 (63·1%)
Yes	757/2,050 (36·9%)
VIOLATION: Low birth weight (<2.5 kg) if age ≤24 months	
No	1,861/2,050 (90·8%)
Yes	43/2,050 (2·1%)
Missing	146/2,050 (7·1%)
VIOLATION: Weight z-score <-2 or >+2	
No	1,903/2,050 (92·8%)
Yes	101/2,050 (4·9%)
Missing	46/2,050 (2·2%)
VIOLATION: Length/height z-score <-2 or >+2	
No	1,880/2,050 (91·7%)
Yes	120/2,050 (5·9%)
Missing	50/2,050 (2·4%)
VIOLATION: Weight for length z-score <-2 or >+2	
No	1,872/2,050 (91·3%)
Yes	125/2,050 (6·1%)
Missing	53/2,050 (2·6%)
VIOLATION: BMI z-score <-2 or >+2	
No	1,863/2,050 (90·9%)
Yes	137/2,050 (6·7%)
Missing	50/2,050 (2·4%)
VIOLATION: Anaemia (mother)	
No	1,757/2,050 (85·7%)
Yes	120/2,050 (5·9%)
Missing	173/2,050 (8·4%)
VIOLATION: Birth (<37 weeks or > 42 weeks) if age ≤24 months	
No	1,721/2,050 (84·0%)
Yes	37/2,050 (1·8%)
Missing	292/2,050 (14·2%)
VIOLATION: Allergies	
No	1,006/2,050 (49·1%)
Yes	81/2,050 (4·0%)
Missing	963/2,050 (47·0%)
VIOLATION: Asthma	
No	1,650/2,050 (80·5%)
Yes	103/2,050 (5·0%)
Missing	297/2,050 (14·5%)
VIOLATION: Austism/PDD	
No	1,737/2,050 (84·7%)
Yes	12/2,050 (0·6%)
Missing	301/2,050 (14·7%)
VIOLATION: Development delay	
No	1,046/2,050 (51·0%)
Yes	37/2,050 (1·8%)
Missing	967/2,050 (47·2%)
VIOLATION: Diabetes	
No	1,745/2,050 (85·1%)
Yes	3/2,050 (0·1%)
Missing	302/2,050 (14·7%)
VIOLATION: At least one diagnosis	
No	1,323/2,050 (64·5%)
Yes	372/2,050 (18·1%)
Missing	355/2,050 (17·3%)
VIOLATION: Overweight	
No	1,072/2,050 (52·3%)
Yes	3/2,050 (0·1%)
Missing	975/2,050 (47·6%)
VIOLATION: IBD	
No	469/2,050 (22·9%)
Missing	1,581/2,050 (77·1%)
VIOLATION: Cancer	
No	598/2,050 (29·2%)
Missing	1,452/2,050 (70·8%)

	Overall sample<br>Male and Female<br>N=2,050
VIOLATION: Other diagnosis	
No	1,052/2,050 (51·3%)
Yes	124/2,050 (6·0%)
Missing	874/2,050 (42·6%)
VIOLATION: Ill past month	
No	354/2,050 (17·3%)
Yes	259/2,050 (12·6%)
Missing	1,437/2,050 (70·1%)
VIOLATION: Regular prescribed medicine	
No	1,741/2,050 (84·9%)
Yes	95/2,050 (4·6%)
Missing	214/2,050 (10·4%)
VIOLATION: Prescribed medicine past 2 weeks	
No	621/2,050 (30·3%)
Yes	64/2,050 (3·1%)
Missing	1,365/2,050 (66·6%)
VIOLATION: Cold or flu medicine past month	
No	294/2,050 (14·3%)
Yes	139/2,050 (6·8%)
Missing	1,617/2,050 (78·9%)
VIOLATION: Other medicine past month	
No	282/2,050 (13·8%)
Yes	167/2,050 (8·1%)
Missing	1,601/2,050 (78·1%)
VIOLATION: Cold or flu medicine past 2 weeks	
No	622/2,050 (30·3%)
Yes	55/2,050 (2·7%)
Missing	1,373/2,050 (67·0%)
VIOLATION: Other medicine past 2 weeks	
No	623/2,050 (30·4%)
Yes	54/2,050 (2·6%)
Missing	1,373/2,050 (67·0%)
VIOLATION: Iron (e.g., Ferinsol, Palafer) intake	
No	1,347/2,050 (65·7%)
Yes	21/2,050 (1·0%)
Missing	682/2,050 (33·3%)
VIOLATION: Other iron intake	
No	680/2,050 (33·2%)
Yes	6/2,050 (0·3%)
Missing	1,364/2,050 (66·5%)
VIOLATION: Traditional remedies or cosmetics last 3 months	
No	571/2,050 (27·9%)
Yes	35/2,050 (1·7%)
Missing	1,444/2,050 (70·4%)
VIOLATION: Lead (household)	
No	546/2,050 (26·6%)
Yes	59/2,050 (2·9%)
Missing	1,445/2,050 (70·5%)
VIOLATION: Smoking (household)	
No	1,626/2,050 (79·3%)
Yes	218/2,050 (10·6%)
Missing	206/2,050 (10·0%)
VIOLATION: Ferritin<12ug/L	
No	1,911/2,050 (93·2%)
Yes	139/2,050 (6·8%)
VIOLATION: C-Reactive Protein>5mg/L	
No	1,906/2,050 (93·0%)
Yes	144/2,050 (7·0%)
VIOLATION: Albumin<32g/L	
No	1,818/2,050 (88·7%)
Yes	1/2,050 (0·0%)
Missing	231/2,050 (11·3%)
VIOLATION: Alkaline Phosphatase (age and sex dependent)	
No	1,777/2,050 (86·7%)
Yes	41/2,050 (2·0%)
Missing	232/2,050 (11·3%)
VIOLATION: ALT serum≥35U/L	

	Overall sample<br>Male and Female<br>N=2,050
No	2,023/2,050 (98·7%)
Yes	27/2,050 (1·3%)

CAN = Canada, TARGet Kids! = The Applied Research Group for Kids, BMI = Body Mass Index.
Data are presented as n/total (%) based on participants with non-missing haemoglobin, ferritin, and C-reactive protein.
Missing: No answer. If a measurement of one or more of the healthy criteria is missing, then the participant is included in the reference sample.
[1] Ferritin≥12ug/L and C-reactive protein≤5mg/L.

Table 4.3.2 ECU ENSANUT 2011-2013: Participant characteristics of violations (24-59 months).

	**Overall sample Male and Female N=1,260**
Reference sample[1]	180/1,260 (14·3%)
VIOLATION: Altitude > 750 meters	
No	692/1,260 (54·9%)
Yes	568/1,260 (45·1%)
VIOLATION: Health problems in the last 30 days	
No	474/1,260 (37·6%)
Yes	786/1,260 (62·4%)
VIOLATION: Hospitalisation in the last 30 days	
No	1,201/1,260 (95·3%)
Yes	59/1,260 (4·7%)
VIOLATION: Diarrhea in the last 2 weeks	
No	1,112/1,260 (88·3%)
Yes	134/1,260 (10·6%)
No data	1/1,260 (0·1%)
Missing	13/1,260 (1·0%)
VIOLATION: Cough, runny nose in the last 2 weeks	
No	676/1,260 (53·7%)
Yes	571/1,260 (45·3%)
Missing	13/1,260 (1·0%)
VIOLATION: Ferritin<12ug/L	
No	1,210/1,260 (96·0%)
Yes	50/1,260 (4·0%)
VIOLATION: C-Reactive Protein>5mg/L	
No	1,112/1,260 (88·3%)
Yes	148/1,260 (11·7%)

ECU = Ecuador, ENSANUT = Encuesta National de Salud y Nutricion.
Data are presented as unweighted n/total (%) based on participants with non-missing haemoglobin, ferritin, and C-reactive protein.
No data: No answer/refused, Don't know; Missing: Not applicable, Not asked. If a measurement of one or more of the healthy criteria is missing, then the participant is included in the reference sample.
[1] Ferritin≥12ug/L and C-reactive protein≤5mg/L.

Table 4.3.3 US NHANES 1999-2018: Participant characteristics of violations (24-59 months).

	**Overall sample Male and Female N=2,559**
Reference sample[1]	937/2,559 (36·6%)
VIOLATION: BMI age and sex dependent	
No	2,179/2,559 (85·2%)
Yes	266/2,559 (10·4%)
No data	81/2,559 (3·2%)
Missing	33/2,559 (1·3%)
VIOLATION: Doctor told you have diabetes	
No	2,555/2,559 (99·8%)
Yes	2/2,559 (0·1%)
No data	2/2,559 (0·1%)
VIOLATION: Still have asthma	
No	88/2,559 (3·4%)
Yes	209/2,559 (8·2%)
No data	6/2,559 (0·2%)
Missing	2,256/2,559 (88·2%)
VIOLATION: Taking treatment for anaemia/past 3 months	
No	2,504/2,559 (97·9%)
Yes	53/2,559 (2·1%)
No data	2/2,559 (0·1%)
VIOLATION: Overnight hospital patient in last year	
No	2,430/2,559 (95·0%)
Yes	127/2,559 (5·0%)
No data	2/2,559 (0·1%)
VIOLATION: SP have head cold or chest cold?	
No	1,518/2,559 (59·3%)
Yes	879/2,559 (34·3%)
No data	162/2,559 (6·3%)
VIOLATION: SP have stomach or intestinal illness?	
No	2,124/2,559 (83·0%)
Yes	279/2,559 (10·9%)
No data	156/2,559 (6·1%)
VIOLATION: SP have flu, pneumonia, ear infection?	
No	2,176/2,559 (85·0%)
Yes	228/2,559 (8·9%)
No data	155/2,559 (6·1%)
VIOLATION: Taken prescription medicine, past month	
No	2,102/2,559 (82·1%)
Yes	455/2,559 (17·8%)
No data	2/2,559 (0·1%)
VIOLATION: For anaemia, such as low iron	
No	319/2,559 (12·5%)
Yes	22/2,559 (0·9%)
No data	882/2,559 (34·5%)
Missing	1,336/2,559 (52·2%)
VIOLATION: Ferritin<12ug/L	
No	2,365/2,559 (92·4%)
Yes	194/2,559 (7·6%)
VIOLATION: C-Reactive Protein>5mg/L	
No	2,390/2,559 (93·4%)
Yes	169/2,559 (6·6%)

US = United States, NHANES = National Health and Nutrition Examination Survey, BMI = Body Mass Index, SP = Survey Participant.
Data are presented as unweighted n/total (%) based on participants with non-missing haemoglobin, ferritin, and C-reactive protein. Data was combined from 1999-2018. No ferritin was measured in participants less than 3 years in survey years 1999-2000, 2001-2002, 2007-2008 and 2009-2010. No ferritin was measured in participants in survey years 2011-2012 and 2013-2014. VIOLATION: For anaemia, such as low iron - only asked in survey years 2007-2008, 2009-2010, 2015-2016, and 2017-2018.
No data: No answer/refused, Don't know; Missing: Not applicable, Not asked. If a measurement of one or more of the healthy criteria is missing, then the participant is included in the reference sample.
[1] Ferritin≥12ug/L and C-reactive protein≤5mg/L.

4.4 Detailed Individual Exclusions Children (5-11 years)

Figure 4.4 Summary of exclusions of special interest from overall sample in children (5-11 years).

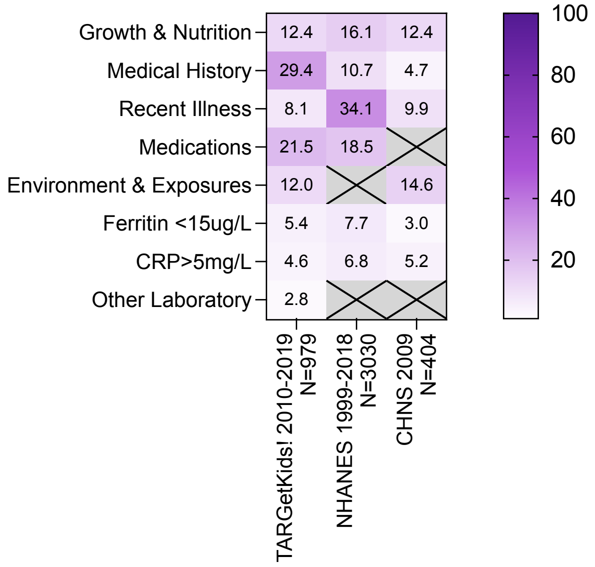

This represents the percentage of the total data set in each survey excluded by individual or grouped criteria. Data sources consisted of TARGet Kids!, NHANES, and CHNS. CRP=C-Reactive Protein.

Table 4.4.1 CAN TARGet Kids: Participant characteristics of violations (5-11 years).

	**Overall sample Male and Female N=979**
Reference sample[1]	
No	587/979 (60·0%)
Yes	392/979 (40·0%)
VIOLATION: Weight z-score <-2 or >+2	
No	866/979 (88·5%)
Yes	71/979 (7·3%)
Missing	42/979 (4·3%)
VIOLATION: Length/height z-score <-2 or >+2	
No	894/979 (91·3%)
Yes	65/979 (6·6%)
Missing	20/979 (2·0%)
VIOLATION: Weight for length z-score <-2 or >+2	
No	133/979 (13·6%)
Yes	7/979 (0·7%)
Missing	839/979 (85·7%)
VIOLATION: BMI z-score <-2 or >+2	
No	891/979 (91·0%)
Yes	66/979 (6·7%)
Missing	22/979 (2·2%)
VIOLATION: Anaemia (mother)	
No	873/979 (89·2%)
Yes	49/979 (5·0%)
Missing	57/979 (5·8%)
VIOLATION: Allergies	
No	495/979 (50·6%)
Yes	63/979 (6·4%)
Missing	421/979 (43·0%)
VIOLATION: Asthma	
No	777/979 (79·4%)
Yes	86/979 (8·8%)
Missing	116/979 (11·8%)
VIOLATION: Austism/PDD	
No	837/979 (85·5%)
Yes	15/979 (1·5%)
Missing	127/979 (13·0%)
VIOLATION: Development delay	
No	528/979 (53·9%)
Yes	14/979 (1·4%)
Missing	437/979 (44·6%)
VIOLATION: Diabetes	
No	845/979 (86·3%)
Yes	3/979 (0·3%)
Missing	131/979 (13·4%)
VIOLATION: At least one diagnosis	
No	631/979 (64·5%)
Yes	186/979 (19·0%)
Missing	162/979 (16·5%)
VIOLATION: Overweight	
No	537/979 (54·9%)
Yes	5/979 (0·5%)
Missing	437/979 (44·6%)
VIOLATION: IBD	
No	186/979 (19·0%)
Yes	2/979 (0·2%)
Missing	791/979 (80·8%)
VIOLATION: Cancer	
No	277/979 (28·3%)
Yes	2/979 (0·2%)
Missing	700/979 (71·5%)
VIOLATION: Other diagnosis	
No	558/979 (57·0%)
Yes	62/979 (6·3%)
Missing	359/979 (36·7%)
VIOLATION: Ill past month	
No	194/979 (19·8%)

	Overall sample<br>Male and Female<br>N=979
Yes	79/979 (8·1%)
Missing	706/979 (72·1%)
VIOLATION: Regular prescribed medicine	
No	877/979 (89·6%)
Yes	54/979 (5·5%)
Missing	48/979 (4·9%)
VIOLATION: Prescribed medicine past 2 weeks	
No	292/979 (29·8%)
Yes	19/979 (1·9%)
Missing	668/979 (68·2%)
VIOLATION: Cold or flu medicine past month	
No	95/979 (9·7%)
Yes	51/979 (5·2%)
Missing	833/979 (85·1%)
VIOLATION: Other medicine past month	
No	81/979 (8·3%)
Yes	67/979 (6·8%)
Missing	831/979 (84·9%)
VIOLATION: Cold or flu medicine past 2 weeks	
No	279/979 (28·5%)
Yes	24/979 (2·5%)
Missing	676/979 (69·1%)
VIOLATION: Other medicine past 2 weeks	
No	283/979 (28·9%)
Yes	20/979 (2·0%)
Missing	676/979 (69·1%)
VIOLATION: Iron (e.g. Ferinsol, Palafer) intake	
No	602/979 (61·5%)
Yes	7/979 (0·7%)
Missing	370/979 (37·8%)
VIOLATION: Other iron intake	
No	306/979 (31·3%)
Yes	1/979 (0·1%)
Missing	672/979 (68·6%)
VIOLATION: Traditional remedies or cosmetics last 3 months	
No	259/979 (26·5%)
Yes	12/979 (1·2%)
Missing	708/979 (72·3%)
VIOLATION: Lead (household)	
No	247/979 (25·2%)
Yes	23/979 (2·3%)
Missing	709/979 (72·4%)
VIOLATION: Smoking (household)	
No	822/979 (84·0%)
Yes	99/979 (10·1%)
Missing	58/979 (5·9%)
VIOLATION: Ferritin<15ug/L	
No	926/979 (94·6%)
Yes	53/979 (5·4%)
VIOLATION: C-Reactive Protein>5mg/L	
No	934/979 (95·4%)
Yes	45/979 (4·6%)
VIOLATION: Albumin<32g/L	
No	955/979 (97·5%)
Missing	24/979 (2·5%)
VIOLATION: Alkaline Phosphatase (age and sex dependent)	
No	942/979 (96·2%)
Yes	13/979 (1·3%)
Missing	24/979 (2·5%)
VIOLATION: ALT serum ≥35U/L	
No	962/979 (98·3%)
Yes	16/979 (1·6%)
Missing	1/979 (0·1%)

CAN = Canada, TARGet Kids! = The Applied Research Group for Kids, BMI = Body Mass Index.

Data are presented as n/total (%) based on participants with non-missing haemoglobin, ferritin, and C-reactive protein.
Missing: No answer. If a measurement of one or more of the healthy criteria is missing, then the participant is included in the reference sample.
[1] Ferritin≥15ug/L and C-reactive protein≤5mg/L.

Table 4.4.2 US NHANES 1999-2018: Participant characteristics of violations (5-11 years).

	**Overall sample Male and Female N=3,030**
Reference sample[1]	1,201/3,030 (39·6%)
VIOLATION: BMI age and sex dependent	
No	2,514/3,030 (83·0%)
Yes	487/3,030 (16·1%)
No data	29/3,030 (1·0%)
VIOLATION: Doctor told you have diabetes	
No	3,023/3,030 (99·8%)
Yes	4/3,030 (0·1%)
No data	3/3,030 (0·1%)
VIOLATION: Still have asthma	
No	146/3,030 (4·8%)
Yes	292/3,030 (9·6%)
No data	12/3,030 (0·4%)
Missing	2,580/3,030 (85·1%)
VIOLATION: Taking treatment for anaemia/past 3 months	
No	2,992/3,030 (98·7%)
Yes	36/3,030 (1·2%)
No data	2/3,030 (0·1%)
VIOLATION: Overnight hospital patient in last year	
No	2,926/3,030 (96·6%)
Yes	100/3,030 (3·3%)
No data	4/3,030 (0·1%)
VIOLATION: SP have head cold or chest cold?	
No	2,121/3,030 (70·0%)
Yes	742/3,030 (24·5%)
No data	167/3,030 (5·5%)
VIOLATION: SP have stomach or intestinal illness?	
No	2,568/3,030 (84·8%)
Yes	300/3,030 (9·9%)
No data	162/3,030 (5·3%)
VIOLATION: SP have flu, pneumonia, ear infection?	
No	2,677/3,030 (88·3%)
Yes	188/3,030 (6·2%)
No data	165/3,030 (5·4%)
VIOLATION: Pregnancy status at exam	
No	137/3,030 (4·5%)
No data	189/3,030 (6·2%)
Missing	2,704/3,030 (89·2%)
VIOLATION: Urine Pregnancy Result	
No	34/3,030 (1·1%)
No data	292/3,030 (9·6%)
Missing	2,704/3,030 (89·2%)
VIOLATION: Taken prescription medicine, past month	
No	2,471/3,030 (81·6%)
Yes	558/3,030 (18·4%)
No data	1/3,030 (0·0%)
VIOLATION: For anaemia, such as low iron	
No	120/3,030 (4·0%)
Yes	7/3,030 (0·2%)
No data	426/3,030 (14·1%)
Missing	2,477/3,030 (81·7%)
VIOLATION: Ferritin<15ug/L	
No	2,798/3,030 (92·3%)
Yes	232/3,030 (7·7%)
VIOLATION: C-Reactive Protein>5mg/L	
No	2,824/3,030 (93·2%)
Yes	206/3,030 (6·8%)

US = United States, NHANES = National Health and Nutrition Examination Survey, BMI = Body Mass Index, SP = Survey Participant.

Data are presented as unweighted n/total (%) based on participants with non-missing haemoglobin, ferritin, and C-reactive protein. Data was combined from 1999-2018. No ferritin was measured in participants 6 to 11 years in survey years 2003-2004, 2005-2006, 2007-2008, 2009-2010, 2015-2016, 2017-2018. No ferritin was measured in participants in survey years 2011-2012 and 2013-2014. VIOLATION: For anaemia, such as low iron – only asked in

NHANES 2007-2008, 2009-2010, 2015-2016, 2017-2018.
No data: No answer/refused, Don't know; Missing: Not applicable, Not asked. If a measurement of one or more of the healthy criteria is missing, then the participant is included in the reference sample.
[1] Ferritin≥15ug/L and C-reactive protein≤5mg/L.

Table 4.4.3 CHN CHNS 2009: Participant characteristics of violations (5-11 years).

	**Overall sample Male and Female N=404**
Reference sample[1]	249/404 (61·6%)
VIOLATION: BMI age and sex dependent	
No	340/404 (84·2%)
Yes	50/404 (12·4%)
Missing	14/404 (3·5%)
VIOLATION: Illness/Injury – Infectious/parasitic disease	
No	31/404 (7·7%)
Missing	373/404 (92·3%)
VIOLATION: Illness/Injury – Heart disease	
No	31/404 (7·7%)
Missing	373/404 (92·3%)
VIOLATION: Illness/Injury – Tumor	
No	31/404 (7·7%)
Missing	373/404 (92·3%)
VIOLATION: Illness/Injury – Respiratory disease	
No	15/404 (3·7%)
Yes	16/404 (4·0%)
Missing	373/404 (92·3%)
VIOLATION: Illness/Injury – Injury	
No	30/404 (7·4%)
Yes	1/404 (0·2%)
Missing	373/404 (92·3%)
VIOLATION: Illness/Injury – Alcohol poisoning	
No	31/404 (7·7%)
Missing	373/404 (92·3%)
VIOLATION: Illness/Injury – Endocrine disorder	
No	31/404 (7·7%)
Missing	373/404 (92·3%)
VIOLATION: Illness/Injury – Hematological disease	
No	31/404 (7·7%)
Missing	373/404 (92·3%)
VIOLATION: Illness/Injury – Mental/psychiatric disorder	
No	31/404 (7·7%)
Missing	373/404 (92·3%)
VIOLATION: Illness/Injury – Neurological disorder	
No	31/404 (7·7%)
Missing	373/404 (92·3%)
VIOLATION: Illness/Injury – Eye/ear/nose/throat/teeth disease	
No	31/404 (7·7%)
Missing	373/404 (92·3%)
VIOLATION: Illness/Injury – Digestive disease	
No	29/404 (7·2%)
Yes	2/404 (0·5%)
Missing	373/404 (92·3%)
VIOLATION: Illness/Injury – Urinary disease	
No	31/404 (7·7%)
Missing	373/404 (92·3%)
VIOLATION: Illness/Injury – Sexual dysfunction	
No	31/404 (7·7%)
Missing	373/404 (92·3%)
VIOLATION: Illness/Injury – Obstetrical/gynecological disease	
No	31/404 (7·7%)
Missing	373/404 (92·3%)
VIOLATION: Illness/Injury – Dermatological disease	
No	31/404 (7·7%)
Missing	373/404 (92·3%)
VIOLATION: Illness/Injury – Muscular/rheumatological disease	
No	31/404 (7·7%)
Missing	373/404 (92·3%)
VIOLATION: Diagnosed with high blood pressure	
No	5/404 (1·2%)
Missing	399/404 (98·8%)
VIOLATION: Diagnosed with diabetes	
No	5/404 (1·2%)
Missing	399/404 (98·8%)

	Overall sample<br>Male and Female<br>N=404
VIOLATION: Diagnosed with myocardial infarction	
Missing	404/404 (100·0%)
VIOLATION: Diagnosed with apoplexy	
Missing	404/404 (100·0%)
VIOLATION: Dr. told you that you suffer from asthma	
Missing	404/404 (100·0%)
VIOLATION: Past 12 months – had whistling/wheezing in chest	
Missing	404/404 (100·0%)
VIOLATION: Last 4 weeks – been sick or injured	
No	367/404 (90·8%)
Yes	28/404 (6·9%)
Missing	9/404 (2·2%)
VIOLATION: Last 4 weeks – fever, sore throat, cough	
No	370/404 (91·6%)
Yes	32/404 (7·9%)
Missing	2/404 (0·5%)
VIOLATION: Last 4 weeks – diarrhea, stomachache	
No	400/404 (99·0%)
Yes	1/404 (0·2%)
Missing	3/404 (0·7%)
VIOLATION: Last 4 weeks – stomachache	
No	401/404 (99·3%)
Yes	1/404 (0·2%)
Missing	2/404 (0·5%)
VIOLATION: Last 4 weeks – asthma	
No	402/404 (99·5%)
Missing	2/404 (0·5%)
VIOLATION: Last 4 weeks – headache, dizziness	
No	400/404 (99·0%)
Yes	2/404 (0·5%)
Missing	2/404 (0·5%)
VIOLATION: Last 4 weeks – joint, muscle pain	
No	402/404 (99·5%)
Missing	2/404 (0·5%)
VIOLATION: Last 4 weeks – rash, dermatitis	
No	401/404 (99·3%)
Yes	1/404 (0·2%)
Missing	2/404 (0·5%)
VIOLATION: Last 4 weeks – eye/ear disease	
No	402/404 (99·5%)
Missing	2/404 (0·5%)
VIOLATION: Last 4 weeks – heart disease/chest pain	
No	402/404 (99·5%)
Missing	2/404 (0·5%)
VIOLATION: Last 4 weeks – other infectious disease	
No	399/404 (98·8%)
Yes	2/404 (0·5%)
Missing	3/404 (0·7%)
VIOLATION: Last 4 weeks – noncommunicable disease	
No	399/404 (98·8%)
Yes	1/404 (0·2%)
No data	1/404 (0·2%)
Missing	3/404 (0·7%)
VIOLATION: Last 4 weeks – days hospitalised	
Yes	1/404 (0·2%)
Missing	403/404 (99·8%)
VIOLATION: Taking anti-hypertension drugs	
Missing	404/404 (100·0%)
VIOLATION: Province – Guizhou – Altitude	
No	345/404 (85·4%)
Yes	59/404 (14·6%)
VIOLATION: Ferritin<15ug/L	
No	392/404 (97·0%)
Yes	12/404 (3·0%)
VIOLATION: C-Reactive Protein>5mg/L	
No	383/404 (94·8%)
Yes	21/404 (5·2%)

CHN = China, CHNS = China Health and Nutrition Survey, BMI = Body Mass Index.
Data are presented as unweighted n/total (%) based on participants with non-missing haemoglobin, ferritin, and C-reactive protein.
No data: No answer/refused, Don't know; Missing: Not applicable, Not asked. If a measurement of one or more of the healthy criteria is missing, then the participant is included in the reference sample.
[1] Ferritin≥15ug/L and C-reactive protein≤5mg/L.

4.5 Detailed Individual Exclusions Adolescents (12-17 years)

Figure 4.5.1 Summary of exclusions of special interest from overall sample in males (12-17 years).

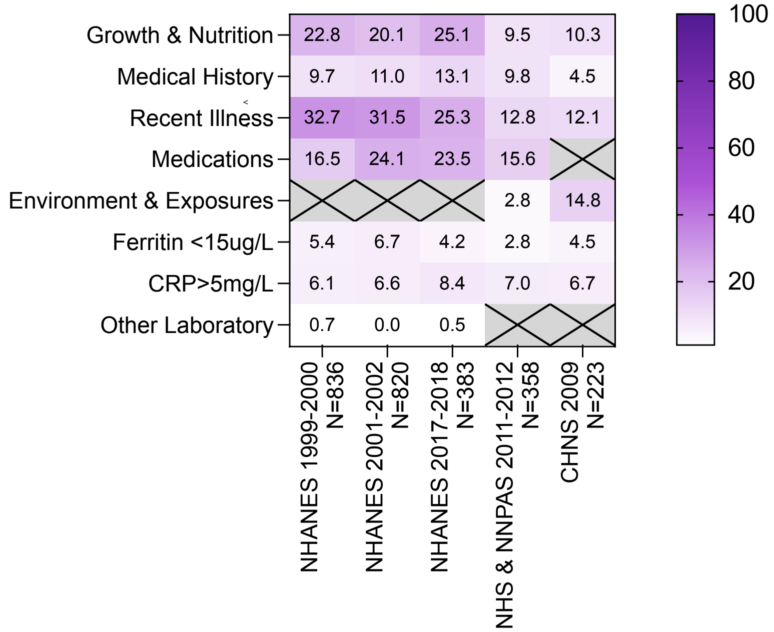

This represents the percentage of the total data set in each survey excluded by individual or grouped criteria. Data sources consisted of NHANES, AHS, and CHNS.CRP=C-Reactive Protein.

Figure 4.5.2 Summary of exclusions of special interest from overall sample in non-pregnant females (12-17 years).

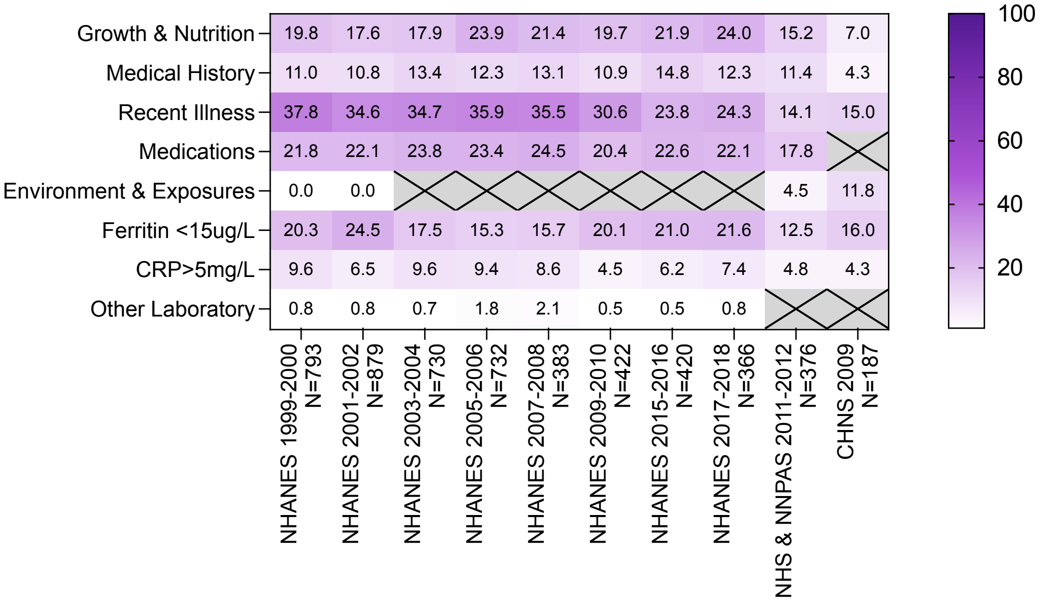

This represents the percentage of the total data set in each survey excluded by individual or grouped criteria. Data sources consisted of NHANES, AHS, and CHNS.CRP=C-Reactive Protein.

Table 4.5.1 US NHANES 1999-2000: Participant characteristics of violations (12-17 years).

	Overall sample	
	**Male N=836**	**Female N=793**
Reference sample[1]	334/836 (40·0%)	233/793 (29·4%)
VIOLATION: BMI age and sex dependent		
No	641/836 (76·7%)	631/793 (79·6%)
Yes	191/836 (22·8%)	157/793 (19·8%)
No data	4/836 (0·5%)	5/793 (0·6%)
VIOLATION: Doctor told you have diabetes		
No	835/836 (99·9%)	791/793 (99·7%)
Yes	1/836 (0·1%)	2/793 (0·3%)
VIOLATION: Still have asthma		
No	48/836 (5·7%)	39/793 (4·9%)
Yes	79/836 (9·4%)	74/793 (9·3%)
No data	4/836 (0·5%)	1/793 (0·1%)
Missing	705/836 (84·3%)	679/793 (85·6%)
VIOLATION: Taking treatment for anaemia/past 3 months		
No	832/836 (99·5%)	780/793 (98·4%)
Yes	4/836 (0·5%)	12/793 (1·5%)
No data	0/836 (0·0%)	1/793 (0·1%)
VIOLATION: Overnight hospital patient in last year		
No	815/836 (97·5%)	759/793 (95·7%)
Yes	20/836 (2·4%)	34/793 (4·3%)
No data	1/836 (0·1%)	0/793 (0·0%)
VIOLATION: SP donated blood in past 12 months		
No	253/836 (30·3%)	225/793 (28·4%)
Yes	14/836 (1·7%)	14/793 (1·8%)
No data	15/836 (1·8%)	9/793 (1·1%)
Missing	554/836 (66·3%)	545/793 (68·7%)
VIOLATION: SP have head cold or chest cold?		
No	628/836 (75·1%)	587/793 (74·0%)
Yes	172/836 (20·6%)	184/793 (23·2%)
No data	36/836 (4·3%)	22/793 (2·8%)
VIOLATION: SP have stomach or intestinal illness?		
No	732/836 (87·6%)	693/793 (87·4%)
Yes	70/836 (8·4%)	79/793 (10·0%)
No data	34/836 (4·1%)	21/793 (2·6%)
VIOLATION: SP have flu, pneumonia, ear infection?		
No	742/836 (88·8%)	706/793 (89·0%)
Yes	57/836 (6·8%)	66/793 (8·3%)
No data	37/836 (4·4%)	21/793 (2·6%)
VIOLATION: Are you pregnant now?		
Yes	0/836 (0·0%)	21/793 (2·6%)
No data	0/836 (0·0%)	343/793 (43·3%)
Missing	836/836 (100·0%)	429/793 (54·1%)
VIOLATION: Taken prescription medicine, past month		
No	698/836 (83·5%)	620/793 (78·2%)
Yes	138/836 (16·5%)	173/793 (21·8%)
VIOLATION: Ferritin<15ug/L		
No	791/836 (94·6%)	632/793 (79·7%)
Yes	45/836 (5·4%)	161/793 (20·3%)
VIOLATION: C-Reactive Protein>5mg/L		
No	785/836 (93·9%)	717/793 (90·4%)
Yes	51/836 (6·1%)	76/793 (9·6%)
VIOLATION: eGFR<90 ml/min/1.73m^2		
No	816/836 (97·6%)	775/793 (97·7%)
Yes	6/836 (0·7%)	6/793 (0·8%)
No data	5/836 (0·6%)	5/793 (0·6%)
Missing	9/836 (1·1%)	7/793 (0·9%)

US = United States, NHANES = National Health and Nutrition Examination Survey, BMI = Body Mass Index, SP = Survey Participant.
Data are presented as unweighted n/total (%) based on participants with non-missing haemoglobin, ferritin, and C-reactive protein.
No data: No answer/refused, Don't know; Missing: Not applicable, Not asked. If a measurement of one or more of the healthy criteria is missing, then the participant is included in the reference sample.

[1] Ferritin≥15ug/L and C-reactive protein≤5mg/L.

Table 4.5.2 US NHANES 2001-2002: Participant characteristics of violations (12-17 years).

	Overall sample	
	**Male N=820**	**Female N=879**
Reference sample[1]	291/820 (35·5%)	245/879 (27·9%)
VIOLATION: BMI age and sex dependent		
No	648/820 (79·0%)	711/879 (80·9%)
Yes	165/820 (20·1%)	155/879 (17·6%)
No data	7/820 (0·9%)	13/879 (1·5%)
VIOLATION: Doctor told you have diabetes		
No	814/820 (99·3%)	876/879 (99·7%)
Yes	6/820 (0·7%)	3/879 (0·3%)
VIOLATION: Still have asthma		
No	70/820 (8·5%)	51/879 (5·8%)
Yes	85/820 (10·4%)	82/879 (9·3%)
No data	1/820 (0·1%)	4/879 (0·5%)
Missing	664/820 (81·0%)	742/879 (84·4%)
VIOLATION: Taking treatment for anaemia/past 3 months		
No	818/820 (99·8%)	868/879 (98·7%)
Yes	2/820 (0·2%)	11/879 (1·3%)
VIOLATION: Overnight hospital patient in last year		
No	796/820 (97·1%)	834/879 (94·9%)
Yes	24/820 (2·9%)	45/879 (5·1%)
VIOLATION: SP donated blood in past 12 months		
No	273/820 (33·3%)	248/879 (28·2%)
Yes	13/820 (1·6%)	11/879 (1·3%)
No data	14/820 (1·7%)	18/879 (2·0%)
Missing	520/820 (63·4%)	602/879 (68·5%)
VIOLATION: SP have head cold or chest cold?		
No	616/820 (75·1%)	637/879 (72·5%)
Yes	167/820 (20·4%)	192/879 (21·8%)
No data	37/820 (4·5%)	50/879 (5·7%)
VIOLATION: SP have stomach or intestinal illness?		
No	727/820 (88·7%)	753/879 (85·7%)
Yes	57/820 (7·0%)	76/879 (8·6%)
No data	36/820 (4·4%)	50/879 (5·7%)
VIOLATION: SP have flu, pneumonia, ear infection?		
No	735/820 (89·6%)	772/879 (87·8%)
Yes	49/820 (6·0%)	57/879 (6·5%)
No data	36/820 (4·4%)	50/879 (5·7%)
VIOLATION: Pregnancy status at exam		
No	0/820 (0·0%)	832/879 (94·7%)
Yes	0/820 (0·0%)	17/879 (1·9%)
No data	0/820 (0·0%)	30/879 (3·4%)
Missing	820/820 (100·0%)	0/879 (0·0%)
VIOLATION: Urine Pregnancy Result		
No	0/820 (0·0%)	849/879 (96·6%)
Yes	0/820 (0·0%)	17/879 (1·9%)
No data	0/820 (0·0%)	13/879 (1·5%)
Missing	820/820 (100·0%)	0/879 (0·0%)
VIOLATION: Are you pregnant now?		
No	0/820 (0·0%)	17/879 (1·9%)
Yes	0/820 (0·0%)	15/879 (1·7%)
No data	0/820 (0·0%)	842/879 (95·8%)
Missing	820/820 (100·0%)	5/879 (0·6%)
VIOLATION: Taken prescription medicine, past month		
No	622/820 (75·9%)	685/879 (77·9%)
Yes	198/820 (24·1%)	194/879 (22·1%)
VIOLATION: Ferritin<15ug/L		
No	765/820 (93·3%)	664/879 (75·5%)
Yes	55/820 (6·7%)	215/879 (24·5%)
VIOLATION: C-Reactive Protein>5mg/L		
No	766/820 (93·4%)	822/879 (93·5%)
Yes	54/820 (6·6%)	57/879 (6·5%)
VIOLATION: eGFR<90 ml/min/1.73m^2		
No	807/820 (98·4%)	862/879 (98·1%)
Yes	0/820 (0·0%)	7/879 (0·8%)
No data	7/820 (0·9%)	5/879 (0·6%)
Missing	6/820 (0·7%)	5/879 (0·6%)

US = United States, NHANES = National Health and Nutrition Examination Survey, BMI = Body Mass Index, SP = Survey Participant.
Data are presented as unweighted n/total (%) based on participants with non-missing haemoglobin, ferritin, and C-reactive protein.
No data: No answer/refused, Don't know; Missing: Not applicable, Not asked. If a measurement of one or more of the healthy criteria is missing, then the participant is included in the reference sample.
[1] Ferritin≥15ug/L and C-reactive protein≤5mg/L.

Table 4.5.3 US NHANES 2003-2004: Participant characteristics of violations (Females) (12-17 years).

	Overall sample Female N=730
Reference sample[1]	243/730 (33·3%)
VIOLATION: BMI age and sex dependent	
No	590/730 (80·8%)
Yes	131/730 (17·9%)
No data	9/730 (1·2%)
VIOLATION: Doctor told you have diabetes	
No	724/730 (99·2%)
Yes	4/730 (0·5%)
No data	2/730 (0·3%)
VIOLATION: Still have asthma	
No	39/730 (5·3%)
Yes	80/730 (11·0%)
No data	3/730 (0·4%)
Missing	608/730 (83·3%)
VIOLATION: Taking treatment for anaemia/past 3 months	
No	714/730 (97·8%)
Yes	16/730 (2·2%)
VIOLATION: Overnight hospital patient in last year	
No	698/730 (95·6%)
Yes	32/730 (4·4%)
VIOLATION: SP donated blood in past 12 months	
No	221/730 (30·3%)
Yes	11/730 (1·5%)
No data	10/730 (1·4%)
Missing	488/730 (66·8%)
VIOLATION: SP have head cold or chest cold?	
No	535/730 (73·3%)
Yes	157/730 (21·5%)
No data	38/730 (5·2%)
VIOLATION: SP have stomach or intestinal illness?	
No	646/730 (88·5%)
Yes	47/730 (6·4%)
No data	37/730 (5·1%)
VIOLATION: SP have flu, pneumonia, ear infection?	
No	647/730 (88·6%)
Yes	46/730 (6·3%)
No data	37/730 (5·1%)
VIOLATION: Pregnancy status at exam	
No	697/730 (95·5%)
Yes	20/730 (2·7%)
No data	13/730 (1·8%)
VIOLATION: Urine Pregnancy Result	
No	705/730 (96·6%)
Yes	20/730 (2·7%)
No data	5/730 (0·7%)
VIOLATION: Are you pregnant now?	
No	24/730 (3·3%)
Yes	15/730 (2·1%)
No data	691/730 (94·7%)
VIOLATION: Taken prescription medicine, past month	
No	555/730 (76·0%)
Yes	174/730 (23·8%)
No data	1/730 (0·1%)
VIOLATION: Ferritin<15ug/L	
No	602/730 (82·5%)
Yes	128/730 (17·5%)
VIOLATION: C-Reactive Protein>5mg/L	
No	660/730 (90·4%)
Yes	70/730 (9·6%)
VIOLATION: eGFR<90 ml/min/1.73m^2	
No	715/730 (97·9%)
Yes	5/730 (0·7%)
No data	10/730 (1·4%)

US = United States, NHANES = National Health and Nutrition Examination Survey, BMI = Body Mass Index, SP = Survey Participant.
Data are presented as unweighted n/total (%) based on participants with non-missing haemoglobin, ferritin, and C-reactive protein. No ferritin was collected in males.
No data: No answer/refused, Don't know; Missing: Not applicable, Not asked. If a measurement of one or more of the healthy criteria is missing, then the participant is included in the reference sample.
[1] Ferritin≥15ug/L and C-reactive protein≤5mg/L.

Table 4.5.4 US NHANES 2005-2006: Participant characteristics of violations (Females) (12-17 years).

	**Overall sample Female N=732**
Reference sample[1]	220/732 (30·1%)
VIOLATION: BMI age and sex dependent	
No	552/732 (75·4%)
Yes	175/732 (23·9%)
No data	5/732 (0·7%)
VIOLATION: Doctor told you have diabetes	
No	728/732 (99·5%)
Yes	3/732 (0·4%)
No data	1/732 (0·1%)
VIOLATION: Still have asthma	
No	37/732 (5·1%)
Yes	80/732 (10·9%)
No data	2/732 (0·3%)
Missing	613/732 (83·7%)
VIOLATION: Taking treatment for anaemia/past 3 months	
No	725/732 (99·0%)
Yes	7/732 (1·0%)
VIOLATION: Overnight hospital patient in last year	
No	692/732 (94·5%)
Yes	40/732 (5·5%)
VIOLATION: SP donated blood in past 12 months	
No	223/732 (30·5%)
Yes	11/732 (1·5%)
No data	19/732 (2·6%)
Missing	479/732 (65·4%)
VIOLATION: SP have head cold or chest cold?	
No	529/732 (72·3%)
Yes	160/732 (21·9%)
No data	43/732 (5·9%)
VIOLATION: SP have stomach or intestinal illness?	
No	618/732 (84·4%)
Yes	71/732 (9·7%)
No data	43/732 (5·9%)
VIOLATION: SP have flu, pneumonia, ear infection?	
No	646/732 (88·3%)
Yes	43/732 (5·9%)
No data	43/732 (5·9%)
VIOLATION: Pregnancy status at exam	
No	697/732 (95·2%)
Yes	17/732 (2·3%)
No data	18/732 (2·5%)
VIOLATION: Urine Pregnancy Result	
No	704/732 (96·2%)
Yes	16/732 (2·2%)
No data	12/732 (1·6%)
VIOLATION: Are you pregnant now?	
No	28/732 (3·8%)
Yes	14/732 (1·9%)
No data	690/732 (94·3%)
VIOLATION: Taken prescription medicine, past month	
No	561/732 (76·6%)
Yes	171/732 (23·4%)
VIOLATION: Ferritin<15ug/L	
No	620/732 (84·7%)
Yes	112/732 (15·3%)
VIOLATION: C-Reactive Protein>5mg/L	
No	663/732 (90·6%)
Yes	69/732 (9·4%)
VIOLATION: eGFR<90 ml/min/1.73m^2	
No	715/732 (97·7%)
Yes	13/732 (1·8%)
No data	4/732 (0·5%)

US = United States, NHANES = National Health and Nutrition Examination Survey, BMI = Body Mass Index, SP = Survey Participant.

Data are presented as unweighted n/total (%) based on participants with non-missing haemoglobin, ferritin, and C-reactive protein. No ferritin was collected in males.
No data: No answer/refused, Don't know; Missing: Not applicable, Not asked. If a measurement of one or more of the healthy criteria is missing, then the participant is included in the reference sample.
[1] Ferritin≥15ug/L and C-reactive protein≤5mg/L.

Table 4.5.5 US NHANES 2007-2008: Participant characteristics of violations (Females) (12-17 years).

	Overall sample Female N=383
Reference sample[1]	119/383 (31·1%)
VIOLATION: BMI age and sex dependent	
No	293/383 (76·5%)
Yes	82/383 (21·4%)
No data	8/383 (2·1%)
VIOLATION: Doctor told you have diabetes	
No	380/383 (99·2%)
Yes	3/383 (0·8%)
VIOLATION: Still have asthma	
No	28/383 (7·3%)
Yes	46/383 (12·0%)
Missing	309/383 (80·7%)
VIOLATION: Taking treatment for anaemia/past 3 months	
No	381/383 (99·5%)
Yes	2/383 (0·5%)
VIOLATION: Overnight hospital patient in last year	
No	367/383 (95·8%)
Yes	16/383 (4·2%)
VIOLATION: SP donated blood in past 12 months	
No	111/383 (29·0%)
Yes	11/383 (2·9%)
No data	8/383 (2·1%)
Missing	253/383 (66·1%)
VIOLATION: SP have head cold or chest cold?	
No	269/383 (70·2%)
Yes	87/383 (22·7%)
No data	27/383 (7·0%)
VIOLATION: SP have stomach or intestinal illness?	
No	317/383 (82·8%)
Yes	39/383 (10·2%)
No data	27/383 (7·0%)
VIOLATION: SP have flu, pneumonia, ear infection?	
No	337/383 (88·0%)
Yes	19/383 (5·0%)
No data	27/383 (7·0%)
VIOLATION: Taken prescription medicine, past month	
No	288/383 (75·2%)
Yes	94/383 (24·5%)
No data	1/383 (0·3%)
VIOLATION: For anaemia, such as low iron	
Yes	1/383 (0·3%)
No data	382/383 (99·7%)
VIOLATION: Ferritin<15ug/L	
No	323/383 (84·3%)
Yes	60/383 (15·7%)
VIOLATION: C-Reactive Protein>5mg/L	
No	350/383 (91·4%)
Yes	33/383 (8·6%)
VIOLATION: eGFR<90 ml/min/1.73m^2	
No	371/383 (96·9%)
Yes	8/383 (2·1%)
No data	4/383 (1·0%)

US = United States, NHANES = National Health and Nutrition Examination Survey, BMI = Body Mass Index, SP = Survey Participant.
Data are presented as unweighted n/total (%) based on participants with non-missing haemoglobin, ferritin, and C-reactive protein. No ferritin was collected in males.
No data: No answer/refused, Don't know; Missing: Not applicable, Not asked. If a measurement of one or more of the healthy criteria is missing, then the participant is included in the reference sample.
[1] Ferritin≥15ug/L and C-reactive protein≤5mg/L.

Table 4.5.6 US NHANES 2009-2010: Participant characteristics of violations (Females) (12-17 years).

	Overall sample Female N=422
Reference sample[1]	147/422 (34·8%)
VIOLATION: BMI age and sex dependent	
No	334/422 (79·1%)
Yes	83/422 (19·7%)
No data	5/422 (1·2%)
VIOLATION: Doctor told you have diabetes	
No	421/422 (99·8%)
Yes	1/422 (0·2%)
VIOLATION: Still have asthma	
No	21/422 (5·0%)
Yes	45/422 (10·7%)
Missing	356/422 (84·4%)
VIOLATION: Taking treatment for anaemia/past 3 months	
No	419/422 (99·3%)
Yes	3/422 (0·7%)
VIOLATION: Overnight hospital patient in last year	
No	405/422 (96·0%)
Yes	17/422 (4·0%)
VIOLATION: SP donated blood in past 12 months	
No	122/422 (28·9%)
Yes	15/422 (3·6%)
No data	12/422 (2·8%)
Missing	273/422 (64·7%)
VIOLATION: SP have head cold or chest cold?	
No	319/422 (75·6%)
Yes	75/422 (17·8%)
No data	28/422 (6·6%)
VIOLATION: SP have stomach or intestinal illness?	
No	367/422 (87·0%)
Yes	28/422 (6·6%)
No data	27/422 (6·4%)
VIOLATION: SP have flu, pneumonia, ear infection?	
No	372/422 (88·2%)
Yes	23/422 (5·5%)
No data	27/422 (6·4%)
VIOLATION: Taken prescription medicine, past month	
No	339/422 (80·3%)
Yes	83/422 (19·7%)
VIOLATION: For anaemia, such as low iron	
Yes	3/422 (0·7%)
No data	419/422 (99·3%)
VIOLATION: Ferritin<15ug/L	
No	337/422 (79·9%)
Yes	85/422 (20·1%)
VIOLATION: C-Reactive Protein>5mg/L	
No	403/422 (95·5%)
Yes	19/422 (4·5%)
VIOLATION: eGFR<90 ml/min/1.73m^2	
No	417/422 (98·8%)
Yes	2/422 (0·5%)
No data	3/422 (0·7%)

US = United States, NHANES = National Health and Nutrition Examination Survey, BMI = Body Mass Index, SP = Survey Participant.

Data are presented as unweighted n/total (%) based on participants with non-missing haemoglobin, ferritin, and C-reactive protein. No ferritin was collected in males.

No data: No answer/refused, Don't know; Missing: Not applicable, Not asked. If a measurement of one or more of the healthy criteria is missing, then the participant is included in the reference sample.

[1] Ferritin≥15ug/L and C-reactive protein≤5mg/L.

Table 4.5.7 US NHANES 2015-2016: Participant characteristics of violations (Females) (12-17 years).

	Overall sample Female N=420
Reference sample[1]	154/420 (36·7%)
VIOLATION: BMI age and sex dependent	
No	321/420 (76·4%)
Yes	92/420 (21·9%)
No data	7/420 (1·7%)
VIOLATION: Doctor told you have diabetes	
No	418/420 (99·5%)
Yes	2/420 (0·5%)
VIOLATION: Still have asthma	
No	31/420 (7·4%)
Yes	51/420 (12·1%)
Missing	338/420 (80·5%)
VIOLATION: Taking treatment for anaemia/past 3 months	
No	410/420 (97·6%)
Yes	10/420 (2·4%)
VIOLATION: Ever been told you have jaundice?	
No	417/420 (99·3%)
Yes	2/420 (0·5%)
No data	1/420 (0·2%)
VIOLATION: Overnight hospital patient in last year	
No	408/420 (97·1%)
Yes	12/420 (2·9%)
VIOLATION: SP donated blood in past 12 months	
No	135/420 (32·1%)
Yes	19/420 (4·5%)
No data	6/420 (1·4%)
Missing	260/420 (61·9%)
VIOLATION: SP have head cold or chest cold?	
No	338/420 (80·5%)
Yes	62/420 (14·8%)
No data	20/420 (4·8%)
VIOLATION: SP have stomach or intestinal illness?	
No	366/420 (87·1%)
Yes	34/420 (8·1%)
No data	20/420 (4·8%)
VIOLATION: SP have flu, pneumonia, ear infection?	
No	384/420 (91·4%)
Yes	16/420 (3·8%)
No data	20/420 (4·8%)
VIOLATION: Taken prescription medicine, past month	
No	328/420 (78·1%)
Yes	91/420 (21·7%)
No data	1/420 (0·2%)
VIOLATION: For anaemia, such as low iron	
Yes	8/420 (1·9%)
No data	412/420 (98·1%)
VIOLATION: Ferritin<15ug/L	
No	332/420 (79·0%)
Yes	88/420 (21·0%)
VIOLATION: C-Reactive Protein>5mg/L	
No	394/420 (93·8%)
Yes	26/420 (6·2%)
VIOLATION: eGFR<90 ml/min/1.73m^2	
No	418/420 (99·5%)
Yes	2/420 (0·5%)

US = United States, NHANES = National Health and Nutrition Examination Survey, BMI = Body Mass Index, SP = Survey Participant.
Data are presented as unweighted n/total (%) based on participants with non-missing haemoglobin, ferritin, and C-reactive protein. No ferritin was collected in males.
No data: No answer/refused, Don't know; Missing: Not applicable, Not asked. If a measurement of one or more of the healthy criteria is missing, then the participant is included in the reference sample.
[1] Ferritin≥15ug/L and C-reactive protein≤5mg/L.

Table 4.5.8 US NHANES 2017-2018: Participant characteristics of violations (12-17 years).

	Overall sample	
	**Male N=383**	**Female N=366**
Reference sample[1]	141/383 (36·8%)	109/366 (29·8%)
VIOLATION: BMI age and sex dependent		
No	283/383 (73·9%)	274/366 (74·9%)
Yes	96/383 (25·1%)	88/366 (24·0%)
No data	4/383 (1·0%)	4/366 (1·1%)
VIOLATION: Doctor told you have diabetes		
No	380/383 (99·2%)	362/366 (98·9%)
Yes	3/383 (0·8%)	4/366 (1·1%)
VIOLATION: Still have asthma		
No	31/383 (8·1%)	26/366 (7·1%)
Yes	46/383 (12·0%)	32/366 (8·7%)
No data	2/383 (0·5%)	1/366 (0·3%)
Missing	304/383 (79·4%)	307/366 (83·9%)
VIOLATION: Taking treatment for anaemia/past 3 months		
No	383/383 (100·0%)	353/366 (96·4%)
Yes	0/383 (0·0%)	12/366 (3·3%)
No data	0/383 (0·0%)	1/366 (0·3%)
VIOLATION: Ever told you had any liver condition (age12-19)		
No	382/383 (99·7%)	366/366 (100·0%)
Yes	1/383 (0·3%)	0/366 (0·0%)
VIOLATION: Ever been told you have jaundice?		
No	382/383 (99·7%)	364/366 (99·5%)
Yes	0/383 (0·0%)	1/366 (0·3%)
No data	1/383 (0·3%)	1/366 (0·3%)
VIOLATION: SP donated blood in past 12 months		
No	105/383 (27·4%)	120/366 (32·8%)
Yes	17/383 (4·4%)	15/366 (4·1%)
No data	5/383 (1·3%)	5/366 (1·4%)
Missing	256/383 (66·8%)	226/366 (61·7%)
VIOLATION: Overnight hospital patient in last year		
No	376/383 (98·2%)	358/366 (97·8%)
Yes	7/383 (1·8%)	8/366 (2·2%)
VIOLATION: SP have head cold or chest cold?		
No	320/383 (83·6%)	299/366 (81·7%)
Yes	48/383 (12·5%)	55/366 (15·0%)
No data	15/383 (3·9%)	12/366 (3·3%)
VIOLATION: SP have stomach or intestinal illness?		
No	343/383 (89·6%)	336/366 (91·8%)
Yes	25/383 (6·5%)	21/366 (5·7%)
No data	15/383 (3·9%)	9/366 (2·5%)
VIOLATION: SP have flu, pneumonia, ear infection?		
No	351/383 (91·6%)	341/366 (93·2%)
Yes	17/383 (4·4%)	15/366 (4·1%)
No data	15/383 (3·9%)	10/366 (2·7%)
VIOLATION: For anaemia, such as low iron		
No	382/383 (99·7%)	358/366 (97·8%)
Yes	1/383 (0·3%)	8/366 (2·2%)
VIOLATION: Ferritin<15ug/L		
No	367/383 (95·8%)	287/366 (78·4%)
Yes	16/383 (4·2%)	79/366 (21·6%)
VIOLATION: C-Reactive Protein>5mg/L		
No	351/383 (91·6%)	339/366 (92·6%)
Yes	32/383 (8·4%)	27/366 (7·4%)
VIOLATION: eGFR<90 ml/min/1.73m^2		
No	380/383 (99·2%)	361/366 (98·6%)
Yes	2/383 (0·5%)	3/366 (0·8%)
No data	1/383 (0·3%)	2/366 (0·5%)

US = United States, NHANES = National Health and Nutrition Examination Survey, BMI = Body Mass Index, SP = Survey Participant.
Data are presented as unweighted n/total (%) based on participants with non-missing haemoglobin, ferritin, and C-reactive protein.
No data: No answer/refused, Don't know; Missing: Not applicable, Not asked. If a measurement of one or more of the healthy criteria is missing, then the participant is included in the reference sample.

[1] Ferritin$\geq$15ug/L and C-reactive protein$\leq$5mg/L.

Table 4.5.9 AUS NHS 2011-2012: Participant characteristics of violations (12-17 years).

	Overall sample	
	**Male N=209**	**Female N=243**
Reference sample[1]		
No	119/209 (56·9%)	164/243 (67·5%)
Yes	90/209 (43·1%)	79/243 (32·5%)
VIOLATION: BMI violation*		
No	172/209 (82·3%)	189/243 (77·8%)
Yes	23/209 (11·0%)	37/243 (15·2%)
Missing	14/209 (6·7%)	17/243 (7·0%)
VIOLATION: Co-morbidities		
No	176/209 (84·2%)	202/243 (83·1%)
Yes	33/209 (15·8%)	41/243 (16·9%)
VIOLATION: Dialysis		
No	209/209 (100·0%)	243/243 (100·0%)
VIOLATION: Hospital last 12 months		
No	Not displayed†	Not displayed†
Yes	<10/209 (<4·8%)	<10/243 (<4·1%)
VIOLATION: Hospital last 2 weeks		
No	Not displayed†	Not displayed†
Yes	<10/209 (<4·8%)	<10/243 (<4·1%)
VIOLATION: Time off work or study due to illness		
No	162/206 (78·6%)	184/235 (78·3%)
Yes	44/206 (21·4%)	51/235 (21·7%)
VIOLATION: Time off work due to illness		
No	Not displayed†	Not displayed†
Yes	<10/209 (<4·8%)	<10/243 (<4·1%)
Missing	169/209 (80·9%)	168/243 (69·1%)
VIOLATION: Time off study due to illness		
No	161/204 (78·9%)	181/229 (79·0%)
Yes	43/204 (21·1%)	48/229 (21·0%)
VIOLATION: Medication		
No	153/209 (73·2%)	176/243 (72·4%)
Yes	56/209 (26·8%)	67/243 (27·6%)
VIOLATION: Smoking		
No	Not displayed†	133/243 (54·7%)
Yes	<10/209 (<4·8%)	12/243 (4·9%)
Missing	105/209 (50·2%)	98/243 (40·3%)
VIOLATION: Alcohol		
No	Not displayed†	Not displayed†
Yes	<10/209 (<4·8%)	<10/243 (<4·1%)
Missing	189/209 (90·4%)	218/243 (89·7%)
VIOLATION: Ferritin<15ug/L		
No	Not displayed†	Not displayed†
Yes	<10/209 (<4·8%)	34/243 (14·0%)
VIOLATION: C-Reactive Protein>5mg/L		
No	197/209 (94·3%)	230/243 (94·7%)
Yes	12/209 (5·7%)	13/243 (5·3%)

AUS = Australia, NHS = National Health Survey, BMI = Body Mass Index.
Data are presented as unweighted n/total (%) based on participants with non-missing haemoglobin, ferritin, and C-reactive protein.
If a measurement of one or more of the healthy criteria is missing, then the participant is included in the reference sample.
*BMI – underweight class 1/2/3 and obese class 1.
† Not displayed due to Australian Bureau of Statistics requirements for output clearance.
[1] Ferritin≥15ug/L and C-reactive protein≤5mg/L.

Table 4.5.10 AUS NNPAS 2011-2012: Participant characteristics of violations (12-17 years).

	Overall sample	
	Male<br>N=149	Female<br>N=133
Reference sample[1]		
No	32/149 (21·5%)	39/133 (29·3%)
Yes	117/149 (78·5%)	94/133 (70·7%)
VIOLATION: BMI violation*		
No	Not displayed†	Not displayed†
Yes	11/149 (7·4%)	20/133 (15·0%)
Missing	<10	<10
VIOLATION: Co-morbidities		
No	Not displayed†	Not displayed†
Yes	<10/149 (<6·7%)	<10/133 (<7·5%)
VIOLATION: Smoking		
No	Not displayed†	Not displayed†
Yes	<10/149 (<6·7%)	<10/133 (<7·5%)
Missing	69/149 (46·3%)	64/133 (48·1%)
VIOLATION: Ferritin<15ug/L		
No	Not displayed†	120/133 (90·2%)
Yes	<10/149 (<6·7%)	13/133 (9·8%)
VIOLATION: C-Reactive Protein>5mg/L		
No	136/149 (91·3%)	Not displayed†
Yes	13/149 (8·7%)	<10/133 (<7·5%)

AUS = Australia, NNPAS = National Nutrition and Physical Activity Survey, BMI = Body Mass Index.
Data are presented as unweighted n/total (%) based on participants with non-missing haemoglobin, ferritin, and C-reactive protein.
If a measurement of one or more of the healthy criteria is missing, then the participant is included in the reference sample.
*BMI – underweight class 1/2/3 and obese class 1.
† Not displayed due to Australian Bureau of Statistics requirements for output clearance.
[1] Ferritin≥15ug/L and C-reactive protein≤5mg/L.

Table 4.5.11 CHN CHNS 2009: Participant characteristics of violations (12-17 years).

	Overall sample	
	**Male N=223**	**Female N=187**
Reference sample[1]	130/223 (58·3%)	102/187 (54·5%)
VIOLATION: BMI age and sex dependent		
No	186/223 (83·4%)	171/187 (91·4%)
Yes	23/223 (10·3%)	13/187 (7·0%)
Missing	14/223 (6·3%)	3/187 (1·6%)
VIOLATION: Illness/Injury - Infectious/parasitic disease		
No	15/223 (6·7%)	12/187 (6·4%)
Missing	208/223 (93·3%)	175/187 (93·6%)
VIOLATION: Illness/Injury - Heart disease		
No	14/223 (6·3%)	12/187 (6·4%)
Yes	1/223 (0·4%)	0/187 (0·0%)
Missing	208/223 (93·3%)	175/187 (93·6%)
VIOLATION: Illness/Injury - Tumor		
No	15/223 (6·7%)	12/187 (6·4%)
Missing	208/223 (93·3%)	175/187 (93·6%)
VIOLATION: Illness/Injury - Respiratory disease		
No	10/223 (4·5%)	6/187 (3·2%)
Yes	5/223 (2·2%)	6/187 (3·2%)
Missing	208/223 (93·3%)	175/187 (93·6%)
VIOLATION: Illness/Injury - Injury		
No	14/223 (6·3%)	12/187 (6·4%)
Yes	1/223 (0·4%)	0/187 (0·0%)
Missing	208/223 (93·3%)	175/187 (93·6%)
VIOLATION: Illness/Injury - Alcohol poisoning		
No	15/223 (6·7%)	12/187 (6·4%)
Missing	208/223 (93·3%)	175/187 (93·6%)
VIOLATION: Illness/Injury - Endocrine disorder		
No	15/223 (6·7%)	12/187 (6·4%)
Missing	208/223 (93·3%)	175/187 (93·6%)
VIOLATION: Illness/Injury - Hematological disease		
No	15/223 (6·7%)	12/187 (6·4%)
Missing	208/223 (93·3%)	175/187 (93·6%)
VIOLATION: Illness/Injury - Mental/psychiatric disorder		
No	15/223 (6·7%)	12/187 (6·4%)
Missing	208/223 (93·3%)	175/187 (93·6%)
VIOLATION: Illness/Injury - Neurological disorder		
No	15/223 (6·7%)	12/187 (6·4%)
Missing	208/223 (93·3%)	175/187 (93·6%)
VIOLATION: Illness/Injury - Eye/ear/nose/throat/teeth disease		
No	14/223 (6·3%)	12/187 (6·4%)
Yes	1/223 (0·4%)	0/187 (0·0%)
Missing	208/223 (93·3%)	175/187 (93·6%)
VIOLATION: Illness/Injury - Digestive disease		
No	14/223 (6·3%)	10/187 (5·3%)
Yes	1/223 (0·4%)	2/187 (1·1%)
Missing	208/223 (93·3%)	175/187 (93·6%)
VIOLATION: Illness/Injury - Urinary disease		
No	15/223 (6·7%)	12/187 (6·4%)
Missing	208/223 (93·3%)	175/187 (93·6%)
VIOLATION: Illness/Injury - Sexual dysfunction		
No	15/223 (6·7%)	12/187 (6·4%)
Missing	208/223 (93·3%)	175/187 (93·6%)
VIOLATION: Illness/Injury - Obstetrical/gynecological disease		
No	15/223 (6·7%)	12/187 (6·4%)
Missing	208/223 (93·3%)	175/187 (93·6%)
VIOLATION: Illness/Injury - Dermatological disease		
No	14/223 (6·3%)	12/187 (6·4%)
Yes	1/223 (0·4%)	0/187 (0·0%)
Missing	208/223 (93·3%)	175/187 (93·6%)
VIOLATION: Illness/Injury - Muscular/rheumatological disease		
No	15/223 (6·7%)	12/187 (6·4%)
Missing	208/223 (93·3%)	175/187 (93·6%)
VIOLATION: Diagnosed with high blood pressure		
No	217/223 (97·3%)	186/187 (99·5%)
No data	0/223 (0·0%)	1/187 (0·5%)

	Overall sample	
	Male<br>N=223	Female<br>N=187
Missing	6/223 (2·7%)	0/187 (0·0%)
VIOLATION: Diagnosed with diabetes		
No	217/223 (97·3%)	187/187 (100·0%)
Missing	6/223 (2·7%)	0/187 (0·0%)
VIOLATION: Diagnosed with myocardial infarction		
No	1/223 (0·4%)	2/187 (1·1%)
Missing	222/223 (99·6%)	185/187 (98·9%)
VIOLATION: Diagnosed with apoplexy		
No	1/223 (0·4%)	2/187 (1·1%)
Missing	222/223 (99·6%)	185/187 (98·9%)
VIOLATION: Dr. told you that you suffer from asthma		
No	1/223 (0·4%)	2/187 (1·1%)
Missing	222/223 (99·6%)	185/187 (98·9%)
VIOLATION: Past 12 months - had whistling/wheezing in chest		
Missing	223/223 (100·0%)	187/187 (100·0%)
VIOLATION: Last 4 weeks - been sick or injured		
No	201/223 (90·1%)	169/187 (90·4%)
Yes	17/223 (7·6%)	14/187 (7·5%)
No data	0/223 (0·0%)	2/187 (1·1%)
Missing	5/223 (2·2%)	2/187 (1·1%)
VIOLATION: Last 4 weeks - fever, sore throat, cough		
No	202/223 (90·6%)	164/187 (87·7%)
Yes	18/223 (8·1%)	20/187 (10·7%)
No data	0/223 (0·0%)	2/187 (1·1%)
Missing	3/223 (1·3%)	1/187 (0·5%)
VIOLATION: Last 4 weeks - diarrhea, stomachache		
No	216/223 (96·9%)	178/187 (95·2%)
Yes	4/223 (1·8%)	5/187 (2·7%)
No data	0/223 (0·0%)	3/187 (1·6%)
Missing	3/223 (1·3%)	1/187 (0·5%)
VIOLATION: Last 4 weeks - stomachache		
No	219/223 (98·2%)	181/187 (96·8%)
Yes	1/223 (0·4%)	4/187 (2·1%)
No data	0/223 (0·0%)	2/187 (1·1%)
Missing	3/223 (1·3%)	0/187 (0·0%)
VIOLATION: Last 4 weeks - asthma		
No	220/223 (98·7%)	184/187 (98·4%)
Yes	0/223 (0·0%)	1/187 (0·5%)
No data	0/223 (0·0%)	2/187 (1·1%)
Missing	3/223 (1·3%)	0/187 (0·0%)
VIOLATION: Last 4 weeks - headache, dizziness		
No	216/223 (96·9%)	177/187 (94·7%)
Yes	4/223 (1·8%)	7/187 (3·7%)
No data	0/223 (0·0%)	2/187 (1·1%)
Missing	3/223 (1·3%)	1/187 (0·5%)
VIOLATION: Last 4 weeks - joint, muscle pain		
No	217/223 (97·3%)	183/187 (97·9%)
Yes	3/223 (1·3%)	1/187 (0·5%)
No data	0/223 (0·0%)	2/187 (1·1%)
Missing	3/223 (1·3%)	1/187 (0·5%)
VIOLATION: Last 4 weeks - rash, dermatitis		
No	219/223 (98·2%)	183/187 (97·9%)
Yes	1/223 (0·4%)	1/187 (0·5%)
No data	0/223 (0·0%)	2/187 (1·1%)
Missing	3/223 (1·3%)	1/187 (0·5%)
VIOLATION: Last 4 weeks - eye/ear disease		
No	220/223 (98·7%)	183/187 (97·9%)
Yes	0/223 (0·0%)	1/187 (0·5%)
No data	0/223 (0·0%)	2/187 (1·1%)
Missing	3/223 (1·3%)	1/187 (0·5%)
VIOLATION: Last 4 weeks - heart disease/chest pain		
No	220/223 (98·7%)	184/187 (98·4%)
No data	0/223 (0·0%)	2/187 (1·1%)
Missing	3/223 (1·3%)	1/187 (0·5%)
VIOLATION: Last 4 weeks - other infectious disease		
No	220/223 (98·7%)	183/187 (97·9%)
No data	0/223 (0·0%)	2/187 (1·1%)

	Overall sample	
	Male<br>N=223	Female<br>N=187
Missing	3/223 (1·3%)	2/187 (1·1%)
VIOLATION: Last 4 weeks - noncommunicable disease		
No	219/223 (98·2%)	182/187 (97·3%)
Yes	1/223 (0·4%)	1/187 (0·5%)
No data	0/223 (0·0%)	2/187 (1·1%)
Missing	3/223 (1·3%)	2/187 (1·1%)
VIOLATION: Last 4 weeks - days hospitalised		
Missing	223/223 (100·0%)	187/187 (100·0%)
VIOLATION: Taking anti-hypertension drugs		
Missing	223/223 (100·0%)	187/187 (100·0%)
VIOLATION: Province – Guizhou – Altitude		
No	198/223 (88·8%)	166/187 (88·8%)
Yes	25/223 (11·2%)	21/187 (11·2%)
VIOLATION: still smokes cigarettes		
No	4/223 (1·8%)	0/187 (0·0%)
Yes	9/223 (4·0%)	0/187 (0·0%)
No data	210/223 (94·2%)	187/187 (100·0%)
VIOLATION: Alcohol consumption almost every day		
No	24/223 (10·8%)	7/187 (3·7%)
Yes	0/223 (0·0%)	1/187 (0·5%)
Missing	199/223 (89·2%)	179/187 (95·7%)
VIOLATION (# beer bottles per week): >6 for women and >8 for men		
No	23/223 (10·3%)	3/187 (1·6%)
Missing	200/223 (89·7%)	184/187 (98·4%)
VIOLATION (# wine drinks per week): >6 for women and >8 for men		
No	8/223 (3·6%)	5/187 (2·7%)
Yes	0/223 (0·0%)	1/187 (0·5%)
Missing	215/223 (96·4%)	181/187 (96·8%)
VIOLATION (# liquor drinks per week): >6 for women and >8 for men		
No	9/223 (4·0%)	2/187 (1·1%)
Missing	214/223 (96·0%)	185/187 (98·9%)
VIOLATION (# beer, wine, liquor): >6 for women and >8 for men		
No	23/223 (10·3%)	7/187 (3·7%)
Yes	1/223 (0·4%)	1/187 (0·5%)
Missing	199/223 (89·2%)	179/187 (95·7%)
VIOLATION: Ferritin<15ug/L		
No	213/223 (95·5%)	157/187 (84·0%)
Yes	10/223 (4·5%)	30/187 (16·0%)
VIOLATION: C-Reactive Protein>5mg/L		
No	208/223 (93·3%)	179/187 (95·7%)
Yes	15/223 (6·7%)	8/187 (4·3%)

CHN = China, CHNS = China Health and Nutrition Survey, BMI = Body Mass Index
Data are presented as unweighted n/total (%) based on participants with non-missing haemoglobin, ferritin, and C-reactive protein.
No data: No answer/refused, Don't know; Missing: Not applicable, Not asked. If a measurement of one or more of the healthy criteria is missing, then the participant is included in the reference sample.
[1] Ferritin≥15ug/L and C-reactive protein≤5mg/L.

4.6 Detailed Individual Exclusions Pregnant women

Table 4.6.1 NL Generation R: Participant characteristics of violations (Trimester 1) (18-45 years).

	**Overall sample Female N = 5,054**
Reference sample[1]	
No	4,279 / 5,054 (84·7%)
Yes	775 / 5,054 (15·3%)
VIOLATION: Outcome pregnancy not live birth singleton or missing	
No	4,904 / 5,054 (97·0%)
Yes	150 / 5,054 (3·0%)
VIOLATION: PIH, PE, HELLP, Eclampsia, superimposed PE/HELLP, preexisting RR (from medical records) or missing	
No	4,562 / 5,054 (90·3%)
Yes	492 / 5,054 (9·7%)
VIOLATION: Preeclampsia or missing	
No	4,562 / 5,054 (90·3%)
Yes	492 / 5,054 (9·7%)
VIOLATION: Preexisting hypertension (based on questionnaire and medical records) or missing	
No	4,307 / 5,054 (85·2%)
Yes	747 / 5,054 (14·8%)
VIOLATION: Pregnancy Induced Hypertension (positive PE_total excluded) or missing	
No	4,562 / 5,054 (90·3%)
Yes	492 / 5,054 (9·7%)
VIOLATION: Ferritin (<15ug/L or >150ug/L) or missing	
No	4,098 / 5,054 (81·1%)
Yes	956 / 5,054 (18·9%)
VIOLATION: Folic acid plasma mother <18 weeks (≤6.0nmol/l) or missing	
No	4,505 / 5,054 (89·1%)
Yes	549 / 5,054 (10·9%)
VIOLATION: Active vitamin B12 serum mother <18 weeks (≤23pmol/L) or missing	
No	3,088 / 5,054 (61·1%)
Yes	1,966 / 5,054 (38·9%)
VIOLATION: C-reactive protein plasma mother <18 weeks (>5mg/L) or missing	
No	2,601 / 5,054 (51·5%)
Yes	2,453 / 5,054 (48·5%)
VIOLATION: Body Mass Index first trimester <18.5kg/m^2 or >30kg/m^2 or missing	
No	4,307 / 5,054 (85·2%)
Yes	747 / 5,054 (14·8%)
VIOLATION: Smoked till pregnancy was known or continued smoking in pregnancy or missing	
No	3,244 / 5,054 (64·2%)
Yes	1,810 / 5,054 (35·8%)

NL = The Netherlands.
Data are presented as n/total (%) based on participants with participants with non-missing haemoglobin in first trimester of pregnancy.
PIH = Pregnancy Induced Hypertension, PE = Pulmonary Embolus, HELLP = Hemolysis, Elevated Liver enzymes and Low Platelets, RR = …
[1] Ferritin≥15 and ≤150 ug/L and C-reactive protein≤5mg/L (both first trimester for pregnancy). If a measurement of one or more of the healthy criteria is missing, then the participant is excluded from the reference sample.

Table 4.6.2 NL Generation R: Participant characteristics of violations (Trimester 2) (18-45 years).

	**Overall sample Female N = 2,194**
Reference sample[1]	
No	2,083 / 2,194 (94·9%)
Yes	111 / 2,194 (5·1%)
VIOLATION: Outcome pregnancy not live birth singleton or missing	
No	2,144 / 2,194 (97·7%)
Yes	50 / 2,194 (2·3%)
VIOLATION: PIH, PE, HELLP, Eclampsia, superimposed PE/HELLP, preexisting RR (from medical records) or missing	
No	1,968 / 2,194 (89·7%)
Yes	226 / 2,194 (10·3%)
VIOLATION: Preeclampsia or missing	
No	1,968 / 2,194 (89·7%)
Yes	226 / 2,194 (10·3%)
VIOLATION: Preexisting hypertension (based on questionnaire and medical records) or missing	
No	1,776 / 2,194 (80·9%)
Yes	418 / 2,194 (19·1%)
VIOLATION: Pregnancy Induced Hypertension (positive PE_total excluded) or missing	
No	1,968 / 2,194 (89·7%)
Yes	226 / 2,194 (10·3%)
VIOLATION: Ferritin (<15ug/L or >150ug/L) or missing	
No	567 / 2,194 (25·8%)
Yes	1,627 / 2,194 (74·2%)
VIOLATION: Folic acid plasma mother <18 weeks (≤6.0nmol/l) or missing	
No	607 / 2,194 (27·7%)
Yes	1,587 / 2,194 (72·3%)
VIOLATION: Active vitamin B12 serum mother <18 weeks (≤23pmol/L) or missing	
No	423 / 2,194 (19·3%)
Yes	1,771 / 2,194 (80·7%)
VIOLATION: C-reactive protein plasma mother <18 weeks (>5mg/L) or missing	
No	373 / 2,194 (17·0%)
Yes	1,821 / 2,194 (83·0%)
VIOLATION: Body Mass Index first trimester <18.5kg/m^2 or >30kg/m^2 or missing	
No	684 / 2,194 (31·2%)
Yes	1,510 / 2,194 (68·8%)
VIOLATION: Smoked till pregnancy was known or continued smoking in pregnancy or missing	
No	1,368 / 2,194 (62·4%)
Yes	826 / 2,194 (37·6%)

NL = The Netherlands.
Data are presented as n/total (%) based on participants with non-missing haemoglobin in second trimester of pregnancy.
PIH = Pregnancy Induced Hypertension, PE = Pulmonary Embolus, HELLP = Hemolysis, Elevated Liver enzymes and Low Platelets, RR = Pre-existing Hypertension
[1] Ferritin≥15 and ≤150 ug/L and C-reactive protein≤5mg/L (both first trimester for pregnancy). If a measurement of one or more of the healthy criteria is missing, then the participant is excluded from the reference sample.

Table 4.6.3 NL Generation R: Participant characteristics of violations (Trimester 3) (18-45 years).

	Overall sample<br>Female<br>N = 239
Reference sample[1]	
No	238 / 239 (99·6%)
Yes	1 / 239 (0·4%)
VIOLATION: Outcome pregnancy not live birth singleton or missing	
No	232.0 / 239.0 (97·1%)
Yes	7.0 / 239.0 (2·9%)
VIOLATION: PIH, PE, HELLP, Eclampsia, superimposed PE/HELLP, preexisting RR (from medical records) or missing	
No	211.0 / 239.0 (88·3%)
Yes	28.0 / 239.0 (11·7%)
VIOLATION: Preeclampsia or missing	
No	211.0 / 239.0 (88·3%)
Yes	28.0 / 239.0 (11·7%)
VIOLATION: Preexisting hypertension (based on questionnaire and medical records) or missing	
No	159.0 / 239.0 (66·5%)
Yes	80.0 / 239.0 (33·5%)
VIOLATION: Pregnancy Induced Hypertension (positive PE_total excluded) or missing	
No	211.0 / 239.0 (88·3%)
Yes	28.0 / 239.0 (11·7%)
VIOLATION: Ferritin (<15ug/L or >150ug/L) or missing	
No	6.0 / 239.0 (2·5%)
Yes	233.0 / 239.0 (97·5%)
VIOLATION: Folic acid plasma mother <18 weeks (≤6.0nmol/l) or missing	
No	6.0 / 239.0 (2·5%)
Yes	233.0 / 239.0 (97·5%)
VIOLATION: Active vitamin B12 serum mother <18 weeks (≤23pmol/L) or missing	
No	4.0 / 239.0 (1·7%)
Yes	235.0 / 239.0 (98·3%)
VIOLATION: C-reactive protein plasma mother <18 weeks (>5mg/L) or missing	
No	4.0 / 239.0 (1·7%)
Yes	235.0 / 239.0 (98·3%)
VIOLATION: Body Mass Index first trimester <18.5kg/m^2 or >30kg/m^2 or missing	
No	7.0 / 239.0 (2·9%)
Yes	232.0 / 239.0 (97·1%)
VIOLATION: Smoked till pregnancy was known or continued smoking in pregnancy or missing	
No	130.0 / 239.0 (54·4%)
Yes	109.0 / 239.0 (45·6%)

NL = The Netherlands.
Data are presented as n/total (%) based on participants with participants with non-missing haemoglobin in third trimester of pregnancy.
PIH = Pregnancy Induced Hypertension, PE = Pulmonary Embolus, HELLP = Hemolysis, Elevated Liver enzymes and Low Platelets, RR = …
[1] Ferritin≥15 and ≤150 ug/L and C-reactive protein≤5mg/L (both first trimester for pregnancy). If a measurement of one or more of the healthy criteria is missing, then the participant is excluded from the reference sample.

5. Discrete Thresholds

Figure 5.1: Adults (18-65 years): 2·5th centile (90% CI).

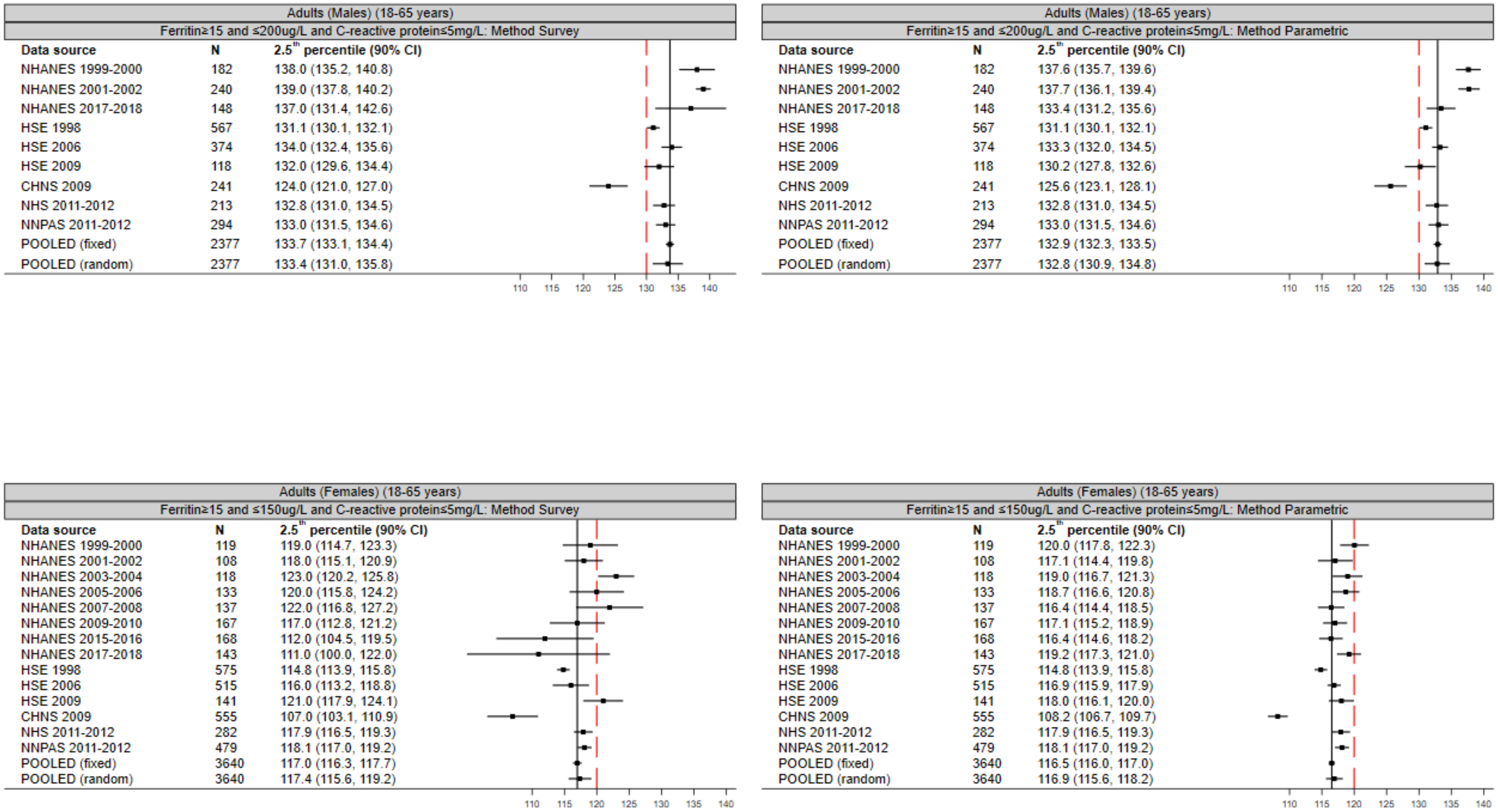

Values are haemoglobin concentration (g/L). CI = Confidence Interval. Red line = cut-off anaemia according to WHO guideline (2011). Black line = pooled estimate of fixed-effect meta-analysis. NHS & NNPAS centile not displayed if sample size less than 200, as per Australian Bureau of Statistics requirements for output clearance.

Figure 5.2: Children (6-23 months, 24-59 months, 5-11 years): 2·5th centile (90% CI).

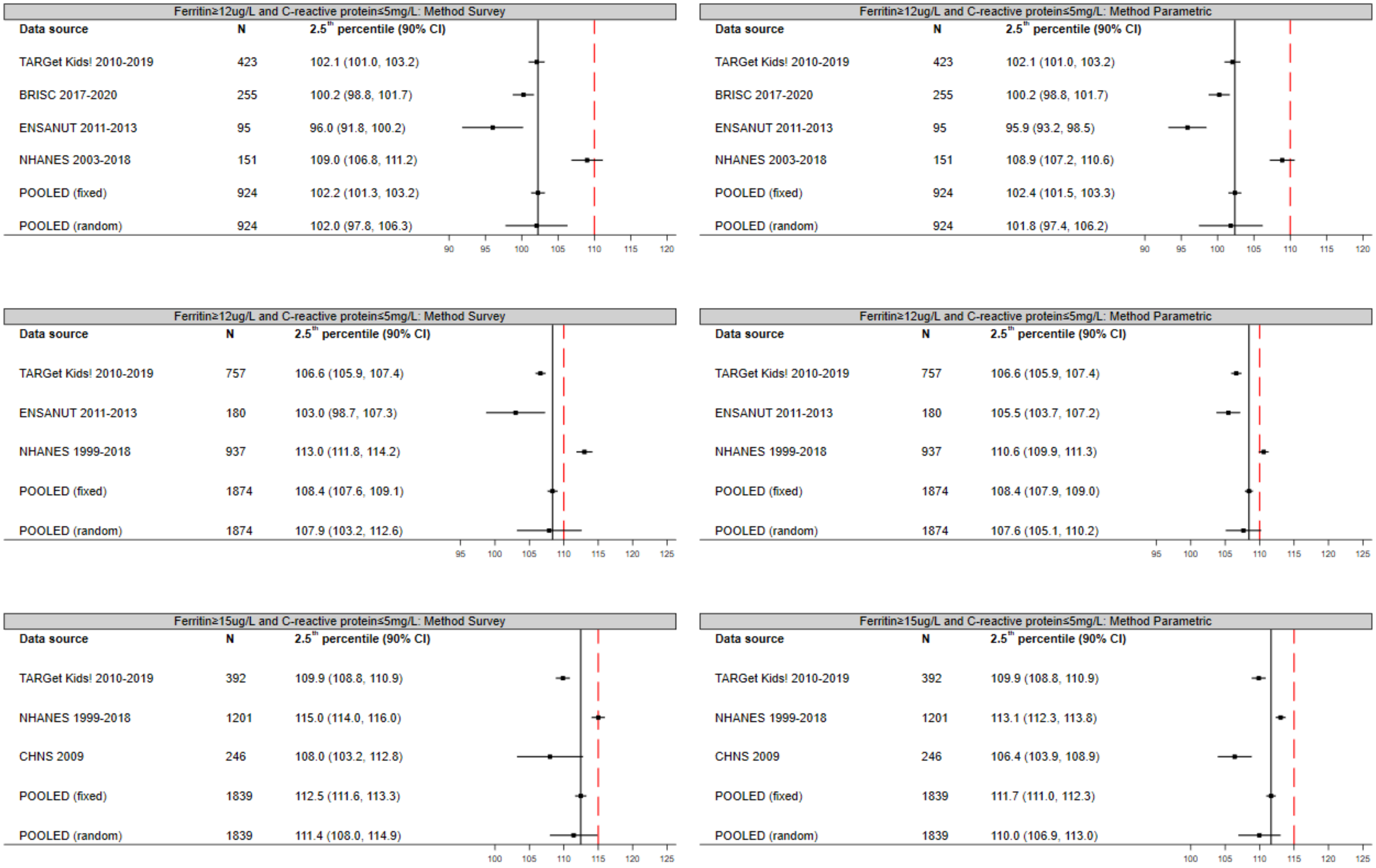

Values are haemoglobin concentration (g/L). CI = Confidence Interval. Red line = cut-off anaemia according to WHO guideline (2011). Black line = pooled estimate of fixed-effect meta-analysis.

Figure 5.3: Adolescents (12-17 years): 2·5th centile (90% CI).

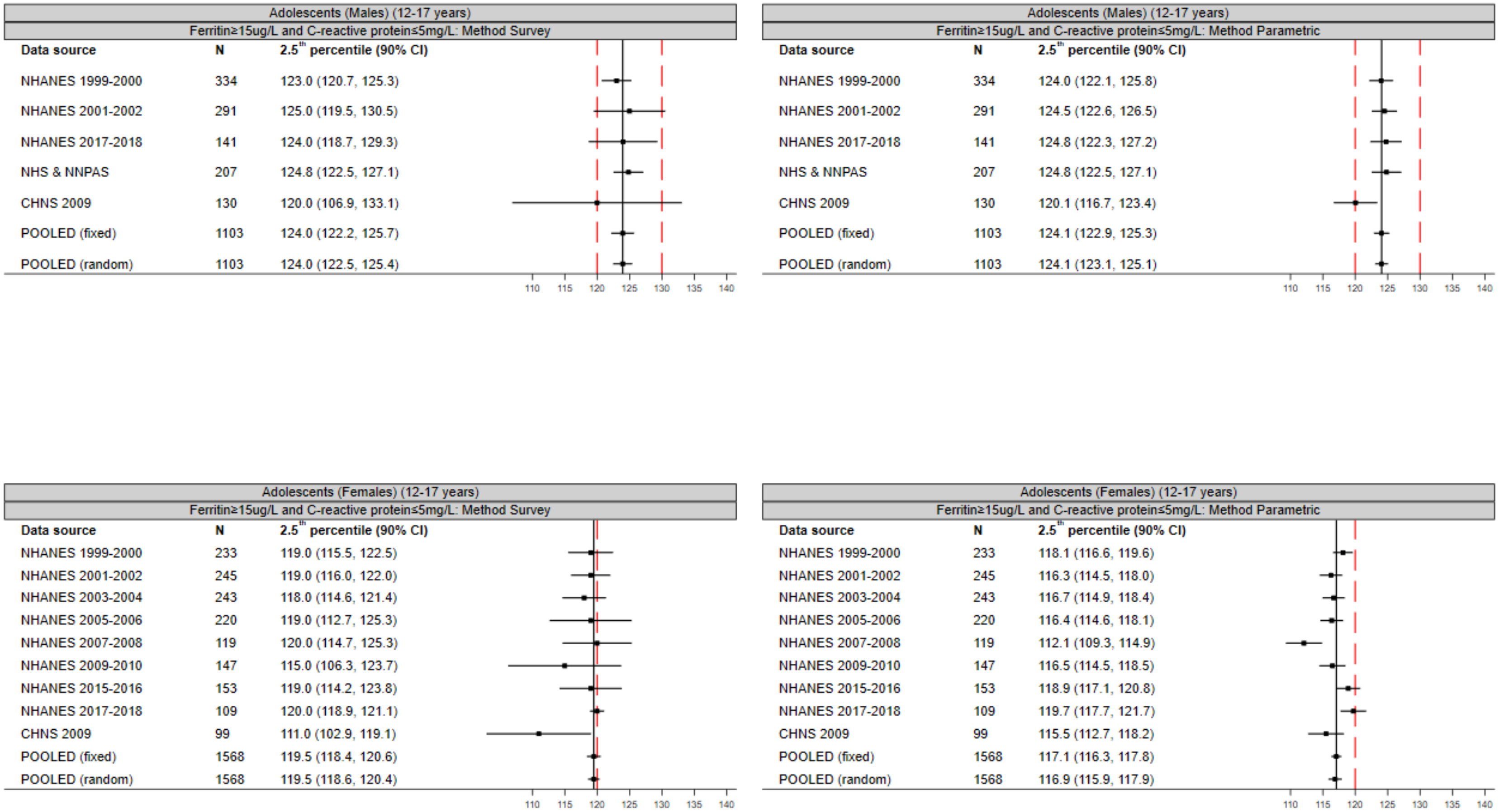

Values are haemoglobin concentration (g/L). CI = Confidence Interval. Red line = cut-off anaemia according to WHO guideline (2011). For males, the cut-off depends on age. Black line = pooled estimate of fixed-effect meta-analysis. NHS & NNPAS centile not displayed if sample size less than 200, as per Australian Bureau of Statistics requirements for output clearance.

Figure 5.4: Sensitivity Analyses Adults (Males) (18-65 years): 5th centile (90% CI).

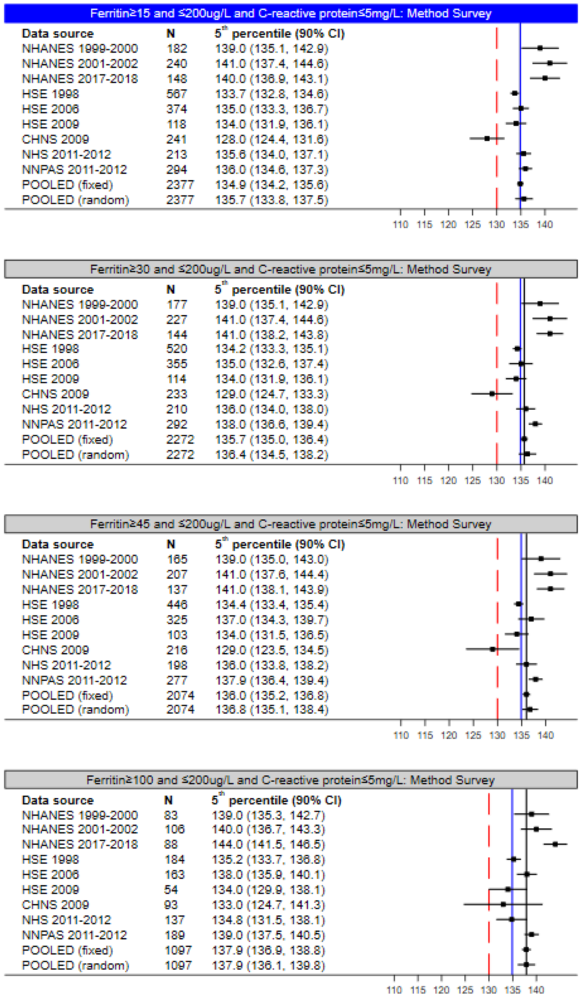

Values are haemoglobin concentration (g/L). CI = Confidence Interval. Red line = cut-off anaemia according to WHO guideline (2011). Blue line = pooled estimate of fixed-effect meta-analysis of primary definition and analysis method (top left figure). Black line = pooled estimate of fixed-effect meta-analysis. If N<20, no centile is derived. NHS or NNPAS centile not displayed if sample size less than 100, as per Australian Bureau of Statistics requirements for output clearance.

Figure 5.5: Sensitivity Analyses Adults (Females) (18-65 years): 5th centile (90% CI).

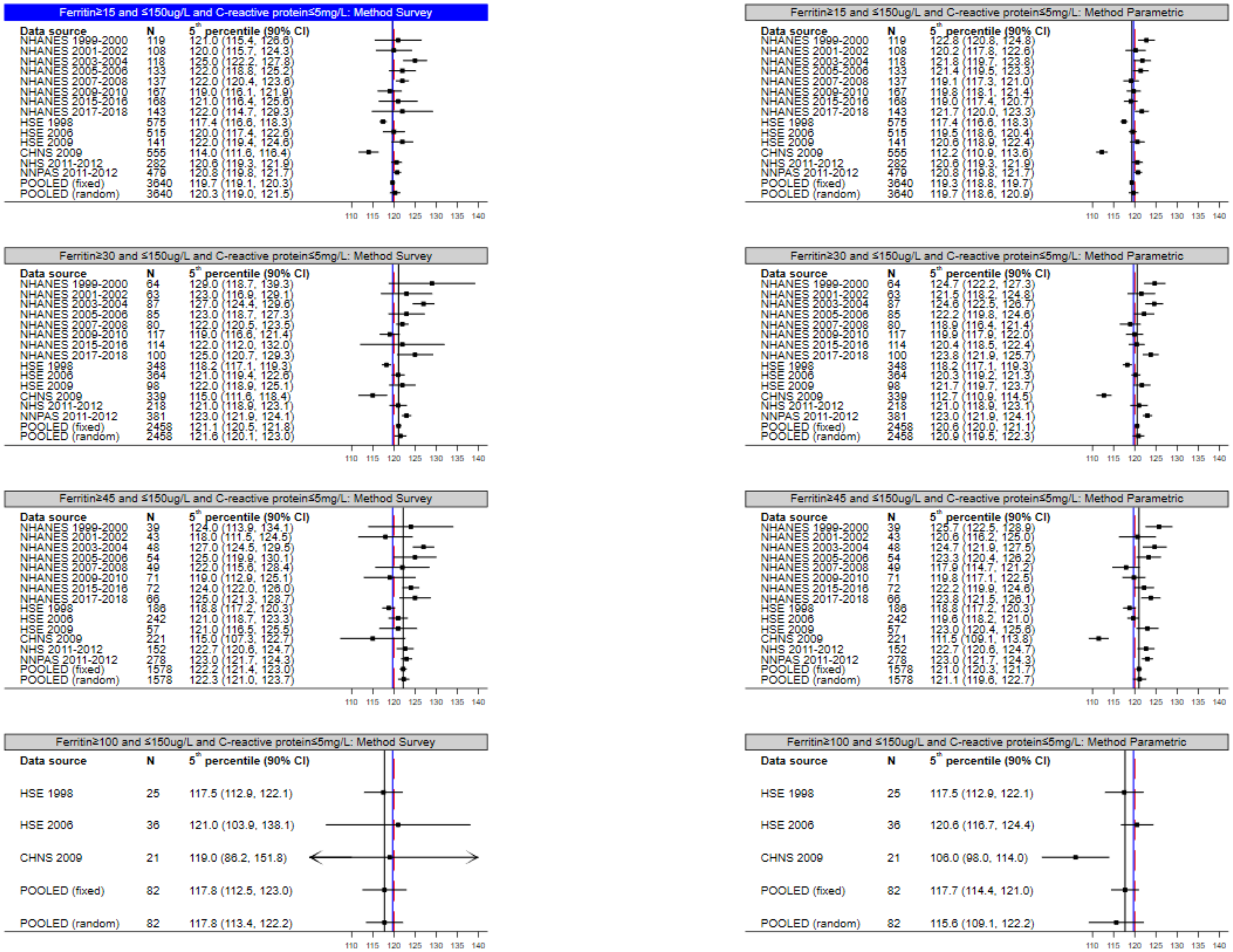

Values are haemoglobin concentration (g/L). CI = Confidence Interval. Red line = cut-off anaemia according to WHO guideline (2011). Blue line = pooled estimate of fixed-effect meta-analysis of primary definition and analysis method (top left figure). Black line = pooled estimate of fixed-effect meta-analysis. If N<20, no centile is derived. NHS or NNPAS centile not displayed if sample size less than 100, as per Australian Bureau of Statistics requirements for output clearance.

Figure 5.6: Sensitivity Analyses Children (6-23 months): 5th centile (90% CI) (Part 1).

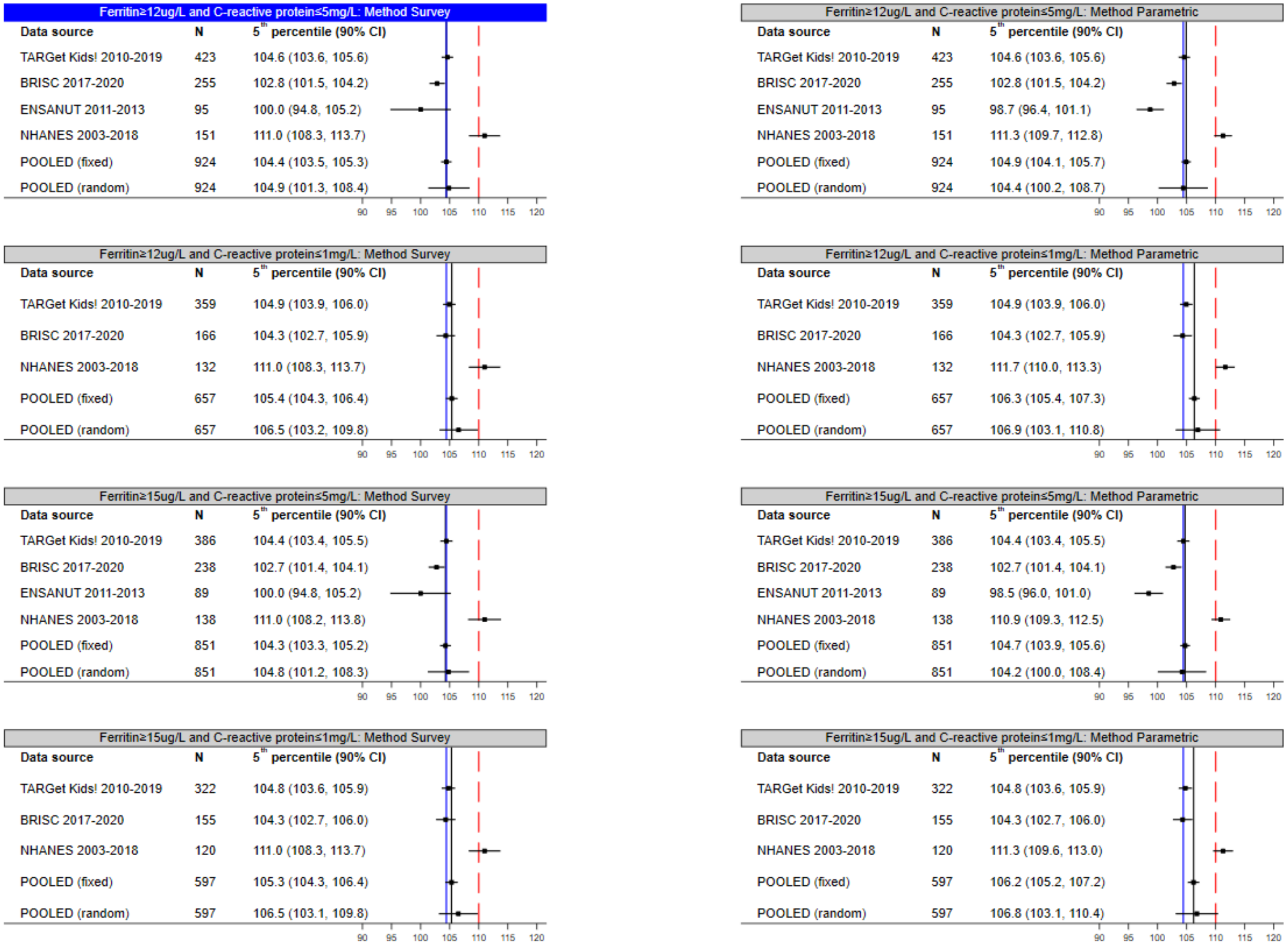

Values are haemoglobin concentration (g/L). CI = Confidence Interval. Red line = cut-off anaemia according to WHO guideline (2011). Blue line = pooled estimate of fixed-effect meta-analysis of primary definition and analysis method (top left figure). Black line = pooled estimate of fixed-effect meta-analysis. If N<20, no centile is derived.

Figure 5.7: Sensitivity Analyses Children (6-23 months): 5th centile (90% CI) (Part 2).

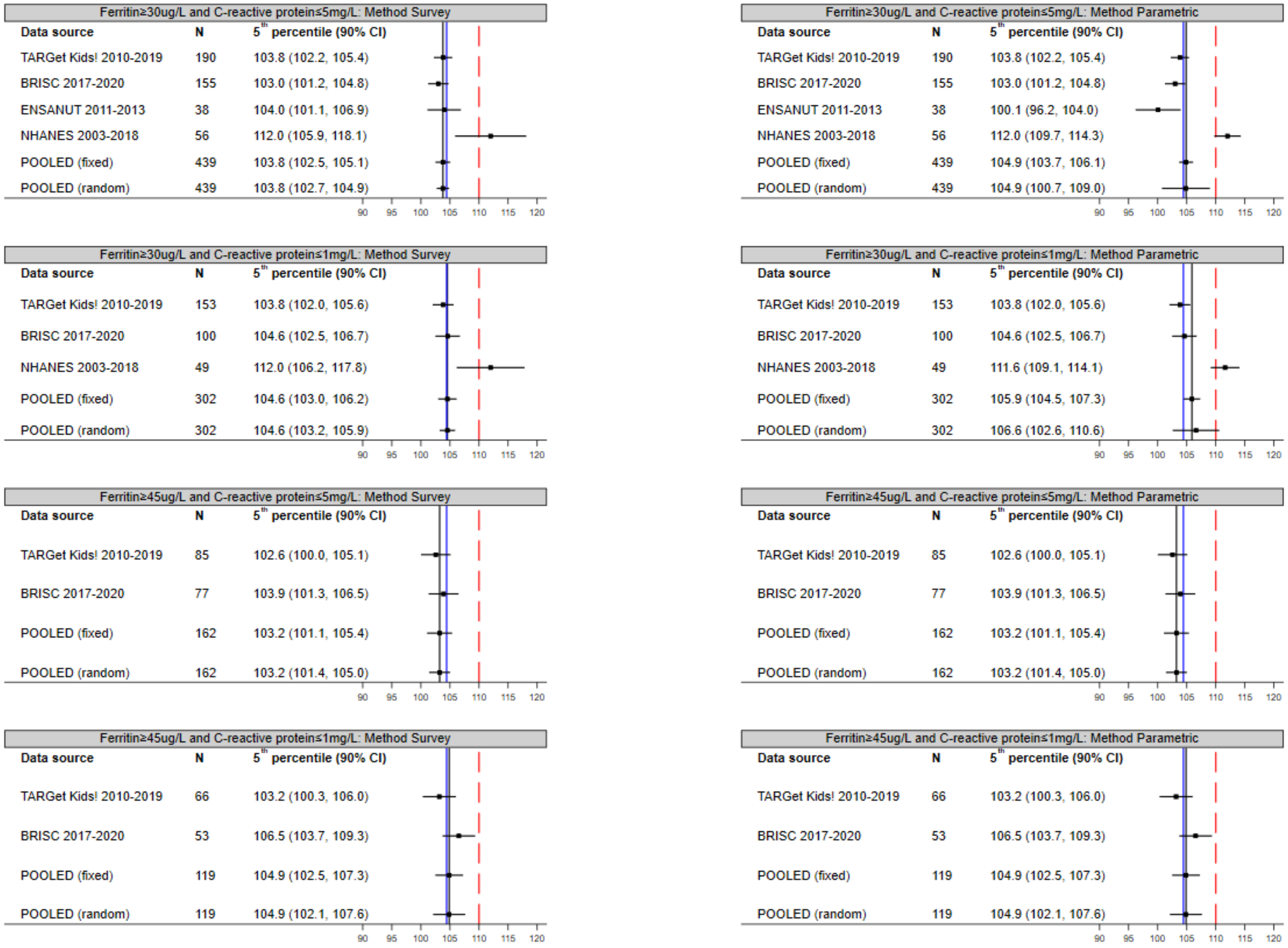

Values are haemoglobin concentration (g/L). CI = Confidence Interval. Red line = cut-off anaemia according to WHO guideline (2011). Blue line = pooled estimate of fixed-effect meta-analysis of primary definition and analysis method. Black line = pooled estimate of fixed-effect meta-analysis. If N<20, no centile is derived.

Figure 5.8: Sensitivity Analyses Children (6-23 months): 5th centile (90% CI) (Part 3).

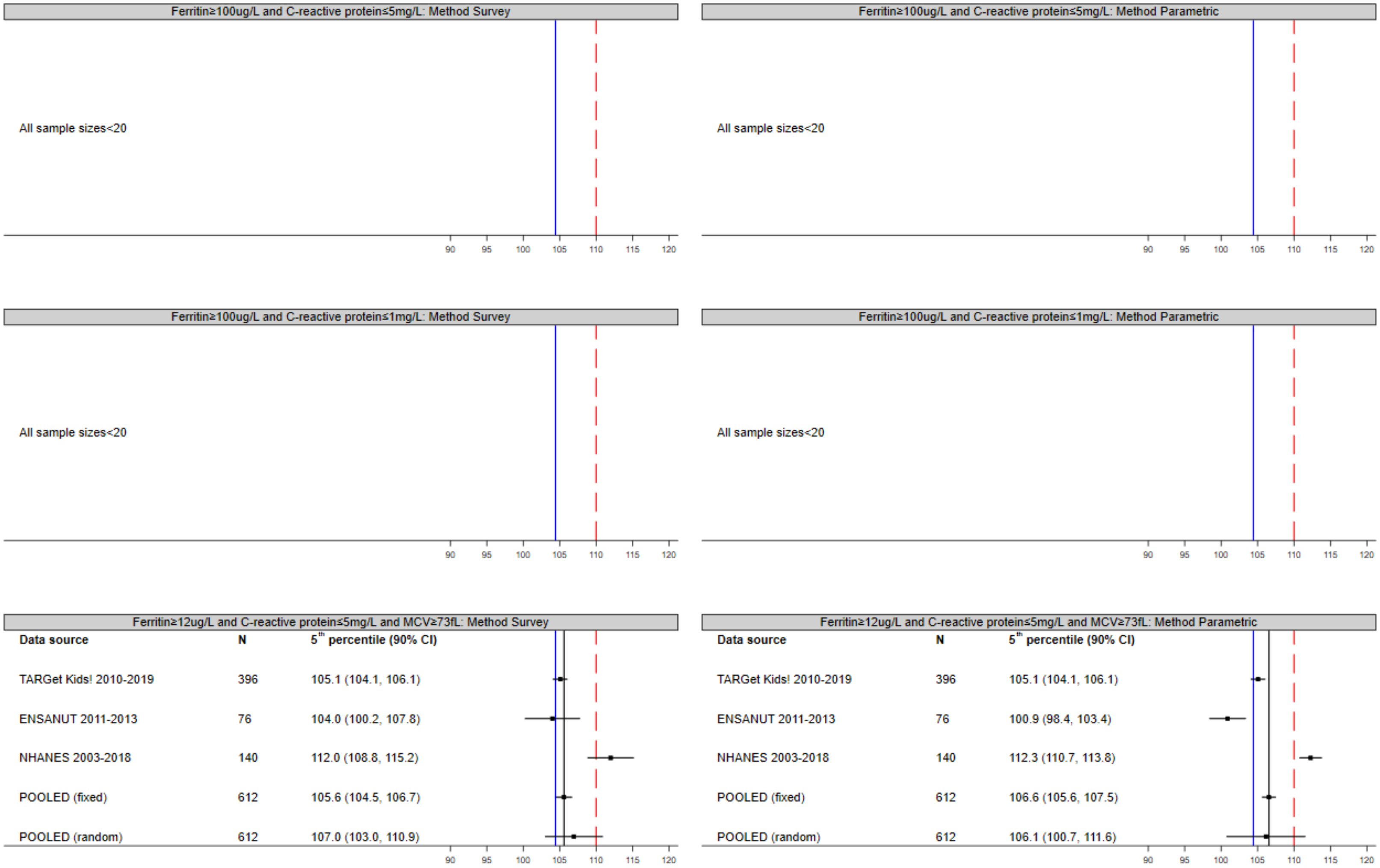

Values are haemoglobin concentration (g/L). CI = Confidence Interval. Red line = cut-off anaemia according to WHO guideline (2011). Blue line = pooled estimate of fixed-effect meta-analysis of primary definition and analysis method. Black line = pooled estimate of fixed-effect meta-analysis. If N<20, no centile is derived. No MCV collected as part of BRISC.

Figure 5.9: Sensitivity Analyses Children (24-59 months): 5th centile (90% CI) (Part 1).

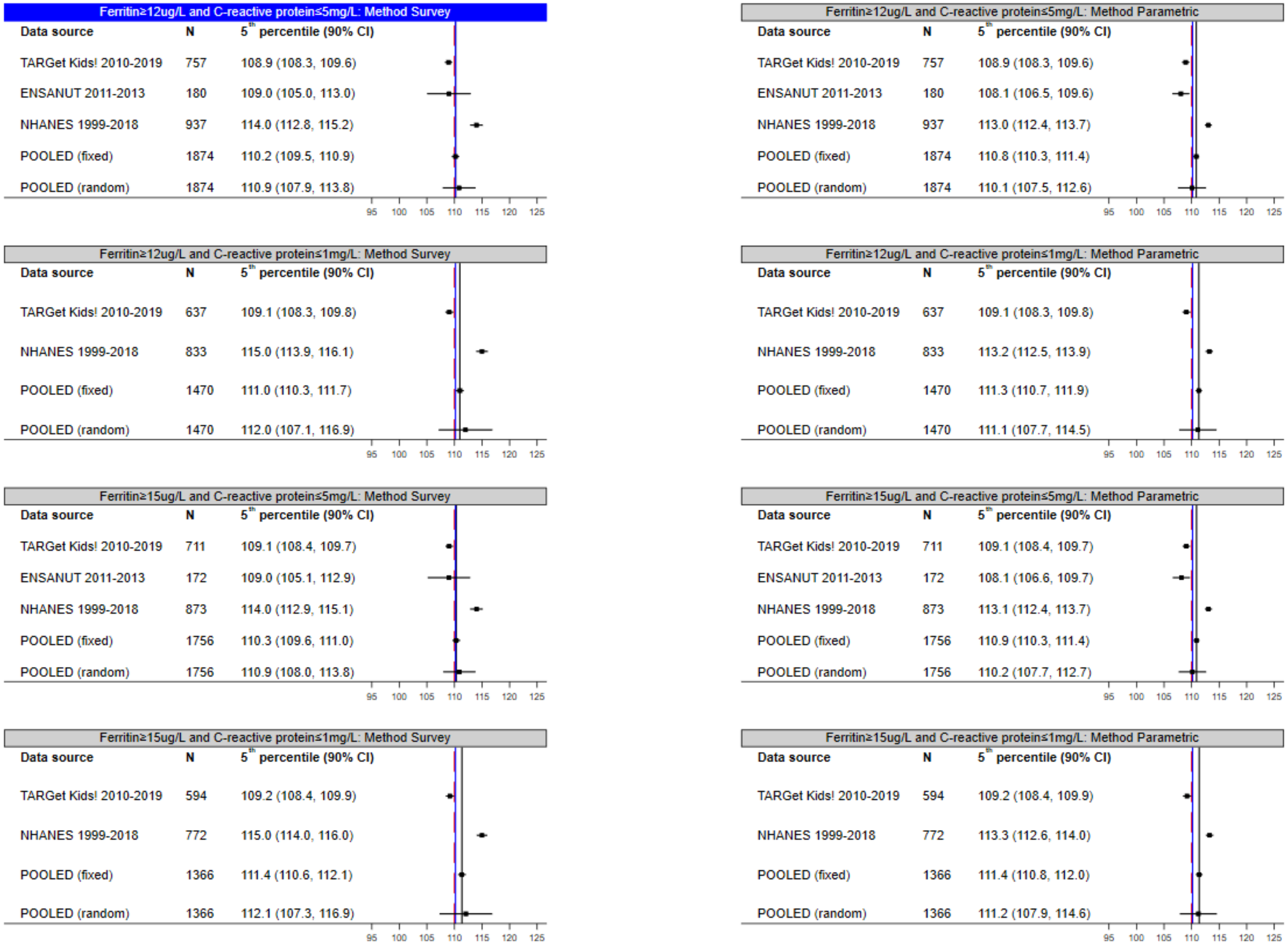

Values are haemoglobin concentration (g/L). CI = Confidence Interval. Red line = cut-off anaemia according to WHO guideline (2011). Blue line = pooled estimate of fixed-effect meta-analysis of primary definition and analysis method (top left figure). Black line = pooled estimate of fixed-effect meta-analysis. If N<20, no centile is derived.

Figure 5.10: Sensitivity Analyses Children (24-59 months): 5th centile (90% CI) (Part 2).

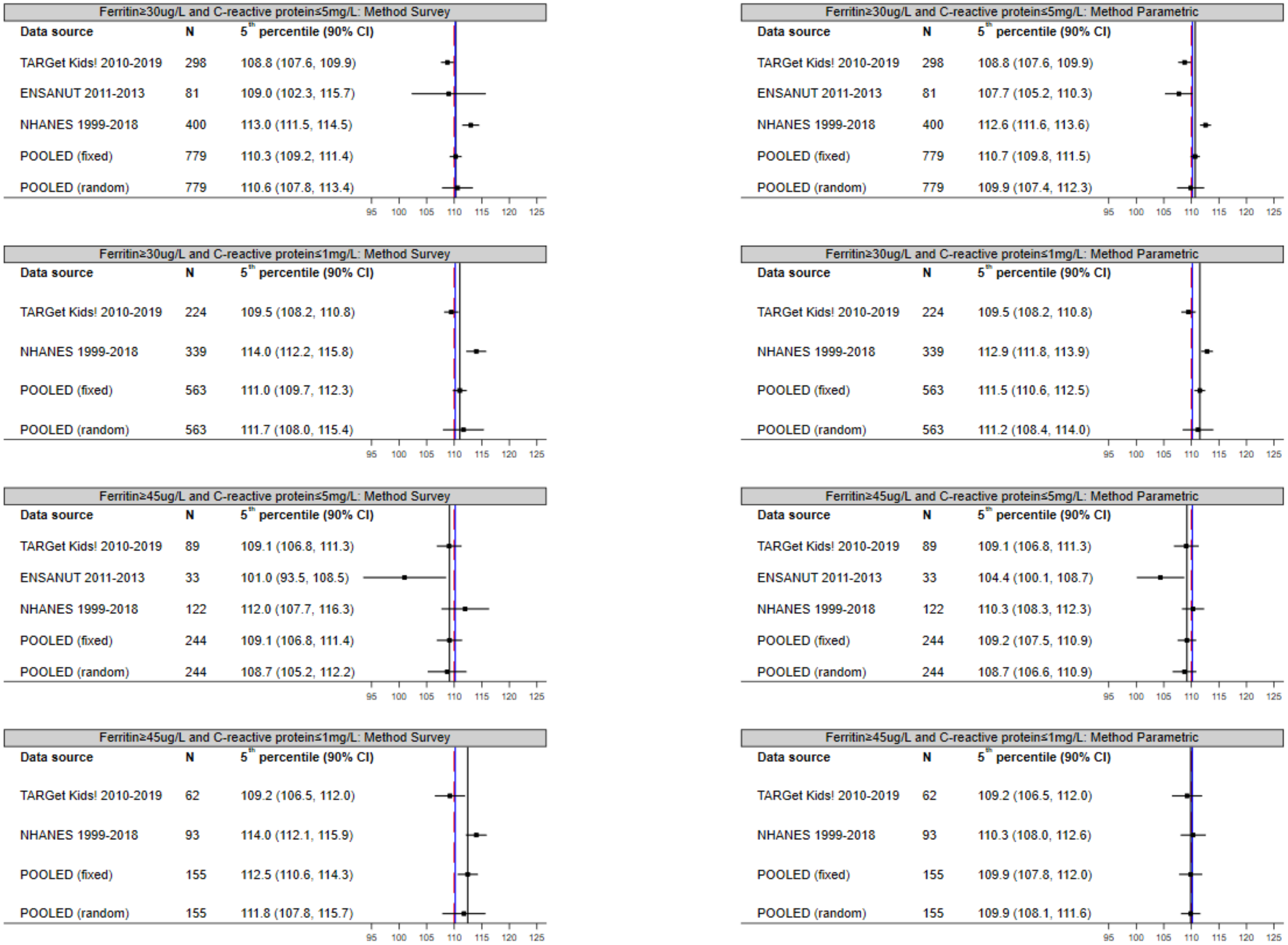

Values are haemoglobin concentration (g/L). CI = Confidence Interval. Red line = cut-off anaemia according to WHO guideline (2011). Blue line = pooled estimate of fixed-effect meta-analysis of primary definition and analysis method. Black line = pooled estimate of fixed-effect meta-analysis. If N<20, no centile is derived.

Figure 5.11: Sensitivity Analyses Children (24-59 months): 5th centile (90% CI) (Part 3).

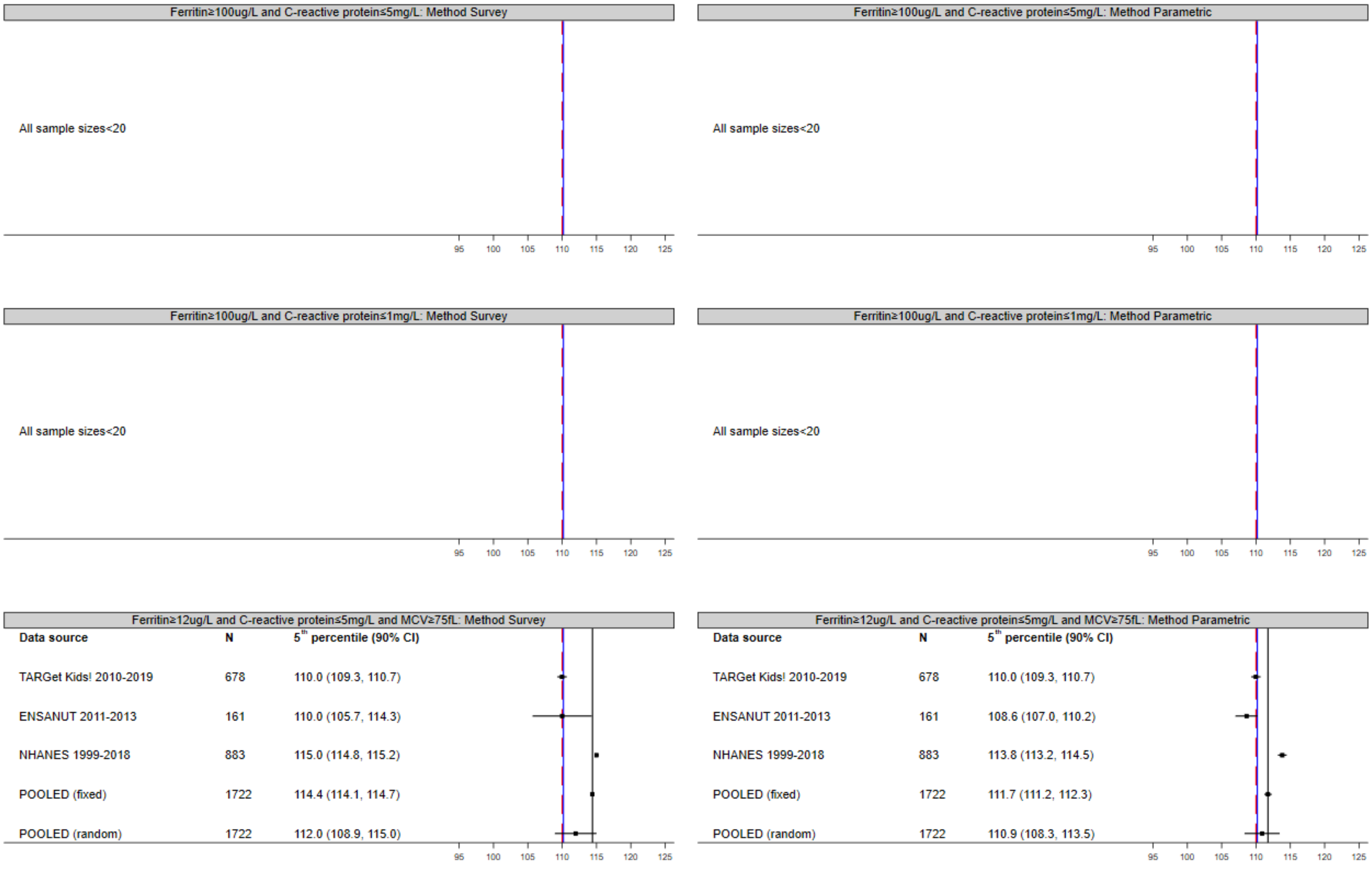

Values are haemoglobin concentration (g/L). CI = Confidence Interval. Red line = cut-off anaemia according to WHO guideline (2011). Blue line = pooled estimate of fixed-effect meta-analysis of primary definition and analysis method. Black line = pooled estimate of fixed-effect meta-analysis. If N<20, no centile is derived.

Figure 5.12: Sensitivity Analyses Children (5-11 years): 5th centile (90% CI) (Part 1).

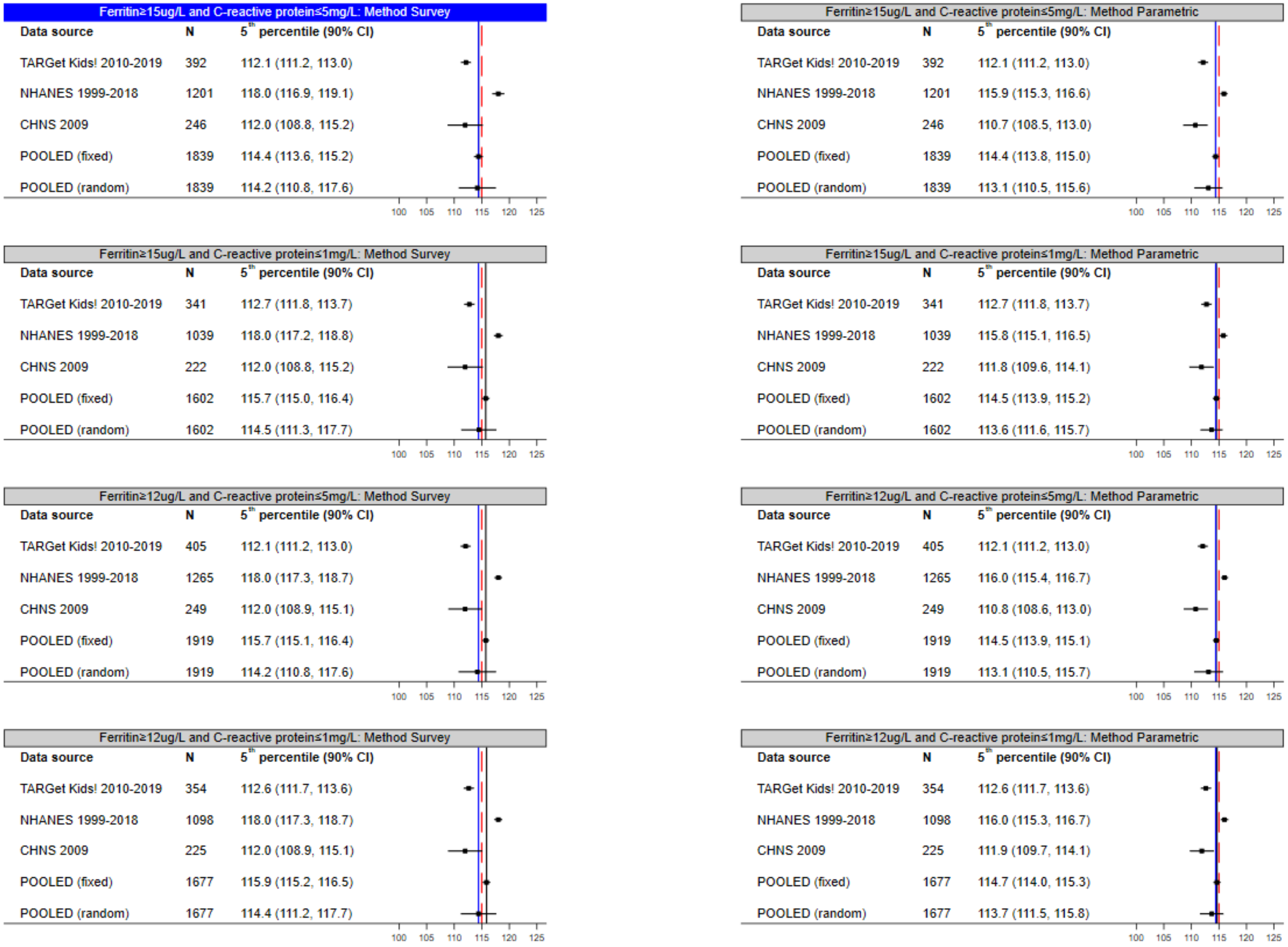

Values are haemoglobin concentration (g/L). CI = Confidence Interval. Red line = cut-off anaemia according to WHO guideline (2011). Blue line = pooled estimate of fixed-effect meta-analysis of primary definition and analysis method (top left figure). Black line = pooled estimate of fixed-effect meta-analysis. If N<20, no centile is derived.

Figure 5.13: Sensitivity Analyses Children (5-11 years): 5th centile (90% CI) (Part 2).

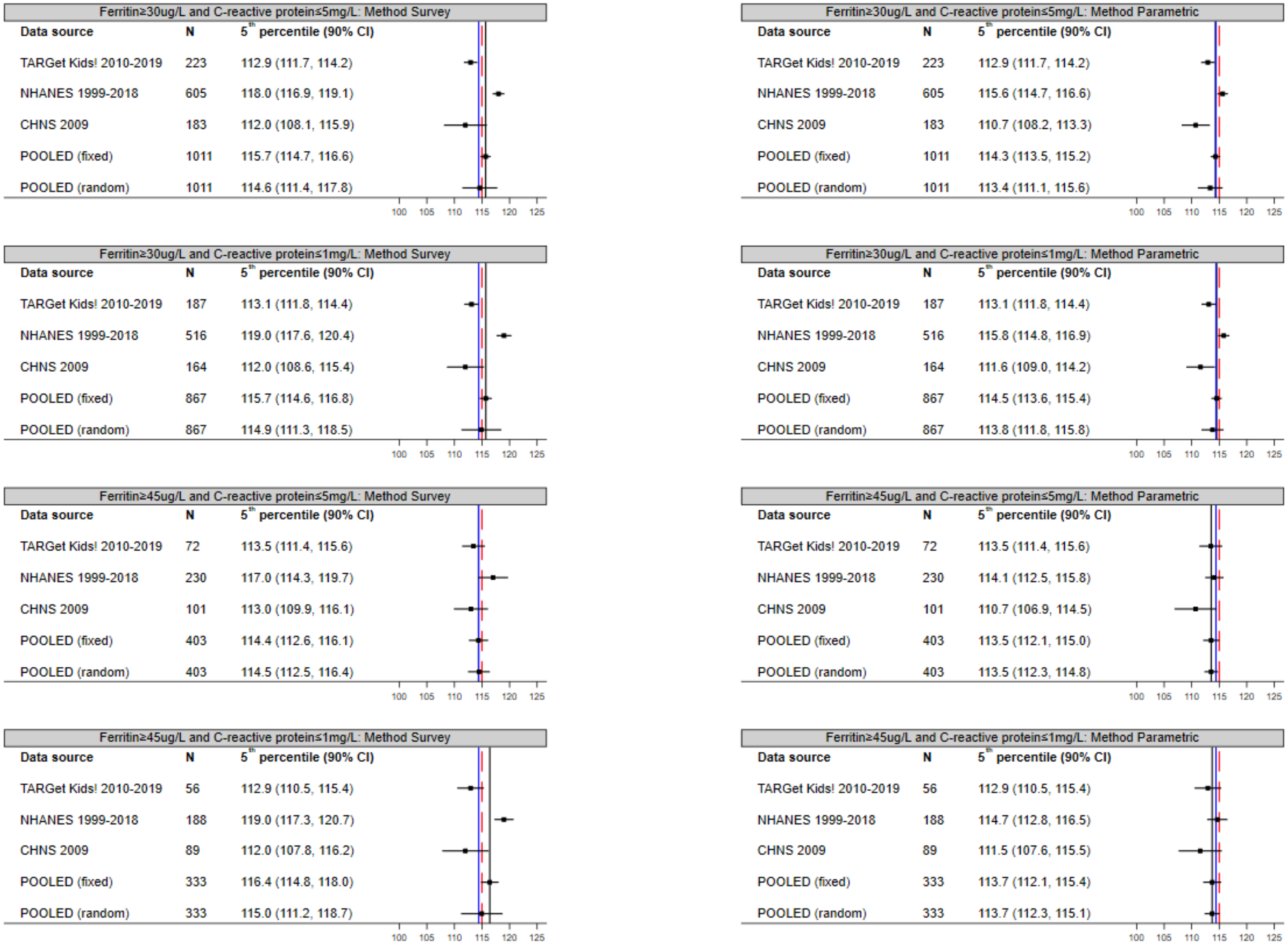

Values are haemoglobin concentration (g/L). CI = Confidence Interval. Red line = cut-off anaemia according to WHO guideline (2011). Blue line = pooled estimate of fixed-effect meta-analysis of primary definition and analysis method. Black line = pooled estimate of fixed-effect meta-analysis. If N<20, no centile is derived.

Figure 5.14: Sensitivity Analyses Children (5-11 years): 5th centile (90% CI) (Part 3).

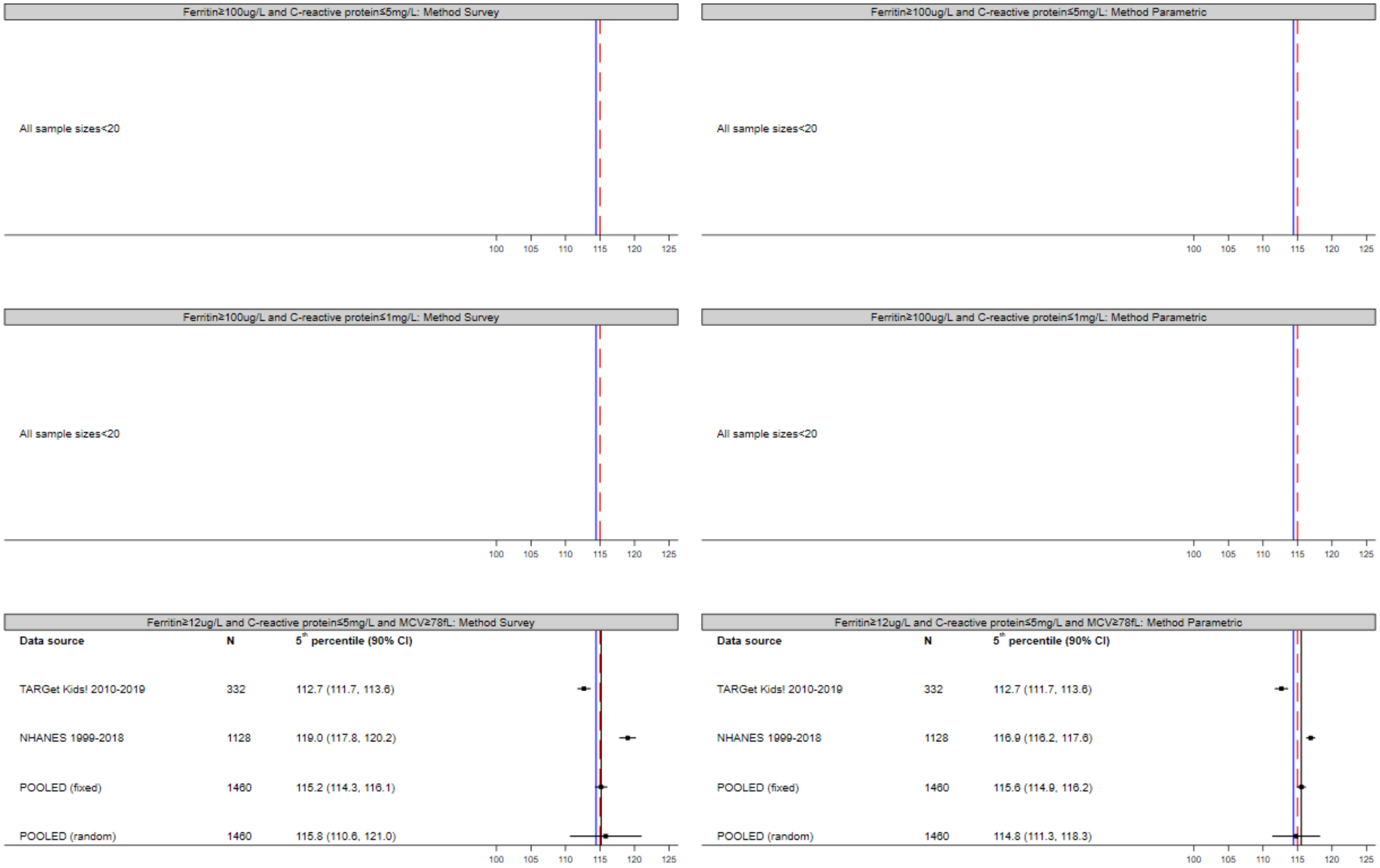

Values are haemoglobin concentration (g/L). CI = Confidence Interval. Red line = cut-off anaemia according to WHO guideline (2011). Blue line = pooled estimate of fixed-effect meta-analysis of primary definition and analysis method. Black line = pooled estimate of fixed-effect meta-analysis. If N<20, no centile is derived. No MCV collected as part of CHNS.

Figure 5.15: Sensitivity Analyses Adolescents (Males) (12-17 years): 5th centile (90% CI) (Part 1).

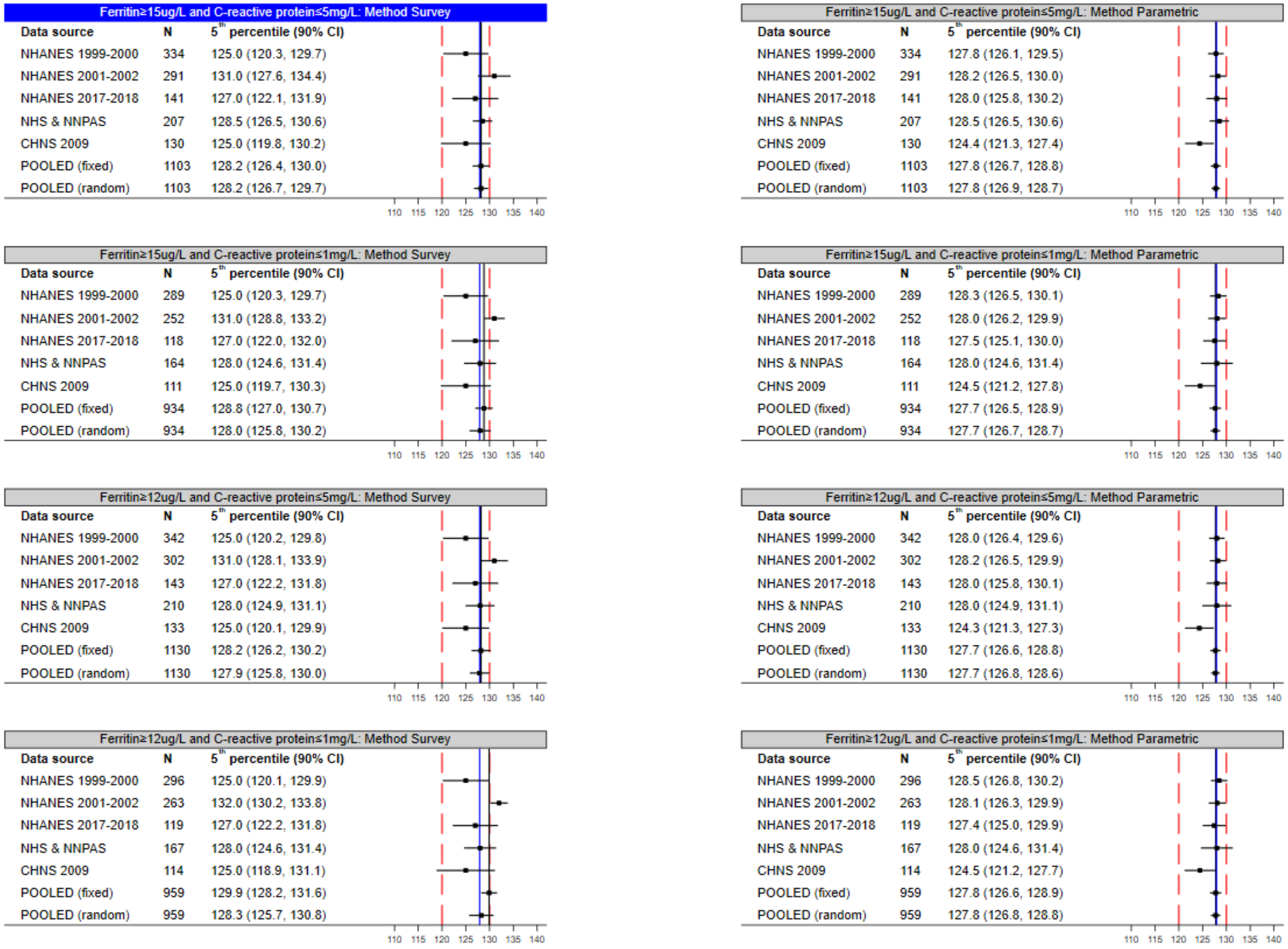

Values are haemoglobin concentration (g/L). CI = Confidence Interval. Red line = cut-off anaemia according to WHO guideline (2011). For males, the cut-off depends on age. Blue line = pooled estimate of fixed-effect meta-analysis of primary definition and analysis method (top left figure). Black line = pooled estimate of fixed-effect meta-analysis. If N<20, no centile is derived. NHS & NNPAS centile not displayed if sample size less than 100, as per Australian Bureau of Statistics requirements for output clearance.

Figure 5.16: Sensitivity Analyses Adolescents (Males) (12-17 years): 5th centile (90% CI) (Part 2).

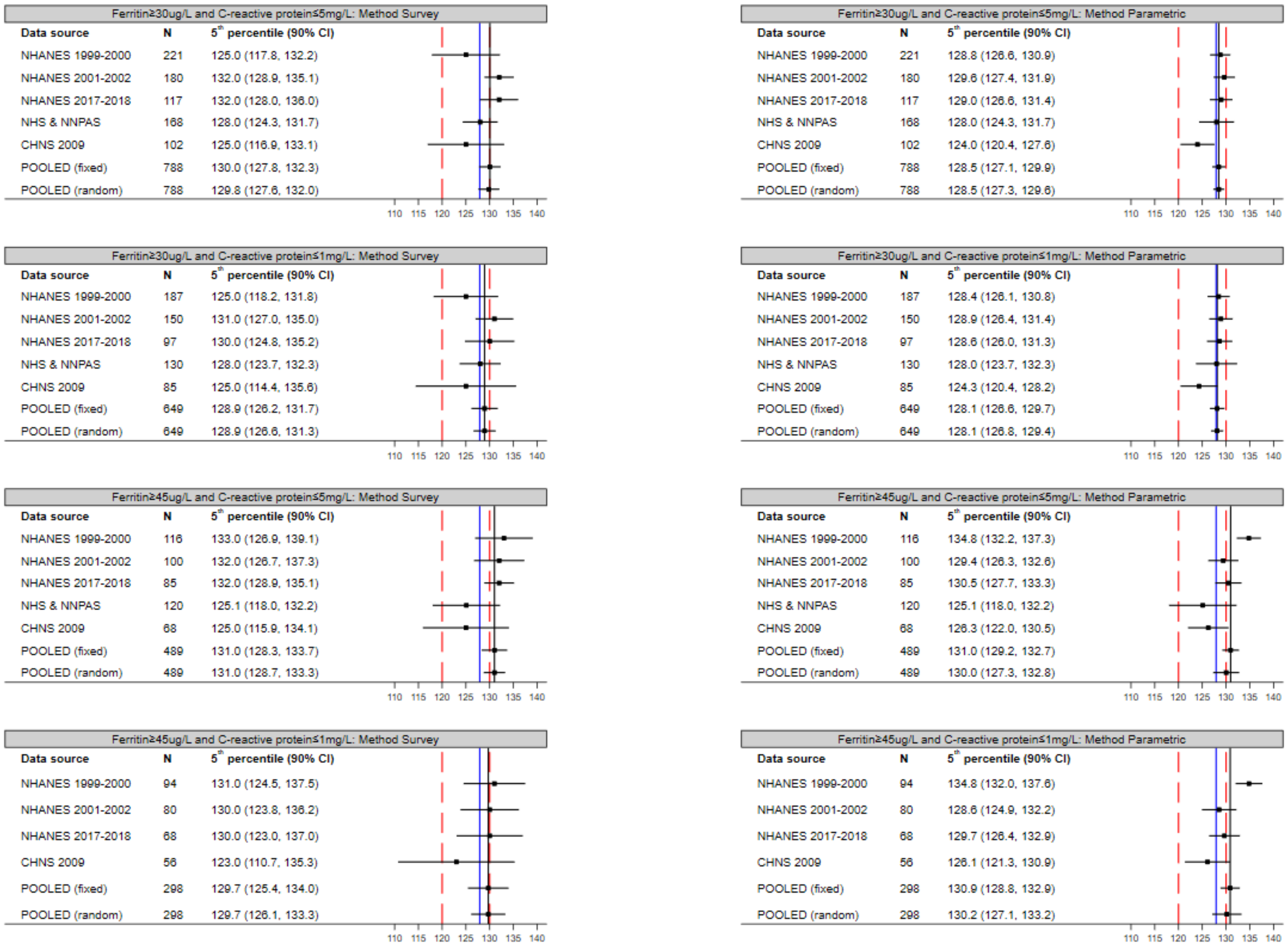

Values are haemoglobin concentration (g/L). CI = Confidence Interval. Red line = cut-off anaemia according to WHO guideline (2011). For males, the cut-off depends on age. Blue line = pooled estimate of fixed-effect meta-analysis of primary definition and analysis method. Black line = pooled estimate of fixed-effect meta-analysis. If N<20, no centile is derived. NHS & NNPAS centile not displayed if sample size less than 100, as per Australian Bureau of Statistics requirements for output clearance.

Figure 5.17: Sensitivity Analyses Adolescents (Males) (12-17 years): 5th centile (90% CI) (Part 3).

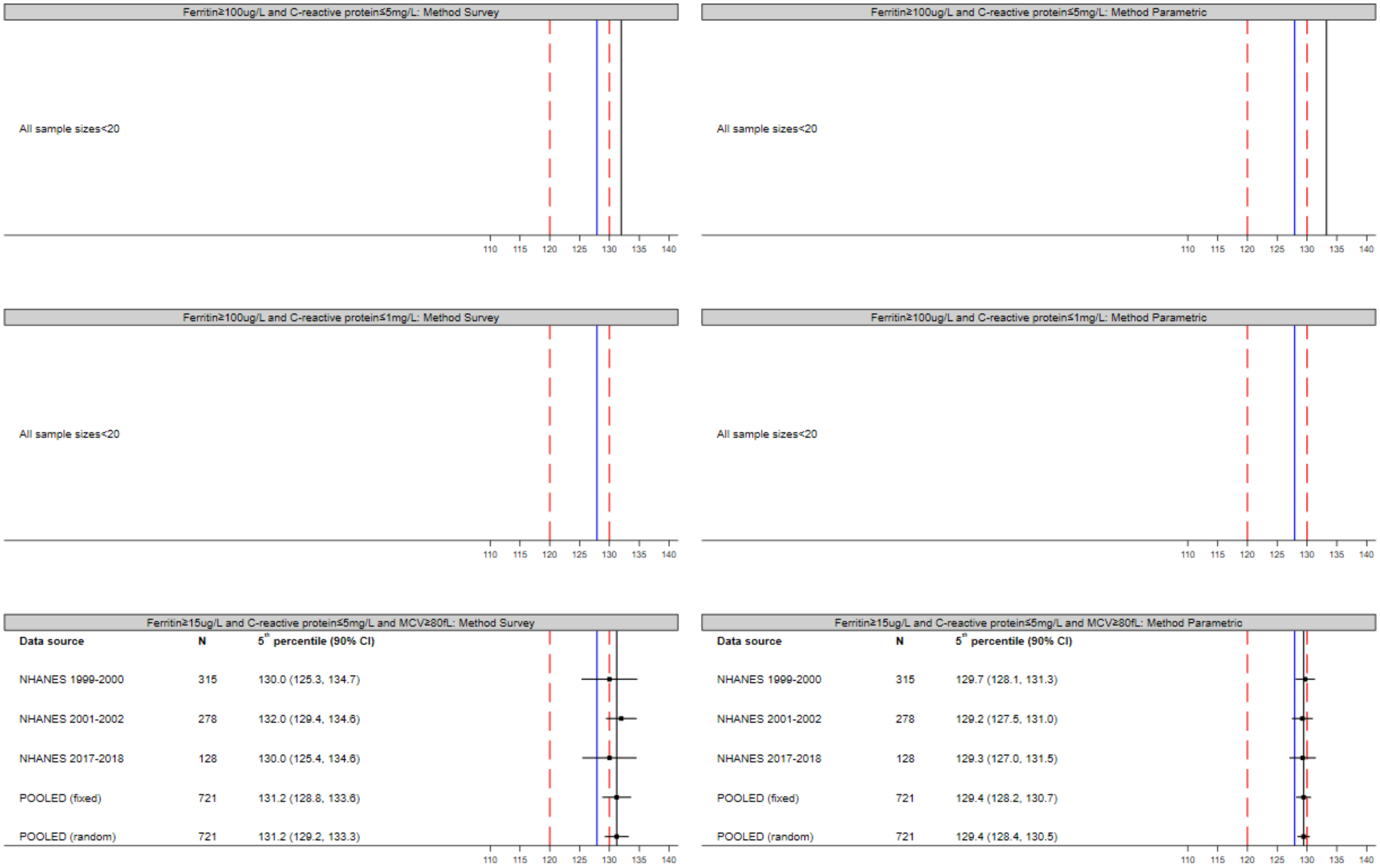

Values are haemoglobin concentration (g/L). CI = Confidence Interval. Red line = cut-off anaemia according to WHO guideline (2011). For males, the cut-off depends on age. Blue line = pooled estimate of fixed-effect meta-analysis of primary definition and analysis method. Black line = pooled estimate of fixed-effect meta-analysis. If N<20, no centile is derived. NHS & NNPAS centile not displayed if sample size less than 100, as per Australian Bureau of Statistics requirements for output clearance. No MCV collected as part of NHS, NNPAS, and CHNS.

Figure 5.18: Sensitivity Analyses Adolescents (Females) (12-17 years): 5th centile (90% CI) (Part 1).

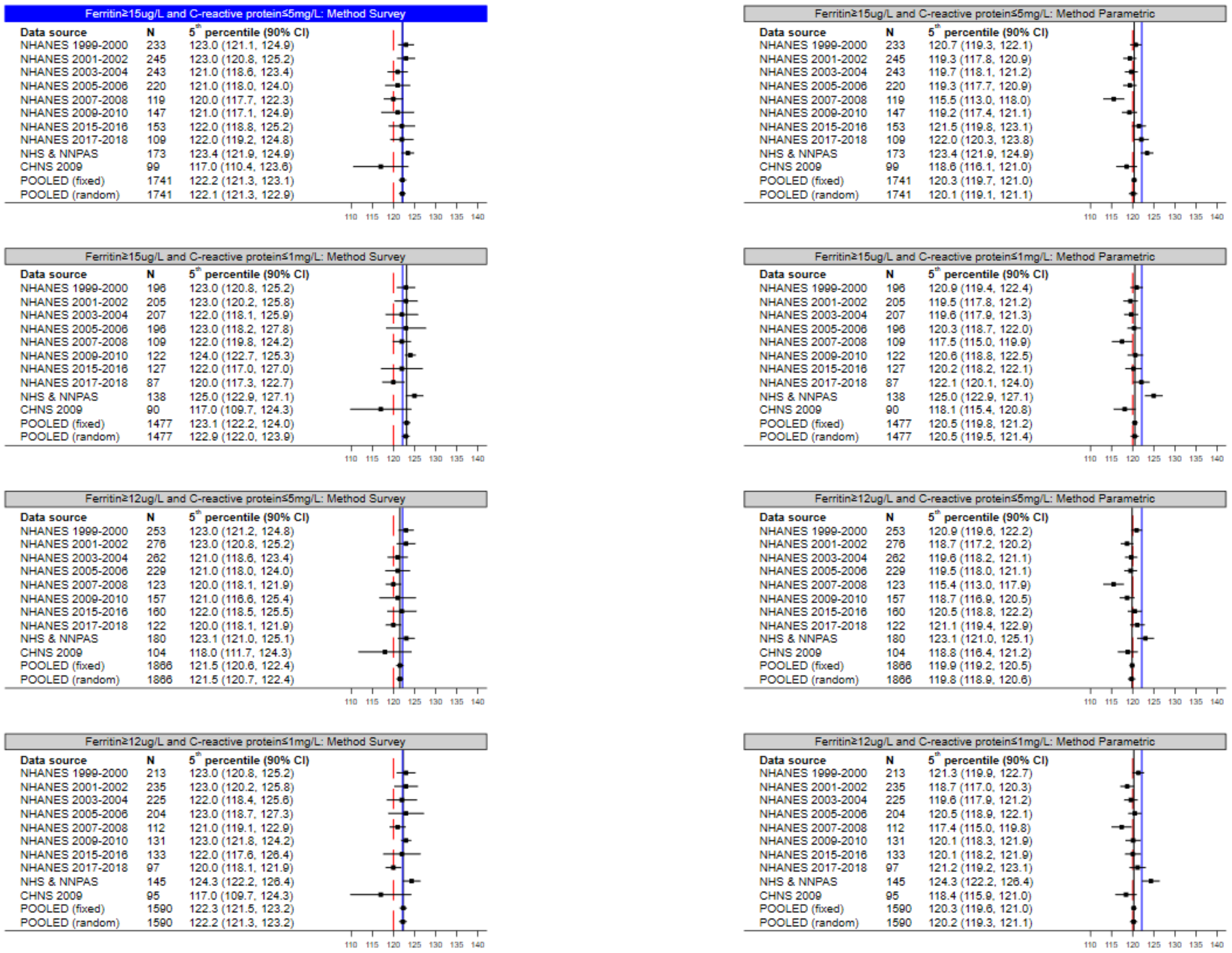

Values are haemoglobin concentration (g/L). CI = Confidence Interval. Red line = cut-off anaemia according to WHO guideline (2011). Blue line = pooled estimate of fixed-effect meta-analysis of primary definition and analysis method (top left figure). Black line = pooled estimate of fixed-effect meta-analysis. If N<20, no centile is derived. NHS & NNPAS centile not displayed if sample size less than 100, as per Australian Bureau of Statistics requirements for output clearance.

Figure 5.19: Sensitivity Analyses Adolescents (Females) (12-17 years): 5th centile (90% CI) (Part 2).

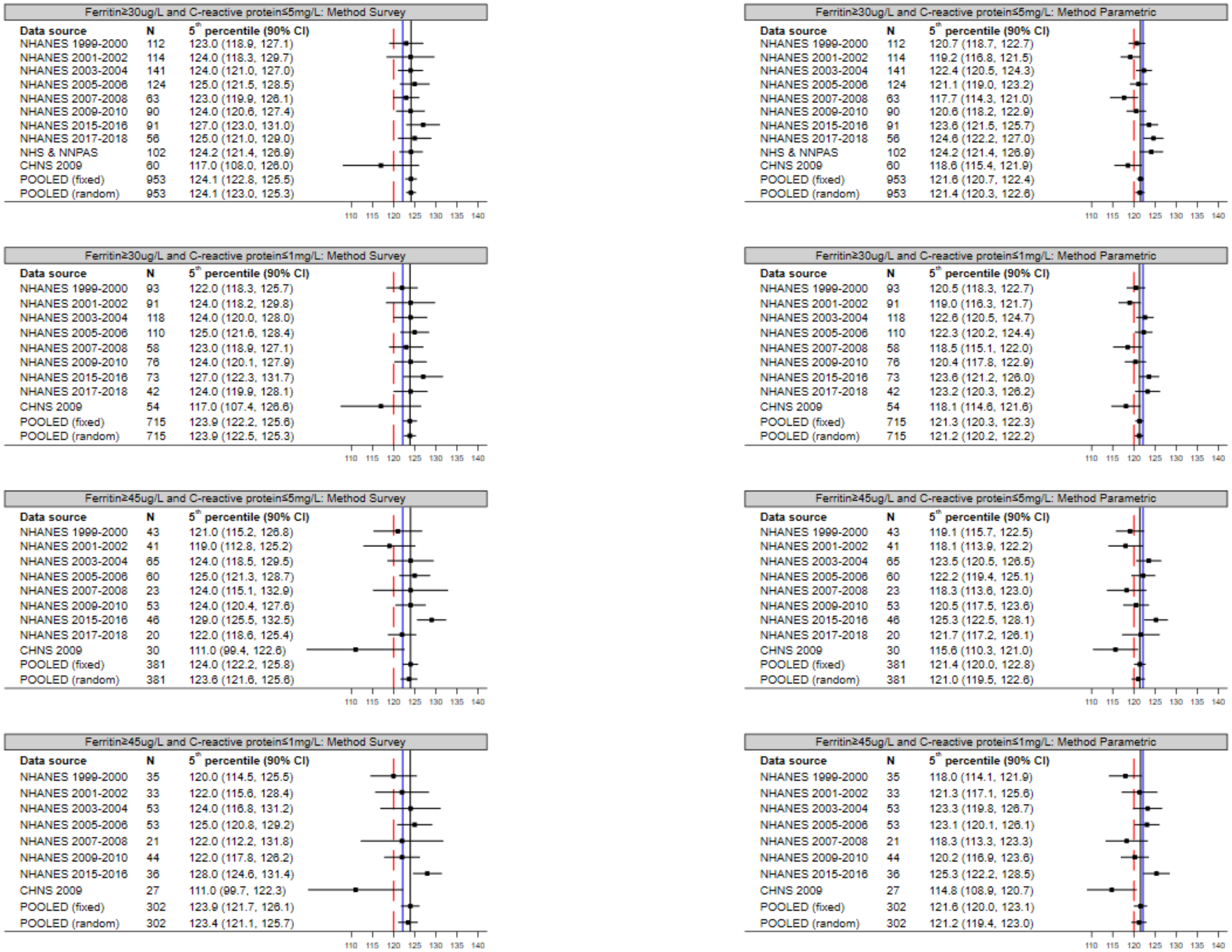

Values are haemoglobin concentration (g/L). CI = Confidence Interval. Red line = cut-off anaemia according to WHO guideline (2011). Blue line = pooled estimate of fixed-effect meta-analysis of primary definition and analysis method. Black line = pooled estimate of fixed-effect meta-analysis. If N<20, no centile is derived. NHS & NNPAS centile not displayed if sample size less than 100, as per Australian Bureau of Statistics requirements for output clearance.

Figure 5.20: Sensitivity Analyses Adolescents (Females) (12-17 years): 5th centile (90% CI) (Part 3).

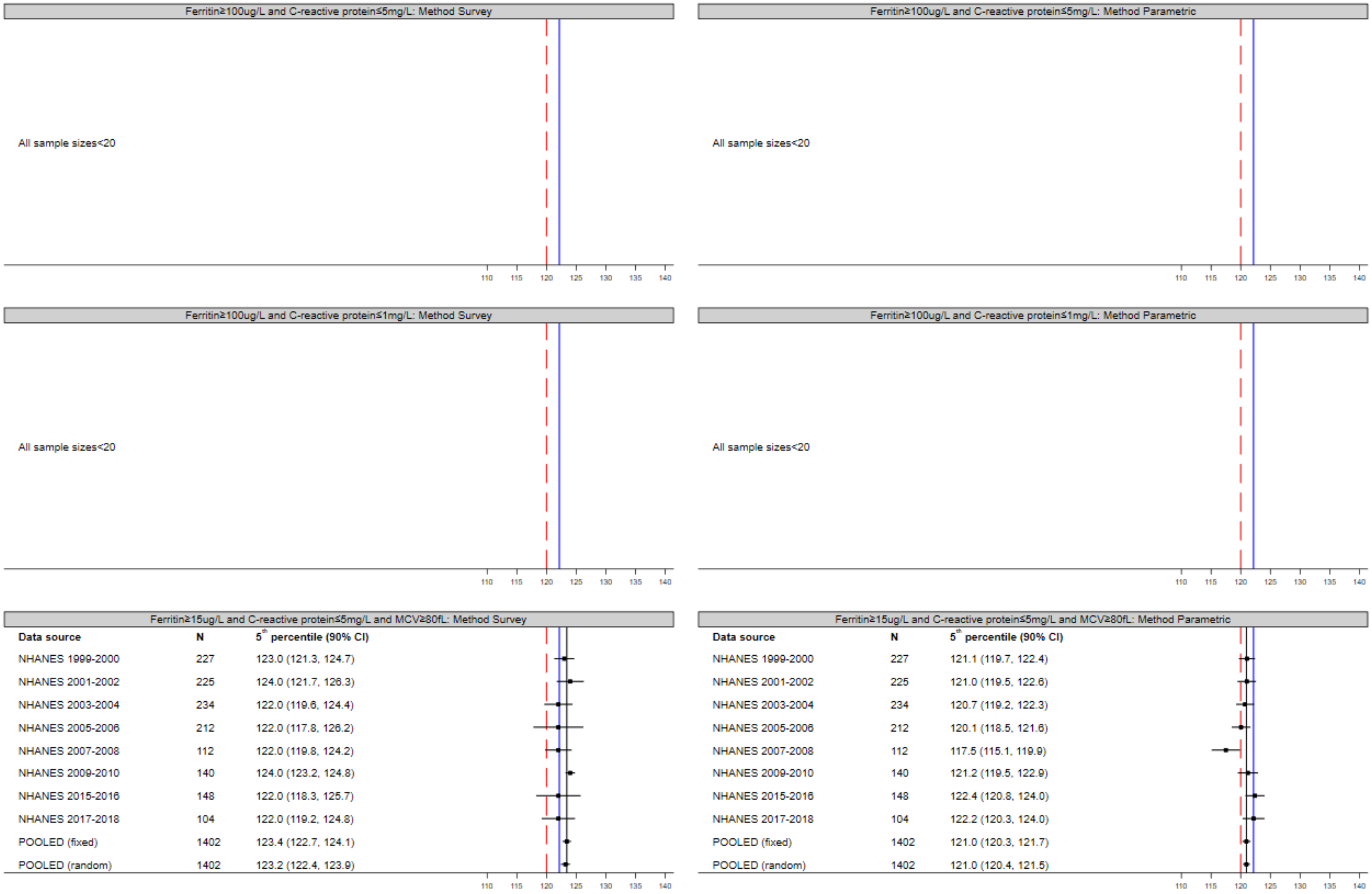

Values are haemoglobin concentration (g/L). CI = Confidence Interval. Red line = cut-off anaemia according to WHO guideline (2011). Blue line = pooled estimate of fixed-effect meta-analysis of primary definition and analysis method. Black line = pooled estimate of fixed-effect meta-analysis. If N<20, no centile is derived. NHS & NNPAS centile not displayed if sample size less than 100, as per Australian Bureau of Statistics requirements for output clearance. No MCV collected as part of NHS, NNPAS, and CHNS.

Figure 5.21: Sensitivity Analyses Pregnant Women (18-45 years): 5th centile (90% CI).

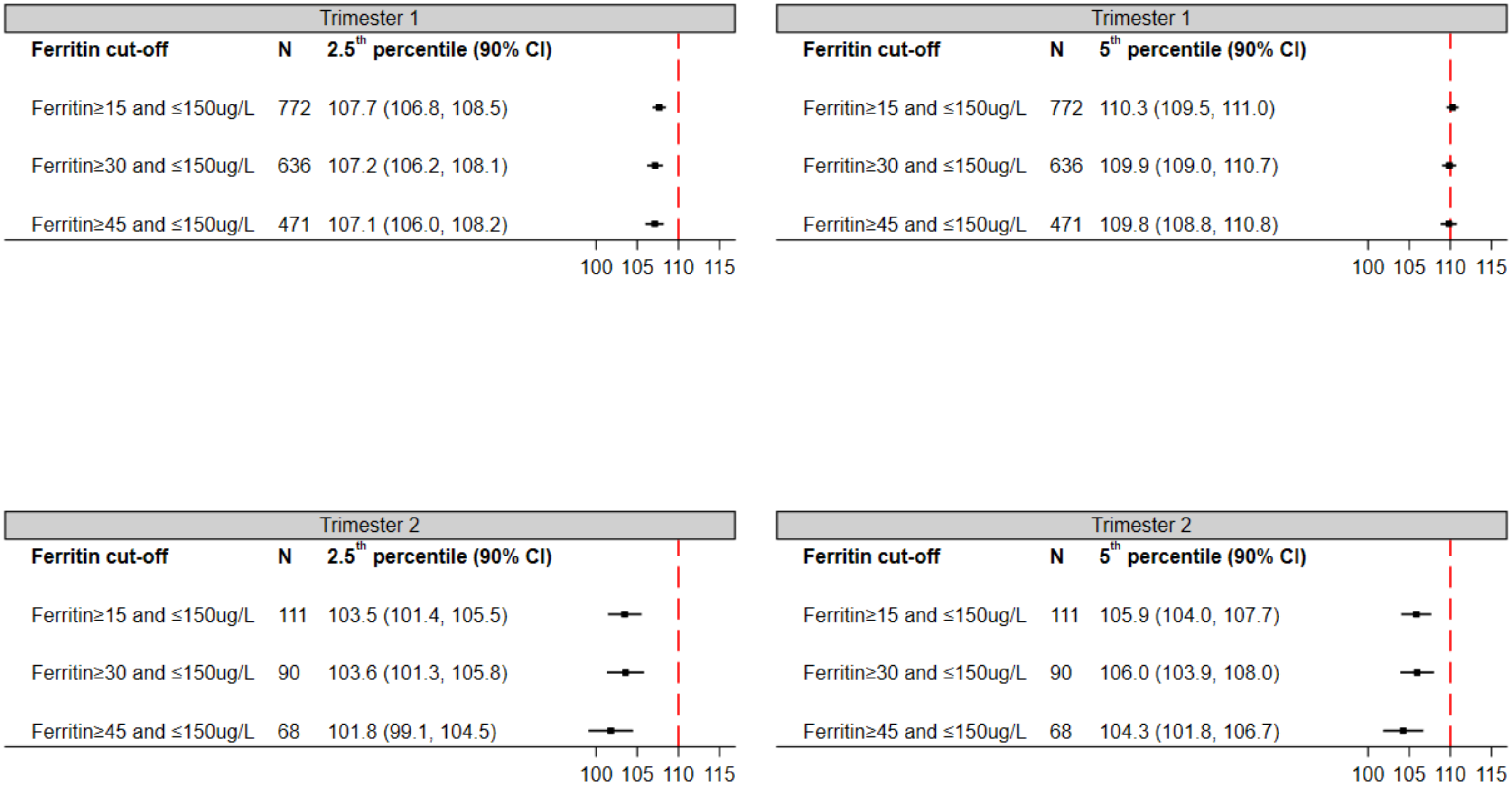

Values are haemoglobin concentration (g/L). CI = Confidence Interval. Red line = cut-off anaemia according to WHO guideline (2011). All include only women with C-reactive protein≤5mg/L. Ferritin and C-reactive protein as collected in the first trimester of pregnancy.

6. Continuous Thresholds

Figure 6.1. Continuous 5th centile thresholds across the life course (6 months to 65 years).

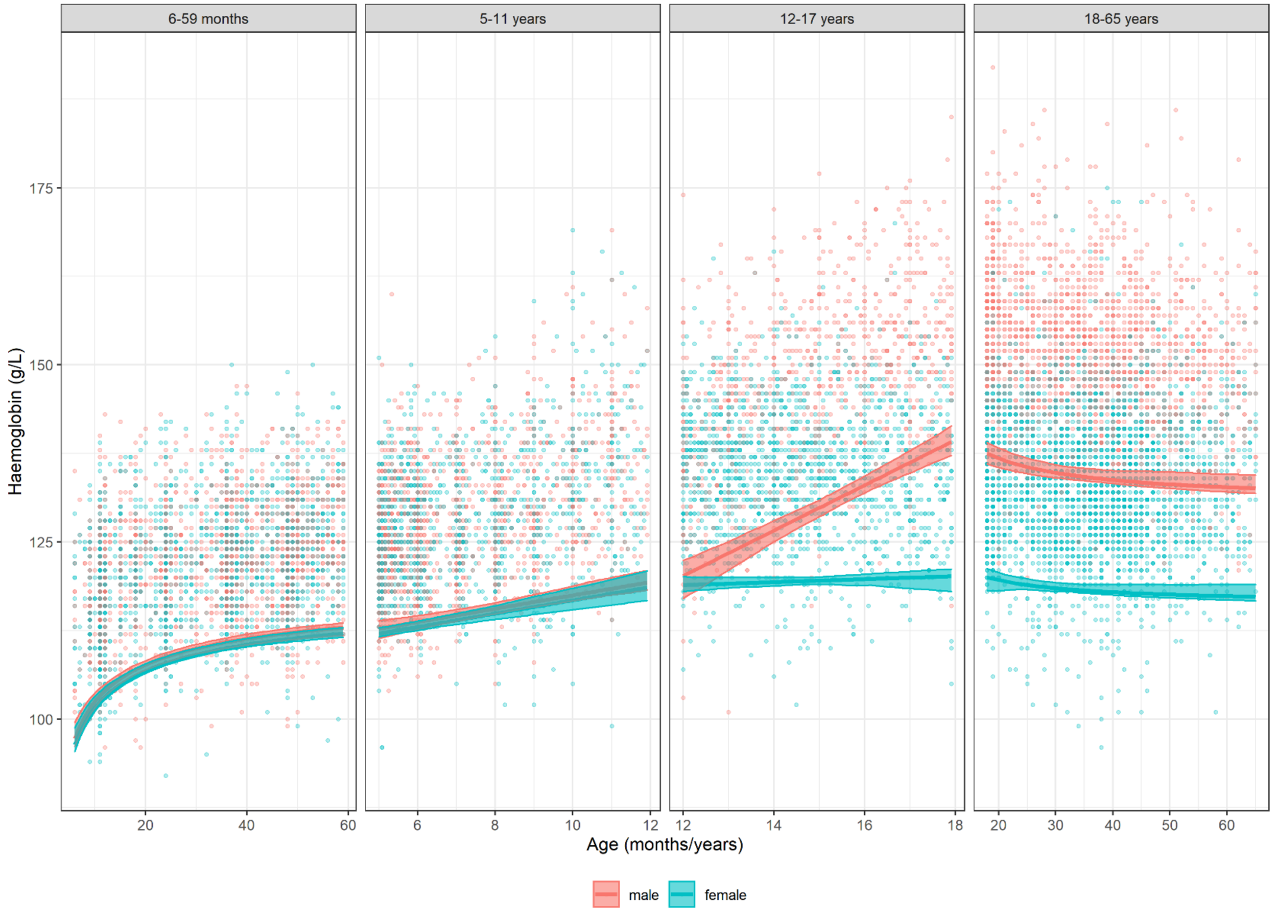

Summary of continuous 5th centile in males (red) and females (blue) across the life course (6 months to 65 years). The pooled continuous centiles and confidence intervals across data sources were estimated without accounting for the complex survey design and weighting. For 6-59 months, data sources consisted of TARGet Kids!, BRISC, ENSANUT, and NHANES; 5-11 years, TARGet Kids!, NHANES, and CHNS; 12-17 years, NHANES, and CHNS; 18-65 years, NHANES, HSE, and CHNS. AHS individual participant data was not available outside the Australian Bureau of Statistics DataLab environment.

7. Ancestry/Genetics

7.1 Ancestry-Specific Genetic Associations with Variation in Haemoglobin Concentration

We investigated whether common genetic variation may explain variation in haemoglobin concentrations among different ancestry groups using summary statistics from three large-scale, multi-ancestry Genome Wide Association Studies (GWAS) of haemoglobin concentrations. (52-54) We sought to identify whether there are genetic variants with large effects that substantially influence overall variance in haemoglobin concentration in particular ancestries. To perform this, we summarised genetic variants (SNPs and Structural Variations, SVs) affecting haemoglobin concentrations and the distribution of effect sizes across different ancestries.

We found 402, 21, 2, 2, and 3 independent genome-wide significant ($P<5\times10^{-9}$) SNPs in the European (EUR), East Asian (EAS), African (AFR), South Asian (SAS) (52) and SAS (Genes & Health study) (54) groups, respectively (Figure 7.1). The larger sample size of EUR and multi-ancestry analyses provided greater power for analysis in this group. There was an inverse relationship between minor allele frequency (MAF) and effect sizes (Figure 7.2.A), as is commonly seen in complex genetic traits. No ancestries had common SNPs (MAF > 0·05) with large effect size, except for rs13331259 (a *FAM234A* intronic variant) which shows a larger effect size ($P=3{\cdot}32 \times 10^{-41}$, inverse rank normal transformation (IRNT) effect size = -0·2495, approximately -3·10 g/L per allele) and has a MAF of 0·11 in the AFR population. This variant is in linkage disequilibrium ($r^2=0{\cdot}48$ in AFR) with rs76462751, a *HBA1* downstream variant. This variant was very rare in the EUR and multi-ancestry analyses, with MAFs of 0·0001 and 0·0038, respectively.

Across the two analyses of SAS individuals, three SNPs representing independent signals were identified; of these, two were specific to that ancestry and notably did not reach genome-wide significance in the non-SAS GWAS, despite larger sample sizes. These were rs529302116 (an *OR52E3P* and *OR52J1P* intergenic variant on Chr11) and rs563555492 (a *PIEZO1* missense variant) (Figure 7.2.B). rs529302116 is in strong LD ($r^2=0{\cdot}83$) with rs33915217 (a *HBB* intronic variant) in the SAS population. This variant occurs more commonly in populations of South Asian ancestry and is one of the more prominent variants observed in patients with β-thalassemia. (65)

Next, we summarized SVs associations (Figure 7.3). (53) There were two SVs chr16:172001-177200_DEL (*HBA1*, *HBA2*) and chr22:37067818-37067888_DUP (*TMPRSS6*) at genome-wide significance ($P< 5\times10^{-8}$). Of these, chr22:37067818-37067888_DUP (*TMPRSS6*) is in LD ($r^2=0{\cdot}77$) with rs5756504 (a *TMPRSS6* intronic variant), that has previously been identified through GWAS. The second SV, chr16:172001-177200_DEL (*HBA1*, *HBA2*) occurred at a high frequency in the AFR ancestry cohort (MAF=0·176) and is located ~80kb away from the SNP GWAS signal rs13331259 that also showed higher MAF in the AFR ancestry. Conditional analyses found the SNP and SV signals to be independent, suggesting there may be multiple variants in this region influencing haemoglobin concentrations that occur at higher frequencies in African populations, potentially due to positive selection.

Single-variant-based GWAS is typically focused on the identification of common variants (MAF>0·05) but is underpowered for low-frequency and rare variants due to small sample sizes and low minor allele frequencies. However, these less-common variants could explain additional disease risk or trait variability. To address this limitation, gene-based collapsing analysis of multiple variants in a defined genetic region can identify genes in which low-frequency and rare variants are, in aggregate, associated with a phenotype. To evaluate this, we accessed the AstraZeneca PheWAS Portal (55) which contains gene–phenotype associations calculated from exome-sequenced UK Biobank participants of European ancestry. From the gene-based results, we identified 13 genes associated with haemoglobin concentrations, with $P < 2{\cdot}665\times10^{-6}$, of which five (*HBB, KLF1, PIEZO1, SLC4A1, TMPRSS6*) were considered clinically relevant causes of rare anaemia via the Genomics England PanelApp 'Green' gene list (56) (Figure 7.4). Most genes show small effect sizes, except for *HBB* which was strongly associated with lower haemoglobin concentrations ($P = 2{\cdot}31 \times 10^{-92}$, IRNT effect size=-1·8941, approximately -23·51 g/L per allele). Across these five clinically relevant rare anaemia genes, predicted loss-of-function (pLoF) variants occurred at varying frequencies in different populations (Figure 7.5). For example, *HBB* has a higher prevalence in individuals of EAS, SAS, and Middle Eastern ancestries; however, the cumulative pLoF variants frequency is lower than 0·006. Rare pLoF variants in *TMPRSS6* have the highest prevalence in African/African American populations, with a cumulative pLoF variant frequency of around 0·03. The gene-based association with *TMPRSS6* (in Europeans), showed a more modest effect ($P=2{\cdot}41 \times 10^{-7}$, IRNT effect size=-0·1419, approximately -1·76 g/L per allele).

Table 7.1: Genetic association results (summary statistics) of haemoglobin concentrations used.

Variant	Sample size	Number of variants	Ancestry	Reference
SNP	746,667	~ 45 million autosomal variants	76% European<br>20% East Asian<br>2% African<br>1% Hispanic/Latino<br>1% South Asian	Chen et al., 2020 (52)
SNP	35,418	24,092,221 autosomal variants	South Asian	Genes & Health Study (54)
SV	50,675	96,049 structural variants	59% European<br>24% African<br>16% Hispanic/Latino<br>1% Asian	Wheeler et al., 2022 (53)

SNP = Single Nucleotide Polymorphisms, SV = Structural Variants.

Figure 7.1: Multi-ancestry and ancestry-specific haemoglobin-associated genome-wide significant loci.

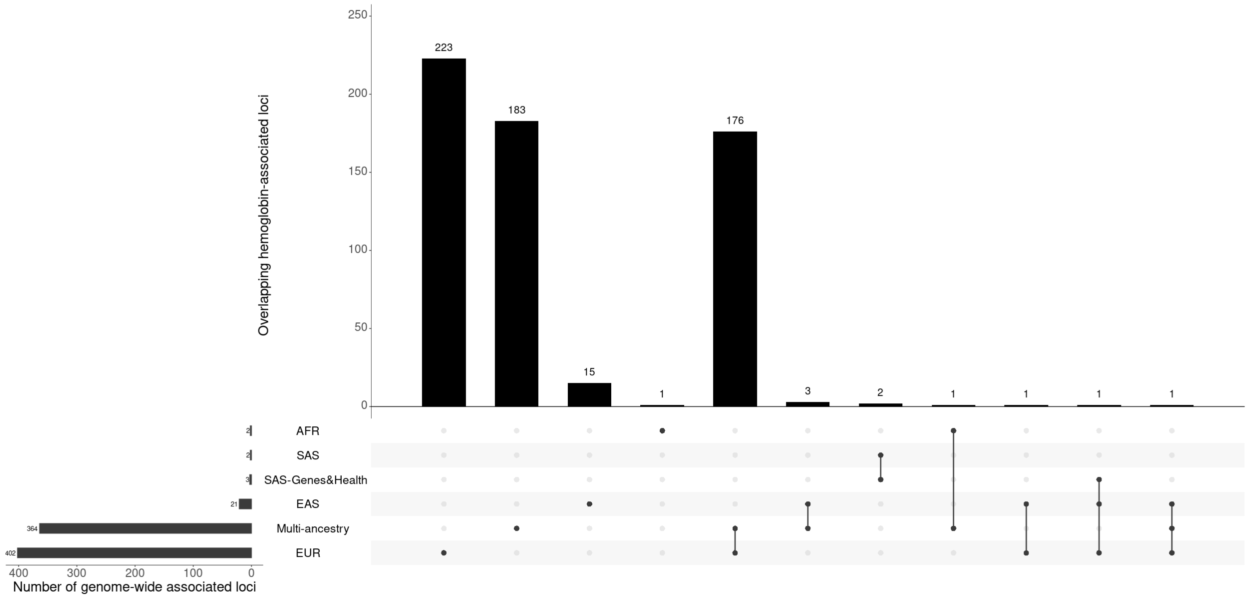

EUR, European; EAS, East Asian; AFR, African; SAS, South Asian; SAS-Genes&Health, South Asian from Genes & Health study.

Figure 7.2: Scatter plot of minor allele frequency (MAF) and standardized effect size for SNPs.

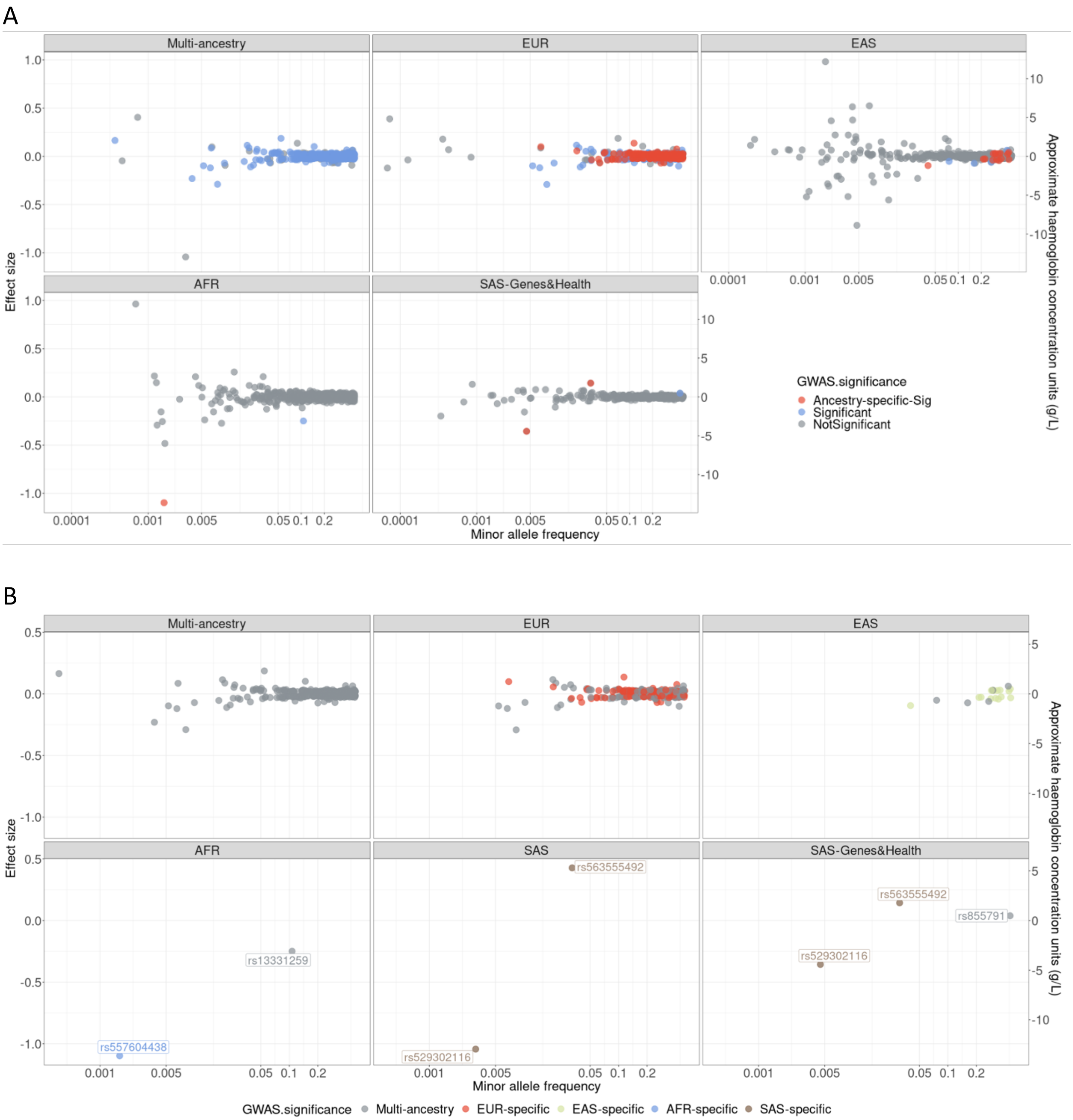

MAF (x-axis on a $\log_{10}$ scale) and standardized effect size (y-axis) of the minor alleles of the 607 variants (both ancestry-specific and cross-ancestry loci) in the GWAS summary statistics. To convert the standardized effect size to an approximate haemoglobin unit (g/L), we regarded standard deviation per minor allele as 12·41 units (g/L), which was estimated from the UKBB population-based cohort. (A) 607 haemoglobin variants with a $P < 5 \times 10^{-9}$ from a single or multi-ancestry analysis. Genome-wide significant variants for the stated ancestry are indicated in red. Variants that were genome-wide significant in the stated ancestry, and in at least one other ancestry are indicated in blue. Variants that did not reach genome-wide significance in the stated ancestry are indicated in grey. (B) Genome-wide significant variants in the GWAS summary statistics for each ancestry. Colored by Ancestry-specific or cross-ancestry variants.

Figure 7.3. Scatter plot of minor allele frequency (MAF) and standardized effect size for SVs.

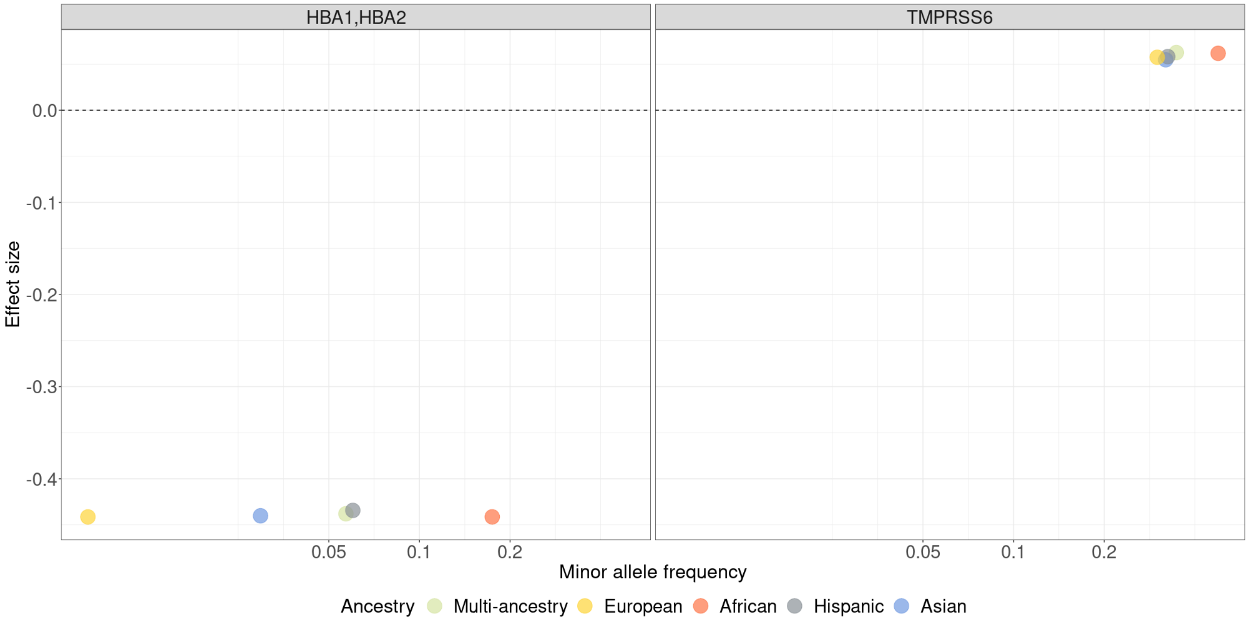

We plotted the MAF (x-axis on a $\log_{10}$ scale) and effect size (y-axis) of the minor alleles of the two structural variants that reached genome-wide significance ($P < 5 \times 10^{-8}$) in the GWAS summary statistics. Colored by ancestry.

Figure 7.4: Scatter plot of frequency and effect size in the UKBB cohort.

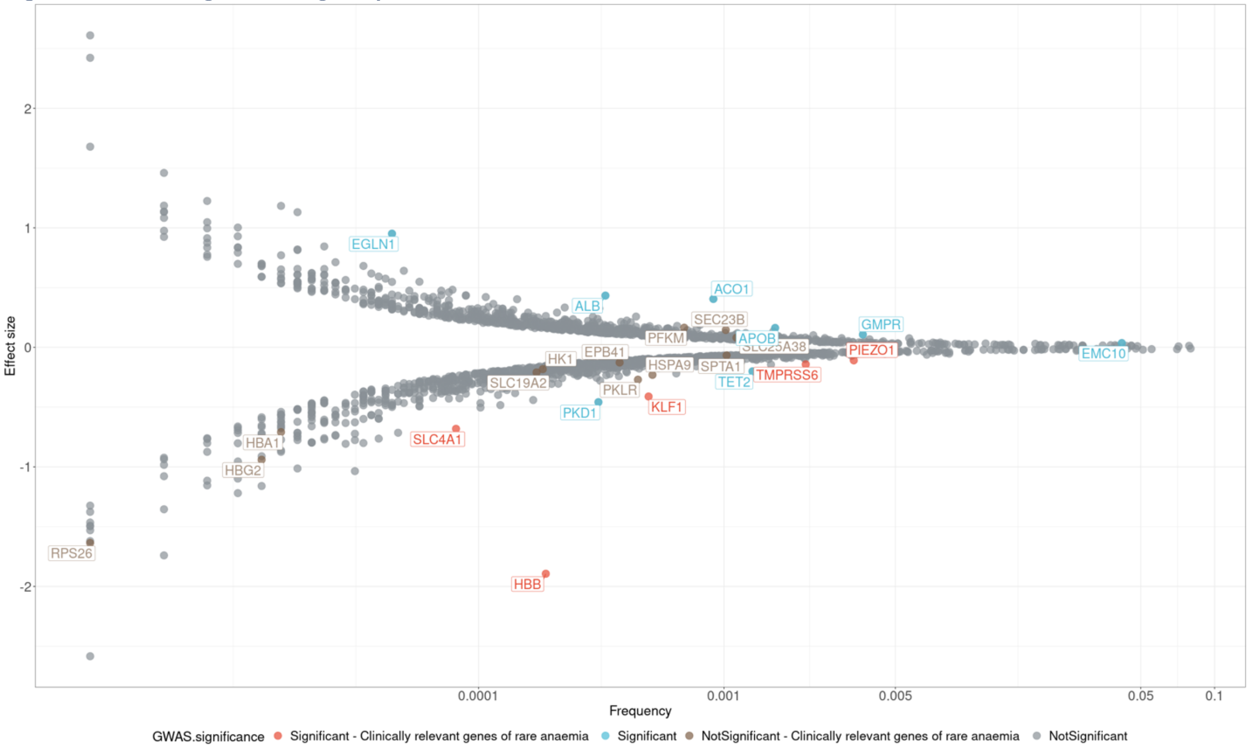

Frequency represents the proportion of individuals with a qualifying variant for gene-level results. The effect size is computed from the gene-based collapsing model. Significant associations are those with $P < 2{\cdot}665 \times 10^{-6}$. The color is represented by whether it is significant and whether it is a clinically relevant cause of rare anaemia from the Genomics England PanelApp 'Green' gene list.

Figure 7.5: Allele frequency plot of predicted loss-of-function (pLOF) variants.

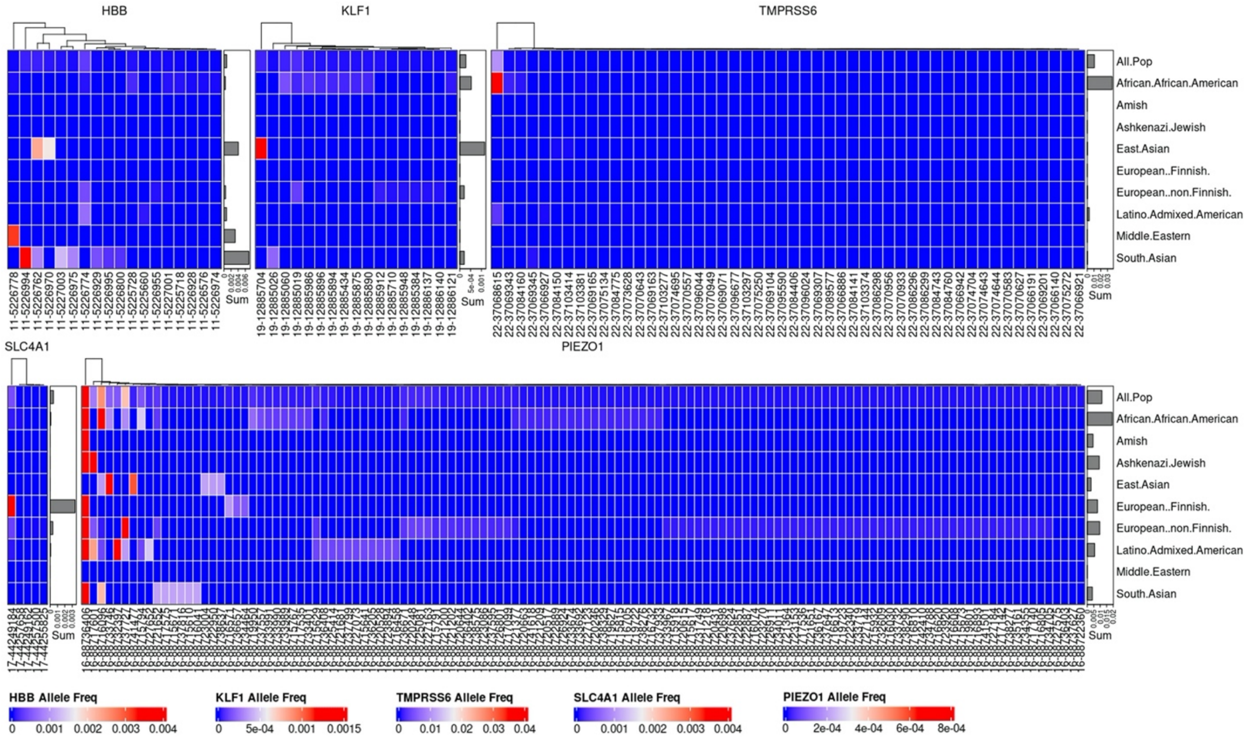

Frequency distribution in different gnomAD populations of five significant genes ($P < 2{\cdot}665 \times 10^{-6}$) that have been identified as clinically relevant causes of rare anaemia from the Genomics England PanelApp 'Green' gene list. The color scale for each gene ranges from blue for the lowest allele frequency to red for the highest allele frequency.

7.2 Genes & Health Research Team

The current members from the Genes & Health Research Team (in alphabetical order by surname) are:

Shaheen Akhtar, Mohammad Anwar, Elena Arciero, Omar Asgar, Samina Ashraf, Saeed Bidi, Gerome Breen, James Broster, Raymond Chung, David Collier, Charles J Curtis, Shabana Chaudhary, Megan Clinch, Grainne Colligan, Panos Deloukas, Ceri Durham, Faiza Durrani, Fabiola Eto, Sarah Finer, Joseph Gafton, Ana Angel, Chris Griffiths, Joanne Harvey, Teng Heng, Sam Hodgson, Qin Qin Huang, Matt Hurles, Karen A Hunt, Shapna Hussain, Kamrul Islam, Vivek Iyer, Benjamin M Jacobs, Ahsan Khan, Claudia Langenberg, Cath Lavery, Sang Hyuck Lee, Daniel MacArthur, Sidra Malik, Daniel Malawsky, Hilary Martin, Dan Mason, Rohini Mathur, Mohammed Bodrul Mazid, John McDermott, Caroline Morton, Bill Newman, Elizabeth Owor, Asma Qureshi, Shwetha Ramachandrappa, Mehru Raza, Jessry Russell, Nishat Safa, Miriam Samuel, Michael Simpson, John Solly, Marie Spreckley. Daniel Stow, Michael Taylor, Richard C Trembath, Karen Tricker, David A van Heel, Klaudia Walter, Caroline Winckley, Suzanne Wood, John Wright, Ishevanhu Zengeya, Julia Zöllner

8. References

1. World Health Organization. WHO guideline on use of ferritin concentrations to assess iron status in individuals and populations. Geneva: World Health Organization. 2020.
2. Inker LA, Eneanya ND, Coresh J, Tighiouart H, Wang D, Sang Y, et al. New Creatinine- and Cystatin C-Based Equations to Estimate GFR without Race. N Engl J Med. 2021;385(19):1737-49.
3. Delgado C, Baweja M, Crews DC, Eneanya ND, Gadegbeku CA, Inker LA, et al. A Unifying Approach for GFR Estimation: Recommendations of the NKF-ASN Task Force on Reassessing the Inclusion of Race in Diagnosing Kidney Disease. Am J Kidney Dis. 2022;79(2):268-88.e1.
4. Australian Bureau of Statistics. Australian Health Survey: Users' Guide, 2011-13 2017 [Available from: https://www.abs.gov.au/ausstats/abs@.nsf/PrimaryMainFeatures/4363.0.55.001?OpenDocument]
5. Pasricha S-R, Hasan MI, Braat S, Larson LM, Tipu SMM-U, Hossain SJ, et al. Benefits and Risks of Iron Interventions in Infants in Rural Bangladesh. New England Journal of Medicine. 2021;385(11):982-95.
6. UNC Chapel Hill. China Health and Nutrition Survey [Available from: https://www.cpc.unc.edu/projects/china.]
7. Freire WB. R-LM, Belmont P., Mendieta MJ., Silva-Jaramillo MK., Romero N., Sáenz K., Piñeiros P., Gómez LF., Monge R. Tomo I: Encuesta Nacional de Salud y Nutrición de la población ecuatoriana de cero a 59 años. ENSANUT-ECU 2012. In: Pública/Instituto MdS, Censos NdEy, editors. Quito-Ecuador2014.
8. Jaddoe VW, Mackenbach JP, Moll HA, Steegers EA, Tiemeier H, Verhulst FC, et al. The Generation R Study: Design and cohort profile. Eur J Epidemiol. 2006;21(6):475-84.
9. NHS Digital. Health Survey for England 2022 [Available from: https://digital.nhs.uk/data-and-information/publications/statistical/health-survey-for-england]
10. National Center for Health Statistics. National Health and Nutrition Examination Survey 2023 [Available from: https://www.cdc.gov/nchs/nhanes/index.html]
11. Carsley S, Borkhoff CM, Maguire JL, Birken CS, Khovratovich M, McCrindle B, et al. Cohort Profile: The Applied Research Group for Kids (TARGet Kids!). Int J Epidemiol. 2015;44(3):776-88.
12. Australian Bureau of Statistics. [Available from: https://www.abs.gov.au/ausstats/abs@.nsf/Lookup/D79750D8923E9049CA257AA30014B6A1?opendocument.]
13. Australian Bureau of Statistics. Microdata and TableBuilder: National Health Survey 2023
14. Australian Bureau of Statistics. Microdata and TableBuilder: Australian Health Survey: Nutrition and Physical Activity 2023
15. Yan S, Li J, Li S, Zhang B, Du S, Gordon-Larsen P, et al. The expanding burden of cardiometabolic risk in China: the China Health and Nutrition Survey. Obes Rev. 2012;13(9):810-21.
16. Mindell J, Biddulph JP, Hirani V, Stamatakis E, Craig R, Nunn S, et al. Cohort profile: the health survey for England. Int J Epidemiol. 2012;41(6):1585-93.
17. Erens B, P P. Health Survey for England ‘98. Series HS no. 8 London: The Stationery Office, 1999
18. Craig R MJe. Health Survey for England 2006. Series HS no. 16 London: National Centre of Social Research2008 [Available from: https://digital.nhs.uk/data-and-information/publications/statistical/health-survey-for-england/health-survey-for-england-2006-latest-trends.]

19. NHS Digital. Health Survey for England - 2006, CVD and risk factors for adults, obesity and risk factors for children 2008 [Available from: https://digital.nhs.uk/data-and-information/publications/statistical/health-survey-for-england/health-survey-for-england-2006-cvd-and-risk-factors-for-adults-obesity-and-risk-factors-for-children.]
20. Craig R, Hirani V. Health Survey for England 2009 Leeds: NHS Information Centre2010 [Available from: https://digital.nhs.uk/data-and-information/publications/statistical/health-survey-for-england/health-survey-for-england-2009-health-and-lifestyles]
21. NHS Digital. Health Survey for England - 2009, Health and lifestyles 2010 [Available from: https://digital.nhs.uk/data-and-information/publications/statistical/health-survey-for-england/health-survey-for-england-2009-health-and-lifestyles#resources.]
22. National Center for Health Statistics. National Health and Nutrition Examination Survey (NHANES) Appendix B: Metadata 2023 [Available from: https://www.epa.gov/sites/default/files/2015-05/documents/aceappendixb_nationalhealthandnutritionexaminationsurvey.pdf.]
23. National Health and Nutrition Examination Survey. 1999-2000 [Available from: https://wwwn.cdc.gov/nchs/nhanes/continuousnhanes/overview.aspx?BeginYear=1999.]
24. National Health and Nutrition Examination Survey. 2001-2002 [Available from: https://wwwn.cdc.gov/nchs/nhanes/continuousnhanes/overview.aspx?BeginYear=2001.]
25. National Health and Nutrition Examination Survey. 2003-2004 [Available from: https://wwwn.cdc.gov/nchs/nhanes/continuousnhanes/overview.aspx?BeginYear=2003.]
26. National Health and Nutrition Examination Survey. 2005-2006 [Available from: https://wwwn.cdc.gov/nchs/nhanes/continuousnhanes/overview.aspx?BeginYear=2005.]
27. National Health and Nutrition Examination Survey. 2007-2008 [Available from: https://wwwn.cdc.gov/nchs/nhanes/continuousnhanes/overview.aspx?BeginYear=2007.]
28. National Health and Nutrition Examination Survey. 2009-2010 [Available from: https://wwwn.cdc.gov/nchs/nhanes/continuousnhanes/overview.aspx?BeginYear=2009.]
29. National Health and Nutrition Examination Survey. 2015-2016 [Available from: https://wwwn.cdc.gov/nchs/nhanes/continuousnhanes/overview.aspx?BeginYear=2015.]
30. National Health and Nutrition Examination Survey. 2017-2018 [Available from: https://wwwn.cdc.gov/nchs/nhanes/continuousnhanes/overview.aspx?BeginYear=2017.]
31. TARGet Kids! [Available from: https://www.targetkids.ca]
32. Parkin PC, Hamid J, Borkhoff CM, Abdullah K, Atenafu EG, Birken CS, et al. Laboratory reference intervals in the assessment of iron status in young children. BMJ Paediatr Open. 2017;1(1):e000074.
33. CLSI. Defining, establishing, and verifying reference intervals in the clinical laboratory; approved guideline. CLSI document C28–A3. 2008.
34. Hickman PE, Koerbin G, Potter JM, Glasgow N, Cavanaugh JA, Abhayaratna WP, et al. Choice of Statistical Tools for Outlier Removal Causes Substantial Changes in Analyte Reference Intervals in Healthy Populations. Clinical Chemistry. 2020;66(12):1558-61.

35. Tukey JW. Exploratory Data Analysis. Addison-Wesley. 1977.
36. Daly CH, Higgins V, Adeli K, Grey VL, Hamid JS. Reference interval estimation: Methodological comparison using extensive simulations and empirical data. Clin Biochem. 2017;50(18):1145-58.
37. Box GEP, Cox DR. An Analysis of Transformations. Journal of the Royal Statistical Society Series B (Methodological). 1964;26(2):211-52.
38. Canty AJ, AC D. Resampling-based Variance Estimation for Labour Force Surveys. Journal of the Royal Statistical Society: Series D. The Statistician. 1999;48(3):379-91.
39. Efron B, R.J T. An Introduction to the Bootstrap (1st ed.). Hall/CRC Ca, editor. New York1994.
40. Woodruff RS. Confidence Intervals for Medians and Other Position Measures. Journal of the American Statistical Association. 1952;47:635-46.
41. Lumley T. Complex Surveys: A Guide to Analysis Using R. Sons JWa, editor2010.
42. Cochran WG. Sampling Techniques. 3rd Edition. John Wiley & Sons NY, editor. United States of America1977.
43. Koenker R BJG. Regression quantiles. Econometrica. 1978;46(1):33-50.
44. Viechtbauer W. Conducting Meta-Analyses in R with the metafor Package. Journal of Statistical Software. 2010;36(3):1 - 48.
45. Hoq M, Matthews S, Karlaftis V, Burgess J, Cowley J, Donath S, et al. Reference Values for 30 Common Biochemistry Analytes Across 5 Different Analyzers in Neonates and Children 30 Days to 18 Years of Age. 2019:1317.
46. Royston P, Wright EM. A Method for Estimating Age-Specific Reference Intervals (`Normal Ranges') Based on Fractional Polynomials and Exponential Transformation. Journal of the Royal Statistical Society Series A (Statistics in Society). 1998;161(1):79-101.
47. Benner A. mfp: Multivariable fractional polynomials. R News. 2005;5(2):20-3.
48. Sauerbrei W, Meier-Hirmer C, Benner A, Royston P. Multivariable regression model building by using fractional polynomials: Description of SAS, STATA and R programs. Computational Statistics & Data Analysis. 2006;50(12):3464-85.
49. Ambler G, Royston P. Fractional polynomial model selection procedures: Investigation of type I error rate. Journal of Statistical Computation and Simulation. 2001;69(1):89-108.
50. Chernick MR. An introduction to bootstrap methods with applications to R: Wiley; 2011.
51. Davison AC, Hinkley DV. Bootstrap Methods and their Application. Cambridge: Cambridge University Press; 1997.
52. Chen M-H, Raffield LM, Mousas A, Sakaue S, Huffman JE, Moscati A, et al. Trans-ethnic and Ancestry-Specific Blood-Cell Genetics in 746,667 Individuals from 5 Global Populations. Cell. 2020;182(5):1198-213.e14.
53. Wheeler MM, Stilp AM, Rao S, Halldórsson BV, Beyter D, Wen J, et al. Whole genome sequencing identifies structural variants contributing to hematologic traits in the NHLBI TOPMed program. Nat Commun. 2022;13(1):7592.

54. Finer S, Martin HC, Khan A, Hunt KA, MacLaughlin B, Ahmed Z, et al. Cohort Profile: East London Genes & Health (ELGH), a community-based population genomics and health study in British Bangladeshi and British Pakistani people. International Journal of Epidemiology. 2020;49(1):20-1i.
55. Wang Q, Dhindsa RS, Carss K, Harper AR, Nag A, Tachmazidou I, et al. Rare variant contribution to human disease in 281,104 UK Biobank exomes. Nature. 2021;597(7877):527-32.
56. Genomics England. Rare anaemia (Version 3.0) 2023 [Available from: https://nhsgms-panelapp.genomicsengland.co.uk/panels/518/v3.]
57. Karczewski KJ, Francioli LC, Tiao G, Cummings BB, Alföldi J, Wang Q, et al. The mutational constraint spectrum quantified from variation in 141,456 humans. Nature. 2020;581(7809):434-43.
58. Collins RL, Brand H, Karczewski KJ, Zhao X, Alföldi J, Francioli LC, et al. A structural variation reference for medical and population genetics. Nature. 2020;581(7809):444-51.
59. StataCorp. Stata Statistical Software: Release 16. College Station, TX: StataCorp LLC.; 2019.
60. R Core Team. R: A language and environment for statistical computing. Vienna, Austria: R Foundation for Statistical Computing; 2022.
61. StataCorp. Stata Statistical Software: Release 17. College Station, TX: StataCorp LLC.; 2021.
62. R Core Team. R: A language and environment for statistical computing. Vienna, Austria: R Foundation for Statistical Computing; 2021.
63. Conway JR, Lex A, Gehlenborg N. UpSetR: an R package for the visualization of intersecting sets and their properties. Bioinformatics. 2017;33(18):2938-40.
64. Gu Z, Eils R, Schlesner M. Complex heatmaps reveal patterns and correlations in multidimensional genomic data. Bioinformatics. 2016;32(18):2847-9.
65. Hu Y, Stilp AM, McHugh CP, Rao S, Jain D, Zheng X, et al. Whole-genome sequencing association analysis of quantitative red blood cell phenotypes: The NHLBI TOPMed program. Am J Hum Genet. 2021;108(5):874-93.
